# Transcriptome Analysis of Tomato Flower Pedicel Tissues Reveals Abscission Zone-Specific Modulation of Key Meristem Activity Genes

**DOI:** 10.1371/journal.pone.0055238

**Published:** 2013-02-04

**Authors:** Xiang Wang, Danmei Liu, Aili Li, Xiuli Sun, Rongzhi Zhang, Liang Wu, Yanchun Liang, Long Mao

**Affiliations:** 1 National Center for Wheat Research, Henan Agricultural University, Zhengzhou, People’s Republic of China; 2 National Key Facility for Crop Gene Resources and Genetic Improvement, Institute of Crop Science, MOA Key Lab for Germplasm and Biotechnology, Chinese Academy of Agricultural Sciences (CAAS), Beijing, People’s Republic of China; 3 Key Laboratory of Symbol Computation and Knowledge Engineering of Ministry of Education, College of Computer Science and Technology, Jilin University, Changchun, People’s Republic of China; UMass, United States of America

## Abstract

Tomato flower abscises at the anatomically distinct abscission zone that separates the pedicel into basal and apical portions. During abscission, cell separation occurs only at the abscission zone indicating distinctive molecular regulation in its cells. We conducted a transcriptome analysis of tomato pedicel tissues during ethylene promoted abscission. We found that the abscission zone was the most active site with the largest set of differentially expressed genes when compared with basal and apical portions. Gene Ontology analyses revealed enriched transcription regulation and hydrolase activities in the abscission zone. We also demonstrate coordinated responses of hormone and cell wall related genes. Besides, a number of ESTs representing homologs of key Arabidopsis shoot apical meristem activity genes were found to be preferentially expressed in the abscission zone, including *WUSCHEL* (*WUS*), *KNAT6*, *LATERAL ORGAN BOUNDARIES DOMAIN PROTEIN 1*(*LBD1*), and *BELL*-like homeodomain protein 1 (*BLH1*), as well as tomato axillary meristem genes *BLIND* (*Bl*) and *LATERAL SUPPRESSOR* (*Ls*). More interestingly, the homologs of *WUS* and the potential functional partner *OVATE FAMILIY PROTEIN* (*OFP*) were subsequently down regulated during abscission while *Bl* and *AGL12* were continuously and specifically induced in the abscission zone. The expression patterns of meristem activity genes corroborate the idea that cells of the abscission zone confer meristem-like nature and coincide with the course of abscission and post-abscission cell differentiation. Our data therefore propose a possible regulatory scheme in tomato involving meristem genes that may be required not only for the abscission zone development, but also for abscission.

## Introduction

Organ abscission is a ubiquitous physiological process in the plant kingdom. While seasonal senescence and foliage abscission set the enchanted autumn scenery, the dispersal of seeds and the efficiency of grain threshing and fruit picking are highly relevant to agriculture. Abscission occurs at specific sites called abscission zones. Up to date, a vast number of genes, particularly hydrolytic enzymes, such as polygalacturonases (PGs), xyloglucan endotransglucosylase/hydrolase (XTH), β-1,4-glucanase (cellulase), and expansins have been shown to be implicated in abscission for the degradation of cell wall and middle lamella at the abscission zones that eventually result in the shedding of distal organs [1], [2], [3], [4], [5], [6].

During this process, ethylene is known to be an efficient accelerator for organ abscission, although the initiation of abscission is considered to be timed or repressed by auxin [6], [7], [8], [9]. In this regard, abscission zones are made up with so-called type II cells whose expansive growth can be enhanced by ethylene, but not auxin [7]. Despite the dominant role of ethylene in plant organ abscission, genes whose function in abscission is independent of ethylene are increasingly reported, especially in Arabidopsis. For instance, HAESA and HSL2 (HAESA-like 2) are receptors of the small peptide ligand IDA (INFLORESCENCE DEFICIENT IN ABSCISSION) and together modulate the downstream MAPK cascade to induce floral organ shedding in an ethylene-independent manner [10]. Other genes contributing to the abscission zone development and its capacity are transcription factors (TFs) including MADS-box genes *AGL15* and *AGL18* [11], *KNAT/BP* [12], and *AtZFP2* (*ZINC FINGER PROTEIN2*) [13]. Ethylene-independent mechanisms are proposed to be involved in the establishment of the abscission zone that is subsequently responsive to ethylene signals, either through guiding the correct architecture formation of the abscission zone itself, or by facilitating the efficacy of ethylene signaling pathways [14], [15], [16]. Thus, an intrinsic relation between the development of the abscission zone and its function is plausible.

The tomato flower abscission zone is located in the middle of the pedicel dividing it into two distinct sections: the basal and the apical portions. The abscission zone is composed of a few layers of smaller cells lacking vacuoles and any aspect of maturation [17], indicating that cell growth and differentiation is arrested at an early stage of development [18], [19]. In this regard, the persistent expression of KNOX family genes in the abscission zone were suggested [20]. In tomato, a few prominent genes have been found to affect the abscission zone development, including *JOINTLESS* (*J*), *LATERAL SUPPRESSOR* (*Ls*), and *JOINTLESS2* (*J2*) [21], [22], [23]. *BLIND* (*Bl*), encoding a R2R3-class Myb transcription factor, genetically interacts with *J* [24], [25], [26], suggesting a possible role of this gene in abscission. In Arabidopsis, the *Bl* homolog *REGULATOR OF AXILLARY MERISTEMS1* (*RAX1*) is also required for lateral meristem initiation [27], [28]. Furthermore, mutations of *RAX* genes affect the expression domains of the meristem activity gene *SHOOTMERISTEMLESS* (*STM*) which works with *KNAT6*, both KNOX-family genes, in shoot apical meristem maintenance and organ separation [29]. Recently, KNAT6, together with KNAT1 and 2 were shown to act downstream in the IDA-HAE/HSL2 signaling pathway to regulate floral organ abscission [30]. Therefore, a picture of regulatory network involving meristem activity genes in abscission is emerging.

In tomato, two recent transcriptome analyses were performed using pedicels as materials. Meir *et al.* (2010) compared the transcriptome differences between the abscission zone and the basal portion of pedicel in a time course after flower removal with or without the treatment of 1-methylcyclopropene (1-MCP), an effective inhibitor of ethylene action, while Nakano *et al.* (2012) compared the whole pedicel of the wild type and the jointless mutant or *MACROCYLYX* (*MC*) knock-down transgenic lines at pre-abscission stage under normal growth conditions. Although it has long been observed that application of exogenous ethylene accelerates tomato flower abscission, a comparative analysis of the transcriptome profiles in abscission zone relative to the neighboring tissues (the basal and apical portions) in abscission is still lacking. We performed a microarray assay on three pedicel tissues at 0, 3, 6 h time points of ethylene treatment. Our results showed that homologs of meristem activity genes were preferentially expressed in abscission zone when compared with the neighboring tissues. We also show that some of these genes were subsequently repressed in the abscising tissues. The expression patterns of meristem activity genes support the idea that they may play a role in maintaining meristem-like status of abscission zone cells, correlating with their performance before and after abscission.

## Results

### The Experimental Design

**Figure 1:**
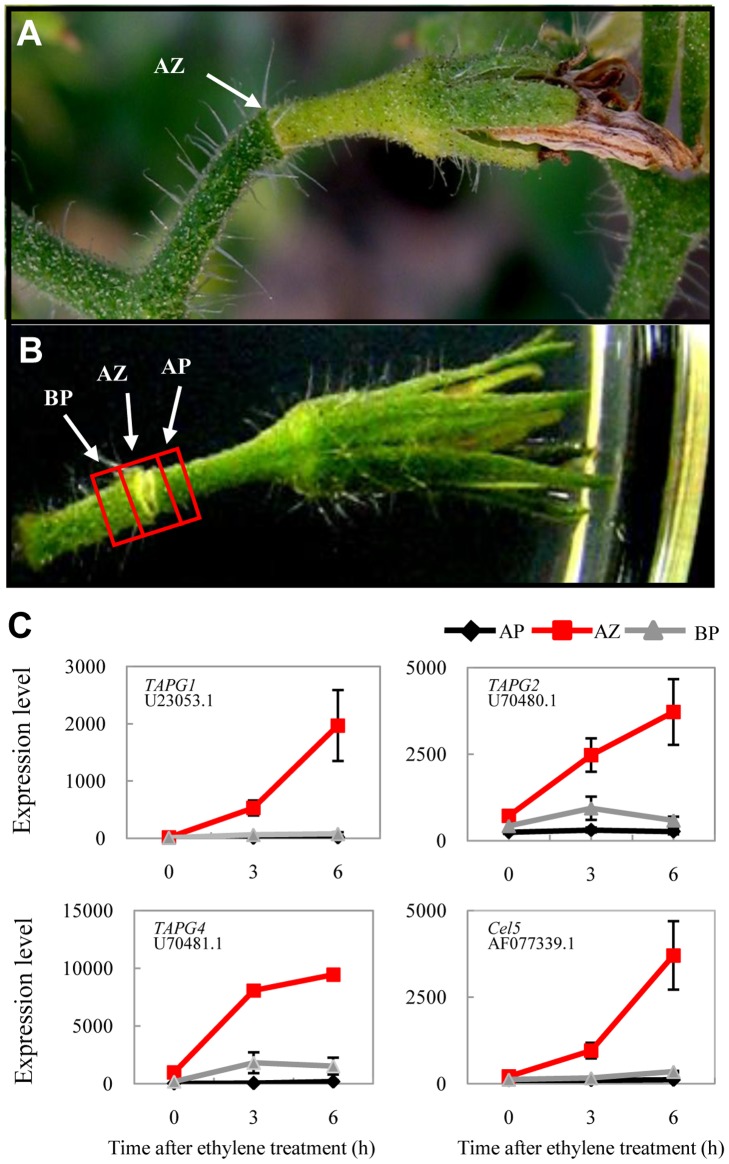
A tomato flower in abscission. A, The morphological change of a tomato pedicel during natural flower senescence; Note the color changes at abscission zone (AZ) and its apical portion while the basal portion remains green. B, An abscising floral explant on the ½ MS media under ethylene treatment, showing the portions of tissues collected for microarray analysis. BP, basal portion; AP, apical portion. C, Induction of cell wall degradation marker genes from microarray analysis in the tomato abscission zone during ethylene-promoted abscission. Marker genes include *TAPG1*, *TAPG2*, *TAPG4*, and *Cel5*, with corresponding GenBank accession number indicated below. Error bars derived from three biological replicates.

During the natural senescence of a tomato flower, the abscission zone and the apical portion become yellowish, withered, and eventually separated along the abscission zone whereas the basal portion often remains green (Fig.1A; [16], [31]). It has been shown that application of 10 µl/L ethylene can initiate abscission of floral explants within 6 hour (h) while in the absence of ethylene abscission does not occur for up to 5 days [32]. We adopted a higher ethylene concentration (50 µl/L) to treat pre-anthesis floral explants (see Materials and Methods). Under our condition, the abscission zone started to lose green color after about 6 h. To study the early molecular events during tomato pedicel abscission, we manually collected ∼1.5 mm of the abscission zone and neighboring tissues at 0, 3, and 6 h ethylene treatment and froze them immediately in the liquid nitrogen (Fig. 1B). RNAs were extracted and applied in microarray analyses using Affymatrix GeneChip Tomato Genome Array that consists more than 10,000 tomato probe sets from around 9,000 ESTs (or genes). Initial assessment showed that previously characterized abscission zone-specific genes, such as tomato abscission polygalacturonase 1 (*TAPG1*, U23053.1), *TAPG2* (U70480.1), *TAPG4* (U70481.1), and the cellulase gene *Cel5* (AF077339.1) were specifically induced in the abscission zone under our experimental condition (Fig. 1C), indicating that application of exogenous ethylene had indeed caused changes in internal gene expressions. Twenty five genes with various expression patterns in the three tissues as detected by microarrays were further validated using RT-PCR assays. Except two genes (AI777697 and BT012857.1), all the other 23 genes showed similar expression patterns as detected by the microarray, giving a consistency rate of >92% (Table S1). To assess the effects of wounding incurred during the preparation of flower explants and the subsequent experiment, we chose 68 genes and tested their expression patterns under 6 h air treatment using real time PCR. As shown in Table S2, it seems that wounding to some extent affects expressions of some of the genes under air condition. However, considering the high exogenous ethylene concentration applied, the effect of wounding can be largely ignored. In the following sections, analyses were performed on differentially expressed genes with a fold change ≥2 and a false discovery rate q ≤0.01 at either 3 h and/or 6 h ethylene-treatment when compared with their expression levels at 0 h or between respective pedicel tissues.

### Differential Transcriptome Responses of Three Tomato Pedicel Tissues during Abscission

**Figure 2:**
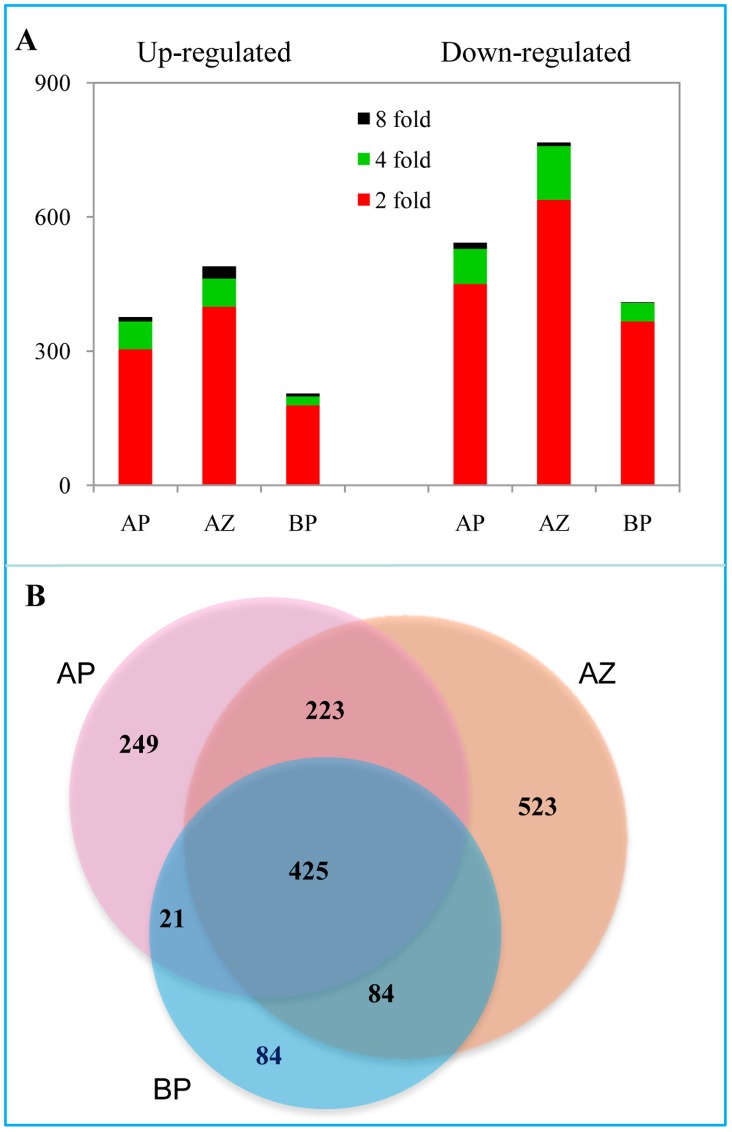
Transcriptome responses of different tissues of tomato pedicels during ethylene-promoted abscission. A, The total numbers of genes differentially expressed (fold changes ≥2, 4, 8; *p*<0.05) at 3 h and/or 6 h after ethylene treatment. B, Venn diagram showing the overlapping of differentially expressed genes in the abscission zone and flanking tissues (the basal portion and the apical portion). Lists of genes in each tissue are presented in Tables S3,S4,S5.

**Figure 3:**
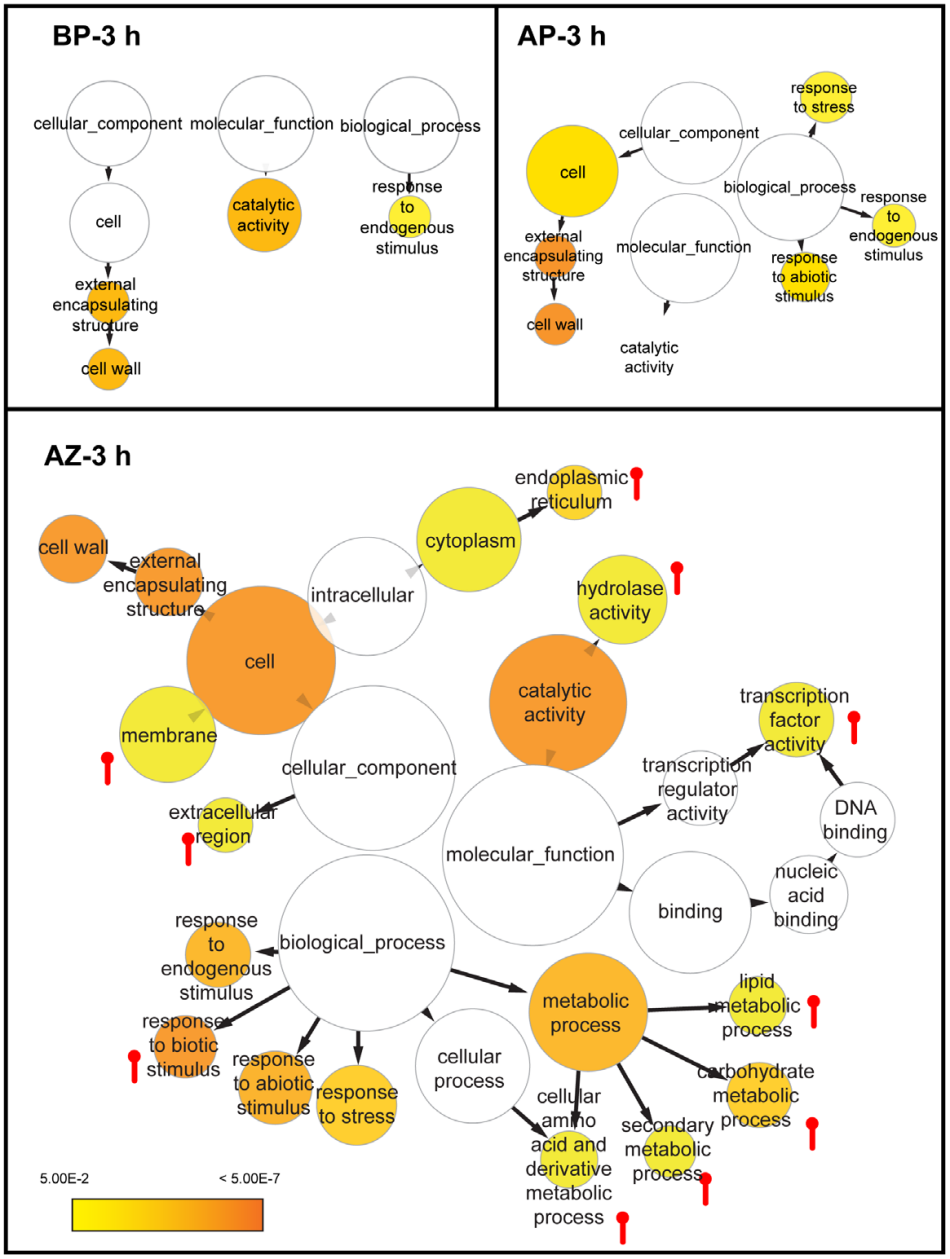
Enriched GO terms among genes differentially expressed in abscission zone after 3 h ethylene treatment. A Cytoscape view of enriched GO terms in BinGO where categories in GoSlimPlants (Maere *et al*., 2005) were used to simplify the analysis. The color bar shows the statistical significance, with enrichment significance level *P*<0.05, and the false discovery rate *FDR* <0.05. The size of the node is proportional to the number of genes in the GO category. The pin-like symbols indicate enriched GO terms specifically in the abscission zone relative to the basal portion and the apical portion.

A total of 1,255 ESTs (or in general “genes”) were found to be differentially expressed in the abscission zone, much more than those in the basal portion (614 ESTs) and the apical portion (918 ESTs; Table S3,S4,S5). In all three tissues, more genes were down regulated than up regulated, mostly at the range of 2 fold change (Fig. 2A). A total of 425 differentially expressed genes were shared in all three tissues that may represent the common ethylene responsive genes (Fig. 2B). More genes (41% of the total) were specifically regulated in the abscission zone than in the apical portion (27%) and the basal portion (13.7%) respectively. These numbers demonstrate that the abscission zone is the most active site in gene expression during abscission. Gene Ontology (GO) enrichment analyses showed that the Cellular Component (CC) term “cell wall” and the Molecular Function (MF) term “catalytic activity” were enriched among differentially expressed genes of all three tissues, suggesting a general effect of ethylene on cell wall structures and catalytic enzymes, while the Biological Process (BP) term “response to abiotic stimulus” was shared only by genes from the abscission zone and the apical portion (Fig. 3). The abscission zone conferred several specifically enriched GO terms, including CC terms “membrane”, “extracellular region”, and “endoplasmic reticulum”; MF terms “hydrolase activity” and “transcription factor”; and BP terms “response to biotic stimulus” and various metabolic processes. These observations indicate that the abscission zone is distinctively more sensitive to ethylene than its neighboring tissues during abscission.

### Clustering Analysis of Ethylene Responsive Genes in the Abscission Zone

**Figure 4:**
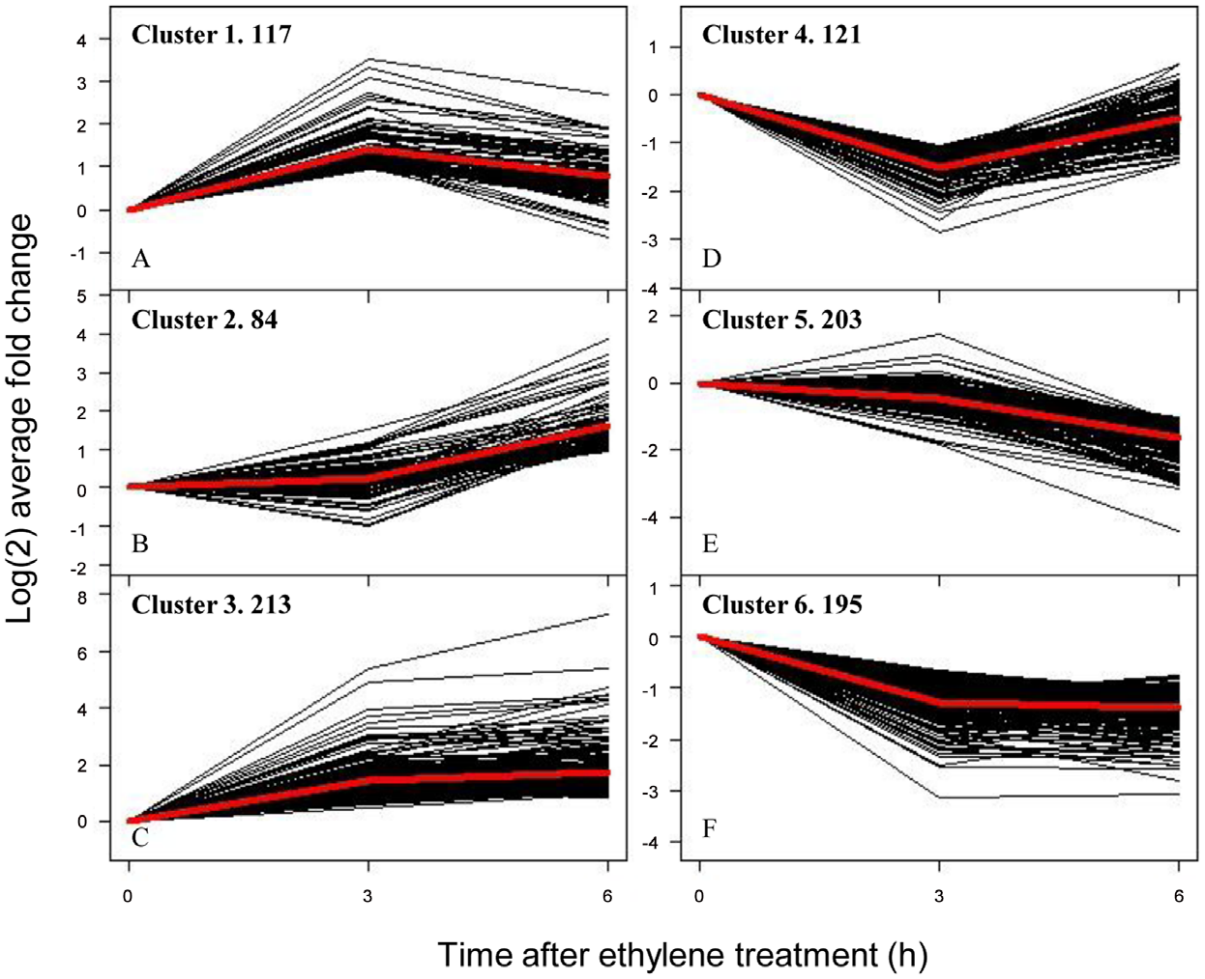
*K* means clustering of 933 non-redundant differentially expressed genes. Genes in clusters 1–3 are generally induced while genes in clusters 4–6 are repressed.

Based on the similarity of the kinetic expression patterns we classified 933 non-redundant differential expression genes of the abscission zone into six clusters. Cluster 1 comprises 117 genes that were transiently induced at 3 h and were then repressed to the initial expression level at 6 h (Fig. 4A). MapMan classification revealed genes involved in amino acid metabolism, development and transcription, and ethylene signaling genes (Table S6). The tomato *AGL15* homolog (BG725110) was significantly induced at 3 h in both the abscission zone and the apical portion and was slightly up regulated in the basal portion (Fig. S1). Cluster 2 contains 84 genes that were not induced until 6 h after ethylene treatment (Fig. 4B). Hormone related genes were featured in this cluster, indicating a delay in non-ethylene hormone responses during abscission. Meanwhile, pectate lyases (2), expansins (2), and peroxidases (4) seem to become the major cell wall modification proteins. Cluster 3 genes (213) were induced immediately at 3 h and most maintained high expression levels at 6 h (Fig. 4C). It was the largest group among the six clusters comprising nearly half of all up regulated genes. This cluster was featured by genes encoding TFs, biotic/abiotic responses, ethylene signaling/biogenesis, and cell wall degradation enzymes. The varieties of TFs include C2C2 zinc finger protein (5 genes), AP2 domain (2), WRKYs (2), as well as the MADS-box gene *AGL12* homolog (AY098737.2) and *Bl*. The presence of two ethylene signaling genes (homologs of Arabidopsis *EIN4* and *AtERF-1*) and three synthesis-degradation genes (X58885.1, AW223067, AJ715790.1) indicates rapid responses of ethylene related genes.

Clusters 4, 5, and 6 contain genes that were largely suppressed during abscission (Fig. 4D–F). Again, genes associated with “RNA transcription” were predominant in all three clusters. In cluster 4 (121 genes), for instance, there were 10 RNA transcription associated genes, including Arabidopsis *LBD4* (*LATERAL ORGAN BOUNDARIES* domain protein 4; AI774397) and *AS1* (*ASYMMETRIC LEAVES 1*; AF148934.1) homologs. In tomato pedicel, *AS1* was clearly down regulated at the abscission zone during abscission, much significantly than in the apical portion and the basal portion (Table S6). Cluster 4 also contains 10 cell wall modification genes, including four genes similar to Arabidopsis expansins A6, A8, A10, and A13. Cluster 5 genes (203) were not repressed until 6 h. There were 21 photosynthesis related genes, 17 of which were for the light reaction in the light harvest center whereas the other seven were enzymes for the Calvin cycle. The steady and profound repression of photosynthesis related genes explains the pale color of the abscission zone tissues during abscission when the basal portion and the apical portion still remain green (Fig. 1B). Cluster 6 consists of 195 genes that were characterized with 14 RNA transcription/regulation genes, including the putative ortholog of Arabidopsis *SHOOT MERISTEMLESS* (*STM*, AF000141.1) and the tomato *KNOTTED 2* gene (U76408), a homolog of *KNAT6*. The rapid repression of the KNOX genes may imply possible involvement of meristem gene regulation in tomato pedicel abscission [29].

### Responses of Hormone and Cell Wall Related Genes

**Figure 5:**
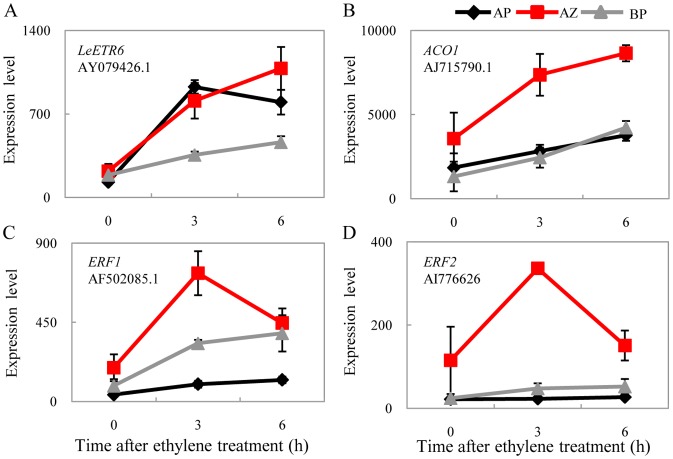
Response of ethylene related genes. Induction of (A) the ethylene receptor *LeETR6*, (B) ethylene biosynthesis gene 1-aminocyclopropane-1-carboxylate oxidase gene (*ACO1*), and (C-D) two ERF transcription factors during ethylene-promoted abscission.

To distinguish the effect caused by endogenous ethylene concentration changes over the general responses to exogenous ethylene treatment, we mostly focused on genes with expression patterns distinguishable among the three tissues. We found that a third (12 out of 37) of differentially expressed hormone genes in the abscission zone was for ethylene, but most of them also differentially expressed in the basal portion and/or the apical portion. There were two ethylene receptors. LeETR6 (AY079426.1) was continuously induced by ethylene in the abscission zone, from 3 h to 6 h (3.67 and 4.89 fold respectively; Fig. 5A). It was more significantly induced in the apical portion (7.18 and 6.19 fold at 3 h and 6 h respectively) and was marginally induced (1.89 and 2.46 fold respectively) in the basal portion. In contrast, LeETR2 (AF043085.1) was repressed in the abscission zone (0.38 and 0.48 fold at 3 h and 6 h respectively; Table S6). Interestingly, LeETR4, which exhibited significant induction in the flower removal experiment [8], was not significantly regulated in our study. In spite of this, one 1-aminocyclopropane-1-carboxylate oxidase gene (*ACO1*; AJ715790.1) was specifically induced in the abscission zone (Fig. 5B), together with two ERFs (ethylene response factor; AF502085.1, AI776626; Fig. 5C & D), indicating that the specificity of ethylene functions is probably achieved by downstream effective genes.

**Figure 6:**
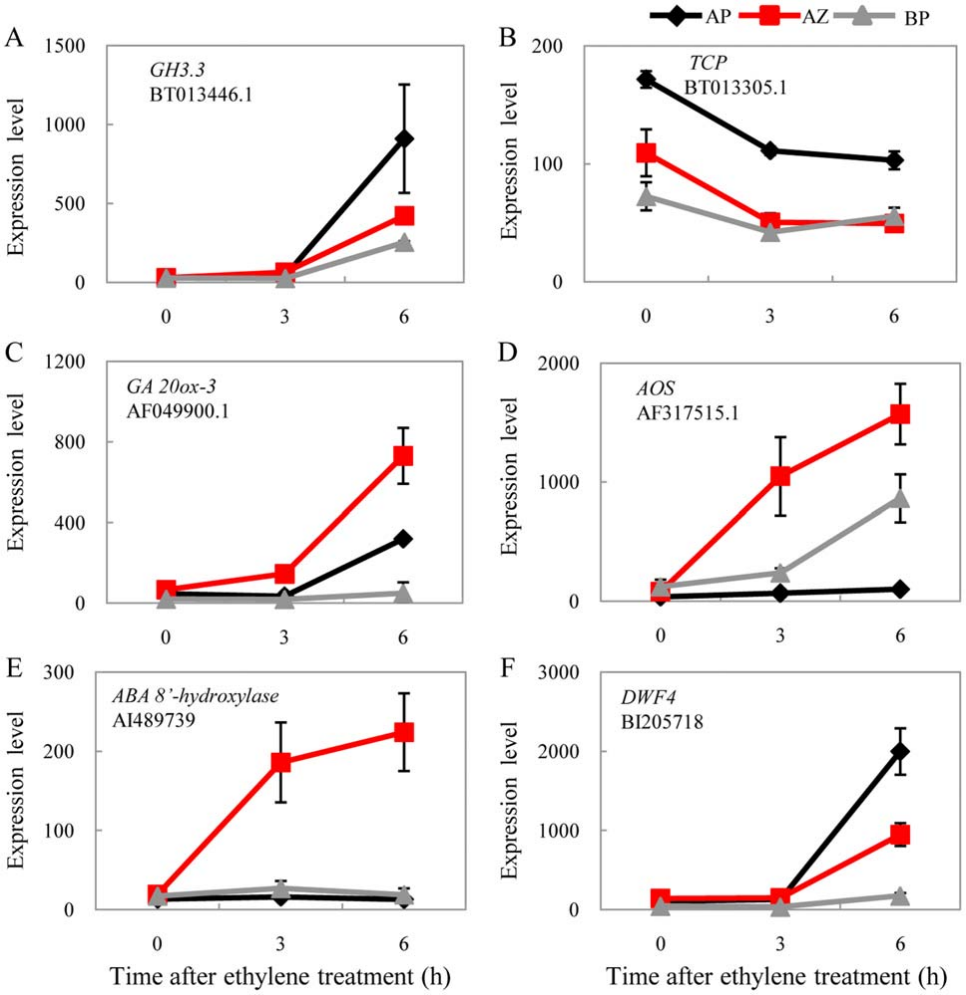
Coordinated expression of auxin and other hormone related genes. Note the formation of distal to proximal gradient expression pattern for *GH3.3* (A), *TCP* (B), and *DWF4* (F). *GA 20ox-3* (C), *AOS* (D), and *ABA 8′-hydoxylase* (E) genes were most highly expressed in the abscission zone during abscission.

Similar tissue specific patterns were also found for genes related to auxin, the functionally antagonistic hormone of ethylene during abscission which is considered to be generated in the flower and transported along the pedicel [8]. The homolog of the Arabidopsis auxin homeostasis gene *GH3.3* (BT013446.1) that encodes the indole-3-acetic acid amido synthetase for IAA-amino acid conjugation was induced 33, 14, and 9 fold in the apical portion, the abscission zone, and the basal portion respectively after 6 h ethylene treatment. Such a distal to proximal expression gradient along the pedicel indicates a negative regulation on auxin levels in the corresponding tissues (Fig. 6A), consistent with the repression patterns of the auxin regulated TCP transcription factor (BT013305.1; Fig. 6B). Except for the induction of ABA 8′-hydroxylase *CYP707A3* gene homolog (AI489739) that may promote increased ABA breakdown specifically in the abscission zone, abscission induced differential responses in rate limiting genes were found for gibberellin (*gibberellin 20-oxidase-3*, AF049900.1), jasmonic acid (*ALLENE OXIDE SYNTHASE*, or *AOS*; AF317515.1), and brassinosteroid (*DWF4*, BI205718) in the three pedicel tissues (Fig. 6C–F). It has to be noted that *ACO*1 and *DWF4* were also strongly induced by wounding (Table S2).

Cell wall and middle lamella degradation is one of the major targets of abscission. Despite the overall complexity in expression patterns for cell wall modification genes, three PGs and one cellulase were specifically induced in abscising abscission zones (Fig. 1C), while three *XTHs* were induced with two of them *XTH8* (X82684.1) and *SlXTH* (AY497476.1) only transiently at 3 h (Fig. S2). Two expansins, *EXP11* (AJ560646.1) and the Arabidopsis *AtEXPa4* homolog (AF548376.1) were later induced significantly at 6 h, together with one pectin lyase gene (BT012714.1) that is involved in cell wall enlargement and pectin re-modification [33], [34], [35]. Thus, our data confirmed previous reports how cell wall modification genes respond and corroborated with the functions of ethylene in abscission.

### Abscission Zone-preferential Expression of Key Meristem Protein Complex Genes

Expression domains may indicate unique functions of a gene in a particular tissue. A total of 107 genes were specifically expressed in the abscission zone relative to their expression levels in the apical portion and the basal portion (Table 1 & Table S7). Among them, *WUSCHEL* (*WUS*), *Bl*, and *Ls* also displayed differential expressions between wild type and *mc* mutant (and hence jointless) pedicels [36]. What was unexpected was the abscission zone specific expression patterns for all three homologs of Arabidopsis *KNAT6* (U76408), *BELL*-like homeodomain protein 1 (*BLH1*, AF375967), and *OFP* (*OVATE FAMILY PROTEIN*, AY140893) whose family members can potentially form ternary complexes critical for meristem activities [37], [38], [39], [40]. Homologs of a *LATERAL ORGAN BOUNDARIES DOMAIN PROTEIN 1*(*LBD1*) and *Myb78* were also highly expressed in the abscission zone. In addition, the expression of the homolog for the second key meristem gene *STM* was the highest in the abscission zone, but was also expressed in the basal portion and the apical portion (Table S7). Since these genes have been shown to interact with each other in Arabidopsis shoot apical meristem and axillary meristem, their preferential expressions in the abscission zone support the idea that meristem activity genes play roles in maintaining the undeveloped status of tomato abscission zone cells [16]. On the contrary, the expression of two MADS-box genes *TAG1* (the tomato *AGAMOUS*) and *TDR8* (the Arabidopsis *SHATTERPROOF 2* homolog) were excluded from the abscission zone (Table 1). And so was a *SQUAMOSA-*promoter binding protein gene *LeSPL3* (BI929558) that could potentially regulate the abscission development gene *MC*. The sequestration of *TAG1* and *TDR8* expressions in the abscission zone, in contrast to the requirement of two additional MADS-box genes *J* and *MC*, demonstrates diverse roles of MADS-box genes in abscission zone functions.

**Table 1:** List of abscission zone-specific transcription factors.

Probe Set ID	GenBank#	Annotation	Arabidopsis homolog^1^	Fold change		Raw readings		
				AZ:AP	AZ:BP	AP	AZ	BP
**Preferentially expressed** **in AZ**								
Les.3693.1.S1_at	AF426174	BLIND		24.79	9.73	14.9	368.41	37.88
LesAffx.62669.2.A1_at	AW442297	LOB domain protein 1	LBD1(AT1G07900)	20.35	3.15	34.2	696.58	221.31
Les.4136.1.S1_at	AJ538329	WUSCHEL		16.62	15.32	61.2	1016.3	66.33
Les.3571.1.S1_at	U76408	KNOTTED 2 protein	KNAT6 (AT1G23380)	10.86	2.05	51.4	558.11	271.83
Les.4309.1.S1_at	AF375967	BELL-like homeodomainprotein 4	BLH1(AT2G02850)	10.61	4.29	30.5	323.88	75.47
Les.69.1.S1_at	AF098674	LATERAL SUPPRESSOR		8	7.73	17	135.83	17.58
Les.3819.1.S1_at	AY140893	OVATE	AT2G18500	4.3	3.34	260	1120.7	335.53
LesAffx.62138.1.S1_at	AW737374	MYP Cpm10	MYB78 (AT5G49620)	2.63	2.7	110	289.36	107.14
**Preferentially expressed** **in AP and BP**								
Les.46.1.S1_at	BT014391	TDR8	SHP2 (AT2G42830)	0.49	0.3	148	71.98	238.13
Les.3065.1.S1_at	BI929558	SQUAMOSA PROMOTER-BINDING PROTEIN LIKE protein	SPL3 (AT2G33810)	0.32	0.5	965	309.75	618.07
LesAffx.53591.1.S1_at	AI899018	MYB, methyl jasmonateinduced	MYB13 (AT1G06180)	0.31	0.5	390	121.56	242.3
Les.3620.1.S1_at	L26295	TAG1		0.31	0.47	335	102.7	220.81

1 Best Arabidopsis homolog if not functional studied in tomato; See text for tomato gene details.

### Transcription Repression of Key Meristem Activity Genes in Abscission Zone during Abscission

**Figure 7:**
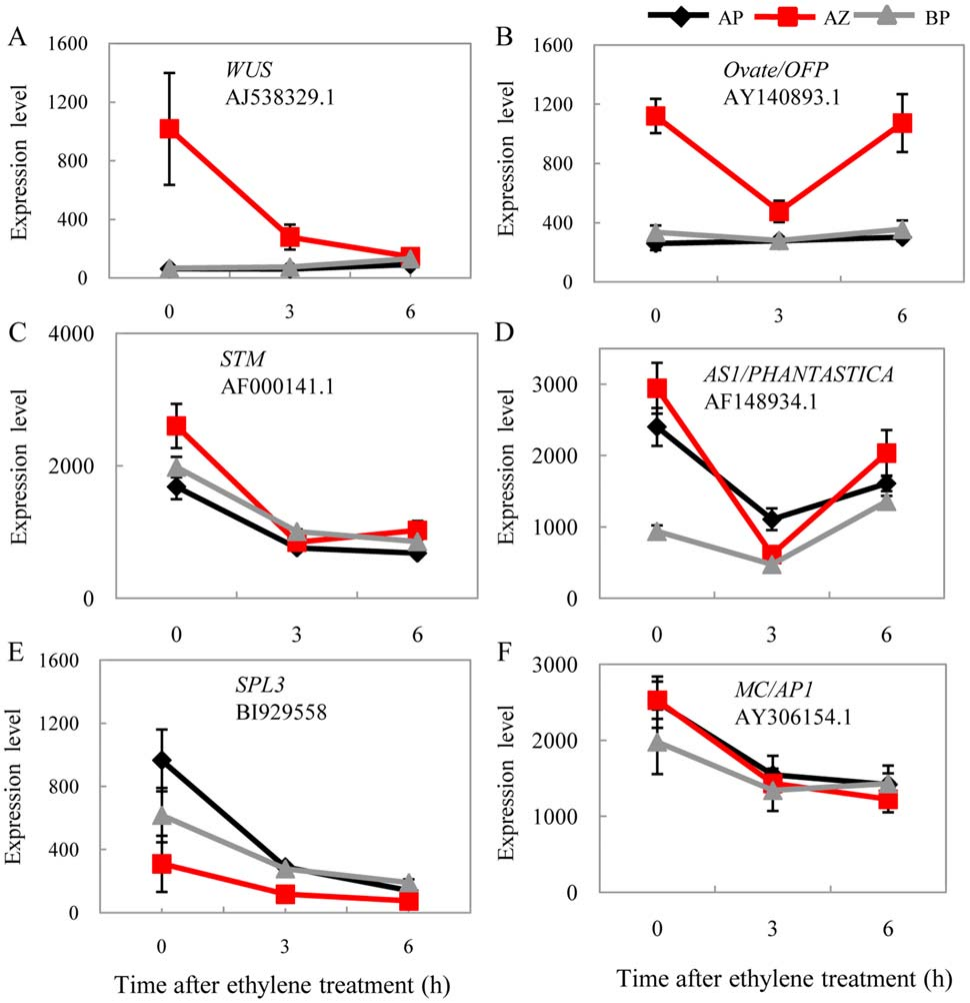
Repression of meristem activity genes during abscission. Homologs of *WUS* (A) and *OVATE* (B) are specifically repressed in the abscission zone. *STM* (C) and *AS1* (D) were repressed in the abscission zone, but also in one or more additional tissues. E–F show the repression of the *SPL3* homolog and the tomato *MC* gene, a putative ortholog of the Antirrhinum *SQUAMOSA* gene.

**Figure 8:**
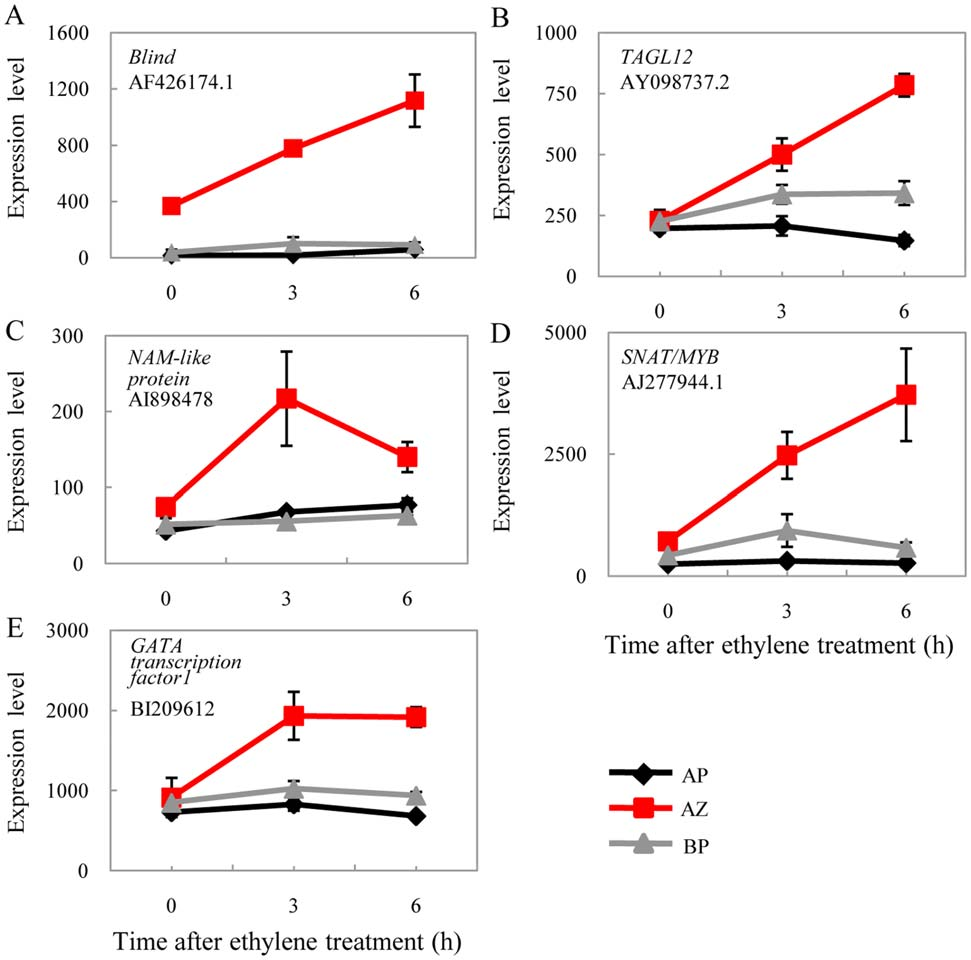
Induction of transcription factor genes during abscission. (A) *Blind* is specifically expressed in the abscission zone and is further induced significantly during the course of abscission. (B-E) *De novo* significant induction of four transcription factors.

Despite their preferred expressions in the abscission zone, homologs of *WUS* and the putative interacting partner *OFP* were clearly repressed during abscission (Fig. 7A & B), while their expressions in the basal portion and the apical portion remained unchanged. The tomato *WUS* displayed strong tissue-specific expression in the abscission zone, 15 fold higher than both the basal portion and the apical portion. The expression of *WUS* was reduced to the basal level as in the basal portion and the apical portion after 6 h ethylene-promoted abscission. As a matter of fact, *STM* was also significantly repressed during abscission, but in all three of the tissues. And so was the *AS1* (*ASYMETRIC LEAVES1*; Fig. 7C & D). In Arabidopsis, all these genes were found to be functionally related in the shoot apical meristem and the axillary meristem that regulate (or being regulated by) KNOX genes [38]. The co-regulation of these genes in tomato pedicel abscission zones and during abscission support the under developed meristem status of the abscission zone cells [16] and may be also necessary for post-abscission cell differentiation [41]. Except for the KNOX genes, the expression of the *SPL3* homolog was also further repressed in all three tissues, together with *MC*, the *APETALA1* (*AP1*) and *SQUAMOSA* (*SQUA*) orthologs and potential regulating target [42], but not in the abscission zone-specific manner (Fig. 7E & F). In monocots, a SPL gene *LIGULELESS 1* (*LG1*) and its orthologs have been found to be required for the proper development of auricles of grass plants (maize, [43]; barley, [44]; rice, [45]), which define regions equivalent to tomato abscission zones. These data therefore prompt speculation of a common *SPL* regulatory pathway for the abscission zone in both monocots and eudicots, which deserves further investigation. In contrast, *TAGL12* and *Bl* were continuously and specifically induced in the abscission zone during ethylene treatment, together with three other transcription factors (Fig. 8). Expression patterns of these genes coincide with abscission genes such as those for hormone biogenesis and signaling and cell wall modifications. The repression of meristem genes and induction of various transcription factors may indicate a certain regulatory relationship among them.

## Discussion

Organ abscission is one of the key steps in the plant life cycle. Both promoted abscission, such as in cotton defoliation and mechanical citrus harvest, and delayed abscission, such as in most vegetables and horticultural crops, are desirable for farmers to provide higher quality products [15]. The effect of ethylene on regulating plant organ abscission has long been established [46] and applied in horticulture and agriculture practices. But comparative transcriptome profiling of the three pedicel tissues is still not available. The better understanding of the molecular events associated with the abscission zone development and physiology may provide new insights in the basic biology of abscission and assist its manipulation in an agriculturally favorable manner.

### Experimental Approaches to Study Abscission Zone-specific Gene Profiles

The distinct location of the tomato flower abscission zone makes it one of the most convenient materials for abscission study. Our work, together with Meir *et al.* (2010) and Nakano *et al.* (2012), demonstrate that results derived from manually collected abscission zone tissues were reliable and repeatable. However, despite a large number of genes were differentially expressed in each experiment, only a portion of them were co-regulated under both conditions, indicating that different strategies reveal different biological scenarios. One difference is in material preparation. For instance, we used pedicel explants with flower attached while Meir *et al.* (2010) used pedicels with flowers removed. Similarly, Meir *et al.* (2010) studied a 14-h time course and we are interested in the early molecular events at 6 h with relatively high ethylene concentration. These differences should contribute to the discrepancies in gene expression patterns. Despite this, our approach was substantiated by the expected expression patterns of the abscission zone marker genes such as *TAPG2* and *TAPG4* that have been experimentally characterized [8], [47]. Particularly, most abscission zone differentially expressed genes in our study showed unique expression patterns when compared with its expressions in the basal and apical portions, demonstrating that responses of different tissues during abscission are underlined by different molecular mechanisms.

### Differential Gene Expressions among Tomato Pedicel Tissues

Despite its simplicity in cell type (small, undifferentiated), our transcriptome analyses showed that the tomato pedicel abscission zone conferred a dominant number of differentially expressed genes over its neighboring tissues in terms of both tissue specificity and abscission. Consistent with previous work, a variety of genes were found in the abscission zone. Major categories include those for the degradation and synthesis of cell wall polysaccharides (PGs. XTHs, CELs, EXPs), hormone metabolism (ethylene, jasmonic acid, auxin, abscisic acid, brassinosteroid, gibberellin), defense and interaction with the environment (ROS, PRs, LRRs), secondary metabolism (flavonoids, isoprenoids, phenylpropanoids), PS, signaling (kinases, calcium-binding proteins, G-proteins), and transcription (TFs). Generally, genes involved in stress and amino acid metabolism were largely induced while those for PS, secondary metabolism, cell wall, and transport were significantly suppressed, indicating a slow down of biochemical reactions inside the abscission zone and the activation of the stress response and protection systems during abscission.

The interplay of plant hormones has been widely reported. Similar to previously reported, the induction of ethylene biosynthesis and signaling genes were characterized among differentially expressed genes during abscission, although none of the three ethylene receptors exhibited abscission zone-specific patterns. As expected, auxin regulating genes were largely suppressed, mostly in non-tissue-specific manners, displaying an antagonistic interaction between these two major players in abscission. The expressions of auxin related genes, such as the auxin homeostasis gene *GH3.3*, often form steep gradient from distal to proximal along the pedicel, consistent with the proposed polar transportation of auxin from the flower to the base of the pedicel [7], [8], [48]. For other hormones, the induction of rate limiting enzyme genes for gibberellin, jasmonic acid, and brassinosteroid suggests a coordinated regulatory modes among these hormone related genes, while enhanced expression of the ABA catabolism gene *8′-hydroxylase CYP707A3* [49] may indicate increased abscisic acid breakdown during abscission. It has to be noted that the immediate responses of cell wall modification genes in the abscission zone, especially those encoding TAPGs and Cels should provide additional evidence for the effectiveness and specificity of our experimental approach.

### Transcription Regulation in the Tomato Flower Abscission Zone: From Development to Abscission

Abscission is generally considered to be achieved through four major steps: the development of the abscission zone cells, the acquisition of competence to respond to abscission signals, the activation of abscission, and the differentiation of a protective layer at the distal end of the basal portion [50]. This last step requires (1) the abscission zone cells to confer meristem-like nature and (2) the meristem status be removed by repressing the expression of meristem maintenance genes, such as *WUS* and *STM*. Our genome-wide tissue specific expression analysis of tomato pedicels supports such a notion. We found that key meristem maintenance genes were preferentially expressed in the abscission zone, including homologs of Arabidopsis *WUS*, *KNAT6*, *LBD1*, *LH1*and *OFP*, as well as the axillary meristem initiation genes *Bl* and *Ls*. Our findings are consistent with the recent discoveries in Arabidopsis where shoot apical meristem and axillary meristem genes work together for lateral organ development including leaf and flower, in which the formation of the abscission zone is an integrative step [51], [52]. Unexpectedly, homologs of *WUS* and its potential functional partner *OFP* appear to be specifically repressed in the abscission zone during abscission when their expression level in the basal portion and the apical portion remained largely unchanged. In fact, *STM,* as well as its closely interacting gene *AS1*, was also significantly repressed in the abscission zone, but not in an abscission zone specific manner. These data together corroborate the idea that reduction of meristem activity gene expression is necessary for additional cell differentiation in the abscission zone during or after abscission. Finally, the modulation of the *LeSPL3* homolog could add another layer of regulation of abscission zone functions by microRNAs [53]. Since similar genes are found for grass auricle development, the regulatory scheme proposed in our work may represent a common scheme for both monocot and eudicot abscission.

## Materials and Methods

### Plant Materials, Growth Conditions, and Ethylene Treatment

Tomato plants, *Solanum lycopersicon* accession LA3021, were grown under greenhouse conditions with a 16-h photoperiod and 25°C constant temperatures. Floral explants were inserted into a petri dish with 1/2 MS agar located inside in a Plexiglass box in a tray filled with a layer of water. Ethylene gas from an airtight vessel was conducted into the box with a soft tube through the water and reached the final concentration of 50 ul/L. Three duplicates were prepared so that tissues can be obtained at 0, 3, and 6 h once treatment started.

### Flower Dissection and RNA Extraction

Pedicels of pre-anthesis flowers were dissected using a sharp razor blade into three segments (abscission zone, distal, and proximal tissues; Fig. 1B), each about 1.5 mm, and were put into liquid nitrogen immediately. RNA was isolated using TRIZOL (Invitrogen) according to manufacturer’s instructions. Three independent biology repeats were performed.

### Microarray Analysis

Microarray hybridization and initial data subtraction were performed by Bo-ao company (Beijing) using the Affymatrix platform. We used Robust Multichip Analysis [54] in the Affymatrix package for initially probe signal summarization, normalization, and background subtraction with default parameters. Data comparison of different time points and tissues were performed using the SAM software [55]. The approach of [56] was applied to generate adjusted P values (q values) so as to control the level of false discoveries due to multiple comparisons. Three biology replicates were performed per treatment. Differentially expressed genes were screened using threshold fold change ≥2 and FDR (false discovery rate) ≤0.01. Microarray probe sequences were further annotated by comparison with Arabidopsis proteins using BlastX. Redundant ESTs that were annotated to be the same Arabidopsis genes were removed. Non-redundant dataset were imported into the MapMan program for functional classification. Gene ontology enrichment analysis was performed in the BinGO website [57]. The mean normalized signal intensity for each probe set was calculated from three biological replications. Signal intensities of each gene were standardized so that each gene (row) would have a mean of zero and an SD of 1. Six clusters were chosen by the gap statistic [58]. The *K-*mean clustering method was used to assign membership of each probe set [59].

### Semi-quantitative and Real Time RT-PCR

RNA samples were reverse transcribed using superscript II reverse transcriptase (Invitrogen). cDNA then diluted 10 times for semi-quantitative RT-PCR reaction. To ensure equal amounts of cDNA, a control reaction was performed with the tomato *ACTIN* gene. Quantitative real time RT-PCR reaction was performed in triplicate using QuantiTect SYBR Green PCR Master Mix (Qiagen) on an Iq5 Real Time PCR Detection System (Bio-Rad). Average threshold cycle (CT) values were calculated for each gene of interest, and were normalized and used to calculate relative transcript levels of the transcript using the 2^−ΔΔCT^ method described by [60]. The *GAPDH* (glyceraldehyde-3-phosphate dehydrogenase) from tomato was used as an internal standard for normalization.

## Supporting Information

Figure S1 Modulation of additional transcription factors in tomato pedicel tissues during ethylene-promoted abscission.
(TIF)

Figure S2 Induction of additional cell wall modification genes in tomato pedicel tissues during ethylene-promoted abscission.
(TIF)

Table S1 RT-PCR validation of differentially expressed genes. RNA concentrations were adjusted using the actin gene.
(PDF)

Table S2 Change in gene expression induced by ethylene compared to air for 68 genes.
(PDF)

Table S3 Differentially expressed genes after 3 h or 6 h ethylene treatment in the abscission zone.
(PDF)

Table S4 Differentially expressed genes in the basal portion after 3 h or 6 h ethylene treatment.
(PDF)

Table S5 Differentially expressed genes in the apical portion after 3 h or 6 h of ethylene treatment.
(PDF)

Table S6 MapMan classification of differentially expressed non-redundant genes in abscising abscission zone.
(PDF)

Table S7 The full list of the abscission zone-preferentially expressed genes.
(PDF)
