# Supplementary figures and images for: Transcriptome Analysis of Tomato Flower Pedicel Tissues Reveals Abscission Zone-Specific Modulation of Key Meristem Activity Genes
Source: PLoS One. 2013 Feb 4;8(2):e55238. doi: 10.1371/journal.pone.0055238 (PMC3563536; doi:10.1371/journal.pone.0055238)

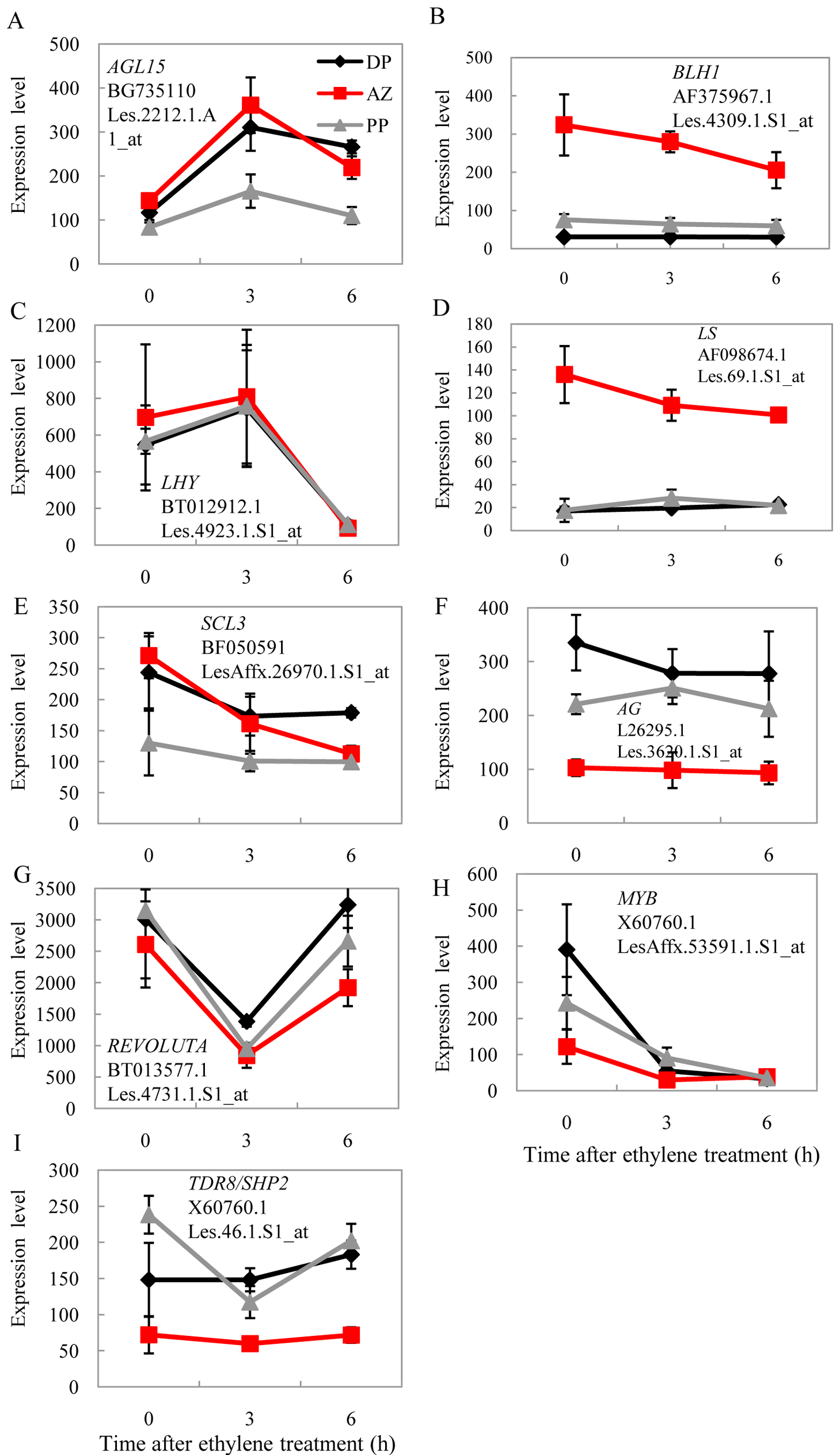

Supplement: Figure S1 — Modulation of additional transcription factors in tomato pedicel tissues during ethylene-promoted abscission. (TIF) [file pone.0055238.s001.tif]

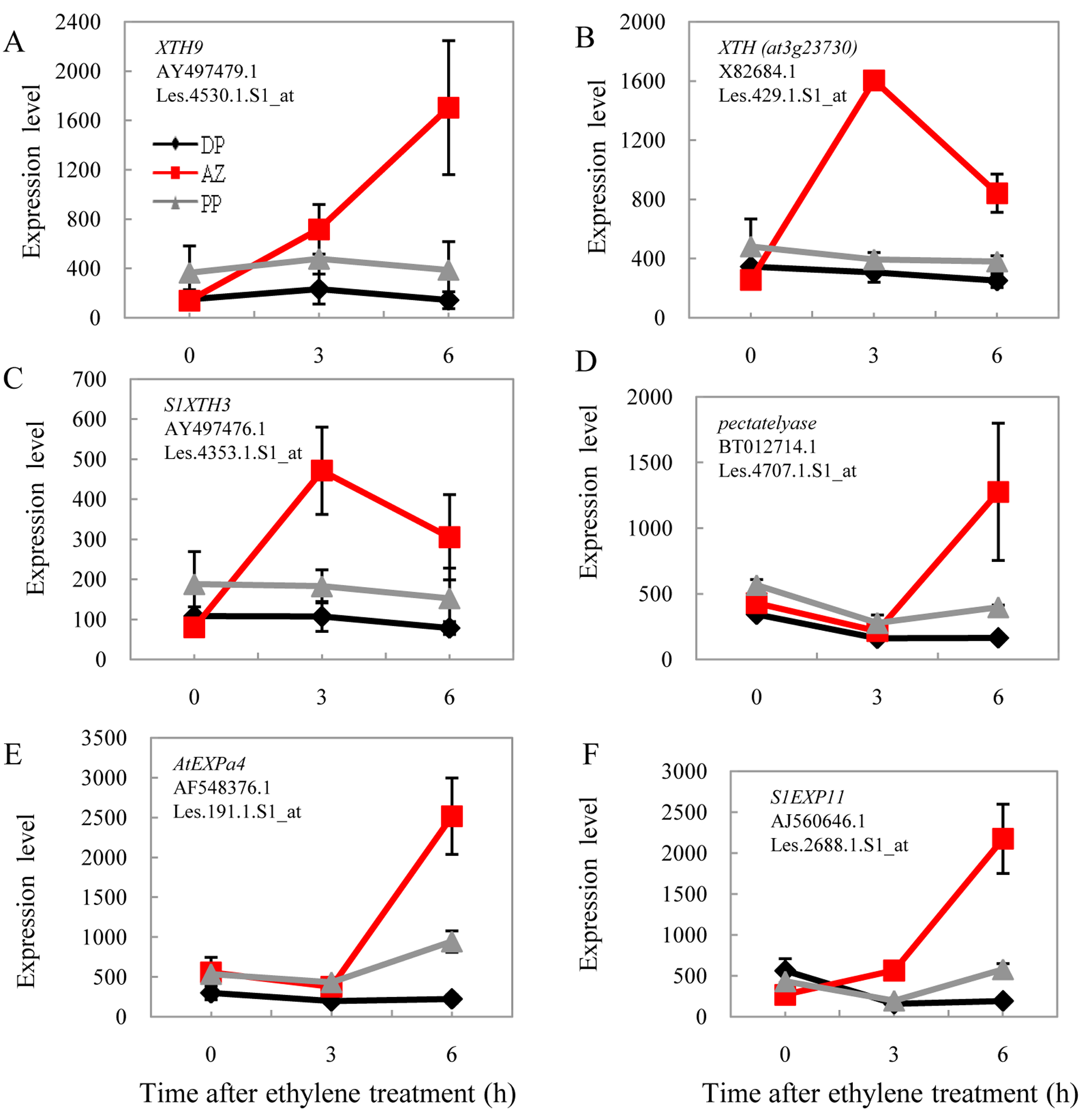

Supplement: Figure S2 — Induction of additional cell wall modification genes in tomato pedicel tissues during ethylene-promoted abscission. (TIF) [file pone.0055238.s002.tif]
