# Supplementary material for: Transcriptome Analysis of Tomato Flower Pedicel Tissues Reveals Abscission Zone-Specific Modulation of Key Meristem Activity Genes
Source: PLoS One. 2013 Feb 4;8(2):e55238. doi: 10.1371/journal.pone.0055238 (PMC3563536; doi:10.1371/journal.pone.0055238)
Supplement: Table S1 — RT-PCR validation of differentially expressed genes. RNA concentrations were adjusted using the actin gene. (PDF) [file pone.0055238.s003.pdf]

Table S1. *RT-PCR validation of differentially expressed genes. RNA concentrations were adjusted using the actin gene.*

Categories	Probe set ID	Genbank Accession	Gene	Ratio of microarray signal	RT PCR
**Tissue-specific**				0h AP:0h AZ:0h BP	
AZ					
	LesAffx.39.1.S1_at	CN385433	Peroxidase 72 precursor	0.009:1:0.087	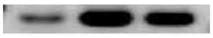
	Les.3668.1.S1_at	U09026.1	Lipoxygenase	0.044:1:0.477	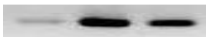
	LesAffx.61901.1.S1_at	AW931428	Patatin-like phospholipase	0.093:1:0.114	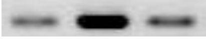
	Les.4938.1.S1_at	BT012940.1	Unknown	0.059:1:0.278	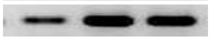
	Les.4136.1.S1_at	AJ538329.1	Wuschel	0.06:1:0.065	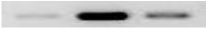
	LesAffx.24134.1.S1_at	CK720539	Oxidoreductase	0.1:1:0.188	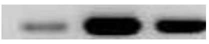
	Les.3693.1.S1_at	AF426174.1	Blind	0.041:1:0.103	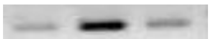
	Les.1175.2.S1_at	AI777697	Photoassimilate protein	0.073:1:0.226	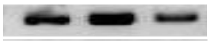
	Les.69.1.S1_at	AF098674.1	Lateral suppressor	0.125:1:0.132	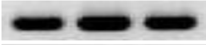
	LesAffx.62669.1.S1_at	AW442297	LOB domain protein	0.276:1:0.476	
non-AZ					
	LesAffx.26180.1.S1_at	BG132890	Nudix hydrolase	4.69 : 1 : 2.74	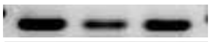
	Les.2001.1.S1_at	AW029653	Unknown protein	2.847 : 1 : 3.058	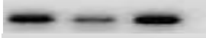
	Les.144.1.S1_at	Z75519.1	Glycine-rich protein	2.871 : 1 : 2.909	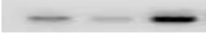
	Les.3415.3.S1_at	AI779314	Chloroplast protein 12	2.05 : 1 : 2.65	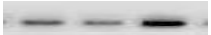
	Les.4896.1.S1_at	BT012857.1	Pollen allergen Phl p 11	3.564 : 1 : 3.941	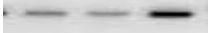
**Ethylene response**					
**AP**				AP (0h:3h:6h)	
	LesAffx.56389.1.S1_at	AW034707	NADH- oxidoreductase	0.122 : 1 : 3.98	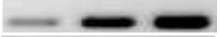
	LesAffx.59842.1.S1_at	BI205718	Cytochrome P450 724B	0.813 : 1 : 15.6	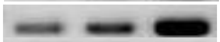
	LesAffx.65984.1.S1_at	BG125327	Glycosyl hydrolase	3.75 : 1 : 0.261	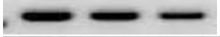
	Les.3733.1.S1_at	AF096776.1	Expansin	4.91 : 1 : 0.319	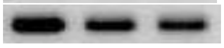
AZ				AZ (0h:3h:6h)	
	LesAffx.67592.1.S1_at	AW093105	Protease inhibitor	0.09 : 1 : 1.68	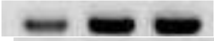
	Les.5442.1.S1_at	BT013931.1	AUX/IAA family protein	0.232 : 1 : 2.674	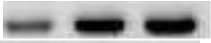
	Les.2316.1.S1_at	AW623527	Cellulose synthase	3.545 : 1 : 0.166	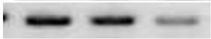
	Les.2316.2.A1_at	BG626036	Glycine-rich protein	3.093 : 1 : 0.164	
BP				BP (0h:3h:6h)	
	LesAffx.8850.1.S1_at	AW034398	Pepper esterase	0.358 : 1 : 3.085	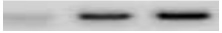
	LesAffx.29797.1.S1_at	AJ784483	Sulfotransferase	1.271 : 1 : 0.148	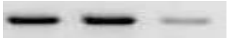
