# Supplementary material for: Transcriptome Analysis of Tomato Flower Pedicel Tissues Reveals Abscission Zone-Specific Modulation of Key Meristem Activity Genes
Source: PLoS One. 2013 Feb 4;8(2):e55238. doi: 10.1371/journal.pone.0055238 (PMC3563536; doi:10.1371/journal.pone.0055238)
Supplement: Table S2 — Change in gene expression induced by ethylene compared to air for 68 genes. (PDF) [file pone.0055238.s004.pdf]

Table S2 Change in gene expression induced by ethylene compared to air for 68 genes. Changes are expressed as the ratio of expression at 6 h to that at zero h

Gene Index	GB acc.	Gene symbol	Ethylene change[1] Microarray	Air change[2] qPCR
**I. Genes with constant expression in air (less than 2-fold change)**				
TC21734	U23053.1	**TAPG1**[3]	↑ 159	1.43
TC21734	U70480.1	**TAPG2**	41.6	1.06
TC23081	AF077339.1	**Cel5**	17.6	0.48
TC24190	BT013446.1	**GH3.3**	14.5	1.22
TC21992	AY497479.1	XTH9	12.2	1.28
TC22864	AF049900.1	**GA20**	11	0.89
TC22599	AI490099	BFN1	6.72	1.11
BF11399	BF113993	BT4	5.2	0.61
TC21718	AY079426.1	**LeETR6**	4.89	0.74
TC21771	AF548376.1	Expansin	4.52	1.11
TC21831	BT014429.1	RD19	4.32	0.97
TC21715	AF154421.1	BGAL1	4.29	1.00
TC22139	BI923132	CML	4.05	1.33
TC23203	AW442721	MEP1	4.04	1.36
TC23714	CD002272	PNAE	3.68	0.73
TC23074	BE461086	PB1	3.68	0.64
TC22193	AY098737.2	**TAGL12**	3.45	0.68
TC22898	AW031388	PPA	3.43	0.64
TC22813	AF426174.1	**BLIND**	3.03	1.20
TC23564	AI898478	NAM	2.4	0.69
TC21752	AF502085.1	**ERF1**	2.33	0.63
TC23336	BI209612	**GATA**	2.1	1.44
TC22524	AY306154.1	**MC**	↓ 0.48	0.69
TC24276	BT013305.1	**TCP**	0.45	0.90
NP00014	AF000141.1	**STM**	0.39	0.80
TC21818	BT013322.1	PORB	0.34	1.25
TC22899	BT014416.1	SPDS3	0.31	0.75
TC23130	BT013032.1	APS1	0.27	1.43
TC21903	AY497478.1	XTH7	0.26	0.55
TC22543	BI929558	**SPL3**	0.24	0.96
TC22946	BT013056.1	plastocyanin	0.23	0.97
TC23669	BI931445	M20	0.23	0.70
TC23566	BG631822	gatA	0.22	0.94
TC23249	BG631141	POT	0.21	0.82
TC22972	BG627575	EXL3	0.21	1.57
TC22795	BG626083	LTP	0.18	0.97
TC23911	AI487223	SALAT	0.13	1.26
GO37548	CK574995	ARA6	0.12	1.48

II. Genes with changed expression in air (more than 2-fold change)

TC21823	AF317515.1	**AOS**	↑ 19.68	2.94
TC23987	AI489739	**ABA8H**	11.76	6.09
TC21735	U70481.1	**TAPG4**	9.76	38.66
TC22594	AY007561.1	ST4	9.11	3.71
TC22082	BI921137	FBL4	7.29	4.93
TC21823	BI205718	**DWF4**	6.75	14.44
TC24138	BT012835.1	TR	6.47	3.06
GO37635	BT014302.1	CYS	6.33	2.43
TC22806	AJ277944.1	**SNAT**	5.18	2.22
TC22030	X79338.1	RNS1	4.59	2.59
BG13289	BG132890	MutT	4.37	3.69
TC22810	BG628131	PMSR	3.71	4.29
TC24026	BI208638	BOU	3.45	2.15
TC22384	AI773710	SDR	3.37	5.24
TC21740	AJ715790.1	**ACO1**	2.42	38.27
TC24128	BT013703.1	CBF	↓ 0.27	0.46
TC24140	BI931777	SPP	0.25	0.48
TC23425	BT013249.1	LPSP	0.22	4.81
TC23040	BI933687	CYCP3	0.21	0.32
TC22401	BT014414.1	XHB	0.21	12.11
TC23312	BT014212.1	GAPN	0.21	0.18
TC22961	BT014378.1	RPT2	0.19	0.20
TC23773	AY497477.1	XTH6	0.17	3.23
TC23069	AI491145	MGLL	0.15	0.24
TC21735	AF191823.1	**AGAL2**	0.15	0.38
TC21723	U50151.1	lap	0.15	16.55
NP59756	AJ538329.1	**LeWUS**	0.14	0.26

III. Genes with constant expression in air and in ethylene

AI77662	AI776626	**ERF2**	1.31	0.60
TC21842	AY140893.1	**OFP**	0.96	0.93
TC22018	AF148934.1	**AS1**	0.69	0.77

Genes within each section of the table are grouped by the magnitude and sign of their response to ethylene. [1]Data are from the microarray results described in the main text; [2]Gene expression for air treated samples was measured by qPCR. Relative transcript level of each gene was obtained as 2^-DCT, where DCT is the difference in the number of cycles to the threshold between the gene and the reference (tomato actin gene, GB acc.U60482.1). The data show the ratio of expression at 6 h to that at 0 h. Results are the average of three biological replicates which each had three technical replicates.; [3]Bold face indicates genes mentioned in the text.
