# Supplementary material for: Transcriptome Analysis of Tomato Flower Pedicel Tissues Reveals Abscission Zone-Specific Modulation of Key Meristem Activity Genes
Source: PLoS One. 2013 Feb 4;8(2):e55238. doi: 10.1371/journal.pone.0055238 (PMC3563536; doi:10.1371/journal.pone.0055238)
Supplement: Table S3 — Differentially expressed genes after 3 h or 6 h ethylene treatment in the abscission zone. (PDF) [file pone.0055238.s005.pdf]

Table S3. Differentially expressed genes after 3 h and/or 6 h ethylene treatment in the abscission zone

Affymetrix Probe Set ID	GenBank Accession	Tomato Gene Index	0AZ	3AZ	6AZ	3AZ/0AZ	6AZ/0AZ	GenBank Annotation
Les.3665.1.S1_at	U23053.1	TC21734	12	528	1970	42.62	159.18	polygalacturonase precursor
Les.3647.1.S1_at	U70480.1	TC21734	98	2959	4085	30.15	41.62	abscission polygalacturonase
Les.5915.1.S1_at	AY344539.1	TC22182	22	331	467	15.25	21.48	alpha-DOX1
Les.129.1.S1_at	AF317515.1	TC21823	80	1049	1571	13.14	19.68	divinyl ether synthase
Les.4885.1.S1_at	BT012835.1	TC24138	554	6412	3585	11.57	6.47	Lycopersicon esculentum clone 113896R, mRNA sequence
LesAffx.67592.1.S1_at	AW093105	TC22664	221	2467	4144	11.19	18.79	Transcribed locus, weakly similar to NP_061135.1 (Asp-Glu-Ala-Asp) box polypeptide 43 [Homo sapiens]
LesAffx.68306.1.S1_at	BE461086	TC23074	128	1265	470	9.92	3.68	Transcribed locus, weakly similar to NP_001055605.1 [Oryza sativa (japonica cultivar-group)]
LesAffx.22699.1.A1_at	AI489739	TC23987	19	186	224	9.76	11.76	Transcribed locus, moderately similar to NP_001047855.1 [Oryza sativa (japonica cultivar-group)]
Les.4527.1.S1_at	AJ635323.1	TC22837	122	1043	457	8.57	3.75	polyphenol oxidase A
Les.3646.1.S1_at	U70481.1	TC21735	967	8077	9439	8.36	9.76	abscission polygalacturonase
Les.3712.1.S1_at	AF154003.1	TC24028	417	3446	2653	8.26	6.36	pirin
LesAffx.56389.1.S1_at	AW034707	TC23934	273	2189	2448	8.02	8.97	Transcribed locus, weakly similar to NP_001033715.2 F synthase [Sus scrofa]
Les.132.1.S1_at	X58885.1	TC21743	1254	9707	9772	7.74	7.79	ethylene-forming enzyme
LesAffx.52594.1.S1_at	AI895802	TC22372	43	314	555	7.27	12.88	Transcribed locus, weakly similar to NP_001047179.1 [Oryza sativa (japonica cultivar-group)]
LesAffx.63489.1.S1_at	BI921137	TC22082	277	1864	2020	6.73	7.29	Transcribed locus, weakly similar to XP_001117063.1 transmembrane BAX inhibitor motif containing 4 isoform 1 [Macaca mulatta]
LesAffx.66354.1.S1_at	AI773917	TC22427	117	769	300	6.57	2.56	Transcribed locus, weakly similar to NP_001057807.1 [Oryza sativa (japonica cultivar-group)]
Les.429.1.S1_at	X82684.1	TC22396	256	1603	841	6.27	3.29	xyloglycan endo-transglycosylase
Les.4353.1.S1_at	AY497476.1	TC21758	80	471	305	5.87	3.8	xyloglucan endotransglucosylase-hydrolase XTH3
LesAffx.8850.1.S1_at	AW034398	TC22181	96	538	2565	5.62	26.75	Subtilisin-like protease
Les.4287.1.S1_at	CB751564	TC21784	748	4170	7420	5.57	9.92	Transcribed locus, weakly similar to XP_001094505.1 proteasome 26S non-ATPase subunit 3 isoform 4 [Macaca mulatta]
LesAffx.13365.1.S1_at	BM411110	TC21920	314	1666	376	5.31	1.2	Transcribed locus, weakly similar to NP_001066487.1 [Oryza sativa (japonica cultivar-group)]
LesAffx.66301.2.S1_at	BI423134	TC22633	2	9	5	5.29	3.32	Transcribed locus, weakly similar to NP_001042589.1 [Oryza sativa (japonica cultivar-group)]
Les.4530.1.S1_at	AY497479.1	TC21992	139	717	1703	5.15	12.23	xyloglucan endotransglucosylase-hydrolase XTH9

Les.2899.1.S1_at	BG628131	TC22810	2352	12106	8732	5.15	3.71	Transcribed locus, weakly similar to XP_001108862.1 cathepsin H isoform 2 [Macaca mulatta]
Les.3766.1.S1_at	U77719.1	TC21779	235	1182	1092	5.02	4.64	ethylene-responsive late embryogenesis-like protein
LesAffx.3554.1.A1_at	CK720570	CK72057	16	78	346	5	22.28	---
Les.113.1.S1_at	CN384955	TC23130	1970	9667	9343	4.91	4.74	CDNA, clone: FC02BG03, HTC in fruit
LesAffx.56301.1.S1_at	AI895341	TC22182	1136	5437	5614	4.79	4.94	Transcribed locus, weakly similar to NP_001048145.1 [Oryza sativa (japonica cultivar-group)]
Les.934.1.S1_at	BG628248	GO37331	1670	7875	6408	4.72	3.84	Cell wall protein
Les.4307.1.S1_at	AY257487.1	TC21819	2194	10162	12323	4.63	5.62	PR5-like protein
Les.5672.1.S1_at	BT014363.1	TC23335	62	285	473	4.56	7.59	Lycopersicon esculentum clone 133649F, mRNA sequence
Les.3491.1.S1_at	AF077339.1	TC23081	210	955	3704	4.55	17.64	endo-1,4-beta-glucanase
Les.1524.1.A1_at	BG626458	BG62645	442	1943	1128	4.39	2.55	Transcribed locus
LesAffx.37595.1.S1_at	CN385844	TC22360	149	647	467	4.35	3.14	---
Les.5530.1.S1_at	BT014086.1	TC23075	906	3931	3197	4.34	3.53	Lycopersicon esculentum clone 133190R, mRNA sequence
Les.5442.1.S1_at	BT013931.1	TC22814	176	760	2032	4.31	11.54	Lycopersicon esculentum clone 132929R, mRNA sequence
LesAffx.69998.1.S1_at	BI203963	TC23259	70	296	225	4.23	3.21	Transcribed locus, moderately similar to NP_001048806.1 [Oryza sativa (japonica cultivar-group)]
Les.3575.1.S1_at	U89256.1	TC22509	52	220	245	4.2	4.7	Pti5
LesAffx.31873.1.S1_at	BI928574	BI92857	487	2036	4330	4.18	8.88	---
Les.26.1.S1_at	AF088276.1	TC21861	78	323	752	4.15	9.63	NADPH oxidase
LesAffx.64582.1.S1_at	AI894448	TC24338	392	1617	758	4.13	1.93	---
Les.4038.1.S1_at	AY081908.1	TC21781	177	725	651	4.09	3.68	Lycopersicon esculentum N-hydroxycinnamoyl-CoA:tyramine N-hydroxycinnamoyl transferase THT1-4 (THT1-4) pseudogene
LesAffx.67116.1.S1_at	BM413085	TC21926	209	849	810	4.07	3.88	Transcribed locus, weakly similar to NP_065266.1 translocase [Mus musculus]
Les.2899.2.S1_at	BM536145	TC22810	1101	4418	2846	4.01	2.58	Transcribed locus, weakly similar to XP_001108862.1 cathepsin H isoform 2 [Macaca mulatta]
Les.4888.1.S1_at	BI927238	TC24096	754	3009	3539	3.99	4.7	Lycopersicon esculentum clone 113905R, mRNA sequence
LesAffx.37595.1.A1_at	CN385844	TC22360	32	126	81	3.97	2.56	---
LesAffx.71377.1.S1_at	BG123375	TC22940	78	309	172	3.96	2.2	Transcribed locus, weakly similar to NP_001047645.1 [Oryza sativa (japonica cultivar-group)]
LesAffx.59668.1.S1_at	BI208638	TC24026	516	2015	1779	3.91	3.45	Transcribed locus, weakly similar to NP_065266.1 translocase [Mus musculus]
Les.1046.1.S1_at	X72730.1	TC22785	268	1041	651	3.89	2.43	(ERT 10) ripening-related mRNA
LesAffx.62570.2.S1_at	CD003040	TC24016	476	1849	1810	3.88	3.8	Transcribed locus, weakly similar to NP_001044685.1 [Oryza sativa (japonica cultivar-group)]
LesAffx.22583.1.S1_at	AI484022	TC23670	358	1383	999	3.86	2.79	Transcribed locus, weakly similar to NP_001044974.1 [Oryza sativa (japonica cultivar-group)]

Les.4055.1.S1_at	BM536050	TC23122	91	350	244	3.86	2.68	Lycopersicon esculentum (DB222) meloidogyne-induced giant cell protein mRNA, 3' end
LesAffx.50687.1.S1_at	AI773710	TC22384	485	1869	1635	3.85	3.37	Transcribed locus, weakly similar to NP_001046905.1 [Oryza sativa (japonica cultivar-group)]
LesAffx.69872.1.S1_at	CN385882	TC23745	101	387	631	3.84	6.25	Transcribed locus
Les.4222.1.S1_at	AY034149.1	TC21756	167	637	477	3.82	2.86	alternative oxidase 1b
Les.5051.1.S1_at	BT013182.1	TC22934	275	1047	1207	3.81	4.39	Lycopersicon esculentum clone 134258F, mRNA sequence
Les.3818.1.S1_at	AF502085.1	TC21752	192	729	446	3.8	2.33	ethylene responsive element binding protein
Les.2708.1.S1_at	BG734625	TC21907	2182	8284	8140	3.8	3.73	Transcribed locus, moderately similar to NP_001044464.1 [Oryza sativa (japonica cultivar-group)]
LesAffx.70522.1.S1_at	BI423015	TC23029	567	2147	2806	3.78	4.94	Transcribed locus, weakly similar to NP_001055496.1 [Oryza sativa (japonica cultivar-group)]
LesAffx.64582.2.A1_at	AI490624	TC24338	212	797	318	3.75	1.5	---
LesAffx.62570.2.A1_at	CD003040	TC24016	75	280	192	3.74	2.57	Transcribed locus, weakly similar to NP_001044685.1 [Oryza sativa (japonica cultivar-group)]
LesAffx.60582.1.S1_at	AI899413	TC23540	264	985	804	3.73	3.04	Transcribed locus, weakly similar to NP_001048083.1 [Oryza sativa (japonica cultivar-group)]
Les.2256.1.S1_at	BT014302.1	GO37635	120	441	756	3.69	6.33	Lycopersicon esculentum clone 133553F, mRNA sequence
Les.3465.1.S1_at	AY079426.1	TC21718	221	811	1082	3.67	4.89	ethylene receptor-like protein
LesAffx.69349.2.S1_at	AW623862	AW62386	251	916	970	3.65	3.87	Transcribed locus, weakly similar to NP_001043801.1 [Oryza sativa (japonica cultivar-group)]
Les.2717.1.S1_at	AW218373	TC22994	576	2094	1948	3.63	3.38	Transcribed locus, moderately similar to NP_001057280.1 [Oryza sativa (japonica cultivar-group)]
Les.4819.1.S1_at	BT013586.1	TC24267	684	2482	827	3.63	1.21	Lycopersicon esculentum hsc-1 mRNA for heat shock protein 70 kD
Les.559.1.A1_at	BG626246	TC22601	229	828	1081	3.62	4.73	Transcribed locus, weakly similar to NP_001043106.1 [Oryza sativa (japonica cultivar-group)]
Les.4888.1.A1_at	BT012839.1	AW39852	329	1182	1146	3.6	3.49	Lycopersicon esculentum clone 113905R, mRNA sequence
LesAffx.54536.1.S1_at	BM409044	TC23408	351	1256	1050	3.58	3	Transcribed locus, weakly similar to NP_001046834.1 [Oryza sativa (japonica cultivar-group)]
Les.5884.1.S1_at	CK716273	TC21763	1125	3978	7818	3.54	6.95	Transcribed locus, weakly similar to NP_173228.1 inhibitor [Arabidopsis thaliana]
Les.1665.1.S1_at	AW623646	DB71244	511	1795	1067	3.52	2.09	Transcribed locus, weakly similar to NP_001042158.1 [Oryza sativa (japonica cultivar-group)]
LesAffx.33607.2.S1_s_at	AI775872	TC23890	603	2100	1839	3.48	3.05	Calmodulin
LesAffx.43329.1.S1_at	AI898478	TC23564	136	473	328	3.47	2.4	Transcribed locus, weakly similar to NP_001041763.1 [Oryza sativa (japonica cultivar-group)]

LesAffx.70406.1.S1_at	AW036249	TC22360	206	709	1173	3.45	5.71	Transcribed locus, weakly similar to XP_001114236.1 branched chain aminotransferase 2, mitochondrial isoform 2 [Macaca mulatta]
Les.3716.1.S1_at	AJ277944.1	TC22806	718	2473	3718	3.44	5.18	SANT/MYB domain protein
LesAffx.68459.2.S1_at	BM411992	TC22747	191	656	682	3.44	3.57	Transcribed locus, weakly similar to NP_001051596.1 [Oryza sativa (japonica cultivar-group)]
LesAffx.735.1.S1_at	AW217158	TC22685	293	1002	736	3.42	2.51	Transcribed locus, weakly similar to NP_001042221.1 [Oryza sativa (japonica cultivar-group)]
Les.423.1.S1_at	BM409421	TC22953	1000	3413	2260	3.41	2.26	CDNA, clone: FC09CF07, HTC in fruit
Les.2417.2.A1_at	BG735382	TC23220	163	557	382	3.41	2.34	Transcribed locus
LesAffx.71065.1.S1_at	BM413117	TC22472	853	2901	3161	3.4	3.7	Transcribed locus, weakly similar to NP_001042582.1 [Oryza sativa (japonica cultivar-group)]
LesAffx.57303.1.S1_at	AW625293	TC22186	458	1551	1936	3.39	4.23	Transcribed locus, weakly similar to NP_198885.1 protein [Arabidopsis thaliana]
LesAffx.9910.1.S1_at	CN385590	TC22147	823	2779	2593	3.38	3.15	Transcribed locus, weakly similar to NP_001046094.1 [Oryza sativa (japonica cultivar-group)]
Les.4022.1.S1_at	X94946.1	TC21814	349	1172	2103	3.36	6.02	proteinase inhibitor II
Les.2084.1.S1_at	BF097539	TC22764	298	994	845	3.34	2.84	Transcribed locus, weakly similar to NP_001041763.1 [Oryza sativa (japonica cultivar-group)]
LesAffx.58497.1.S1_at	BM411367	TC23480	49	164	121	3.32	2.45	---
LesAffx.43198.1.S1_at	BF113993	BF11399	118	391	615	3.3	5.2	Transcribed locus, weakly similar to NP_001045064.1 [Oryza sativa (japonica cultivar-group)]
Les.702.1.S1_at	BG627003	TC21772	1175	3875	1348	3.3	1.15	Lycopersicon esculentum clone 134862F, mRNA sequence
Les.868.1.A1_at	BG627808	TC22948	34	112	39	3.26	1.15	Transcribed locus, weakly similar to NP_001042402.1 [Oryza sativa (japonica cultivar-group)]
LesAffx.67643.1.S1_at	BI926164	TC22219	364	1188	380	3.26	1.04	Transcribed locus, weakly similar to XP_001116534.1 similar to asparaginase-like 1 protein isoform 1 [Macaca mulatta]
Les.1826.1.A1_at	BG629397	TC23561	288	935	1023	3.25	3.56	Transcribed locus
LesAffx.22830.1.S1_at	CN385777	TC22584	168	544	409	3.24	2.44	Transcribed locus, weakly similar to NP_001063070.1 [Oryza sativa (japonica cultivar-group)]
LesAffx.62138.1.S1_at	AW737374	TC24364	289	936	970	3.23	3.35	---
Les.244.2.S1_a_at	AI485696	TC22935	173	556	510	3.21	2.95	Calmodulin
LesAffx.57363.1.S1_at	AW035806	TC21958	132	423	561	3.21	4.26	Transcribed locus, weakly similar to NP_001042342.1 [Oryza sativa (japonica cultivar-group)]
LesAffx.16102.1.S1_at	BI208311	TC23896	82	261	202	3.19	2.48	Transcribed locus, weakly similar to NP_001042808.1 [Oryza sativa (japonica cultivar-group)]
LesAffx.69783.1.S1_at	AW220917	TC23448	601	1882	2066	3.13	3.44	Transcribed locus, weakly similar to NP_001067067.1 [Oryza sativa (japonica cultivar-group)]

LesAffx.63935.1.S1_at	AW442721	TC23203	224	701	904	3.13	4.04	Transcribed locus, weakly similar to XP_001098000.1 matrix metalloproteinase 8 [Macaca mulatta]
LesAffx.33607.1.S1_s_at	AW223067	TC23890	250	777	698	3.11	2.8	Calmodulin
Les.5934.1.S1_at	AI895164	TC21861	354	1101	2831	3.11	7.99	Transcribed locus, weakly similar to NP_001047927.1 [Oryza sativa (japonica cultivar-group)]
LesAffx.15921.1.S1_at	BM412286	TC23758	727	2257	1795	3.11	2.47	Transcribed locus, weakly similar to NP_001057315.1 [Oryza sativa (japonica cultivar-group)]
Les.3683.1.S1_at	AY093595.1	TC24086	352	1092	1785	3.1	5.07	PR-5x
LesAffx.11542.1.S1_at	BI922302	TC21973	74	230	245	3.1	3.29	Transcribed locus, weakly similar to NP_001044352.1 [Oryza sativa (japonica cultivar-group)]
Les.3710.1.S1_at	AJ312131.1	TC21745	135	417	386	3.09	2.86	alternaria stem canker resistance protein
Les.2708.2.S1_at	AW218185	TC21907	1742	5274	5454	3.03	3.13	Transcribed locus, moderately similar to NP_001044464.1 [Oryza sativa (japonica cultivar-group)]
LesAffx.36193.1.S1_at	AW034622	TC22914	134	398	435	2.98	3.25	Transcribed locus, weakly similar to NP_001050443.1 [Oryza sativa (japonica cultivar-group)]
LesAffx.17017.1.S1_at	CN384671	TC24170	163	486	455	2.97	2.79	Transcribed locus, weakly similar to NP_186978.1 protein [Arabidopsis thaliana]
LesAffx.8720.1.S1_at	BI921446	BI92223	282	833	1438	2.96	5.11	Transcribed locus, weakly similar to NP_663449.2 P450-like [Mus musculus]
Les.5759.1.S1_at	BM956714	TC22993	378	1115	2305	2.95	6.09	Lycopersicon esculentum clone 133897F, mRNA sequence
LesAffx.71608.1.A1_at	CN385447	TC22596	32	93	74	2.94	2.34	Transcribed locus, weakly similar to NP_186978.1 protein [Arabidopsis thaliana]
LesAffx.38395.2.A1_at	AI488594	AI48859	110	321	272	2.92	2.48	---
LesAffx.3059.1.S1_at	AI776626	AI77662	115	336	151	2.91	1.31	---
Les.3735.1.S1_at	AY007561.1	TC22594	129	376	1176	2.91	9.11	hypothetical LOC543816
Les.5557.1.S1_at	BT014139.1		101	293	141	2.9	1.4	Lycopersicon esculentum clone 133258F, mRNA sequence
LesAffx.43329.1.A1_at	AI489164	TC23564	75	217	140	2.9	1.87	Transcribed locus, weakly similar to NP_001041763.1 [Oryza sativa (japonica cultivar-group)]
LesAffx.63659.1.S1_at	BI921813	TC22794	962	2787	2814	2.9	2.93	Transcribed locus, weakly similar to NP_001047062.1 [Oryza sativa (japonica cultivar-group)]
LesAffx.64990.1.S1_at	BI422984	TC21791	705	2042	1788	2.89	2.53	Transcribed locus, weakly similar to NP_001051696.1 [Oryza sativa (japonica cultivar-group)]
Les.4223.1.S1_at	AY034148.1	TC21729	645	1854	1134	2.87	1.76	alternative oxidase 1a
Les.2717.2.A1_at	BG631585	TC22994	884	2534	2449	2.87	2.77	Transcribed locus, moderately similar to NP_001057280.1 [Oryza sativa (japonica cultivar-group)]
LesAffx.71608.1.S1_at	AI486489	TC22596	61	175	138	2.86	2.25	Transcribed locus, weakly similar to NP_186978.1 protein [Arabidopsis thaliana]
LesAffx.3554.1.S1_at	CK720570	CK72057	19	54	158	2.85	8.31	---
LesAffx.71606.1.S1_s_at	AI896002	TC24027	1830	5197	4644	2.84	2.54	Lycopersicon esculentum clone 114334F, mRNA sequence
Les.5126.1.S1_at	BT013336.1	TC22310	428	1203	1253	2.81	2.93	Lycopersicon esculentum clone 135126R, mRNA sequence

LesAffx.61149.1.S1_at	BE460847	TC24084	459	1284	1756	2.8	3.83	Transcribed locus, weakly similar to NP_001042177.1 [Oryza sativa (japonica cultivar-group)]
LesAffx.64741.1.S1_at	AI775317	TC22074	1285	3544	2935	2.76	2.28	Transcribed locus, weakly similar to XP_001103467.1 similar to 3-hydroxyisobutyryl-Coenzyme A hydrolase isoform 1 [Macaca mulatta]
LesAffx.68107.1.S1_at	AI485479	TC23454	135	371	371	2.75	2.75	Transcribed locus, moderately similar to NP_001048067.1 [Oryza sativa (japonica cultivar-group)]
Les.2813.2.A1_at	BG734870	TC24032	648	1781	1662	2.75	2.56	Transcribed locus, moderately similar to NP_001045523.1 [Oryza sativa (japonica cultivar-group)]
LesAffx.38395.1.S1_at	BI933523	DB72634	509	1376	1471	2.7	2.89	Transcribed locus, moderately similar to NP_001057975.1 [Oryza sativa (japonica cultivar-group)]
LesAffx.57163.2.S1_at	BI421649	TC22802	341	918	145	2.7	0.43	Transcribed locus, weakly similar to NP_001044707.1 [Oryza sativa (japonica cultivar-group)]
LesAffx.30145.1.S1_at	AI775220	ES89125	2676	7210	7615	2.69	2.85	Transcribed locus, weakly similar to NP_001046577.1 [Oryza sativa (japonica cultivar-group)]
LesAffx.124.1.S1_at	AW443936	TC23232	37	99	108	2.69	2.93	CDNA, clone: FC20CD10, HTC in fruit
LesAffx.24637.1.S1_at	CN550618	TC22796	748	2010	4189	2.69	5.6	Transcribed locus, weakly similar to NP_001049089.1 [Oryza sativa (japonica cultivar-group)]
Les.5091.1.S1_at	BT013255.1	TC22888	306	823	457	2.69	1.49	Lycopersicon esculentum clone 134773R, mRNA sequence
LesAffx.8720.2.S1_at	AI895030	TC22019	482	1295	2210	2.68	4.58	Transcribed locus, weakly similar to NP_663449.2 P450-like [Mus musculus]
Les.1798.1.S1_at	AW221951	TC22540	136	365	227	2.68	1.67	Transcribed locus, weakly similar to NP_001046050.1 [Oryza sativa (japonica cultivar-group)]
Les.3619.1.S1_at	J04099.1	TC23203	2782	7424	9845	2.67	3.54	proteinase inhibitor I
Les.4999.1.A1_at	BT013077.1	TC23341	504	1344	1016	2.67	2.02	Lycopersicon esculentum clone 114334F, mRNA sequence
Les.1899.1.A1_at	BG630030	TC24035	16	42	19	2.66	1.22	Transcribed locus
LesAffx.37563.1.S1_at	Z70216.1	TC23549	234	620	409	2.65	1.75	---
LesAffx.51158.1.S1_at	AI897068	TC23014	180	476	355	2.64	1.97	Transcribed locus, weakly similar to NP_001048008.1 [Oryza sativa (japonica cultivar-group)]
LesAffx.47070.1.S1_at	AW651406	AW65140	23	62	93	2.64	3.98	Transcribed locus, moderately similar to NP_001042375.1 [Oryza sativa (japonica cultivar-group)]
Les.4999.1.S1_at	BT013077.1	TC23341	2510	6613	5895	2.63	2.35	Lycopersicon esculentum clone 114334F, mRNA sequence
LesAffx.71311.1.S1_at	CN384672	TC22685	129	340	190	2.63	1.47	CDNA, clone: FC16CB09, HTC in fruit
Les.5848.2.S1_at	BM535694	TC22461	210	550	316	2.62	1.5	Transcribed locus, weakly similar to XP_001121814.1 similar to CG9009-PA [Apis mellifera]
LesAffx.26054.1.S1_at	BG135770	TC21811	133	346	229	2.61	1.72	CDNA, clone: FC09AA01, HTC in fruit
Les.3774.1.S1_at	AJ010942.1	TC23863	1488	3864	2205	2.6	1.48	hexose transporter protein

LesAffx.7602.1.S1_at	AW443858	TC23283	940	2437	2131	2.59	2.27	Transcribed locus, weakly similar to XP_001106031.1 adenylate kinase 2 isoform 2 [Macaca mulatta]
Les.3935.1.S1_at	BT013403.1	TC21790	3663	9481	8863	2.59	2.42	Lycopersicon esculentum clone 135717R, mRNA sequence
LesAffx.52595.1.S1_at	AI778688	TC22841	878	2272	2340	2.59	2.66	Transcribed locus, weakly similar to XP_001103792.1 ERO1-like isoform 1 [Macaca mulatta]
Les.5014.1.S1_at	BT013105.1	TC23486	1248	3229	4039	2.59	3.24	Lycopersicon esculentum clone 114391R, mRNA sequence
LesAffx.47179.1.S1_at	BM411655	TC24073	510	1313	859	2.58	1.69	Transcribed locus, weakly similar to NP_010015.2 serine/threonine protein kinase most similar to cyclic nucleotide-dependent protein kinase subfamily and the protein kinase C subfamily; Kin82p [Saccharomyces cerevisiae]
LesAffx.38395.2.S1_at	AI488594	AI48859	251	646	755	2.57	3.01	---
LesAffx.50112.1.S1_at	AI898985	TC23361	1305	3353	3725	2.57	2.85	Transcribed locus, weakly similar to NP_001047062.1 [Oryza sativa (japonica cultivar-group)]
Les.2682.1.S1_at	CN385606	TC22504	343	881	602	2.57	1.75	Transcribed locus
Les.2591.1.S1_at	M80608.1		1880	4800	9661	2.55	5.14	beta-1,3-glucanase
Les.4528.1.S1_at	AJ635324.1	TC22837	13	34	24	2.55	1.78	polyphenol oxidase A
LesAffx.58326.1.A1_at	CD002272	TC23714	307	783	1129	2.55	3.68	Transcribed locus, weakly similar to NP_001042998.1 [Oryza sativa (japonica cultivar-group)]
LesAffx.51158.2.S1_at	AI898095	TC23014	166	421	338	2.53	2.03	Transcribed locus, weakly similar to NP_001048008.1 [Oryza sativa (japonica cultivar-group)]
LesAffx.58502.1.S1_at	BM412364	TC22738	401	1011	674	2.52	1.68	Transcribed locus, weakly similar to NP_001042057.1 [Oryza sativa (japonica cultivar-group)]
Les.2629.1.A1_at	BG628013	TC22785	7	18	11	2.52	1.56	Transcribed locus, moderately similar to NP_001049773.1 [Oryza sativa (japonica cultivar-group)]
Les.2417.1.S1_at	AI779519	TC23220	310	778	555	2.51	1.79	Transcribed locus
LesAffx.47116.1.S1_at	AW224087	TC23403	1041	2611	2081	2.51	2	Transcribed locus, weakly similar to XP_001089495.1 cystinosis, nephropathic isoform 1 [Macaca mulatta]
Les.2212.1.A1_at	BG735110	BG73511	144	361	219	2.51	1.52	Lycopersicon esculentum clone 133400R, mRNA sequence
Les.4861.1.S1_at	BT012784.1	TC23346	220	551	401	2.5	1.82	Lycopersicon esculentum clone 113774R, mRNA sequence
LesAffx.64978.1.S1_at	CN385434	FS18759	749	1871	1681	2.5	2.24	Transcribed locus, weakly similar to NP_001054079.1 [Oryza sativa (japonica cultivar-group)]
LesAffx.53698.1.S1_at	BG127688	EG55334	623	1542	1272	2.48	2.04	Transcribed locus, weakly similar to NP_001042476.1 [Oryza sativa (japonica cultivar-group)]
LesAffx.1001.1.S1_at	BM535807	TC22526	352	870	633	2.47	1.8	---
LesAffx.59059.1.S1_at	AW622913	TC22426	336	823	734	2.45	2.19	Transcribed locus, weakly similar to NP_001048067.1 [Oryza sativa (japonica cultivar-group)]

Les.2668.2.A1_at	BG631544	TC23628	61	148	166	2.45	2.75	Transcribed locus, weakly similar to NP_001042430.1 [Oryza sativa (japonica cultivar-group)]
LesAffx.71530.1.S1_at	BI930780	TC23505	1150	2807	2781	2.44	2.42	Transcribed locus, weakly similar to NP_001048090.1 [Oryza sativa (japonica cultivar-group)]
LesAffx.59507.1.S1_at	BI203990	TC21999	460	1122	959	2.44	2.09	Transcribed locus, weakly similar to NP_001059980.1 [Oryza sativa (japonica cultivar-group)]
LesAffx.70414.1.S1_at	AW034339	TC21936	1829	4453	2974	2.43	1.63	Transcribed locus, weakly similar to NP_001045308.1 [Oryza sativa (japonica cultivar-group)]
LesAffx.32607.1.S1_at	BI206284	TC23179	10	23	20	2.43	2.06	---
Les.5091.1.A1_at	BT013255.1	TC22888	213	519	225	2.43	1.06	Lycopersicon esculentum clone 134773R, mRNA sequence
LesAffx.10955.3.S1_at	AW035238	TC22366	325	788	376	2.43	1.16	Transcribed locus, moderately similar to NP_001043233.1 [Oryza sativa (japonica cultivar-group)]
Les.54.1.S1_at	AW218809	TC22203	240	580	1351	2.42	5.63	PR-1a1 protein
Les.5433.1.S1_at	BT013909.1	TC22191	312	755	737	2.42	2.36	Lycopersicon esculentum clone 132906R, mRNA sequence
Les.5481.1.S1_at	BT014001.1	TC21763	994	2399	2969	2.41	2.99	Lycopersicon esculentum clone 133050F, mRNA sequence
LesAffx.20450.1.S1_at	AW223514	TC21776	1920	4629	4135	2.41	2.15	Transcribed locus, weakly similar to NP_001042868.1 [Oryza sativa (japonica cultivar-group)]
LesAffx.45975.1.S1_at	BI209975	BI20997	335	804	714	2.4	2.13	---
Les.593.1.A1_at	BG626426	BG62642	71	170	124	2.4	1.76	Transcribed locus
Les.3667.1.S1_at	U13054.1	TC21851	1214	2906	4242	2.39	3.5	endo-1,4-beta-glucanase precursor
LesAffx.52437.1.S1_at	BI925563	TC23341	488	1166	397	2.39	0.81	Transcribed locus, weakly similar to NP_001044627.1 [Oryza sativa (japonica cultivar-group)]
LesAffx.66744.1.S1_at	CN385873	TC23736	1841	4392	4844	2.39	2.63	Xyloglucan endotransglucosylase-hydrolase XTH3
Les.4523.1.S1_at	AY497475.1	TC21776	760	1808	1760	2.38	2.32	xyloglucan endotransglucosylase-hydrolase XTH5
LesAffx.3467.1.S1_at	CN384781	TC23179	695	1653	1638	2.38	2.36	Transcribed locus, weakly similar to NP_001047645.1 [Oryza sativa (japonica cultivar-group)]
LesAffx.55476.1.S1_at	BM410473	TC22139	80	188	139	2.36	1.75	Transcribed locus, weakly similar to XP_001084501.1 hypothetical protein isoform 2 [Macaca mulatta]
LesAffx.60722.1.S1_at	AW092522	TC22954	521	1229	968	2.36	1.86	Transcribed locus, weakly similar to NP_001046772.1 [Oryza sativa (japonica cultivar-group)]
Les.5916.1.S1_at	AJ715788.1	TC23545	412	971	979	2.36	2.38	anaerobic basic leucine zipper protein
LesAffx.10955.2.S1_at	BI923362	TC22366	200	473	232	2.36	1.16	Transcribed locus, moderately similar to NP_001043233.1 [Oryza sativa (japonica cultivar-group)]
LesAffx.24042.1.S1_at	CN385197	DB67876	743	1748	2727	2.35	3.67	Transcribed locus, weakly similar to NP_663449.2 P450-like [Mus musculus]
LesAffx.3467.1.A1_at	CN384781	TC23179	353	827	637	2.35	1.81	Transcribed locus, weakly similar to NP_001047645.1 [Oryza sativa (japonica cultivar-group)]

Les.4609.1.S1_at	BT013258.1	TC23090	382	897	685	2.35	1.79	Lycopersicon esculentum clone 134793F, mRNA sequence
LesAffx.57163.3.A1_at	AI486803	TC22802	171	400	68	2.34	0.4	Transcribed locus, weakly similar to NP_001042186.1 [Oryza sativa (japonica cultivar-group)]
Les.217.1.S1_at	AF243180.1	TC22069	723	1694	2128	2.34	2.94	dicyanin
Les.140.1.S1_at	L08830.1	TC21735	2874	6728	6488	2.34	2.26	glucose-regulated protein 78
LesAffx.2934.1.S1_at	AI775080	TC22515	1419	3322	2515	2.34	1.77	CDNA, clone: FC19BE12, HTC in fruit
Les.4739.1.S1_at	AY622990.1	TC21730	2395	5594	4296	2.34	1.79	UDP-glucose:protein transglucosylase-like protein SlUPTG1
Les.5958.1.S1_at	CN384731	TC23315	566	1319	1477	2.33	2.61	Transcribed locus, weakly similar to NP_998286.2 kinase CKM3 [Danio rerio]
LesAffx.51298.1.S1_at	BI422808	TC21917	55	129	222	2.33	4.01	Transcribed locus, weakly similar to NP_001044747.1 [Oryza sativa (japonica cultivar-group)]
Les.5848.1.A1_at	BG631837	TC23527	553	1287	925	2.33	1.67	Transcribed locus, weakly similar to XP_001121814.1 similar to CG9009-PA [Apis mellifera]
LesAffx.22466.1.S1_at	AI774712	TC23276	151	352	266	2.33	1.76	Transcribed locus, weakly similar to NP_001063376.1 [Oryza sativa (japonica cultivar-group)]
LesAffx.62232.1.S1_at	AW649113	TC23914	43	99	133	2.32	3.11	Transcribed locus, weakly similar to NP_001048167.1 [Oryza sativa (japonica cultivar-group)]
			5	11	6	2.32	1.34	---
LesAffx.10176.1.S1_at	CN385573	TC22551	33	76	80	2.32	2.44	Transcribed locus, weakly similar to NP_001042986.1 [Oryza sativa (japonica cultivar-group)]
Les.4264.1.A1_s_at	L24012.1	TC22590	271	629	358	2.32	1.32	gamma-aminobutyrate transaminase subunit precursor isozyme 3
LesAffx.34363.1.S1_at	BE461912	TC24206	59	137	98	2.32	1.67	---
LesAffx.51285.1.S1_at	CN385118	TC24451	84	193	234	2.3	2.78	---
Les.31.1.S1_s_at	AF110518.1	NP00011	897	2064	1153	2.3	1.29	ethylene-inducible CTR1-like protein kinase
LesAffx.6125.1.S1_at	CK468687	TC22217	670	1541	1431	2.3	2.14	Transcribed locus, weakly similar to NP_001042057.1 [Oryza sativa (japonica cultivar-group)]
LesAffx.63189.1.S1_at	BF113930	TC23356	96	221	206	2.3	2.15	Transcribed locus, weakly similar to NP_001042986.1 [Oryza sativa (japonica cultivar-group)]
Les.1115.1.A1_at	BG629468	BG62946	22	50	66	2.28	3.01	Transcribed locus
LesAffx.1091.1.S1_at	BI933005	TC22991	126	287	242	2.28	1.92	Transcribed locus, weakly similar to XP_001115960.1 hydroxysteroid (17-beta) dehydrogenase 8 [Macaca mulatta]
LesAffx.16086.1.S1_at	AI895361	TC22125	165	376	1193	2.28	7.24	Transcribed locus, weakly similar to NP_001045452.1 [Oryza sativa (japonica cultivar-group)]
LesAffx.32379.1.S1_at	BI421517	BI42151	267	607	421	2.28	1.58	Transcribed locus, weakly similar to NP_001048067.1 [Oryza sativa (japonica cultivar-group)]
LesAffx.805.1.S1_at	BI203776	TC23079	869	1970	2445	2.27	2.81	Transcribed locus, weakly similar to XP_001091858.1 DnaJ (Hsp40) homolog, subfamily B, member 11 [Macaca mulatta]

Les.4852.1.S1_at	BT012761.1	TC22901	1289	2923	3527	2.27	2.74	Lycopersicon esculentum clone 113705F, mRNA sequence
LesAffx.32359.1.S1_at	CK468683	TC22049	554	1254	1012	2.26	1.83	Transcribed locus, weakly similar to NP_001042342.1 [Oryza sativa (japonica cultivar-group)]
LesAffx.1244.2.A1_at	CK715642	TC22520	387	873	1017	2.26	2.63	Transcribed locus, moderately similar to NP_001046015.1 [Oryza sativa (japonica cultivar-group)]
Les.4483.1.S1_at	AY498713.1	TC21812	1438	3225	1633	2.24	1.14	NAC domain protein
LesAffx.1244.2.S1_at	CK715642	TC22520	141	317	308	2.24	2.18	Transcribed locus, moderately similar to NP_001046015.1 [Oryza sativa (japonica cultivar-group)]
Les.5152.1.S1_at	BT013393.1	TC24469	338	756	794	2.24	2.35	Lycopersicon esculentum clone 135647R, mRNA sequence
LesAffx.344.3.S1_at	BI921701	TC21769	267	597	641	2.24	2.4	Transcribed locus, weakly similar to NP_190972.1 protein [Arabidopsis thaliana]
LesAffx.63739.1.S1_at	AW033134	TC21965	491	1096	860	2.23	1.75	Transcribed locus, weakly similar to NP_001043665.1 [Oryza sativa (japonica cultivar-group)]
LesAffx.13053.1.S1_at	AW032379	TC22409	531	1183	1008	2.23	1.9	Transcribed locus, weakly similar to XP_001108084.1 similar to aldehyde dehydrogenase 1 family, member L1 [Macaca mulatta]
Les.5711.1.S1_at	BT014429.1	TC21831	276	615	1194	2.23	4.32	Lycopersicon esculentum clone 133747F, mRNA sequence
LesAffx.61012.2.S1_at	BG132173	TC23271	188	418	510	2.23	2.72	Transcribed locus, weakly similar to NP_001042750.1 [Oryza sativa (japonica cultivar-group)]
LesAffx.71532.1.S1_at	AI772343	TC22607	2245	4996	4262	2.22	1.9	Transcribed locus, weakly similar to NP_001041930.1 [Oryza sativa (japonica cultivar-group)]
Les.5177.1.S1_at	BT013446.1	TC24190	29	65	422	2.22	14.47	Lycopersicon esculentum clone 132101F, mRNA sequence
Les.4105.1.A1_at	BG735198	BG73519	3	7	4	2.22	1.11	Transcribed locus
Les.4934.1.S1_at	BT012932.1	TC22421	104	231	288	2.22	2.77	Lycopersicon esculentum clone 114095R, mRNA sequence
LesAffx.1244.1.S1_at	BG126114	TC22939	327	723	847	2.21	2.59	Transcribed locus, moderately similar to NP_001046015.1 [Oryza sativa (japonica cultivar-group)]
LesAffx.59059.2.S1_at	AW651016	TC22426	211	466	398	2.21	1.89	Transcribed locus, weakly similar to NP_001048067.1 [Oryza sativa (japonica cultivar-group)]
LesAffx.10955.1.S1_at	BI422058	TC22366	247	547	271	2.21	1.1	Transcribed locus, moderately similar to NP_001043233.1 [Oryza sativa (japonica cultivar-group)]
Les.3155.1.S1_at	BT013204.1	TC21967	614	1355	1476	2.21	2.4	Lycopersicon esculentum clone 134378F, mRNA sequence
Les.3545.1.S1_at	Y14809.1	TC22310	1481	3266	1655	2.21	1.12	beta-carotene hydroxylase
Les.3018.1.S1_at	BT013902.1	TC22606	3263	7196	6124	2.21	1.88	Lycopersicon esculentum clone 132899F, mRNA sequence
LesAffx.14542.1.S1_at	AW647748		14	31	26	2.2	1.83	---
LesAffx.48836.1.S1_at	AW623752	TC23087	84	185	175	2.2	2.09	---
Les.3833.1.S1_at	AY098737.2	TC22193	228	500	785	2.2	3.45	TAGL12 transcription factor
LesAffx.58365.1.S1_at	BM536079	TC22511	263	577	596	2.2	2.26	---

LesAffx.49321.1.S1_at	AI486965	TC22769	19	42	30	2.19	1.54	Transcribed locus, weakly similar to NP_001045292.1 [Oryza sativa (japonica cultivar-group)]
Les.63.1.S1_at	AF049900.1	TC22864	67	146	730	2.19	10.97	gibberellin 20-oxidase-3
Les.5044.1.S1_at	BT013168.1	TC22109	929	2029	2574	2.18	2.77	Lycopersicon esculentum clone 134231R, mRNA sequence
Les.2073.1.S1_at	BM410320	TC23644	1880	4097	2576	2.18	1.37	Transcribed locus, weakly similar to XP_001105883.1 similar to CG7222-PA isoform 2 [Macaca mulatta]
LesAffx.56137.1.A1_at	AI490099	TC22599	109	236	732	2.17	6.72	Transcribed locus, weakly similar to NP_001041909.1 [Oryza sativa (japonica cultivar-group)]
Les.1568.1.A1_at	BG626849	TC24275	22	47	52	2.17	2.43	Transcribed locus
Les.5130.1.S1_at	BT013342.1	TC23433	148	320	445	2.16	3	Lycopersicon esculentum clone 135145F, mRNA sequence
Les.4067.1.S1_at	AY240231.1	TC22590	1399	3023	1970	2.16	1.41	gamma-aminobutyrate transaminase subunit precursor isozyme 3
LesAffx.12670.3.S1_at	BF113197	TC22012	82	176	100	2.15	1.23	Transcribed locus, weakly similar to NP_001042749.1 [Oryza sativa (japonica cultivar-group)]
LesAffx.18686.1.S1_at	BI203812	TC22685	33	70	214	2.15	6.57	Transcribed locus, weakly similar to NP_001043492.1 [Oryza sativa (japonica cultivar-group)]
Les.2817.2.S1_at	BI933507	TC24089	250	538	454	2.15	1.82	Transcribed locus, weakly similar to XP_001108114.1 similar to ATP citrate lyase isoform 1 isoform 2 [Macaca mulatta]
LesAffx.50466.1.S1_at	AI780511	TC23061	584	1255	1340	2.15	2.3	CDNA, clone: FC02AC08, HTC in fruit
Les.2817.1.S1_at	BF050945	TC24089	1462	3144	2460	2.15	1.68	Transcribed locus, weakly similar to XP_001108114.1 similar to ATP citrate lyase isoform 1 isoform 2 [Macaca mulatta]
Les.254.1.S1_at	AY081905.1	TC21784	411	883	1022	2.15	2.49	N-hydroxycinnamoyl-CoA:tyramine N-hydroxycinnamoyl transferase THT1-3
LesAffx.58193.1.S1_at	CK715571	TC23961	311	667	344	2.14	1.11	Transcribed locus, weakly similar to NP_001049863.1 [Oryza sativa (japonica cultivar-group)]
LesAffx.51158.2.A1_at	AI898095	TC23014	140	301	199	2.14	1.42	Transcribed locus, weakly similar to NP_001048008.1 [Oryza sativa (japonica cultivar-group)]
LesAffx.3460.1.S1_at	CN384958	EG55377	129	275	373	2.14	2.9	---
Les.4803.1.S1_at	BT013418.1	TC21851	98	210	149	2.13	1.52	Lycopersicon esculentum clone 132056R, mRNA sequence
LesAffx.60242.1.S1_at	BI207558	TC23660	337	717	666	2.13	1.98	Transcribed locus, moderately similar to NP_001058564.1 [Oryza sativa (japonica cultivar-group)]
Les.1054.1.S1_at	BM412386	TC21841	753	1604	1151	2.13	1.53	Transcribed locus, weakly similar to XP_001121618.1 similar to CG10621-PA [Apis mellifera]
Les.2988.1.S1_at	CK715255	TC22567	2275	4838	1845	2.13	0.81	Sucrose transporter
Les.3543.1.S1_at	AJ011914.1	TC23261	97	207	163	2.12	1.68	THY5 protein
Les.3526.1.S1_a_at	AF198259.1	TC22267	641	1360	1071	2.12	1.67	calmodulin-binding diacylglycerol kinase
LesAffx.62131.1.S1_at	BE437087	TC23252	1288	2730	939	2.12	0.73	Transcribed locus, weakly similar to XP_001081745.1 similar to threonine aldolase 1 [Rattus norvegicus]

LesAffx.69747.1.S1_at	BM536068	TC21831	1291	2732	2601	2.12	2.01	Transcribed locus, weakly similar to NP_001042241.1 [Oryza sativa (japonica cultivar-group)]
LesAffx.70928.1.S1_at	BI209612	TC23336	913	1932	1916	2.12	2.1	Transcribed locus, moderately similar to NP_001044227.1 [Oryza sativa (japonica cultivar-group)]
Les.960.1.A1_at	BG628420	BG62842	95	201	138	2.11	1.46	Transcribed locus
Les.3693.1.S1_at	AF426174.1	TC22813	368	777	1117	2.11	3.03	blind
LesAffx.66163.1.S1_at	AW222774	TC22788	418	881	947	2.11	2.27	Transcribed locus, weakly similar to NP_001046543.1 [Oryza sativa (japonica cultivar-group)]
Les.2688.1.S1_at	AJ560646.1	TC21917	268	565	2173	2.11	8.1	expansin11
LesAffx.15850.1.S1_at	AW030423	TC23653	391	825	1072	2.11	2.74	Transcribed locus, weakly similar to NP_001046323.1 [Oryza sativa (japonica cultivar-group)]
Les.1897.1.A1_at	BG630037	TC22522	55	115	103	2.11	1.87	Transcribed locus, weakly similar to NP_001064869.1 [Oryza sativa (japonica cultivar-group)]
LesAffx.51554.1.S1_at	AW093474	GO37601	989	2083	817	2.11	0.83	---
LesAffx.51554.1.A1_at	AW093474	GO37601	722	1520	460	2.11	0.64	---
LesAffx.60006.1.S1_at	BG643009	TC22674	246	517	738	2.1	3	Transcribed locus, weakly similar to NP_001055441.1 [Oryza sativa (japonica cultivar-group)]
LesAffx.3244.2.S1_at	CD002790	TC22222	501	1055	1504	2.1	3	Transcribed locus, weakly similar to NP_001045327.1 [Oryza sativa (japonica cultivar-group)]
Les.2137.1.S1_at	AY359965.1	TC21714	1105	2321	2264	2.1	2.05	EIX receptor 1
Les.3186.1.S1_at	BG123422	TC22761	21	45	36	2.1	1.69	Transcribed locus
Les.244.2.S1_at	AI485696	TC22935	112	235	175	2.09	1.56	Calmodulin
LesAffx.63980.1.S1_at	AI780160	TC22905	418	874	854	2.09	2.04	Transcribed locus, weakly similar to NP_001048257.1 [Oryza sativa (japonica cultivar-group)]
LesAffx.69306.1.S1_at	BI203295	TC23093	104	218	210	2.09	2.02	Transcribed locus, weakly similar to NP_849966.2 protein [Arabidopsis thaliana]
Les.4140.1.S1_at	AY192367.1	TC22892	905	1877	1125	2.07	1.24	Lycopersicon esculentum clone 134153F, mRNA sequence
Les.1429.1.A1_at	BG631546	BG63154	82	171	104	2.07	1.26	Transcribed locus
LesAffx.69808.1.S1_at	CN385324	TC22525	42	87	94	2.07	2.25	Transcribed locus, weakly similar to NP_001043374.1 [Oryza sativa (japonica cultivar-group)]
Les.1054.2.S1_at	BE436393	TC23657	900	1864	1431	2.07	1.59	Transcribed locus, weakly similar to XP_001121618.1 similar to CG10621-PA [Apis mellifera]
Les.3610.1.S1_at	BG629712	TC23477	78	161	770	2.07	9.89	glycine rich protein
LesAffx.43793.1.A1_at	BI206057	TC23097	53	109	120	2.07	2.28	Transcribed locus, weakly similar to XP_001113600.1 KDEL (Lys-Asp-Glu-Leu) endoplasmic reticulum protein retention receptor 1 [Macaca mulatta]
LesAffx.71660.2.S1_at	AW218025	TC24516	3	5	3	2.06	0.99	---
Les.5917.1.S1_at	AJ715790.1	TC21740	3582	7368	8653	2.06	2.42	1-aminocyclopropane-1-carboxylate oxidase

LesAffx.42809.1.S1_at	AW041173	AW04117	10	21	16	2.06	1.53	---
Les.23.1.S1_at	AF011555.1	TC22224	2430	4998	3993	2.06	1.64	jasmonic acid 2
LesAffx.58193.1.A1_at	CK715571	TC23961	666	1370	857	2.06	1.29	Transcribed locus, weakly similar to NP_001049863.1 [Oryza sativa (japonica cultivar-group)]
Les.3493.1.S1_at	AY013255.1	TC21805	800	1645	1229	2.05	1.54	phospholipase PLDb1
LesAffx.63074.1.A1_at	CN385213	TC22246	147	302	217	2.05	1.48	Transcribed locus, weakly similar to NP_001047524.1 [Oryza sativa (japonica cultivar-group)]
LesAffx.2226.1.S1_at	BI422950	TC23401	352	724	817	2.05	2.32	Transcribed locus, moderately similar to XP_001100144.1 similar to ribonucleotide reductase M2 B (TP53 inducible) isoform 3 [Macaca mulatta]
LesAffx.10495.1.S1_at	AW035719	TC23875	437	896	767	2.05	1.76	Transcribed locus, weakly similar to XP_001098727.1 similar to lactate dehydrogenase A-like 6B [Macaca mulatta]
Les.4518.3.A1_at	BG734737	TC22852	850	1739	1892	2.05	2.23	Transcribed locus, moderately similar to XP_001085048.1 similar to 60S ribosomal protein L37a [Macaca mulatta]
Les.5293.1.S1_at	BT013651.1	AW09681	791	1619	1139	2.05	1.44	Lycopersicon esculentum clone 132458F, mRNA sequence
LesAffx.9245.1.S1_at	BE463039	TC24138	976	1996	1880	2.04	1.93	CDNA, clone: FC03AB07, HTC in fruit
LesAffx.37916.1.S1_at	BM536108	TC23806	81	165	243	2.04	2.98	Transcribed locus, weakly similar to NP_001048479.1 [Oryza sativa (japonica cultivar-group)]
Les.2667.2.S1_at	AW621251	TC22610	3090	6286	4519	2.03	1.46	Transcribed locus, weakly similar to NP_001042221.1 [Oryza sativa (japonica cultivar-group)]
LesAffx.32379.2.S1_at	AI896619	BI42151	222	452	327	2.03	1.47	Transcribed locus, weakly similar to NP_001048067.1 [Oryza sativa (japonica cultivar-group)]
Les.638.1.S1_at	BT012771.1	TC21816	2837	5763	5816	2.03	2.05	Lycopersicon esculentum clone 113749F, mRNA sequence
Les.4895.1.S1_at	BT012856.1	TC23595	2066	4196	2688	2.03	1.3	Lycopersicon esculentum clone 113942F, mRNA sequence
LesAffx.22812.2.S1_at	BI204920	TC22300	150	304	167	2.03	1.12	Transcribed locus, weakly similar to NP_001061950.1 [Oryza sativa (japonica cultivar-group)]
LesAffx.22100.1.S1_at	AW216933	TC22078	204	413	490	2.03	2.41	Transcribed locus, weakly similar to XP_001061027.1 similar to Heat shock factor protein 1 (HSF 1) (Heat shock transcription factor 1) (HSTF 1) [Rattus norvegicus]
Les.3759.1.S1_at	Z46654.1	TC21776	1038	2103	2358	2.03	2.27	late embryogenesis (Lea)-like protein
LesAffx.56070.1.S1_at	BF113259	TC23846	580	1174	933	2.02	1.61	Transcribed locus, weakly similar to NP_001043915.1 [Oryza sativa (japonica cultivar-group)]
			2	4	3	2.02	1.76	---
LesAffx.57454.1.S1_at	BI923132	TC22139	56	113	226	2.02	4.05	Transcribed locus, weakly similar to XP_001109440.1 similar to calmodulin 1 [Macaca mulatta]
LesAffx.62967.1.S1_at	BG128004	TC21896	2371	4792	4654	2.02	1.96	Transcribed locus, weakly similar to NP_001047054.1 [Oryza sativa (japonica cultivar-group)]
LesAffx.15898.1.S1_at	AW649847	TC21728	731	1478	909	2.02	1.24	UDP-xylose phenolic glycosyltransferase

LesAffx.20391.1.S1_at	BF176422	TC22123	349	703	488	2.02	1.4	Transcribed locus, weakly similar to NP_001062790.1 [Oryza sativa (japonica cultivar-group)]
Les.176.1.S1_at	U38464.1	TC21796	4001	8063	7407	2.02	1.85	small GTP-binding protein
LesAffx.64861.1.S1_at	BG127249	TC22312	222	446	386	2.02	1.74	Transcribed locus, weakly similar to NP_065724.1 protein RAB-22A [Homo sapiens]
Les.1238.2.A1_at	BG630266	TC23474	79	159	149	2.01	1.89	Transcribed locus, weakly similar to NP_001042653.1 [Oryza sativa (japonica cultivar-group)]
LesAffx.51285.1.A1_at	CN385118	TC24451	46	93	98	2.01	2.13	---
Les.3289.1.S1_at	AY240229.1	TC21835	3304	6640	5264	2.01	1.59	gamma-aminobutyrate transaminase subunit precursor isozyme 1
Les.4510.3.A1_at	AI777050	TC24158	15	30	27	2.01	1.84	Transcribed locus, weakly similar to XP_001108187.1 similar to 40S ribosomal protein S25 [Macaca mulatta]
LesAffx.22297.1.S1_at	AW041000	TC21723	734	1473	1089	2.01	1.48	CYP710A7 mRNA for sterol 22-desaturase
LesAffx.47529.1.S1_at	BG131749	TC24163	60	121	92	2.01	1.53	---
LesAffx.63074.1.S1_at	AW217297	TC22246	172	345	309	2.01	1.8	Transcribed locus, weakly similar to NP_001047524.1 [Oryza sativa (japonica cultivar-group)]
Les.797.1.S1_at	X92854.1	TC24488	734	1470	1803	2	2.46	Exon 1 of AMT1 gene
LesAffx.51274.1.S1_at	CN385340	TC22133	255	510	574	2	2.25	Transcribed locus, moderately similar to NP_001044266.1 [Oryza sativa (japonica cultivar-group)]
LesAffx.56137.1.S1_at	AI490099	TC22599	452	904	3059	2	6.77	Transcribed locus, weakly similar to NP_001041909.1 [Oryza sativa (japonica cultivar-group)]
Les.1159.1.S1_at	AW096608	TC23740	146	291	284	2	1.95	Transcribed locus, weakly similar to NP_190772.1 protein [Arabidopsis thaliana]
			2	3	2	2	1.04	---
Les.5481.1.A1_at	BT014001.1	TC21763	651	1301	1193	2	1.83	Lycopersicon esculentum clone 133050F, mRNA sequence
LesAffx.26180.1.S1_at	BG132890	BG13289	58	116	255	2	4.37	---
LesAffx.59842.1.S1_at	BI205718	TC21823	140	147	947	1.05	6.75	Transcribed locus, weakly similar to NP_001042297.1 [Oryza sativa (japonica cultivar-group)]
Les.3625.1.S1_at	L22188.1	TC23715	613	568	3444	0.93	5.62	RSI-1 protein
LesAffx.65135.1.S1_at	CK468698	TC23660	23	29	122	1.24	5.24	Transcribed locus
Les.1397.1.A1_at	BG631327	BG63132	226	186	1128	0.82	4.99	Transcribed locus, weakly similar to NP_001042152.1 [Oryza sativa (japonica cultivar-group)]
Les.50.1.S1_at	X79338.1	TC22030	521	936	2395	1.79	4.59	ribonuclease
Les.191.1.S1_at	AF548376.1	TC21771	557	377	2516	0.68	4.52	expansin
Les.1.1.S1_at	AF154421.1	TC21715	1409	1879	6050	1.33	4.29	beta-galactosidase
LesAffx.51483.1.A1_at	CK348350	CK34835	22	18	91	0.83	4.17	---
LesAffx.39466.1.S1_at	BI203178	TC24216	176	306	706	1.74	4.02	---
Les.4693.1.S1_at	M69247.1	TC23231	253	268	967	1.06	3.83	pathogenesis-related protein P4

Les.3396.2.S1_at	AW221386	TC22284	1524	2733	5814	1.79	3.81	Transcribed locus, weakly similar to NP_001042402.1 [Oryza sativa (japonica cultivar-group)]
Les.5960.1.S1_at	AY731066.1	TC22652	775	1013	2890	1.31	3.73	LeTIP protein
Les.3042.2.S1_at	AW934591	BE46335	168	325	613	1.93	3.65	Transcribed locus, weakly similar to NP_001062055.1 [Oryza sativa (japonica cultivar-group)]
LesAffx.59625.1.S1_at	BI204607	TC22866	176	248	628	1.41	3.57	Transcribed locus, weakly similar to NP_001049721.1 [Oryza sativa (japonica cultivar-group)]
Les.3707.1.S1_at	AF022013.1	TC24298	51	43	182	0.84	3.56	IAA2 protein
LesAffx.59592.1.S1_at	BI210118	TC22019	20	14	69	0.68	3.5	Transcribed locus, weakly similar to NP_065956.1 dehydrogenase 14 (all-trans and 9-cis) [Homo sapiens]
LesAffx.37714.1.A1_at	CK720577	TC24072	36	47	124	1.32	3.45	Transcribed locus, weakly similar to NP_001046387.1 [Oryza sativa (japonica cultivar-group)]
LesAffx.68092.1.S1_at	BE459012	TC22815	265	490	914	1.85	3.45	Transcribed locus, weakly similar to NP_001044783.1 [Oryza sativa (japonica cultivar-group)]
Les.4346.1.S1_at	AF020390.2	TC21717	756	618	2605	0.82	3.44	ss-galactosidase
LesAffx.63172.1.S1_at	AW031388	TC22898	338	611	1158	1.81	3.43	Transcribed locus, moderately similar to NP_001044907.1 [Oryza sativa (japonica cultivar-group)]
Les.4026.1.S1_at	AY155579.1	TC21731	1006	1674	3436	1.66	3.42	xyloglucan-specific fungal endoglucanase inhibitor protein precursor
Les.3583.1.A1_at	Y15846.1	TC22445	94	96	316	1.01	3.34	TSI-1 protein
Les.3700.1.S1_at	AY026343.1	TC22149	439	519	1466	1.18	3.34	Lycopersicon esculentum non-symbiotic hemoglobin class 1 (Glb1)
LesAffx.68294.1.S1_at	BM408981	TC22580	95	83	316	0.88	3.33	Transcribed locus, weakly similar to NP_001046934.1 [Oryza sativa (japonica cultivar-group)]
Les.3396.1.A1_at	AW224542	TC22094	1086	1841	3599	1.69	3.31	Transcribed locus, weakly similar to NP_001042402.1 [Oryza sativa (japonica cultivar-group)]
LesAffx.62690.1.S1_at	BI206793	TC22770	352	688	1155	1.96	3.28	Transcribed locus, weakly similar to NP_001043439.1 [Oryza sativa (japonica cultivar-group)]
Les.3706.1.S1_at	AF022014.1	TC21953	298	313	943	1.05	3.17	IAA3 protein
LesAffx.59622.1.S1_at	BI210866	TC22863	377	469	1190	1.24	3.16	Transcribed locus, weakly similar to NP_565922.1 (EARLY FLOWERING 4) [Arabidopsis thaliana]
LesAffx.60109.1.S1_at	BI204244	TC22478	696	672	2191	0.97	3.15	Transcribed locus, weakly similar to NP_001042055.1 [Oryza sativa (japonica cultivar-group)]
Les.4317.1.S1_at	AW625684	TC23953	2359	4290	7220	1.82	3.06	asparagine synthetase
Les.4718.1.S1_at	BT013487.1	BI92390	407	472	1235	1.16	3.04	Lycopersicon esculentum clone 132171F, mRNA sequence
Les.3669.1.S1_at	L19762.1	TC21717	501	486	1505	0.97	3	sucrose synthase
Les.4948.1.S1_at	BT012962.1	TC23041	252	462	726	1.84	2.89	Lycopersicon esculentum clone 114152R, mRNA sequence
Les.4873.1.S1_at	BT012806.1	TC23935	634	799	1828	1.26	2.88	Lycopersicon esculentum clone 113825R, mRNA sequence

Les.3042.1.S1_at	BI926173	TC23899	65	115	185	1.76	2.85	Transcribed locus, weakly similar to NP_001062055.1 [Oryza sativa (japonica cultivar-group)]
LesAffx.34771.1.S1_at	AW930136	AW93013	643	888	1832	1.38	2.85	---
Les.1044.1.A1_at	BG629030	TC23609	121	112	342	0.93	2.84	Transcribed locus
Les.4976.1.S1_at	BT013033.1	TC22335	273	306	771	1.12	2.82	Lycopersicon esculentum clone 114281R, mRNA sequence
LesAffx.15953.1.S1_at	AW033344	TC23158	34	54	97	1.57	2.81	Transcribed locus, weakly similar to NP_181965.1 protein [Arabidopsis thaliana]
Les.5597.1.S1_at	BT014226.1	AW09450	49	55	138	1.12	2.81	Lycopersicon esculentum clone 133421F, mRNA sequence
Les.2642.1.S1_at	CN384581	TC22309	3005	5095	8423	1.7	2.8	CDNA, clone: FC01AD09, HTC in fruit
LesAffx.344.10.S1_at	AI773570	AI77357	1106	1678	3085	1.52	2.79	Transcribed locus, weakly similar to XP_001089325.1 similar to cellular repressor of E1A-stimulated genes [Macaca mulatta]
Les.4625.1.S1_at	BT013972.1	AW03556	1365	2569	3793	1.88	2.78	Lycopersicon esculentum clone 133016F, mRNA sequence
Les.3460.1.S1_at	AF506005.1	TC21723	1409	2386	3907	1.69	2.77	acid invertase
Les.909.1.A1_at	BG628078	TC23979	31	42	83	1.36	2.74	Transcribed locus
Les.5343.1.S1_at	BT013743.1		85	103	231	1.21	2.72	Lycopersicon esculentum clone 132606R, mRNA sequence
Les.2643.1.S1_at	BG128842	TC23690	234	350	635	1.5	2.71	Transcribed locus
LesAffx.62690.2.S1_at	CD003021	CD00302	738	1315	2001	1.78	2.71	Transcribed locus, weakly similar to NP_001043439.1 [Oryza sativa (japonica cultivar-group)]
Les.2616.1.A1_at	BG628893	TC23909	142	142	386	1	2.71	Transcribed locus, weakly similar to NP_565764.1 protein [Arabidopsis thaliana]
Les.4040.1.S1_at	X83420.1	AW09681	2105	3902	5691	1.85	2.7	Alu I-fragment
LesAffx.57437.1.S1_at	BI928709	TC21886	366	577	980	1.58	2.68	Transcribed locus, weakly similar to NP_001042998.1 [Oryza sativa (japonica cultivar-group)]
Les.3663.1.S1_at	X55193.1	TC22989	385	219	1023	0.57	2.66	9612 protein
LesAffx.17387.1.S1_at	BM534931	BM53493	430	831	1138	1.93	2.65	---
LesAffx.65882.1.S1_at	BG129533	TC23537	91	86	240	0.94	2.64	Transcribed locus, weakly similar to NP_567804.1 protein [Arabidopsis thaliana]
LesAffx.69492.1.S1_at	AW443978	AW44397	30	33	80	1.09	2.64	---
LesAffx.36979.1.S1_at	AI775450	TC22380	430	784	1134	1.82	2.64	Transcribed locus, weakly similar to XP_001104766.1 calmodulin-like 5 [Macaca mulatta]
LesAffx.15850.1.S1_a_at	AW030423	TC23653	122	202	319	1.65	2.61	Transcribed locus, weakly similar to NP_001046323.1 [Oryza sativa (japonica cultivar-group)]
Les.4383.1.S1_at	M21775.1	BM41072	32	38	82	1.2	2.59	fruit-specific protein
LesAffx.7894.1.S1_at	AW933852	TC23795	1011	1110	2598	1.1	2.57	Transcribed locus, weakly similar to XP_001091674.1 similar to aspartate aminotransferase 2 precursor [Macaca mulatta]
Les.3597.1.S1_at	S40549.1	TC22411	956	492	2449	0.51	2.56	deoxyuridine triphosphatase
Les.3415.3.S1_at	AI779314	TC24218	765	578	1947	0.76	2.54	CDNA, clone: FC24DE01, HTC in fruit
LesAffx.3244.1.S1_at	BI934720	TC22222	951	1803	2414	1.9	2.54	Transcribed locus, weakly similar to NP_001045327.1 [Oryza sativa (japonica cultivar-group)]

Les.1350.1.A1_at	BG631017	TC24319	83	60	210	0.72	2.51	Transcribed locus
Les.3232.1.A1_at	BG627277	TC21879	146	159	367	1.09	2.51	Transcribed locus, weakly similar to NP_001043730.1 [Oryza sativa (japonica cultivar-group)]
Les.4501.1.S1_at	CK468710	TC24359	490	654	1223	1.34	2.5	Transcribed locus, weakly similar to NP_001043332.1 [Oryza sativa (japonica cultivar-group)]
Les.1061.1.A1_at	AI488184	TC23906	113	138	282	1.22	2.5	Transcribed locus, weakly similar to NP_568700.1 protein [Arabidopsis thaliana]
LesAffx.33082.1.S1_at	BG133383	TC23204	50	65	123	1.3	2.47	---
LesAffx.14450.1.S1_at	AW649659	AW64965	188	167	463	0.89	2.46	---
LesAffx.23119.1.S1_at	BG127117	TC23852	105	143	259	1.35	2.46	Transcribed locus, weakly similar to NP_001064365.1 [Oryza sativa (japonica cultivar-group)]
LesAffx.15353.1.S1_at	BM967389	BM96738	44	62	107	1.42	2.45	Transcribed locus, weakly similar to NP_001046337.1 [Oryza sativa (japonica cultivar-group)]
Les.2850.1.S1_at	CN384785	TC23958	90	160	220	1.78	2.45	CDNA, clone: FC09CF05, HTC in fruit
LesAffx.62906.1.S1_at	BI923447	TC23590	66	104	162	1.57	2.45	Transcribed locus, weakly similar to NP_065266.1 translocase [Mus musculus]
LesAffx.68054.1.S1_at	AI898501	TC22471	233	331	569	1.42	2.44	Transcribed locus, weakly similar to NP_001049859.1 [Oryza sativa (japonica cultivar-group)]
Les.3747.1.S1_at	Z46675.1	NP00034	35	29	84	0.84	2.43	extensin
LesAffx.3455.2.S1_at	CN385030	TC23177	164	318	398	1.94	2.43	Transcribed locus, weakly similar to NP_001042297.1 [Oryza sativa (japonica cultivar-group)]
Les.1787.1.A1_at	BG628780	TC23942	430	630	1043	1.47	2.43	Transcribed locus, weakly similar to NP_001055064.1 [Oryza sativa (japonica cultivar-group)]
Les.2832.1.S1_at	CN384480	TC21748	3400	2219	8155	0.65	2.4	peroxidase
Les.4326.1.S1_at	AY235681.1	TC22243	160	307	382	1.91	2.38	P58IPK
Les.3591.1.S1_at	CN385613	FS19591	494	670	1162	1.36	2.35	Partial mRNA (clone SENU1)
LesAffx.3455.1.S1_at	BI421254	TC23177	82	137	191	1.68	2.34	Transcribed locus, weakly similar to NP_001042297.1 [Oryza sativa (japonica cultivar-group)]
LesAffx.6103.1.S1_at	AW647641	TC22739	62	65	144	1.05	2.33	Transcribed locus, weakly similar to NP_001042342.1 [Oryza sativa (japonica cultivar-group)]
LesAffx.49809.1.S1_at	CN384734	TC21755	217	375	506	1.73	2.33	Transcribed locus, weakly similar to NP_001045544.1 [Oryza sativa (japonica cultivar-group)]
Les.4966.1.S1_at	BT013015.1	BF17648	111	105	257	0.95	2.33	Lycopersicon esculentum clone 114253R, mRNA sequence
LesAffx.66953.1.S1_at	AW651552	TC23604	3457	5670	8050	1.64	2.33	Transcribed locus, weakly similar to NP_001042903.1 [Oryza sativa (japonica cultivar-group)]
Les.2565.3.S1_at	BI204284	TC23474	1720	3003	3999	1.75	2.32	Transcribed locus, weakly similar to XP_001087898.1 stromal cell-derived factor 2-like 1 [Macaca mulatta]
Les.3706.1.A1_at	AF022014.1	TC21953	136	147	314	1.09	2.31	IAA3 protein

LesAffx.49103.1.A1_at	AW224716	TC22913	766	1273	1772	1.66	2.31	Transcribed locus, weakly similar to NP_065615.1 acid (GABA(A)) receptor-associated protein-like 1 [Mus musculus]
LesAffx.61012.1.S1_at	BF050668	TC23271	194	358	448	1.85	2.31	Transcribed locus, weakly similar to NP_001042750.1 [Oryza sativa (japonica cultivar-group)]
Les.3623.1.S1_at	L12051.1	TC22145	888	1387	2048	1.56	2.31	GTPase
Les.2127.1.A1_at	BG631801	TC22627	179	282	412	1.58	2.3	Transcribed locus, weakly similar to NP_566133.1 protein [Arabidopsis thaliana]
LesAffx.62690.2.A1_at	CD003021	CD00302	210	392	483	1.87	2.3	Transcribed locus, weakly similar to NP_001043439.1 [Oryza sativa (japonica cultivar-group)]
Les.2938.3.A1_at	BG631240	TC21894	2639	5208	6069	1.97	2.3	Transcribed locus, weakly similar to NP_001063569.1 [Oryza sativa (japonica cultivar-group)]
Les.3408.1.S2_at	Y08804.1	TC21800	19	21	43	1.1	2.29	PR protein
Les.815.1.A1_at	BG627610	BG62761	55	99	125	1.81	2.29	Transcribed locus
LesAffx.61510.2.S1_at	BE433504	TC23546	264	448	604	1.7	2.29	Transcribed locus, weakly similar to NP_001058639.1 [Oryza sativa (japonica cultivar-group)]
LesAffx.64333.1.S1_at	CN384866	TC21750	2543	1844	5802	0.72	2.28	Transcribed locus, weakly similar to NP_565383.1 protein [Arabidopsis thaliana]
LesAffx.15850.2.A1_at	CK715684	TC23653	651	1130	1477	1.74	2.27	Transcribed locus, weakly similar to NP_001046323.1 [Oryza sativa (japonica cultivar-group)]
Les.1967.1.A1_at	BG630553	TC23879	64	58	146	0.9	2.27	Transcribed locus, weakly similar to NP_193738.1 (QUARTET 3) [Arabidopsis thaliana]
LesAffx.2226.2.A1_at	CK716250	TC23401	526	1027	1187	1.95	2.25	Transcribed locus, moderately similar to XP_001100144.1 similar to ribonucleotide reductase M2 B (TP53 inducible) isoform 3 [Macaca mulatta]
Les.2214.1.A1_at	BG735119	TC23773	287	516	646	1.8	2.25	Transcribed locus, moderately similar to NP_001067050.1 [Oryza sativa (japonica cultivar-group)]
Les.5939.1.S1_at	CN385618	TC23300	420	682	945	1.62	2.25	Transcribed locus, moderately similar to NP_001044456.1 [Oryza sativa (japonica cultivar-group)]
LesAffx.60369.1.S1_at	BG130091	TC22835	863	1185	1929	1.37	2.24	Transcribed locus, moderately similar to NP_001064827.1 [Oryza sativa (japonica cultivar-group)]
LesAffx.47467.1.S1_at	AI896278	TC22724	37	39	83	1.05	2.23	Transcribed locus, weakly similar to NP_001045094.1 [Oryza sativa (japonica cultivar-group)]
LesAffx.56868.1.S1_at	AW030921	TC21948	74	131	165	1.77	2.23	Transcribed locus, weakly similar to NP_001045103.1 [Oryza sativa (japonica cultivar-group)]
Les.150.1.S1_at	BT013117.1	AW65015	431	676	957	1.57	2.22	Lycopersicon esculentum clone 114407R, mRNA sequence
Les.4096.1.S1_at	AW220281	TC22846	298	473	660	1.59	2.22	Transcribed locus, weakly similar to NP_001062356.1 [Oryza sativa (japonica cultivar-group)]
Les.2458.1.S1_at	BG713780	TC24476	23	36	51	1.57	2.21	Transcribed locus
Les.4040.1.A1_at	X83420.1	AW09681	510	933	1128	1.83	2.21	Alu I-fragment

Les.1013.1.A1_at	BG628758	TC22465	132	215	291	1.63	2.21	Transcribed locus
LesAffx.62121.1.S1_at	AW220705	TC24448	679	1055	1494	1.55	2.2	Transcribed locus, weakly similar to NP_001039191.1 protein containing an Inosine-uridine preferring nucleoside hydrolase domain [Xenopus tropicalis]
Les.1431.1.A1_at	BG631562	TC23902	817	1560	1790	1.91	2.19	Transcribed locus, moderately similar to NP_001043710.1 [Oryza sativa (japonica cultivar-group)]
LesAffx.44382.1.S1_at	BI921407	GO37430	106	130	232	1.23	2.19	Transcribed locus, weakly similar to NP_001041735.1 [Oryza sativa (japonica cultivar-group)]
LesAffx.60369.2.S1_at	AI782767	TC23268	939	1277	2046	1.36	2.18	Transcribed locus, moderately similar to NP_001064827.1 [Oryza sativa (japonica cultivar-group)]
Les.5881.1.A1_at	CK716328	TC24065	108	150	234	1.39	2.18	Transcribed locus
Les.2565.2.S1_at	BE462384	TC23474	1185	1923	2574	1.62	2.17	Transcribed locus, weakly similar to XP_001087898.1 stromal cell-derived factor 2-like 1 [Macaca mulatta]
LesAffx.837.1.S1_at	AJ831935	TC22608	195	359	423	1.84	2.17	Transcribed locus, weakly similar to NP_001055192.1 [Oryza sativa (japonica cultivar-group)]
Les.4254.1.S1_at	BG631257	TC24002	2077	2639	4494	1.27	2.16	Transcribed locus, moderately similar to NP_001061342.1 [Oryza sativa (japonica cultivar-group)]
Les.122.1.S1_at	Z15141.1	TC22826	5843	11597	12612	1.98	2.16	chitinase
Les.1444.1.S1_at	AI777910	TC22510	1011	1888	2180	1.87	2.16	Transcribed locus, weakly similar to XP_001108613.1 similar to 60S acidic ribosomal protein P1 isoform 2 [Macaca mulatta]
LesAffx.17773.1.S1_at	BF113622	DB69166	1065	1204	2293	1.13	2.15	Transcribed locus, weakly similar to NP_001045544.1 [Oryza sativa (japonica cultivar-group)]
LesAffx.59536.1.S1_at	BI210459	TC22441	2687	4260	5749	1.59	2.14	Transcribed locus, weakly similar to NP_001047018.1 [Oryza sativa (japonica cultivar-group)]
LesAffx.59777.1.S1_at	AI780774	TC22665	3096	5898	6622	1.91	2.14	Transcribed locus
LesAffx.67725.1.S1_at	BF097383	BF09738	49	87	105	1.75	2.13	---
LesAffx.56.15.A1_at	CK715686	TC22818	1568	2829	3318	1.8	2.12	Transcribed locus, weakly similar to NP_001060451.1 [Oryza sativa (japonica cultivar-group)]
Les.300.1.S1_at	BF097631	TC22421	508	563	1074	1.11	2.12	Transcribed locus, weakly similar to NP_001042001.1 [Oryza sativa (japonica cultivar-group)]
LesAffx.52960.1.S1_at	BI207845	TC23617	320	515	676	1.61	2.11	---
Les.527.1.A1_at	BG626073	TC22131	167	299	351	1.8	2.11	Transcribed locus, weakly similar to XP_001106667.1 similar to ADP-ribosylation factor-like 10B [Macaca mulatta]
Les.4465.1.S1_x_at	L24068.1	TC24359	3	5	6	1.64	2.11	Lycopersicon esculentum (DB266) meloidogyne-induced giant cell protein mRNA, 3' end
LesAffx.35954.1.S1_at	AW092295	AW09229	220	305	464	1.38	2.1	---

Les.2513.1.S1_at	BI205120	TC22383	524	673	1100	1.29	2.1	Transcribed locus, weakly similar to NP_065615.1 acid (GABA(A)) receptor-associated protein-like 1 [Mus musculus]
LesAffx.31073.1.A1_at	AY125865	AY12586	10	14	22	1.34	2.1	---
LesAffx.65156.1.A1_at	CD003450	TC23971	20	37	42	1.81	2.1	Transcribed locus
Les.170.1.S1_at	AY026344.1	TC22471	258	314	542	1.21	2.1	non-symbiotic hemoglobin class 2
LesAffx.47666.1.S1_at	BE458190	TC22130	698	1141	1455	1.63	2.08	Transcribed locus, moderately similar to NP_001058156.1 [Oryza sativa (japonica cultivar-group)]
Les.3707.1.A1_at	AF022013.1	TC24298	32	24	66	0.76	2.08	IAA2 protein
Les.827.1.S1_at	BG626882	TC23903	1785	2041	3700	1.14	2.07	TBG6 protein
LesAffx.55662.1.S1_at	AW625299	TC24518	216	429	447	1.98	2.07	---
Les.1243.1.A1_at	BG630316	TC23603	647	1260	1334	1.95	2.06	Transcribed locus
Les.3732.1.S1_at	AF308936.1	NP28032	85	63	176	0.74	2.06	endo-beta-1,4-D-glucanase
Les.3539.1.S1_at	AF203481.1	TC21733	425	676	874	1.59	2.06	phosphoenolpyruvate carboxylase kinase
LesAffx.71544.1.S1_at	BI422043	TC21855	151	277	309	1.84	2.06	Transcribed locus, weakly similar to NP_001045150.1 [Oryza sativa (japonica cultivar-group)]
Les.3086.2.S1_at	CN385936	TC22850	3681	6955	7543	1.89	2.05	Phospholipase PLDa3
Les.2949.1.S1_at	AJ515747.1	TC21956	391	441	798	1.13	2.04	proliferating cell nuclear antigen
LesAffx.15850.2.S1_at	CK715684	TC23653	495	899	1007	1.82	2.03	Transcribed locus, weakly similar to NP_001046323.1 [Oryza sativa (japonica cultivar-group)]
Les.2631.2.S1_at	BI208990	TC23151	1144	1804	2324	1.58	2.03	CDNA, clone: FC04CC10, HTC in fruit
Les.5927.1.S1_at	CK715580	TC21943	452	884	918	1.95	2.03	CDNA, clone: FC22CB04, HTC in fruit
Les.2494.2.A1_at	BG626810	TC22933	2141	3599	4343	1.68	2.03	Transcribed locus, moderately similar to NP_001057280.1 [Oryza sativa (japonica cultivar-group)]
LesAffx.67017.1.S1_at	AW032581	TC22724	40	63	81	1.57	2.03	Transcribed locus, moderately similar to NP_001046101.1 [Oryza sativa (japonica cultivar-group)]
Les.5523.1.S1_at	BT014071.1	TC21750	875	1513	1768	1.73	2.02	Lycopersicon esculentum clone 133171F, mRNA sequence
LesAffx.31514.1.S1_at	BG127453	TC22656	1036	1547	2093	1.49	2.02	Transcribed locus, weakly similar to NP_001041856.1 [Oryza sativa (japonica cultivar-group)]
LesAffx.15986.1.S1_at	CK715617	TC23538	47	68	94	1.44	2.01	Transcribed locus, weakly similar to XP_001116334.1 myo-inositol oxygenase [Macaca mulatta]
LesAffx.60171.1.S1_at	BG134335	TC22657	548	859	1101	1.57	2.01	Transcribed locus, moderately similar to NP_001056054.1 [Oryza sativa (japonica cultivar-group)]
Les.2565.1.S1_at	AW441199	TC23474	2409	3801	4837	1.58	2.01	Transcribed locus, weakly similar to XP_001087898.1 stromal cell-derived factor 2-like 1 [Macaca mulatta]
Les.878.1.A1_at	BG627846	BG62784	5	4	9	0.96	2	Transcribed locus

Les.5804.1.S1_at	BT014601.1	TC22840	1996	3196	3989	1.6	2	CDNA, clone: FC20CF12, HTC in fruit /// Lycopersicon esculentum clone 134079R, mRNA sequence
Les.2494.1.S1_at	AJ785249	BI20405	1939	2991	3873	1.54	2	Transcribed locus, moderately similar to NP_001057280.1 [Oryza sativa (japonica cultivar-group)]
LesAffx.29757.2.S1_at	AI898522	TC23303	2141	3656	4277	1.71	2	Transcribed locus, moderately similar to NP_001062087.1 [Oryza sativa (japonica cultivar-group)]
Les.4920.1.S1_at	BT012902.1	TC23329	1485	2675	2964	1.8	2	Lycopersicon esculentum clone 114011F, mRNA sequence
LesAffx.35173.1.S1_at	AW625495	TC23403	459	876	916	1.91	2	---
Les.388.2.S1_at	BG130490	TC23958	1011	580	510	0.57	0.5	Transcribed locus, weakly similar to NP_001044141.1 [Oryza sativa (japonica cultivar-group)]
LesAffx.13481.1.S1_at	BG134320	TC22621	1613	2031	814	1.26	0.5	Transcribed locus, weakly similar to NP_001044052.1 [Oryza sativa (japonica cultivar-group)]
Les.1234.1.A1_at	BG630249	BG63024	1065	810	537	0.76	0.5	Transcribed locus, weakly similar to NP_199933.2 protein [Arabidopsis thaliana]
LesAffx.58849.1.S1_at	BG123587	TC21996	378	240	191	0.63	0.5	Transcribed locus, weakly similar to NP_001043235.1 [Oryza sativa (japonica cultivar-group)]
LesAffx.3394.1.S1_at	BT013543.1	TC23598	1737	1195	875	0.69	0.5	---
Les.631.2.S1_at	BF113057	TC23074	418	263	210	0.63	0.5	Transcribed locus, weakly similar to NP_001044672.1 [Oryza sativa (japonica cultivar-group)]
LesAffx.56.5.A1_a_at	BI205937	TC23902	2551	2181	1281	0.85	0.5	Transcribed locus, weakly similar to NP_004311.1 Ca++ transporting, fast twitch 1 isoform b [Homo sapiens]
LesAffx.37344.1.S1_at	AI897449	TC21881	185	150	93	0.81	0.5	Transcribed locus, weakly similar to NP_001042390.1 [Oryza sativa (japonica cultivar-group)]
Les.4791.1.S1_at	AI775838	TC23701	327	172	164	0.53	0.5	Transcribed locus, weakly similar to NP_001055100.1 [Oryza sativa (japonica cultivar-group)]
Les.5192.1.S1_at	BT013479.1	TC22963	180	105	90	0.58	0.5	Lycopersicon esculentum clone 132157F, mRNA sequence
LesAffx.64445.1.S1_at	BE450262	TC23904	1803	1069	902	0.59	0.5	Transcribed locus, weakly similar to NP_001049227.1 [Oryza sativa (japonica cultivar-group)]
Les.5370.1.S1_at	BT013789.1	BP90464	444	276	222	0.62	0.5	Lycopersicon esculentum clone 132683R, mRNA sequence
LesAffx.59231.1.S1_at	BM535046	BM53504	264	214	132	0.81	0.5	Transcribed locus, moderately similar to NP_001049763.1 [Oryza sativa (japonica cultivar-group)]
Les.2286.1.S1_at	BT013195.1	TC22726	856	518	428	0.61	0.5	Lycopersicon esculentum clone 134347F, mRNA sequence
Les.3273.1.S1_at	BG627786	TC24518	2274	1796	1136	0.79	0.5	proline rich protein
Les.2722.2.S1_at	BI210554	TC23496	1050	653	524	0.62	0.5	Transcribed locus, weakly similar to NP_001046415.1 [Oryza sativa (japonica cultivar-group)]
LesAffx.10497.2.S1_at	AI897776	TC24435	414	302	206	0.73	0.5	Transcribed locus, weakly similar to NP_001042618.1 [Oryza sativa (japonica cultivar-group)]

Les.1936.1.S1_at	BG626997	TC21753	797	1263	397	1.59	0.5	Lycopersicon esculentum clone 133556R, mRNA sequence
LesAffx.37438.1.A1_at	AI487130	TC23751	171	140	85	0.82	0.5	Transcribed locus, weakly similar to NP_001066441.1 [Oryza sativa (japonica cultivar-group)]
LesAffx.17157.1.S1_at	CK715404	CK71540	206	145	102	0.7	0.5	---
LesAffx.43670.1.S1_at	AW040873	TC22844	758	384	376	0.51	0.5	Transcribed locus, moderately similar to NP_001052293.1 [Oryza sativa (japonica cultivar-group)]
LesAffx.57251.1.S1_at	BI208760	TC22155	37	27	19	0.73	0.5	Transcribed locus, weakly similar to NP_001043814.1 [Oryza sativa (japonica cultivar-group)]
Les.1331.1.A1_at	BG630887	TC24042	95	65	47	0.69	0.5	Transcribed locus
Les.224.1.S1_at	AJ277561.1	TC21885	1162	766	574	0.66	0.49	gts1 protein
Les.3242.1.A1_at	BG627693	TC22682	75	50	37	0.67	0.49	Transcribed locus, weakly similar to XP_001114034.1 similar to Glyceraldehyde-3-phosphate dehydrogenase (GAPDH) isoform 1 [Macaca mulatta]
Les.2722.1.S1_at	AI899162	TC23496	1135	694	559	0.61	0.49	Transcribed locus, weakly similar to NP_001046415.1 [Oryza sativa (japonica cultivar-group)]
LesAffx.24121.2.A1_at	BI211077	TC23027	481	324	236	0.67	0.49	Transcribed locus, weakly similar to NP_001061808.1 [Oryza sativa (japonica cultivar-group)]
Les.2459.2.S1_at	AW945045	TC22764	2482	1627	1215	0.66	0.49	Transcribed locus, weakly similar to NP_036081.1 activated protein kinase 14 [Mus musculus]
Les.482.1.S1_at	BG630028	TC22380	2928	1680	1432	0.57	0.49	photosystem II 23 kDa protein
Les.5696.1.S1_at	BT014400.1	TC23901	1491	922	728	0.62	0.49	Lycopersicon esculentum clone 133705F, mRNA sequence
LesAffx.16710.2.A1_at	AI899221	TC23788	327	293	159	0.9	0.49	Transcribed locus, weakly similar to XP_001097418.1 aquaporin 4 isoform 5 [Macaca mulatta]
Les.4028.1.A1_at	BE354143	TC24194	39	37	19	0.94	0.49	Lycopersicon esculentum (DB#101) meloidogyne-induced giant cell protein mRNA, 3' end
Les.1989.1.A1_at	BG630761	TC24215	1974	1211	960	0.61	0.49	Transcribed locus
LesAffx.66014.1.S1_at	BI926363	TC24281	615	365	299	0.59	0.49	Transcribed locus, moderately similar to NP_001066466.1 [Oryza sativa (japonica cultivar-group)]
Les.3522.1.S1_at	AF071786.3	TC21714	4483	3476	2179	0.78	0.49	sucrose-phosphate synthase
LesAffx.60153.1.S1_at	BI423436	BG13472	179	140	87	0.78	0.49	Transcribed locus, moderately similar to NP_001064880.1 [Oryza sativa (japonica cultivar-group)]
Les.4300.2.S1_at	AW217635	TC21888	8333	4266	4048	0.51	0.49	Transcribed locus, weakly similar to NP_001043243.1 [Oryza sativa (japonica cultivar-group)]
LesAffx.56.14.S1_at	AJ784593	AW62621	193	107	94	0.55	0.49	Transcribed locus, weakly similar to NP_001047056.1 [Oryza sativa (japonica cultivar-group)]
Les.4362.1.A1_at	AY306154.1	TC22524	2529	1433	1224	0.57	0.48	euAP1 APETALA1-like MADS-box

LesAffx.61165.1.S1_at	BM409862	TC22968	990	660	478	0.67	0.48	Transcribed locus, weakly similar to NP_001041945.1 [Oryza sativa (japonica cultivar-group)]
LesAffx.52470.1.S1_at	BM412030	TC23981	1673	888	806	0.53	0.48	Transcribed locus, weakly similar to NP_001045184.1 [Oryza sativa (japonica cultivar-group)]
Les.3234.1.A1_at	BG735047	TC23423	2298	2199	1107	0.96	0.48	Metallocarboxypeptidase inhibitor
LesAffx.64540.1.S1_at	AW441832	TC22052	794	808	382	1.02	0.48	Transcribed locus, moderately similar to NP_001053805.1 [Oryza sativa (japonica cultivar-group)]
LesAffx.48314.2.A1_at	AI486366	TC21917	243	196	117	0.81	0.48	Transcribed locus, weakly similar to XP_001161712.1 ring finger protein 10 isoform 7 [Pan troglodytes]
LesAffx.69867.1.S1_at	BE450245	TC22913	848	591	408	0.7	0.48	Transcribed locus, weakly similar to NP_001043594.1 [Oryza sativa (japonica cultivar-group)]
LesAffx.6294.1.S1_at	BI210923	TC22443	1666	1468	797	0.88	0.48	Transcribed locus, weakly similar to NP_190908.1 protein [Arabidopsis thaliana]
LesAffx.69867.3.S1_at	AW035583	TC22913	992	708	475	0.71	0.48	Transcribed locus, weakly similar to NP_001043594.1 [Oryza sativa (japonica cultivar-group)]
Les.3261.1.A1_at	BG627816	TC23483	1362	710	651	0.52	0.48	Transcribed locus, moderately similar to NP_001050426.1 [Oryza sativa (japonica cultivar-group)]
Les.1724.1.S1_at	AI780669	TC22140	2412	2691	1153	1.12	0.48	Transcribed locus, weakly similar to NP_001049750.1 [Oryza sativa (japonica cultivar-group)]
LesAffx.17157.1.A1_at	CK715404	CK71540	156	105	75	0.67	0.48	---
Les.4039.1.A1_at	AF004881.1	NP00056	234	163	112	0.69	0.48	Lycopersicon esculentum resistance complex protein I2C-4 (I2C-4) possible pseudo mRNA sequence
Les.2608.1.S1_at	BT014160.1	TC23356	1160	767	551	0.66	0.48	Lycopersicon esculentum clone 133280F, mRNA sequence
LesAffx.46734.1.S1_at	AI485163	AI48516	329	218	156	0.66	0.47	Transcribed locus, weakly similar to NP_001045017.1 [Oryza sativa (japonica cultivar-group)]
LesAffx.27440.1.S1_at	BM410131	TC23837	663	346	315	0.52	0.47	Transcribed locus, weakly similar to NP_001055851.1 [Oryza sativa (japonica cultivar-group)]
LesAffx.9367.1.S1_at	CN385420	TC22317	448	398	212	0.89	0.47	Transcribed locus, weakly similar to NP_001049918.1 [Oryza sativa (japonica cultivar-group)]
Les.4099.1.S1_at	AY187634.1	TC21751	741	574	351	0.77	0.47	phosphoenolpyruvate carboxylase kinase 2
LesAffx.38145.1.A1_at	AI487713	BM40940	459	370	217	0.81	0.47	Transcribed locus, weakly similar to NP_001050432.1 [Oryza sativa (japonica cultivar-group)]
Les.3038.1.S1_at	AW094037	TC22677	1638	1037	775	0.63	0.47	Transcribed locus, weakly similar to NP_001059599.1 [Oryza sativa (japonica cultivar-group)]
Les.2605.1.A1_at	BG631278	TC23103	1129	651	534	0.58	0.47	Transcribed locus, weakly similar to NP_001042131.1 [Oryza sativa (japonica cultivar-group)]

Les.232.1.S1_at	AW649825	TC23518	715	533	338	0.75	0.47	Transcribed locus, weakly similar to NP_001048301.1 [Oryza sativa (japonica cultivar-group)]
Les.5576.1.A1_at	BT014182.1	TC23559	722	512	341	0.71	0.47	Lycopersicon esculentum clone 133347F, mRNA sequence
Les.3436.2.S1_a_at	CN385216	TC22872	3174	3739	1499	1.18	0.47	Transcribed locus
Les.5629.1.S1_at	BT014283.1	TC24074	3103	3350	1463	1.08	0.47	Lycopersicon esculentum clone 133517F, mRNA sequence
Les.2174.1.S1_at	CN384833	TC22746	110	64	52	0.58	0.47	Transcribed locus
LesAffx.66847.1.S1_at	AI778064	AI77806	95	79	44	0.83	0.47	---
LesAffx.52453.1.S1_at	BG135842	BI92407	573	303	269	0.53	0.47	Transcribed locus
Les.1514.1.S1_at	BG626292	TC23484	846	595	396	0.7	0.47	Lycopersicon esculentum clone 133815F, mRNA sequence
Les.840.1.A1_at	BG629718	TC24411	352	426	165	1.21	0.47	Transcribed locus
Les.4492.2.S1_at	CK714954	TC22356	2845	1666	1332	0.59	0.47	Transcribed locus, weakly similar to XP_001111095.1 aldolase B isoform 5 [Macaca mulatta]
Les.1379.1.A1_at	BG631211	TC21753	544	394	254	0.72	0.47	Transcribed locus
LesAffx.9260.1.S1_at	AI781857	DB68638	221	116	103	0.52	0.47	Transcribed locus, moderately similar to NP_001048773.1 [Oryza sativa (japonica cultivar-group)]
Les.3564.1.A1_at	X55684.1	TC24079	656	425	306	0.65	0.47	extensin (class I)
Les.4542.1.A1_at	BT014515.1	DB71533	346	213	161	0.61	0.47	Lycopersicon esculentum clone 133903R, mRNA sequence
LesAffx.70590.1.S1_at	AI775408	TC22762	131	109	61	0.84	0.47	Transcribed locus, moderately similar to NP_001044465.1 [Oryza sativa (japonica cultivar-group)]
Les.3072.1.S1_at	BG627918	TC23388	669	388	312	0.58	0.47	MRNA binding protein precursor
LesAffx.29801.1.S1_at	AI489456	TC23187	4669	5239	2174	1.12	0.47	Transcribed locus, weakly similar to NP_001042186.1 [Oryza sativa (japonica cultivar-group)]
Les.3958.1.A1_at	AF385366.1	TC23696	244	124	113	0.51	0.46	violaxanthin de-epoxidase
Les.3671.1.S1_at	M60166.1	TC23533	3646	1922	1690	0.53	0.46	H+-ATPase
Les.5350.1.A1_at	BT013752.1	TC22353	230	122	106	0.53	0.46	Lycopersicon esculentum clone 132615R, mRNA sequence
Les.2703.1.S1_at	BG735318	TC23293	2028	1076	939	0.53	0.46	Lycopersicon esculentum clone 132109R, mRNA sequence
LesAffx.12150.1.A1_at	BG643920	TC24476	238	275	110	1.15	0.46	---
Les.5780.1.A1_at	BT014552.1	TC22185	203	104	94	0.51	0.46	Lycopersicon esculentum clone 133974R, mRNA sequence
LesAffx.66600.1.A1_at	AW036163	TC23173	542	434	249	0.8	0.46	Transcribed locus, moderately similar to NP_001045167.1 [Oryza sativa (japonica cultivar-group)]
Les.375.1.A1_at	BG734771	TC22710	1333	723	612	0.54	0.46	Transcribed locus, weakly similar to NP_565660.1 protein [Arabidopsis thaliana]
LesAffx.3537.1.A1_at	AJ784726	TC24068	1323	945	606	0.71	0.46	---
LesAffx.57811.1.A1_at	AI488979	AI48897	307	165	141	0.54	0.46	---
Les.5468.1.A1_at	BT013978.1	BP88737	38	34	18	0.89	0.46	Lycopersicon esculentum clone 133023F, mRNA sequence
LesAffx.66270.1.S1_at	BM410146	TC23074	307	216	140	0.7	0.46	Transcribed locus, weakly similar to NP_065597.1 kinase C [Mus musculus]

Les.861.1.A1_at	BG627791	TC23388	167	113	76	0.68	0.46	Transcribed locus, moderately similar to NP_001065502.1 [Oryza sativa (japonica cultivar-group)]
Les.2478.1.S1_at	AI780323	TC21757	6008	4010	2746	0.67	0.46	Pathogenesis-related protein P2
Les.3564.1.S1_at	X55684.1	TC24079	167	109	76	0.65	0.46	extensin (class I)
Les.103.1.S1_at	AJ243451.1	TC22759	233	165	106	0.71	0.46	cyclin A1
LesAffx.59375.2.A1_at	CD002142	TC24048	1594	1146	727	0.72	0.46	Transcribed locus, weakly similar to NP_001042037.1 [Oryza sativa (japonica cultivar-group)]
LesAffx.59375.1.A1_at	AI485882	TC24048	966	758	439	0.78	0.45	Transcribed locus, weakly similar to NP_001042037.1 [Oryza sativa (japonica cultivar-group)]
Les.2959.1.S1_at	BG734632	TC22039	2168	1281	986	0.59	0.45	Lycopersicon esculentum clone 132361R, mRNA sequence
LesAffx.71003.1.S1_at	AI779846	TC22050	436	393	198	0.9	0.45	Transcribed locus, weakly similar to NP_001054553.1 [Oryza sativa (japonica cultivar-group)]
LesAffx.69164.1.S1_at	AW649318	TC22018	295	159	134	0.54	0.45	Transcribed locus, weakly similar to NP_001044532.1 [Oryza sativa (japonica cultivar-group)]
LesAffx.65689.1.A1_at	AI486980	TC22449	901	817	409	0.91	0.45	Transcribed locus, weakly similar to NP_001046053.1 [Oryza sativa (japonica cultivar-group)]
LesAffx.7.1.S1_at	AW931153	TC22173	906	504	410	0.56	0.45	Transcribed locus, weakly similar to XP_001094360.1 lamin B receptor isoform 2 [Macaca mulatta]
LesAffx.65428.1.S1_at	BM411427	TC22724	1819	1168	822	0.64	0.45	Transcribed locus, weakly similar to NP_001043901.1 [Oryza sativa (japonica cultivar-group)]
LesAffx.49201.1.S1_at	AW222231	TC24148	1289	774	580	0.6	0.45	Transcribed locus, weakly similar to XP_001100859.1 hect domain and RLD 3 [Macaca mulatta]
Les.2260.1.A1_at	AW621278	TC24093	2674	1512	1204	0.57	0.45	Transcribed locus, weakly similar to NP_001059672.1 [Oryza sativa (japonica cultivar-group)]
Les.5301.1.S1_at	BI204978	TC22541	2747	1863	1235	0.68	0.45	Lycopersicon esculentum clone 132490R, mRNA sequence
LesAffx.37764.1.S1_at	CK715143	CK71514	378	309	170	0.82	0.45	---
Les.3369.2.S1_at	AW934098	TC23328	1529	1021	686	0.67	0.45	Transcribed locus, weakly similar to XP_001096627.1 nuclear VCP-like isoform 4 [Macaca mulatta]
LesAffx.64583.1.S1_at	BM409701	TC22829	700	612	313	0.87	0.45	Transcribed locus, weakly similar to XP_573107.2 hypothetical protein [Rattus norvegicus]
Les.1912.1.A1_at	BG630127	TC23947	743	401	332	0.54	0.45	Transcribed locus, weakly similar to NP_001042378.1 [Oryza sativa (japonica cultivar-group)]
Les.2459.1.S1_at	AI896105	TC22540	1293	844	578	0.65	0.45	Transcribed locus, weakly similar to NP_036081.1 activated protein kinase 14 [Mus musculus]
Les.4428.2.S1_at	CK575032	TC21729	641	649	286	1.01	0.45	carbonic anhydrase

LesAffx.29801.1.A1_at	AI489456	TC23187	288	340	128	1.18	0.45	Transcribed locus, weakly similar to NP_001042186.1 [Oryza sativa (japonica cultivar-group)]
Les.224.1.A1_at	AJ277561.1	TC21885	703	472	313	0.67	0.45	gts1 protein
Les.3266.3.S1_at	BI935106	TC22577	1204	1102	535	0.92	0.44	Transcribed locus, moderately similar to NP_001047794.1 [Oryza sativa (japonica cultivar-group)]
LesAffx.38499.1.S1_at	AJ785244	TC23833	2287	1372	1017	0.6	0.44	Transcribed locus, moderately similar to NP_001044017.1 [Oryza sativa (japonica cultivar-group)]
LesAffx.65306.1.S1_at	AW621362	TC23246	4179	2225	1858	0.53	0.44	Transcribed locus, weakly similar to NP_001062371.1 [Oryza sativa (japonica cultivar-group)]
Les.3072.2.S1_at	BM411575	TC23388	541	304	241	0.56	0.44	MRNA binding protein precursor
Les.4428.1.S1_a_at	AW443158	DB69734	206	230	92	1.12	0.44	carbonic anhydrase
LesAffx.31022.1.S1_at	CK715372	TC23408	2312	1860	1026	0.8	0.44	Transcribed locus, weakly similar to NP_001053420.1 [Oryza sativa (japonica cultivar-group)]
LesAffx.11805.1.A1_at	CN385556	TC23159	176	133	78	0.75	0.44	Transcribed locus, weakly similar to NP_001041858.1 [Oryza sativa (japonica cultivar-group)]
Les.2299.1.A1_at	BG734604	TC23692	255	150	113	0.59	0.44	Transcribed locus, weakly similar to NP_001042472.1 [Oryza sativa (japonica cultivar-group)]
LesAffx.25097.1.S1_at	BI926331	TC22738	375	677	166	1.81	0.44	Transcribed locus, weakly similar to NP_194461.1 factor/ zinc ion binding [Arabidopsis thaliana]
LesAffx.48370.1.S1_at	AW929756	TC22420	118	90	52	0.76	0.44	Transcribed locus, moderately similar to NP_001050138.1 [Oryza sativa (japonica cultivar-group)]
Les.3544.1.S1_at	AW930004	TC21734	939	955	413	1.02	0.44	beta-carotene hydroxylase
Les.695.1.A1_at	BG626949	TC23503	321	173	141	0.54	0.44	Transcribed locus, weakly similar to NP_001042986.1 [Oryza sativa (japonica cultivar-group)]
LesAffx.68320.1.S1_at	BM535633	ES89343	1160	1032	509	0.89	0.44	Transcribed locus, weakly similar to NP_001065587.1 [Oryza sativa (japonica cultivar-group)]
Les.3072.3.S1_at	BG129667	TC23388	675	359	296	0.53	0.44	MRNA binding protein precursor
Les.5861.1.S1_at	BF050616	TC24194	1748	901	765	0.52	0.44	Lycopersicon esculentum clone 133886R, mRNA sequence
LesAffx.45315.4.S1_at	AW622607	TC23315	2815	2717	1231	0.97	0.44	Transcribed locus, weakly similar to XP_001105252.1 similar to NAD(P)H:quinone oxidoreductase type 3, polypeptide A2 [Macaca mulatta]
Les.4050.1.S1_at	L26982.1		409	211	179	0.52	0.44	Lycopersicon esculentum (DB173) mRNA fragment
Les.1603.1.A1_at	BG630194	TC23278	109	60	47	0.55	0.44	Transcribed locus, weakly similar to NP_001043493.1 [Oryza sativa (japonica cultivar-group)]
Les.2964.2.S1_at	BI923596	TC22145	266	232	116	0.87	0.44	Transcribed locus, weakly similar to NP_001042324.1 [Oryza sativa (japonica cultivar-group)]
LesAffx.17150.1.A1_at	AW039265	TC23237	244	142	106	0.58	0.43	Nitrite reductase

LesAffx.65757.1.S1_at	AW624713	TC21840	460	363	200	0.79	0.43	Transcribed locus, weakly similar to NP_001053836.1 [Oryza sativa (japonica cultivar-group)]
LesAffx.41333.2.S1_at	AW934014	TC23167	243	248	105	1.02	0.43	Transcribed locus, weakly similar to XP_001111662.1 protein phosphatase 1B isoform 6 [Macaca mulatta]
Les.4270.1.S1_at	AF112368.1	TC23183	3350	2288	1450	0.68	0.43	catalase 2
Les.4471.1.S1_at	AF514776.1	TC22151	1961	1121	848	0.57	0.43	dCK/dGK-like deoxyribonucleoside kinase
Les.2985.1.A1_at	BG626023	TC23280	413	222	179	0.54	0.43	Transcribed locus, weakly similar to NP_001051630.1 [Oryza sativa (japonica cultivar-group)]
LesAffx.67955.1.S1_at	BM409816	TC24493	2549	1462	1100	0.57	0.43	Transcribed locus, weakly similar to NP_001045017.1 [Oryza sativa (japonica cultivar-group)]
Les.266.1.S1_at	M84801.1	AI48785	96	54	41	0.56	0.43	prosystemin
Les.2121.1.A1_at	AY292201.1	TC22352	489	282	210	0.58	0.43	defense-signaling glycopeptide hormone precursor
Les.1097.1.A1_at	BG629366	TC22902	1250	1196	537	0.96	0.43	Transcribed locus, weakly similar to XP_001097818.1 similar to F46E10.1a [Macaca mulatta]
LesAffx.12783.1.A1_at	AI485230	AI48523	71	54	31	0.76	0.43	---
LesAffx.41703.1.A1_at	BG352060	TC23987	676	602	290	0.89	0.43	Transcribed locus, moderately similar to NP_001062462.1 [Oryza sativa (japonica cultivar-group)]
Les.1968.1.A1_at	BG628724	TC23891	648	527	277	0.81	0.43	Transcribed locus, weakly similar to NP_001065587.1 [Oryza sativa (japonica cultivar-group)]
Les.1966.1.A1_at	BG629907	TC23932	111	77	47	0.7	0.43	Transcribed locus
Les.2568.2.S1_at	BI207248	TC23413	1929	1255	822	0.65	0.43	Transcribed locus, moderately similar to NP_001048363.1 [Oryza sativa (japonica cultivar-group)]
Les.3644.1.S1_at	X86452.1	TC23849	1616	1012	687	0.63	0.42	lycopene beta-cyclase
Les.1248.2.A1_at	BT012749.1	TC21728	558	302	237	0.54	0.42	GDP-mannose pyrophosphorylase
Les.228.1.S1_a_at	CK714929	TC23208	5136	4416	2181	0.86	0.42	hypothetical LOC543672
LesAffx.67482.1.S1_at	BG123945	TC23975	197	177	84	0.9	0.42	---
LesAffx.66600.1.S1_at	AW040689	TC23173	1261	839	535	0.67	0.42	Transcribed locus, moderately similar to NP_001045167.1 [Oryza sativa (japonica cultivar-group)]
LesAffx.1276.1.S1_at	BG130074	TC23044	1826	1244	774	0.68	0.42	Transcribed locus, weakly similar to NP_001043284.1 [Oryza sativa (japonica cultivar-group)]
Les.184.1.S1_at	L46681.1	TC21791	5572	3117	2352	0.56	0.42	aspartic protease precursor
LesAffx.1276.2.S1_at	AI484083	TC23044	2643	1753	1114	0.66	0.42	Transcribed locus, weakly similar to NP_001043284.1 [Oryza sativa (japonica cultivar-group)]
Les.4083.1.S1_at	BG735070	TC22596	6281	4130	2647	0.66	0.42	Lycopersicon esculentum clone 132756F, mRNA sequence
Les.470.1.S1_at	BG134628	TC23506	59	48	25	0.81	0.42	Transcribed locus, weakly similar to NP_200423.2 protein [Arabidopsis thaliana]

LesAffx.3918.1.S1_at	BM410706	TC21958	1405	1617	584	1.15	0.42	Transcribed locus, weakly similar to NP_001047111.1 [Oryza sativa (japonica cultivar-group)]
LesAffx.26970.1.S1_at	BF050591	BF05059	271	161	112	0.59	0.41	---
Les.2946.2.S1_at	BG130169	TC22071	4786	4060	1981	0.85	0.41	CDNA, clone: FC16CC05, HTC in fruit
Les.3180.1.S1_at	AI775150	DB70250	249	149	103	0.6	0.41	Transcribed locus, weakly similar to NP_001048006.1 [Oryza sativa (japonica cultivar-group)]
Les.2886.1.A1_at	BG628514	TC24034	317	486	131	1.53	0.41	Transcribed locus, weakly similar to NP_001042068.1 [Oryza sativa (japonica cultivar-group)]
Les.2478.1.S1_a_at	AI780323	TC21757	8977	5974	3687	0.67	0.41	Pathogenesis-related protein P2
Les.3549.1.A1_at	AF096252.1	TC23183	278	188	114	0.68	0.41	ethylene-responsive catalase
Les.3477.1.S1_at	AF230198.2	TC23205	64	34	26	0.54	0.41	hypothetical LOC543641
LesAffx.23546.1.S1_at	BG126449	TC22663	6952	4299	2840	0.62	0.41	Transcribed locus, moderately similar to NP_001043331.1 [Oryza sativa (japonica cultivar-group)]
Les.3113.1.S1_at	BG131753	TC21889	294	204	120	0.69	0.41	Transcribed locus, weakly similar to XP_001101334.1 sterol-C4-methyl oxidase-like isoform 1 [Macaca mulatta]
Les.4428.1.S1_at	AW443158	DB69734	121	145	49	1.2	0.41	carbonic anhydrase
LesAffx.51226.1.S1_at	AI779132	TC21742	589	475	239	0.81	0.41	Transcribed locus, strongly similar to NP_054512.1 f [Nicotiana tabacum]
Les.376.1.S1_at	BG627516	TC24390	3865	2486	1566	0.64	0.41	ribulose-1,5-bisphosphate carboxylase, small subunit precursor
Les.3740.1.S1_at	X73986.1	TC24046	1543	1145	622	0.74	0.4	cathepsin D inhibitor protein
LesAffx.3537.1.S1_at	AJ784726	TC24068	293	229	118	0.78	0.4	---
Les.2861.1.S1_at	BM412879	TC23421	701	867	282	1.24	0.4	Transcribed locus, weakly similar to NP_201222.1 protein [Arabidopsis thaliana]
LesAffx.70112.1.S1_at	AW092445	TC22090	268	150	108	0.56	0.4	Transcribed locus, moderately similar to NP_001044346.1 [Oryza sativa (japonica cultivar-group)]
Les.3174.1.S1_at	BT012701.1	TC22568	446	449	179	1	0.4	Lycopersicon esculentum clone 113575F, mRNA sequence
Les.4727.1.S1_at	AI776392		2702	1999	1081	0.74	0.4	Lycopersicon esculentum clone 133381R, mRNA sequence
LesAffx.65428.2.A1_at	AW429084	TC22724	1202	806	480	0.67	0.4	Transcribed locus, weakly similar to NP_001043901.1 [Oryza sativa (japonica cultivar-group)]
Les.4050.1.A1_at	L26982.1		387	219	153	0.56	0.4	Lycopersicon esculentum (DB173) mRNA fragment
LesAffx.68945.1.S1_at	BM413364	TC23656	1041	955	412	0.92	0.4	Transcribed locus, weakly similar to NP_009952.2 ORF; Ycr023cp [Saccharomyces cerevisiae]
Les.228.1.S1_at	CK714929	TC23208	2987	2479	1178	0.83	0.39	hypothetical LOC543672
LesAffx.13481.2.S1_at	BI921546	TC22621	1481	1682	584	1.14	0.39	Transcribed locus, weakly similar to NP_001044052.1 [Oryza sativa (japonica cultivar-group)]
Les.1198.1.A1_at	BG630006	TC22356	88	45	35	0.51	0.39	Transcribed locus, weakly similar to XP_001111095.1 aldolase B isoform 5 [Macaca mulatta]

LesAffx.56.3.S1_at	BG132247	TC23902	1413	1056	556	0.75	0.39	Transcribed locus, weakly similar to NP_004311.1 Ca++ transporting, fast twitch 1 isoform b [Homo sapiens]
Les.3017.2.S1_at	AW738744	TC23031	1738	879	684	0.51	0.39	Transcribed locus, weakly similar to XP_001084411.1 aquaporin 1 [Macaca mulatta]
Les.3314.1.S1_at	BG626710	TC23439	4197	2609	1649	0.62	0.39	glycolate oxidase
Les.4849.1.S1_at	BT012755.1	TC21877	871	597	342	0.69	0.39	Lycopersicon esculentum clone 113699R, mRNA sequence
Les.5831.1.S1_at	BT013888.1	TC21723	3075	1867	1202	0.61	0.39	malate dehydrogenase
Les.2219.1.A1_at	BG735134	TC23582	799	405	311	0.51	0.39	Transcribed locus, weakly similar to NP_568624.1 protein [Arabidopsis thaliana]
Les.2633.1.A1_at	BG628982	TC22918	185	151	72	0.81	0.39	Transcribed locus, weakly similar to NP_001042177.1 [Oryza sativa (japonica cultivar-group)]
Les.2568.1.S1_at	BI922309	TC23413	1053	724	405	0.69	0.38	Transcribed locus, moderately similar to NP_001048363.1 [Oryza sativa (japonica cultivar-group)]
Les.2886.3.S1_at	AW038176	TC22563	86	128	33	1.49	0.38	Transcribed locus, weakly similar to NP_001042068.1 [Oryza sativa (japonica cultivar-group)]
Les.692.1.S1_at	BE434920	TC23136	1591	1406	610	0.88	0.38	Transcribed locus, weakly similar to NP_001042690.1 [Oryza sativa (japonica cultivar-group)]
Les.1596.1.A1_at	BG627098	TC23373	2181	2030	833	0.93	0.38	Transcribed locus, moderately similar to NP_001051136.1 [Oryza sativa (japonica cultivar-group)]
Les.4299.1.S1_at	BG625870	TC21761	6953	4545	2655	0.65	0.38	Lycopersicon esculentum clone 134170F, mRNA sequence
LesAffx.41703.1.S1_at	BG352060	TC23987	167	145	64	0.87	0.38	Transcribed locus, moderately similar to NP_001062462.1 [Oryza sativa (japonica cultivar-group)]
Les.3742.1.S1_at	AF146690.1	TC22347	1651	925	629	0.56	0.38	Pto-responsive gene 1 protein
Les.745.1.A1_at	BG627244	GT16450	925	556	351	0.6	0.38	Transcribed locus
Les.2038.1.A1_at	BG631023	TC23348	529	453	201	0.86	0.38	Transcribed locus, weakly similar to NP_199407.1 ester hydrolase/ hydrolase, acting on ester bonds [Arabidopsis thaliana]
LesAffx.54522.1.S1_at	BM411853	TC22000	2395	1286	906	0.54	0.38	Transcribed locus, weakly similar to XP_001101267.1 similar to myeloblastosis proto-oncogene product isoform 2 [Macaca mulatta]
Les.804.1.S1_at	AW094303	TC22945	2812	2154	1063	0.77	0.38	Transcribed locus
Les.608.1.S1_at	BG628276	TC21796	4264	2640	1608	0.62	0.38	Lycopersicon esculentum clone 133776F, mRNA sequence
LesAffx.38499.1.A1_at	BI209699	TC22324	464	334	175	0.72	0.38	Transcribed locus, moderately similar to NP_001044017.1 [Oryza sativa (japonica cultivar-group)]
Les.3073.1.S1_at	AJ785415	TC21760	221	135	83	0.61	0.38	Fruit-ripening protein
Les.3488.1.S1_at	AF348461.1	TC21724	1018	565	382	0.55	0.38	cryptochrome 1b
LesAffx.37344.2.S1_at	BG131634	TC21881	337	233	126	0.69	0.38	Transcribed locus, weakly similar to NP_001042390.1 [Oryza sativa (japonica cultivar-group)]
Les.3550.1.S1_at	AF096251.1	TC24195	308	277	116	0.9	0.37	ethylene-responsive heat shock protein cognate 70

Les.112.1.S1_at	BG734834	TC21719	3221	1757	1203	0.55	0.37	zeaxanthin epoxidase
Les.3648.1.S1_at	X95270.1	TC21719	259	171	97	0.66	0.37	subtilisin-like endoprotease
Les.3290.2.S1_at	AI778507	TC22209	24	13	9	0.56	0.37	Transcribed locus, weakly similar to XP_001093616.1 glutamic-pyruvate transaminase (alanine aminotransferase) isoform 1 [Macaca mulatta]
Les.2173.1.A1_at	BG631444	TC23409	250	250	92	1	0.37	Transcribed locus, weakly similar to NP_001044532.1 [Oryza sativa (japonica cultivar-group)]
Les.4085.1.S1_at	BE459897	TC23484	2268	1223	830	0.54	0.37	Lycopersicon esculentum (DB264) meloidogyne-induced giant cell protein mRNA, 3' end
Les.3119.1.A1_at	AW037265	TC23253	360	226	132	0.63	0.37	Transcribed locus, weakly similar to XP_001094505.1 proteasome 26S non-ATPase subunit 3 isoform 4 [Macaca mulatta]
LesAffx.63776.1.S1_at	BM408779	TC22982	215	155	79	0.72	0.37	Transcribed locus, weakly similar to NP_001057034.1 [Oryza sativa (japonica cultivar-group)]
Les.1997.2.S1_at	AI898214	TC23947	618	315	225	0.51	0.36	Transcribed locus, weakly similar to NP_001737.1 precursor [Homo sapiens]
Les.3552.1.A1_at	AF081021.1	TC24327	56	32	20	0.58	0.36	hypoxia-induced protein 242
LesAffx.34986.1.A1_at	CK715664	TC23304	545	371	198	0.68	0.36	Transcribed locus, weakly similar to NP_001048569.1 [Oryza sativa (japonica cultivar-group)]
LesAffx.5.1.S1_at	AW621769	TC22795	3317	1958	1206	0.59	0.36	Transcribed locus, weakly similar to XP_001071653.1 similar to Protein patched homolog 2 (PTC2) [Rattus norvegicus]
Les.3995.1.S1_at	AW443014	TC21771	882	557	318	0.63	0.36	methionine rich arabinogalactan
Les.3113.2.S1_at	AI780108	FS20183	254	170	91	0.67	0.36	Transcribed locus, weakly similar to XP_001101334.1 sterol-C4-methyl oxidase-like isoform 1 [Macaca mulatta]
Les.1655.1.A1_at	BG627771	BG62777	81	48	29	0.59	0.36	Transcribed locus
Les.3119.3.S1_at	AW092353	TC23253	391	266	139	0.68	0.36	Transcribed locus, weakly similar to XP_001094505.1 proteasome 26S non-ATPase subunit 3 isoform 4 [Macaca mulatta]
Les.3621.1.S1_at	K03290.1	BG63088	1947	1725	690	0.89	0.35	wound-induced proteinase inhibitor I prepropeptide
LesAffx.20929.1.S1_at	BI206813	TC23621	307	235	107	0.76	0.35	Transcribed locus, weakly similar to NP_001051650.1 [Oryza sativa (japonica cultivar-group)]
LesAffx.55337.1.S1_at	BM410261	TC22580	1547	1391	540	0.9	0.35	Transcribed locus, weakly similar to NP_001043235.1 [Oryza sativa (japonica cultivar-group)]
Les.2024.1.A1_at	BG631036	TC23275	977	532	341	0.54	0.35	Transcribed locus, weakly similar to XP_001105201.1 fructose-1,6-bisphosphatase 1 isoform 1 [Macaca mulatta]
Les.4844.1.S1_at	BI921923	TC23893	1434	826	501	0.58	0.35	Lycopersicon esculentum clone 113684R, mRNA sequence
LesAffx.18330.1.S1_at	BG129699	TC24342	534	335	186	0.63	0.35	Transcribed locus, weakly similar to NP_001046074.1 [Oryza sativa (japonica cultivar-group)]
Les.4493.1.S1_at	BG734807	TC22690	2033	1142	705	0.56	0.35	ADP/ATP translocator
LesAffx.67368.1.S1_at	BG126810	BG12681	164	89	57	0.54	0.35	---

Les.2888.1.S1_at	AI491181	TC22589	2368	1528	818	0.65	0.35	Lycopersicon esculentum clone 113533R, mRNA sequence
Les.614.1.A1_at	BG626522	BG62652	240	156	83	0.65	0.35	Transcribed locus
Les.110.1.S1_at	AF154423.1	TC22330	902	513	312	0.57	0.35	TBG5 protein
Les.3210.2.A1_a_at	AJ320060	TC22662	441	371	152	0.84	0.35	Transcribed locus
LesAffx.34276.2.A1_at	AJ784451	TC22379	208	116	72	0.56	0.35	Transcribed locus, weakly similar to NP_001043674.1 [Oryza sativa (japonica cultivar-group)]
Les.1923.1.S1_at	BG630193	TC23497	247	168	85	0.68	0.34	Transcribed locus, moderately similar to NP_001066340.1 [Oryza sativa (japonica cultivar-group)]
Les.971.1.A1_at	CK575003	TC22472	1600	1593	551	1	0.34	Transcribed locus, weakly similar to NP_001043235.1 [Oryza sativa (japonica cultivar-group)]
Les.3210.2.A1_at	AJ320060	TC22662	1152	990	395	0.86	0.34	Transcribed locus
LesAffx.55225.1.A1_at	AJ785322	TC23525	279	207	96	0.74	0.34	Transcribed locus, weakly similar to NP_001043082.1 [Oryza sativa (japonica cultivar-group)]
Les.4868.1.S1_at	BT012795.1	TC22912	98	66	33	0.67	0.34	Lycopersicon esculentum clone 113794R, mRNA sequence
Les.3297.3.S1_at	BM410357	TC22153	222	114	76	0.51	0.34	Transcribed locus, weakly similar to NP_001043493.1 [Oryza sativa (japonica cultivar-group)]
Les.5745.1.S1_at	BT014484.1	TC22393	462	268	157	0.58	0.34	Lycopersicon esculentum clone 133838F, mRNA sequence
Les.1832.1.A1_at	BG629451	TC23307	774	1188	262	1.53	0.34	Transcribed locus, moderately similar to NP_001062542.1 [Oryza sativa (japonica cultivar-group)]
Les.3119.2.S1_at	BI927792	TC23253	232	146	79	0.63	0.34	Transcribed locus, weakly similar to XP_001094505.1 proteasome 26S non-ATPase subunit 3 isoform 4 [Macaca mulatta]
Les.5017.1.S1_at	BT013110.1	TC23613	201	157	67	0.78	0.33	Lycopersicon esculentum clone 114397F, mRNA sequence
Les.3210.1.S1_at	AI483637	TC22662	819	687	272	0.84	0.33	Transcribed locus
LesAffx.344.6.S1_at	BI934296	TC23230	1093	1106	361	1.01	0.33	Transcribed locus, weakly similar to NP_001047408.1 [Oryza sativa (japonica cultivar-group)]
Les.2063.1.A1_at	BG631371	TC24473	54	37	18	0.69	0.33	Transcribed locus
Les.3180.2.S1_at	AI775197	TC22419	224	131	73	0.59	0.33	Transcribed locus, weakly similar to NP_001048006.1 [Oryza sativa (japonica cultivar-group)]
LesAffx.66331.1.S1_at	BI421673	TC21932	243	195	79	0.8	0.33	Transcribed locus, weakly similar to NP_001056463.1 [Oryza sativa (japonica cultivar-group)]
Les.3593.1.S1_at	BT012763.1	TC23407	1370	1253	448	0.91	0.33	TAS14 peptide (AA 1-130)
Les.490.1.A1_at	BG625891	TC22945	275	163	89	0.59	0.33	Transcribed locus, weakly similar to XP_001108145.1 similar to nucleotide binding protein-like [Macaca mulatta]
Les.3127.2.S1_at	AW091641	TC22823	2675	2117	866	0.79	0.32	Transcribed locus, weakly similar to XP_001096387.1 similar to serine hydroxymethyltransferase 1 (soluble) isoform 1 [Macaca mulatta]
Les.3527.1.S1_at	AY008278.1	TC21750	1988	1271	641	0.64	0.32	lipoxygenase

Les.2131.1.A1_at	BG627099	TC22249	2846	2985	913	1.05	0.32	Transcribed locus, weakly similar to NP_001050262.1 [Oryza sativa (japonica cultivar-group)]
LesAffx.11659.1.A1_at	BI422229	TC24331	1344	858	431	0.64	0.32	Transcribed locus, weakly similar to XP_001109382.1 fumarylacetoacetate hydrolase (fumarylacetoacetase) isoform 1 [Macaca mulatta]
LesAffx.71242.2.S1_at	AW093460	TC22502	389	209	125	0.54	0.32	Transcribed locus, weakly similar to NP_001051053.1 [Oryza sativa (japonica cultivar-group)]
LesAffx.69296.1.A1_at	AW092917	TC22654	533	371	169	0.7	0.32	Transcribed locus, weakly similar to NP_001045764.1 [Oryza sativa (japonica cultivar-group)]
LesAffx.65682.1.A1_at	AI489857	TC22844	1175	847	369	0.72	0.31	Transcribed locus, weakly similar to XP_215626.3 similar to recombination activating gene 1 gene activation [Rattus norvegicus]
LesAffx.21904.1.S1_at	BM536146	TC23302	353	209	111	0.59	0.31	Transcribed locus, weakly similar to NP_001048363.1 [Oryza sativa (japonica cultivar-group)]
LesAffx.37588.1.S1_at	BG124246	TC21902	72	59	22	0.82	0.31	Transcribed locus, weakly similar to NP_001046132.1 [Oryza sativa (japonica cultivar-group)]
Les.172.2.S1_a_at	BT014416.1	TC22899	3766	2380	1176	0.63	0.31	SPM1 protein
Les.3668.1.S1_at	U09026.1	TC21838	2019	1331	629	0.66	0.31	lipoxygenase
Les.5157.1.S1_at	BT013405.1	TC23882	2180	1562	676	0.72	0.31	Lycopersicon esculentum clone 135719R, mRNA sequence
Les.3127.1.S1_at	AW092318	TC22823	2787	2027	860	0.73	0.31	Transcribed locus, weakly similar to XP_001096387.1 similar to serine hydroxymethyltransferase 1 (soluble) isoform 1 [Macaca mulatta]
Les.986.1.S1_at	AI897984	TC22607	837	627	258	0.75	0.31	Mitochondrial malate dehydrogenase
LesAffx.34986.1.S1_at	CK715664	TC23304	257	173	79	0.67	0.31	Transcribed locus, weakly similar to NP_001048569.1 [Oryza sativa (japonica cultivar-group)]
LesAffx.58481.1.A1_at	BM535000	TC23994	385	304	116	0.79	0.3	Transcribed locus, weakly similar to NP_001062260.1 [Oryza sativa (japonica cultivar-group)]
LesAffx.34276.2.S1_at	AJ784451	TC22379	521	271	156	0.52	0.3	Transcribed locus, weakly similar to NP_001043674.1 [Oryza sativa (japonica cultivar-group)]
Les.4291.2.S1_at	AW649205	TC22682	3897	2121	1162	0.54	0.3	Transcribed locus, weakly similar to NP_001044127.1 [Oryza sativa (japonica cultivar-group)]
LesAffx.41477.1.S1_at	AI782352	TC22420	1454	889	430	0.61	0.3	Transcribed locus, weakly similar to NP_001041763.1 [Oryza sativa (japonica cultivar-group)]
LesAffx.16710.2.S1_at	AI899221	TC23788	742	616	219	0.83	0.3	Transcribed locus, weakly similar to XP_001097418.1 aquaporin 4 isoform 5 [Macaca mulatta]
Les.2964.3.A1_at	BG626300	TC23599	896	686	264	0.77	0.29	Transcribed locus, weakly similar to NP_001042324.1 [Oryza sativa (japonica cultivar-group)]
Les.3166.1.S1_at	AI775188	TC23957	126	103	37	0.82	0.29	Transcribed locus, weakly similar to NP_001047732.1 [Oryza sativa (japonica cultivar-group)]

Les.2809.1.S1_at	BT014540.1	TC24080	614	349	180	0.57	0.29	Lycopersicon esculentum clone 133955F, mRNA sequence
LesAffx.55337.2.S1_at	AW738358	TC22580	731	668	213	0.91	0.29	Transcribed locus, weakly similar to NP_001043235.1 [Oryza sativa (japonica cultivar-group)]
LesAffx.41477.2.S1_at	BI924534	TC22420	1432	817	416	0.57	0.29	Transcribed locus, weakly similar to NP_001041763.1 [Oryza sativa (japonica cultivar-group)]
Les.4291.1.S1_at	BI934076	TC22682	3187	1788	924	0.56	0.29	Transcribed locus, weakly similar to NP_001044127.1 [Oryza sativa (japonica cultivar-group)]
Les.2131.2.S1_at	BE433043	TC22249	3124	3416	901	1.09	0.29	Transcribed locus, weakly similar to NP_001050262.1 [Oryza sativa (japonica cultivar-group)]
LesAffx.69296.1.S1_at	AW092917	TC22654	1334	842	382	0.63	0.29	Transcribed locus, weakly similar to NP_001045764.1 [Oryza sativa (japonica cultivar-group)]
LesAffx.14110.2.A1_at	AW932597	TC23949	222	131	64	0.59	0.29	Transcribed locus, weakly similar to NP_001042660.1 [Oryza sativa (japonica cultivar-group)]
Les.672.1.S1_at	BM536270	TC22195	512	278	146	0.54	0.29	Transcribed locus, weakly similar to NP_001042227.1 [Oryza sativa (japonica cultivar-group)]
Les.3924.1.S1_at	BE433308	TC23702	4474	3740	1265	0.84	0.28	Hypothetical protein LOC778199
Les.2909.2.S1_at	BM413099	TC22288	3596	1936	1011	0.54	0.28	Transcribed locus, weakly similar to NP_001041799.1 [Oryza sativa (japonica cultivar-group)]
Les.233.1.S1_at	M17558.1	TC23200	2526	1387	694	0.55	0.27	chlorophyll a/b-binding protein precursor
Les.2457.1.A1_at	BG630077	TC22085	1431	1075	392	0.75	0.27	Transcribed locus
Les.1219.1.A1_at	BG630155	TC23499	149	127	41	0.85	0.27	Transcribed locus, weakly similar to NP_001047376.1 [Oryza sativa (japonica cultivar-group)]
LesAffx.21101.1.S1_at	BI924446	TC21935	171	95	46	0.55	0.27	Transcribed locus, weakly similar to NP_001047732.1 [Oryza sativa (japonica cultivar-group)]
Les.4975.1.S1_at	BT013032.1	TC23130	451	369	121	0.82	0.27	Lycopersicon esculentum clone 114280F, mRNA sequence
Les.3515.1.S1_at	AF242849.1	TC21891	561	480	150	0.86	0.27	wound-inducible carboxypeptidase
Les.2909.1.S1_at	AW223174	TC24474	2561	1319	678	0.51	0.26	Transcribed locus, weakly similar to NP_001041799.1 [Oryza sativa (japonica cultivar-group)]
Les.5017.1.A1_at	BT013110.1	TC23613	570	389	150	0.68	0.26	Lycopersicon esculentum clone 114397F, mRNA sequence
LesAffx.14110.2.S1_at	AW932597	TC23949	1580	852	412	0.54	0.26	Transcribed locus, weakly similar to NP_001042660.1 [Oryza sativa (japonica cultivar-group)]
Les.2971.2.A1_at	BG629234	TC23191	3454	3463	896	1	0.26	Wound-induced proteinase inhibitor I prepropeptide
Les.3085.1.S1_at	BM413323	TC22204	1611	1106	417	0.69	0.26	Transcribed locus, weakly similar to NP_001042186.1 [Oryza sativa (japonica cultivar-group)]
Les.147.1.S1_at	BG629070	TC22473	102	80	26	0.78	0.26	chlorophyll a/b-binding protein precursor
LesAffx.8708.1.S1_at	AW033860	AW03386	93	56	24	0.6	0.26	---

Les.4345.4.A1_x_at	BG631293	TC22725	1347	771	342	0.57	0.25	Pathogenesis-related protein P2
Les.3127.3.S1_at	AW932976	AW09372	2340	1740	594	0.74	0.25	Transcribed locus, weakly similar to XP_001096387.1 similar to serine hydroxymethyltransferase 1 (soluble) isoform 1 [Macaca mulatta]
Les.4345.4.A1_at	BG631293	TC22725	1524	926	385	0.61	0.25	Pathogenesis-related protein P2
Les.1157.1.A1_at	BG629717	BG62971	123	74	31	0.6	0.25	Transcribed locus
Les.3718.1.S1_at	AF384374.1	TC21922	2301	1673	580	0.73	0.25	allene oxide cyclase
Les.2934.2.S1_at	BG128445	TC22376	308	219	77	0.71	0.25	Transcribed locus, weakly similar to NP_001046132.1 [Oryza sativa (japonica cultivar-group)]
Les.3127.1.S1_a_at	AW092318	TC22823	2284	1582	569	0.69	0.25	Transcribed locus, weakly similar to XP_001096387.1 similar to serine hydroxymethyltransferase 1 (soluble) isoform 1 [Macaca mulatta]
Les.424.1.S1_at	BG628349	TC22847	1361	860	337	0.63	0.25	Transcribed locus, weakly similar to NP_001041744.1 [Oryza sativa (japonica cultivar-group)]
Les.3741.1.S1_at	AF146691.1	TC23195	1172	1401	290	1.19	0.25	Eli3 protein
Les.4488.1.S1_at	BG628643	TC22827	5559	4864	1342	0.87	0.24	threonine deaminase
Les.1478.1.S1_at	BG631500	TC23304	1156	590	278	0.51	0.24	Lycopersicon esculentum clone 114252R, mRNA sequence
Les.3217.2.S1_at	CK575028	TC24389	4666	4917	1113	1.05	0.24	Ribulose-1,5-bisphosphate carboxylase, small subunit precursor
Les.4345.3.S1_x_at	AI775226	TC22725	2728	1497	644	0.55	0.24	Pathogenesis-related protein P2
Les.424.2.S1_at	AI776305	TC22847	1341	812	315	0.61	0.24	Transcribed locus, weakly similar to NP_001041744.1 [Oryza sativa (japonica cultivar-group)]
Les.3266.2.S1_at	BI931445	TC23669	304	309	71	1.01	0.23	Transcribed locus, moderately similar to NP_001047794.1 [Oryza sativa (japonica cultivar-group)]
Les.4508.1.S1_s_at	AI780576	BG12438	1823	1211	424	0.66	0.23	Lycopersicon esculentum clone 135719R, mRNA sequence
Les.4988.1.S1_at	BT013056.1	TC22946	2355	2536	541	1.08	0.23	Lycopersicon esculentum clone 114312R, mRNA sequence
Les.4508.2.S1_s_at	AW623685	TC23882	2441	1618	553	0.66	0.23	Lycopersicon esculentum clone 135719R, mRNA sequence
Les.2964.1.A1_at	BG627802	TC23599	1328	770	294	0.58	0.22	Transcribed locus, weakly similar to NP_001042324.1 [Oryza sativa (japonica cultivar-group)]
Les.3286.1.S1_at	BT013249.1	TC23425	4004	4169	883	1.04	0.22	Lycopersicon esculentum inducible plastid-lipid associated protein (CHRD)
Les.810.1.S1_at	BG627575	TC22972	549	335	118	0.61	0.21	Lycopersicon esculentum clone 133744F, mRNA sequence
Les.4617.1.S1_at	BT014212.1	TC23312	1506	827	319	0.55	0.21	Lycopersicon esculentum clone 133389F, mRNA sequence
Les.2971.1.S1_at	AI781668	TC23191	1510	1327	314	0.88	0.21	Wound-induced proteinase inhibitor I prepropeptide
Les.3649.1.S1_at	AJ785484	TC23379	7426	4329	1500	0.58	0.2	chalcone synthase
LesAffx.37212.1.S1_at	AI491145	TC23069	732	462	147	0.63	0.2	---
Les.3741.1.A1_at	AF146691.1	TC23195	572	591	113	1.03	0.2	Eli3 protein
LesAffx.10650.1.S1_at	AW036686	TC23252	1690	1345	329	0.8	0.19	CDNA, clone: FC20BC08, HTC in fruit
LesAffx.71535.1.S1_at	AW040299	TC23102	218	172	42	0.79	0.19	Transcribed locus, weakly similar to NP_001049750.1 [Oryza sativa (japonica cultivar-group)]

Les.4345.1.S1_at	BG629758	TC23353	4517	2395	877	0.53	0.19	Lycopersicon esculentum clone 133385R, mRNA sequence
Les.327.1.S1_at	BT014378.1	TC22961	3480	2789	671	0.8	0.19	Lycopersicon esculentum clone 133667F, mRNA sequence
Les.4345.2.A1_x_at	AI781554	TC23670	2872	1495	552	0.52	0.19	Pathogenesis-related protein P2
Les.4345.2.A1_a_at	AI781554	TC23670	2345	1211	433	0.52	0.18	Lycopersicon esculentum clone 133385R, mRNA sequence
Les.3376.1.A1_at	BG630058	TC21732	2814	2048	501	0.73	0.18	CONSTANS interacting protein 1
Les.4181.1.A1_at	BG630015	TC22910	158	81	27	0.51	0.17	Transcribed locus
Les.4522.1.S1_at	AY497477.1	TC23773	946	741	160	0.78	0.17	xyloglucan endotransglucosylase-hydrolase XTH6
Les.3070.2.A1_at	BG629293	TC22028	3090	2455	511	0.79	0.17	Transcribed locus, weakly similar to NP_001042269.1 [Oryza sativa (japonica cultivar-group)]
Les.3070.1.S1_at	BG126941	TC22028	2095	1556	342	0.74	0.16	Transcribed locus, weakly similar to NP_001042269.1 [Oryza sativa (japonica cultivar-group)]
Les.4258.1.A1_at	BG626687	TC23571	880	489	143	0.56	0.16	Transcribed locus, weakly similar to XP_001104139.1 similar to aarF domain containing kinase 1 isoform 2 [Macaca mulatta]
Les.3376.2.S1_at	AW932459	TC21732	1306	828	199	0.63	0.15	CONSTANS interacting protein 1
LesAffx.37212.1.A1_at	AI491145	TC23069	203	124	30	0.61	0.15	---
Les.84.1.S1_at	U50151.1	TC21723	3457	3083	506	0.89	0.15	leucine aminopeptidase
Les.4281.2.S1_at	BI926015	DB69892	542	407	76	0.75	0.14	Transcribed locus, weakly similar to XP_001110787.1 ectonucleoside triphosphate diphosphohydrolase 3 [Macaca mulatta]
LesAffx.1.1.S1_at	AY656838.1	TC21732	2424	1274	332	0.53	0.14	arginase 2
Les.4923.1.S1_at	BT012912.1	TC22925	697	810	93	1.16	0.13	Lycopersicon esculentum clone 114030R, mRNA sequence
LesAffx.29797.1.S1_at	AJ784483	TC23412	409	213	54	0.52	0.13	Transcribed locus, weakly similar to NP_001042863.1 [Oryza sativa (japonica cultivar-group)]
Les.2504.1.A1_at	BG628687	TC23404	1244	808	164	0.65	0.13	Transcribed locus, weakly similar to NP_001043990.1 [Oryza sativa (japonica cultivar-group)]
LesAffx.10091.1.S1_at	AI487223	TC23911	478	346	63	0.72	0.13	Transcribed locus, weakly similar to NP_173851.1 [Arabidopsis thaliana]
LesAffx.56221.1.S1_at	AI894713	TC24290	159	130	21	0.82	0.13	Transcribed locus, weakly similar to NP_001042068.1 [Oryza sativa (japonica cultivar-group)]
Les.4281.3.S1_at	AI771846	TC21921	105	82	13	0.78	0.13	Transcribed locus, weakly similar to XP_001110787.1 ectonucleoside triphosphate diphosphohydrolase 3 [Macaca mulatta]
LesAffx.69609.1.S1_at	AW929283	TC23404	563	308	71	0.55	0.13	Transcribed locus, weakly similar to NP_001043990.1 [Oryza sativa (japonica cultivar-group)]
Les.4281.1.A1_at	BG626715	TC21921	943	681	118	0.72	0.12	Transcribed locus, weakly similar to XP_001110787.1 ectonucleoside triphosphate diphosphohydrolase 3 [Macaca mulatta]
Les.4258.3.S1_at	BG128812	TC23571	613	327	75	0.53	0.12	Transcribed locus, weakly similar to XP_001104139.1 similar to aarF domain containing kinase 1 isoform 2 [Macaca mulatta]
Les.1314.1.S1_at	BG630759	TC24293	364	291	44	0.8	0.12	Transcribed locus

LesAffx.4388.1.S1_at	BI928506	TC22582	1264	638	655	0.5	0.52	Transcribed locus, weakly similar to NP_001048149.1 [Oryza sativa (japonica cultivar-group)]
LesAffx.63209.1.S1_at	AW092854	AW09285	205	103	255	0.5	1.25	---
Les.822.1.A1_at	BG629497	BG73456	617	310	594	0.5	0.96	Transcribed locus
Les.2638.1.A1_at	BG628467	TC24303	456	229	600	0.5	1.32	Transcribed locus, weakly similar to NP_001053399.1 [Oryza sativa (japonica cultivar-group)]
LesAffx.42825.1.S1_at	BI924900	TC23069	583	293	326	0.5	0.56	Transcribed locus, weakly similar to NP_001046792.1 [Oryza sativa (japonica cultivar-group)]
Les.2024.2.S1_at	BG642995	AI48435	1271	638	371	0.5	0.29	Transcribed locus, weakly similar to XP_001105201.1 fructose-1,6-bisphosphatase 1 isoform 1 [Macaca mulatta]
Les.3530.1.S1_at	AF258808.1	TC21713	1907	957	1330	0.5	0.7	aldehyde oxidase
Les.4707.1.S1_at	BT012714.1	TC23557	429	215	1276	0.5	2.98	Lycopersicon esculentum clone 113596R, mRNA sequence
Les.1501.1.A1_at	BG626168	TC24040	7795	3906	3769	0.5	0.48	Transcribed locus, weakly similar to NP_172635.1 protein [Arabidopsis thaliana]
Les.3640.1.S1_at	X95098.1	TC21838	101	51	57	0.5	0.56	ammonium transporter
LesAffx.5781.2.S1_at	AI776965	TC23407	1429	716	957	0.5	0.67	Transcribed locus, weakly similar to XP_001063508.1 similar to emopamil binding related protein, delta8-delta7 sterol isomerase related protein [Rattus norvegicus]
Les.3473.1.S1_at	AF403444.1	TC21727	8983	4495	7541	0.5	0.84	beta-mannosidase enzyme
LesAffx.39.1.S1_at	CN385433	TC22335	458	229	1162	0.5	2.54	Transcribed locus, weakly similar to NP_001042342.1 [Oryza sativa (japonica cultivar-group)]
Les.3180.3.A1_at	BG734557	TC23323	841	421	266	0.5	0.32	Transcribed locus, weakly similar to NP_001048006.1 [Oryza sativa (japonica cultivar-group)]
Les.4276.1.S1_at	BG626765	TC24339	5706	2854	3317	0.5	0.58	Lycopersicon esculentum clone 134975R, mRNA sequence
Les.3299.2.A1_s_at	BG626957	TC21732	1995	995	231	0.5	0.12	arginase 2
Les.2723.2.S1_at	BI203749	TC22825	402	200	283	0.5	0.7	Transcribed locus, weakly similar to XP_001087943.1 similar to carboxypeptidase, vitellogenic-like isoform 4 [Macaca mulatta]
LesAffx.66705.1.S1_at	BI207021	TC23871	1575	785	1105	0.5	0.7	Transcribed locus, moderately similar to NP_197266.1 protein [Arabidopsis thaliana]
LesAffx.61653.1.S1_at	BE353914	TC23734	25	12	22	0.5	0.88	Transcribed locus, weakly similar to NP_001046281.1 [Oryza sativa (japonica cultivar-group)]
LesAffx.45439.1.S1_at	AI772978	TC23250	139	69	73	0.5	0.53	Transcribed locus, moderately similar to NP_568801.2 [Arabidopsis thaliana]
LesAffx.21585.1.S1_at	AW929168	TC21793	2682	1332	1642	0.5	0.61	Transcribed locus, moderately similar to NP_001042660.1 [Oryza sativa (japonica cultivar-group)]
LesAffx.18344.1.S1_at	AI484899	AI48489	147	73	76	0.5	0.52	---
LesAffx.3438.1.A1_at	CN385709	TC23123	1193	592	966	0.5	0.81	Transcribed locus, weakly similar to XP_001092746.1 similar to HTPAP protein [Macaca mulatta]
Les.5075.1.S1_at	BT013228.1	TC21844	937	465	552	0.5	0.59	Lycopersicon esculentum clone 134470F, mRNA sequence

LesAffx.52232.1.S1_at	BE450207	TC22642	643	319	759	0.5	1.18	Transcribed locus, moderately similar to NP_001044846.1 [Oryza sativa (japonica cultivar-group)]
LesAffx.5781.1.S1_at	BI204122	BI93464	1937	959	1243	0.5	0.64	Transcribed locus, weakly similar to XP_001063508.1 similar to emopamil binding related protein, delta8-delta7 sterol isomerase related protein [Rattus norvegicus]
Les.4703.1.S1_at	BT012803.1	TC23339	3749	1854	3020	0.49	0.81	Lycopersicon esculentum clone 113819F, mRNA sequence
LesAffx.66486.1.S1_at	BI209424	BI20942	1052	519	645	0.49	0.61	---
LesAffx.6338.1.A1_at	AW929824	TC23173	3083	1522	1692	0.49	0.55	Transcribed locus, weakly similar to NP_001042207.1 [Oryza sativa (japonica cultivar-group)]
Les.4060.1.S1_at	BT014090.1	TC24190	2597	1280	1693	0.49	0.65	Lycopersicon esculentum (DB217) meloidogyne-induced giant cell protein mRNA, 3' end
Les.753.1.A1_at	BG627285	BG62728	440	217	388	0.49	0.88	Transcribed locus
LesAffx.65478.1.S1_at	AW441264	TC22848	650	320	489	0.49	0.75	Transcribed locus, weakly similar to XP_001108618.1 similar to Mortality factor 4-like protein 1 (MORF-related gene 15 protein) (Transcription factor-like protein MRG15) (Testis-expressed gene 189 protein) isoform 2 [Macaca mulatta]
LesAffx.55861.1.S1_at	AW932065	TC22128	2785	1372	1975	0.49	0.71	Transcribed locus, weakly similar to NP_001042276.1 [Oryza sativa (japonica cultivar-group)]
Les.5579.1.S1_at	BT014190.1	TC23962	586	288	887	0.49	1.52	Lycopersicon esculentum clone 133367R, mRNA sequence
Les.3214.1.S1_at	BG631018	TC21991	784	386	344	0.49	0.44	Transcribed locus, strongly similar to NP_001064609.1 [Oryza sativa (japonica cultivar-group)]
Les.4492.3.S1_at	CK715138	TC22356	1322	650	502	0.49	0.38	Transcribed locus, weakly similar to XP_001111095.1 aldolase B isoform 5 [Macaca mulatta]
Les.4311.1.S1_at	AY269087.1	TC24251	3552	1746	2132	0.49	0.6	GAI-like protein
LesAffx.71442.2.S1_at	BG125039	TC22165	1098	539	722	0.49	0.66	Transcribed locus, moderately similar to NP_001043594.1 [Oryza sativa (japonica cultivar-group)]
Les.2110.1.A1_at	BG631670	TC23758	630	309	338	0.49	0.54	Transcribed locus, weakly similar to NP_001045247.1 [Oryza sativa (japonica cultivar-group)]
Les.1997.1.S1_at	BE354113	TC22008	986	484	429	0.49	0.44	Transcribed locus, weakly similar to NP_001737.1 precursor [Homo sapiens]
Les.2453.1.S1_at	BG735536	TC23082	500	246	543	0.49	1.09	Transcribed locus, weakly similar to NP_001050364.1 [Oryza sativa (japonica cultivar-group)]
Les.3290.3.S1_at	BI933219	BG12945	1200	589	436	0.49	0.36	Transcribed locus, weakly similar to XP_001093616.1 glutamic-pyruvate transaminase (alanine aminotransferase) isoform 1 [Macaca mulatta]
LesAffx.37636.1.S1_at	CN384813	EG55389	44	21	35	0.49	0.81	---
Les.5382.1.S1_at	BT013811.1	AI48888	2430	1192	1988	0.49	0.82	Lycopersicon esculentum clone 132732F, mRNA sequence
Les.4753.1.S1_at	BT013114.1	TC22177	1323	649	1047	0.49	0.79	Lycopersicon esculentum clone 114401R, mRNA sequence
Les.3297.2.S1_at	AI773077	TC23571	603	295	138	0.49	0.23	Transcribed locus, weakly similar to NP_001043493.1 [Oryza sativa (japonica cultivar-group)]

LesAffx.65695.1.A1_at	AA824728	TC23451	758	371	398	0.49	0.53	Transcribed locus, moderately similar to NP_001047304.1 [Oryza sativa (japonica cultivar-group)]
LesAffx.55043.1.S1_at	BI927121	TC22818	2049	1002	1296	0.49	0.63	Transcribed locus, moderately similar to NP_001047304.1 [Oryza sativa (japonica cultivar-group)]
Les.2327.1.A1_at	BG734815	TC24413	273	133	158	0.49	0.58	Transcribed locus
Les.3961.1.S1_at	AY157065.1	TC23373	1215	594	803	0.49	0.66	WRKY transcription factor IId-6
Les.3426.1.A1_at	BG628304	GO37605	965	472	1045	0.49	1.08	Transcribed locus
Les.5780.1.S1_at	BT014552.1	TC22185	1295	632	643	0.49	0.5	Lycopersicon esculentum clone 133974R, mRNA sequence
LesAffx.70441.2.S1_at	BF052016	TC22588	1577	769	965	0.49	0.61	Transcribed locus, weakly similar to XP_001057078.1 similar to Interleukin-1 receptor-associated kinase 1 (IRAK-1) (IRAK) (Pelle-like protein kinase) (mPLK) [Rattus norvegicus]
Les.107.1.S1_at	AJ243452.1	TC22276	248	121	175	0.49	0.71	cyclin A2
LesAffx.1689.1.S1_at	BE436059	TC24218	129	63	99	0.49	0.77	---
Les.5226.1.S1_at	BT013530.1		1008	488	562	0.48	0.56	Lycopersicon esculentum clone 132248F, mRNA sequence
Les.4412.1.A1_at	BG627732	TC23503	592	287	232	0.48	0.39	Transcribed locus, weakly similar to NP_001042986.1 [Oryza sativa (japonica cultivar-group)]
Les.4300.3.S1_at	BI932691	TC21888	7764	3759	3570	0.48	0.46	Transcribed locus, weakly similar to NP_001043243.1 [Oryza sativa (japonica cultivar-group)]
LesAffx.71000.1.S1_at	BF096685	TC23035	1178	568	774	0.48	0.66	Transcribed locus, moderately similar to NP_001067733.1 [Oryza sativa (japonica cultivar-group)]
Les.3962.1.A1_at	BG630092	TC24180	1285	619	817	0.48	0.64	WRKY transcription factor IId-3
Les.4953.1.S1_at	BT012976.1	TC23170	434	209	139	0.48	0.32	Lycopersicon esculentum clone 114187R, mRNA sequence
Les.3322.2.S1_at	AW091831	TC22662	794	382	262	0.48	0.33	Transcribed locus, moderately similar to NP_001047599.1 [Oryza sativa (japonica cultivar-group)]
LesAffx.16672.1.A1_at	AW625117	TC23550	216	104	108	0.48	0.5	Transcribed locus, weakly similar to NP_001055520.1 [Oryza sativa (japonica cultivar-group)]
Les.34.1.S1_at	AF050496.1	TC21713	497	238	189	0.48	0.38	Ca2+-ATPase
Les.5253.1.S1_at	BT013571.1	TC22485	1254	600	767	0.48	0.61	Lycopersicon esculentum clone 132309R, mRNA sequence
Les.3479.1.S1_at	AF347614.1	TC22134	1492	714	654	0.48	0.44	sulfate transporter 2
Les.1079.3.A1_at	BG626088	GO37592	61	29	30	0.48	0.48	Transcribed locus, weakly similar to NP_001043162.1 [Oryza sativa (japonica cultivar-group)]
Les.1163.1.A1_at	BG629779	TC24214	999	478	523	0.48	0.52	Transcribed locus, moderately similar to NP_191007.1 factor [Arabidopsis
LesAffx.53506.1.S1_at	BI206456	TC22555	330	158	366	0.48	1.11	Transcribed locus, weakly similar to XP_001113078.1 similar to protein tyrosine phosphatase-like (proline instead of catalytic arginine), member b [Macaca mulatta]
Les.842.2.S1_at	AI896617	TC23080	2442	1167	924	0.48	0.38	Transcribed locus, weakly similar to NP_001042177.1 [Oryza sativa (japonica cultivar-group)]

Les.1857.1.A1_at	BG629691	TC23786	47	22	35	0.48	0.75	Transcribed locus, weakly similar to NP_001060003.1 [Oryza sativa (japonica cultivar-group)]
Les.5864.1.S1_at	U20596.1	TC23921	5218	2482	1688	0.48	0.32	hypothetical LOC544002
LesAffx.67395.1.A1_at	AI490749	TC23607	292	139	154	0.47	0.53	Transcribed locus, weakly similar to NP_001055520.1 [Oryza sativa (japonica cultivar-group)]
Les.3290.1.S1_at	BG123573	TC22022	1043	495	393	0.47	0.38	Transcribed locus, weakly similar to XP_001093616.1 glutamic-pyruvate transaminase (alanine aminotransferase) isoform 1 [Macaca mulatta]
Les.928.1.S1_at	BG735170	TC22279	2159	1024	2526	0.47	1.17	Lycopersicon esculentum clone 132994F, mRNA sequence
Les.355.1.A1_at	BG627591	TC23931	663	315	310	0.47	0.47	Transcribed locus
LesAffx.286.2.S1_a_at	BG629460	BP89354	11575	5482	5507	0.47	0.48	similar to aquaporin
Les.5316.1.S1_at	BT013701.1	TC24245	808	382	474	0.47	0.59	Lycopersicon esculentum clone 132543F, mRNA sequence
Les.5271.1.S1_s_at	BT013613.1	TC23091	247	117	124	0.47	0.5	HD-ZIP protein
Les.2659.2.A1_at	BG626591	TC23900	298	141	279	0.47	0.94	Transcribed locus, weakly similar to NP_001053382.1 [Oryza sativa (japonica cultivar-group)]
Les.24.1.S1_at	AF191823.1	TC21735	201	95	30	0.47	0.15	alpha-galactosidase
LesAffx.30938.1.A1_at	AJ785088	TC22113	188	89	56	0.47	0.3	Transcribed locus, weakly similar to NP_001042177.1 [Oryza sativa (japonica cultivar-group)]
Les.5263.1.S1_at	BT013600.1	TC22756	143	68	89	0.47	0.62	Lycopersicon esculentum clone 132364F, mRNA sequence
LesAffx.16672.1.S1_at	AW625117	TC23550	870	411	594	0.47	0.68	Transcribed locus, weakly similar to NP_001055520.1 [Oryza sativa (japonica cultivar-group)]
Les.5198.1.A1_at	BT013491.1		154	73	99	0.47	0.64	Lycopersicon esculentum clone 132175F, mRNA sequence
Les.2924.1.S1_at	BT012789.1	TC23389	1642	773	1092	0.47	0.66	Lycopersicon esculentum clone 113783F, mRNA sequence
Les.744.1.S1_at	BT013096.1	TC23212	1489	700	1024	0.47	0.69	Lycopersicon esculentum clone 114362R, mRNA sequence
LesAffx.66165.1.S1_at	AW649540	TC22272	1121	525	604	0.47	0.54	Transcribed locus, weakly similar to XP_001105944.1 similar to WW-domain oxidoreductase [Macaca mulatta]
LesAffx.30937.1.S1_at	AJ785090		1951	914	997	0.47	0.51	---
Les.19.1.S1_at	BG626377	TC24377	3622	1692	1355	0.47	0.37	Transcribed locus, moderately similar to NP_001042358.1 [Oryza sativa (japonica cultivar-group)]
LesAffx.1959.2.S1_at	AW216683	TC23208	2286	1066	1287	0.47	0.56	Transcribed locus, weakly similar to XP_001120892.1 similar to CG14214-PA [Apis mellifera]
Les.1977.1.S1_at	BG630657	TC22750	2032	947	789	0.47	0.39	Transcribed locus, weakly similar to XP_001097638.1 similar to CG32699-PA [Macaca mulatta]
Les.4597.1.S1_at	BT012921.1		267	124	145	0.47	0.54	Lycopersicon esculentum clone 114084R, mRNA sequence
LesAffx.53517.1.S1_at	AI773309	TC23424	3300	1533	2177	0.46	0.66	Transcribed locus, weakly similar to NP_001042342.1 [Oryza sativa (japonica cultivar-group)]

LesAffx.26471.1.S1_at	BG126065	TC22883	947	440	718	0.46	0.76	Transcribed locus, weakly similar to NP_001054088.1 [Oryza sativa (japonica cultivar-group)]
Les.3748.1.A1_at	AB023388.1	TC22967	411	191	168	0.46	0.41	acid phosphatase
LesAffx.41816.1.S1_at	AW649474	TC22094	232	107	170	0.46	0.73	Transcribed locus, weakly similar to XP_001102886.1 hexosaminidase B isoform 1 [Macaca mulatta]
Les.4008.1.S1_a_at	BG630151	TC21741	6129	2843	4048	0.46	0.66	xyloglucan endotransglycosylase/hydrolase 16 protein
Les.5255.1.S1_at	BT013579.1	BP87874	1114	516	630	0.46	0.57	Lycopersicon esculentum clone 132324R, mRNA sequence
LesAffx.10346.1.S1_at	CK715067	TC22642	616	285	329	0.46	0.53	Transcribed locus, weakly similar to NP_001053447.1 [Oryza sativa (japonica cultivar-group)]
Les.109.1.S1_at	AF154424.1	TC22392	2994	1386	2765	0.46	0.92	TBG6 protein
Les.5114.1.S1_at	BT013305.1	TC24276	109	51	49	0.46	0.45	Lycopersicon esculentum clone 134950R, mRNA sequence
Les.2305.1.S1_at	AW622769	TC22654	2692	1243	1343	0.46	0.5	Transcribed locus, weakly similar to NP_001044221.1 [Oryza sativa (japonica cultivar-group)]
Les.1997.3.A1_at	BG630825	TC24117	806	372	242	0.46	0.3	Transcribed locus, weakly similar to NP_001737.1 precursor [Homo sapiens]
Les.3175.1.S1_at	BG626608	TC23958	3439	1585	1408	0.46	0.41	Lycopersicon esculentum clone 132976F, mRNA sequence
Les.3983.1.S1_at	U20591.1	TC23477	213	98	54	0.46	0.25	flower-specific gamma-thionin-like protein/acidic protein precursor
Les.5515.1.S1_at	BT014060.1		1807	831	1067	0.46	0.59	Lycopersicon esculentum clone 133158F, mRNA sequence
Les.5601.1.S1_at	BT014233.1	TC22079	472	217	156	0.46	0.33	Lycopersicon esculentum clone 133432F, mRNA sequence
Les.61.1.S1_at	Y10403.1	TC22152	1715	787	779	0.46	0.45	RNA-directed RNA polymerase
Les.3967.1.S1_at	AY157317.2	TC22150	1725	790	556	0.46	0.32	omega-3 fatty acid desaturase
LesAffx.65695.2.S1_at	AI484733	TC23451	1906	873	1205	0.46	0.63	Transcribed locus, moderately similar to NP_001047304.1 [Oryza sativa (japonica cultivar-group)]
Les.266.1.S1_a_at	M84801.1	AI48785	68	31	23	0.46	0.33	prosystemin
			35	16	31	0.46	0.87	---
LesAffx.69601.2.S1_at	AW429229	TC21962	356	163	244	0.46	0.69	Transcribed locus, weakly similar to NP_001048441.1 [Oryza sativa (japonica cultivar-group)]
Les.2415.2.S1_at	BE431455	TC23842	1478	674	396	0.46	0.27	Transcribed locus, moderately similar to NP_001061906.1 [Oryza sativa (japonica cultivar-group)]
LesAffx.65060.1.A1_at	CK716180	DV10448	1701	775	729	0.46	0.43	---
Les.4857.2.S1_at	BT013315.1	TC24509	1355	617	1157	0.46	0.85	Lycopersicon esculentum clone 113755R, mRNA sequence
Les.842.1.S1_at	BI931450	TC23080	2736	1246	881	0.46	0.32	Transcribed locus, weakly similar to NP_001042177.1 [Oryza sativa (japonica cultivar-group)]
Les.4520.1.S1_at	BT013422.1	GO37596	5428	2470	2743	0.46	0.51	Lycopersicon esculentum clone 132061R, mRNA sequence
LesAffx.64675.2.A1_at	AJ784544	TC21939	434	198	205	0.45	0.47	Transcribed locus, weakly similar to NP_001052406.1 [Oryza sativa (japonica cultivar-group)]
Les.5282.1.S1_at	BT013633.1	BE46052	203	92	114	0.45	0.56	Lycopersicon esculentum clone 132435R, mRNA sequence

Les.1784.2.A1_at	BG628995	TC24140	176	80	62	0.45	0.35	Transcribed locus, weakly similar to XP_001118335.1 similar to intestinal facilitative glucose transporter 7 [Macaca mulatta]
Les.5318.1.S1_at	BT013703.1	TC24128	3932	1779	1064	0.45	0.27	Lycopersicon esculentum clone 132545F, mRNA sequence
LesAffx.34276.1.S1_at	BE433400	TC22379	262	118	68	0.45	0.26	Transcribed locus, weakly similar to NP_001043674.1 [Oryza sativa (japonica cultivar-group)]
LesAffx.9975.1.A1_at	AI487405	AI48740	63	28	33	0.45	0.52	---
LesAffx.30807.1.S1_at	AW624957	AW62495	1172	529	1035	0.45	0.88	Transcribed locus, weakly similar to XP_001116629.1 similar to fatty acid desaturase 2 isoform 2 [Macaca mulatta]
LesAffx.69295.1.S1_at	BI933687	TC23040	243	109	51	0.45	0.21	Transcribed locus, weakly similar to NP_001045746.1 [Oryza sativa (japonica cultivar-group)]
Les.2933.1.S1_at	BT012764.1	TC23454	2013	907	502	0.45	0.25	Lycopersicon esculentum clone 113710R, mRNA sequence
Les.3198.1.S1_at	L13654.1	TC22193	49	22	30	0.45	0.62	Lycopersicon esculentum peroxidase (TPX1)
Les.2044.2.S1_at	AW443278	TC23957	2460	1107	1134	0.45	0.46	Transcribed locus, weakly similar to NP_001043713.1 [Oryza sativa (japonica cultivar-group)]
Les.3279.3.S1_at	BE450546	TC21783	1995	897	841	0.45	0.42	Transcribed locus, weakly similar to NP_001042478.1 [Oryza sativa (japonica cultivar-group)]
Les.5160.1.S1_at	BT013410.1	TC23771	886	399	788	0.45	0.89	Lycopersicon esculentum clone 132046F, mRNA sequence
LesAffx.54132.1.S1_at	BM535606	TC23181	984	442	602	0.45	0.61	Transcribed locus, weakly similar to XP_001110795.1 similar to WD and tetratricopeptide repeats 1 [Macaca mulatta]
LesAffx.70136.2.S1_at	AI899680	TC23538	488	219	102	0.45	0.21	Transcribed locus, weakly similar to NP_001044898.1 [Oryza sativa (japonica cultivar-group)]
LesAffx.51641.1.S1_at	BM535284	TC23056	116	52	60	0.45	0.52	---
LesAffx.65695.2.A1_at	AI484733	TC23451	418	188	202	0.45	0.48	Transcribed locus, moderately similar to NP_001047304.1 [Oryza sativa (japonica cultivar-group)]
Les.21.1.S1_at	AJ006377.1	TC23436	578	259	213	0.45	0.37	subtilisin-like protease
Les.3054.1.S1_at	AI773693	TC23429	2558	1146	395	0.45	0.15	Lycopersicon esculentum clone 135718R, mRNA sequence
LesAffx.3570.4.S1_at	AW624180	TC22612	1001	447	569	0.45	0.57	Ribosomal protein L2
Les.2415.3.A1_at	BG735370	TC23842	2115	943	527	0.45	0.25	Transcribed locus, moderately similar to NP_001061906.1 [Oryza sativa (japonica cultivar-group)]
LesAffx.56.6.S1_at	BM536076	TC23980	92	41	115	0.45	1.25	Transcribed locus, weakly similar to NP_004311.1 Ca++ transporting, fast twitch 1 isoform b [Homo sapiens]
LesAffx.32563.1.S1_at	AI778206	TC22260	695	309	633	0.45	0.91	Transcribed locus, weakly similar to NP_001053075.1 [Oryza sativa (japonica cultivar-group)]
Les.326.1.A1_at	BG630477	TC23669	847	377	995	0.45	1.17	Transcribed locus
LesAffx.52329.1.S1_at	BI927716	BI92771	475	211	200	0.44	0.42	---

LesAffx.31451.1.S1_at	BM409478	TC22107	234	104	108	0.44	0.46	Transcribed locus, weakly similar to NP_001048751.1 [Oryza sativa (japonica cultivar-group)]
Les.4334.2.S1_at	BI928683	TC22597	705	313	331	0.44	0.47	Transcribed locus, weakly similar to XP_001109838.1 ribulose-5-phosphate-3-epimerase isoform 5 [Macaca mulatta]
LesAffx.64675.1.S1_a_at	CN385903	TC21939	844	374	477	0.44	0.57	Transcribed locus, weakly similar to NP_001052406.1 [Oryza sativa (japonica cultivar-group)]
Les.842.2.S1_a_at	AI896617	TC23080	3161	1402	1023	0.44	0.32	Transcribed locus, weakly similar to NP_001042177.1 [Oryza sativa (japonica cultivar-group)]
LesAffx.50610.1.S1_at	AI775987	TC24497	380	168	176	0.44	0.46	---
Les.4825.1.S1_at	BT012681.1	TC22418	761	336	411	0.44	0.54	Lycopersicon esculentum clone 113528F, mRNA sequence
Les.3279.1.A1_at	BG734827	TC22261	1997	883	1043	0.44	0.52	Transcribed locus, weakly similar to NP_001042478.1 [Oryza sativa (japonica cultivar-group)]
LesAffx.69601.1.S1_at	AW030431	TC21962	315	139	205	0.44	0.65	Transcribed locus, weakly similar to NP_001048441.1 [Oryza sativa (japonica cultivar-group)]
Les.3297.1.S1_at	BG630303	TC21776	2131	941	464	0.44	0.22	Transcribed locus, weakly similar to NP_001043493.1 [Oryza sativa (japonica cultivar-group)]
LesAffx.10346.1.A1_at	CK715067	TC22642	1573	694	978	0.44	0.62	Transcribed locus, weakly similar to NP_001053447.1 [Oryza sativa (japonica cultivar-group)]
Les.4982.1.S1_at	BT013044.1	AW62120	132	58	56	0.44	0.42	Lycopersicon esculentum clone 114294R, mRNA sequence
Les.2068.1.A1_at	BG631401	TC24040	513	224	298	0.44	0.58	Transcribed locus, weakly similar to NP_001045667.1 [Oryza sativa (japonica cultivar-group)]
Les.1501.2.S1_at	BM412826	TC24040	5568	2435	2301	0.44	0.41	Transcribed locus, weakly similar to NP_172635.1 protein [Arabidopsis thaliana]
Les.2261.1.A1_at	BG735361	BG73536	95	41	51	0.44	0.54	Transcribed locus
Les.3330.1.S1_at	AW650121	TC24355	850	371	391	0.44	0.46	Transcribed locus, weakly similar to NP_001044764.1 [Oryza sativa (japonica cultivar-group)]
LesAffx.24799.1.S1_at	CK715756	TC23394	295	129	201	0.44	0.68	Transcribed locus, weakly similar to NP_001045764.1 [Oryza sativa (japonica cultivar-group)]
LesAffx.46815.1.S1_at	BI934723	TC21911	398	174	202	0.44	0.51	Hypothetical LOC544002
LesAffx.47142.1.S1_at	BF114155	TC22906	57	25	29	0.44	0.5	Transcribed locus, weakly similar to NP_001042988.1 [Oryza sativa (japonica cultivar-group)]
Les.4563.1.S1_at	BT014344.1	TC23345	430	187	233	0.43	0.54	Lycopersicon esculentum clone 133619F, mRNA sequence
LesAffx.66879.1.A1_at	BG631312	TC22769	891	386	696	0.43	0.78	CDNA, clone: FC04CE09, HTC in fruit
Les.2917.1.S1_at	BM412347	TC22799	464	201	152	0.43	0.33	Transcribed locus
Les.3368.1.S1_at	BT014303.1	TC22868	2553	1105	1358	0.43	0.53	Lycopersicon esculentum clone 133554R, mRNA sequence
Les.1562.1.S1_at	BG629310	TC23006	316	137	119	0.43	0.38	Lycopersicon esculentum clone 133516F, mRNA sequence
Les.4549.1.S1_at	BT013085.1	TC24365	101	44	41	0.43	0.4	Lycopersicon esculentum clone 114349R, mRNA sequence

LesAffx.24799.2.A1_at	BI209670	TC24255	217	94	144	0.43	0.67	Transcribed locus, weakly similar to NP_001045764.1 [Oryza sativa (japonica cultivar-group)]
Les.4334.1.A1_at	BG626518	TC24039	1164	502	553	0.43	0.48	Transcribed locus, weakly similar to XP_001109838.1 ribulose-5-phosphate-3-epimerase isoform 5 [Macaca mulatta]
Les.4899.1.S1_a_at	BT012863.1		22	9	12	0.43	0.54	Lycopersicon esculentum clone 113951F, mRNA sequence
Les.1808.1.A1_at	BG629204		9	4	5	0.43	0.56	Transcribed locus
LesAffx.9007.1.S1_at	CN385923	GO37540	1335	573	830	0.43	0.62	Transcribed locus, weakly similar to NP_001042158.1 [Oryza sativa (japonica cultivar-group)]
Les.4529.1.S1_at	AY497478.1	TC21903	336	144	89	0.43	0.26	xyloglucan endotransglucosylase-hydrolase XTH7
Les.4880.1.S1_at	BT012820.1	TC24340	541	231	533	0.43	0.99	Lycopersicon esculentum clone 113867R, mRNA sequence
Les.799.1.A1_at	BG627521	BG62752	1066	456	270	0.43	0.25	Transcribed locus, weakly similar to NP_001052094.1 [Oryza sativa (japonica cultivar-group)]
Les.5221.1.S1_at	BT013523.1	TC24473	511	218	398	0.43	0.78	Lycopersicon esculentum clone 132241R, mRNA sequence
Les.1079.1.S1_at	BI931317	TC21949	119	51	59	0.43	0.5	Transcribed locus, weakly similar to NP_001043162.1 [Oryza sativa (japonica cultivar-group)]
Les.4615.1.S1_at	BE462687	TC22537	3031	1293	3045	0.43	1	Lycopersicon esculentum clone 113657R, mRNA sequence
LesAffx.7472.2.S1_at	AW651303	TC23954	119	51	42	0.43	0.35	Transcribed locus, weakly similar to NP_001043730.1 [Oryza sativa (japonica cultivar-group)]
LesAffx.24696.1.S1_at	AW034874	DB70637	120	51	162	0.43	1.35	Transcribed locus, weakly similar to NP_001043378.1 [Oryza sativa (japonica cultivar-group)]
Les.3819.1.S1_at	AY140893.1	TC21842	1121	477	1073	0.43	0.96	ovate protein
LesAffx.55463.1.S1_at	AW651085	TC23651	83	35	68	0.43	0.81	---
Les.1977.3.S1_at	BM410747	TC23608	1239	526	419	0.42	0.34	Transcribed locus, weakly similar to XP_001097638.1 similar to CG32699-PA [Macaca mulatta]
LesAffx.58110.1.A1_at	AJ785349	BE43527	265	112	94	0.42	0.36	---
Les.3011.1.S1_at	BT012732.1	TC23275	1382	586	799	0.42	0.58	Light dependent NADH:protochlorophyllide oxidoreductase 2
LesAffx.67395.1.S1_at	AI490749	TC23607	987	418	583	0.42	0.59	Transcribed locus, weakly similar to NP_001055520.1 [Oryza sativa (japonica cultivar-group)]
Les.2122.1.A1_at	BG631753	TC22836	14	6	27	0.42	2	Transcribed locus
LesAffx.65060.1.S1_at	CK716180	DV10448	953	402	299	0.42	0.31	---
Les.1310.1.S1_at	BT013430.1	TC22042	2741	1155	787	0.42	0.29	Lycopersicon esculentum clone 132070F, mRNA sequence
Les.5610.1.S1_at	BT014251.1	TC22162	3516	1481	3012	0.42	0.86	Lycopersicon esculentum clone 133453R, mRNA sequence
LesAffx.46815.2.S1_at	BI929852	TC22829	1145	482	605	0.42	0.53	Hypothetical LOC544002
Les.5865.1.S1_at	BT013275.1	TC23540	1276	537	1964	0.42	1.54	Lycopersicon esculentum clone 134853R, mRNA sequence
Les.67.1.S1_at	U49330.1	TC21724	6290	2639	4581	0.42	0.73	pectin methylesterase

LesAffx.26471.2.S1_at	AW218197	TC22883	46	19	44	0.42	0.96	Transcribed locus, weakly similar to NP_001054088.1 [Oryza sativa (japonica cultivar-group)]
Les.5786.1.S1_at	BT014563.1	TC22614	2066	866	1024	0.42	0.5	Lycopersicon esculentum clone 134017R, mRNA sequence
LesAffx.33890.1.S1_at	BG134582	TC22584	60	25	50	0.42	0.83	Transcribed locus, weakly similar to NP_001067602.1 [Oryza sativa (japonica cultivar-group)]
LesAffx.54718.1.S1_at	BM411779	TC22617	331	138	167	0.42	0.5	Transcribed locus, moderately similar to NP_001044227.1 [Oryza sativa (japonica cultivar-group)]
Les.2012.1.S1_at	BG627650	TC23373	196	82	46	0.42	0.24	Myo-inositol-1-phosphate synthase
LesAffx.71563.1.A1_at	AI487345	TC22892	310	129	122	0.42	0.39	---
Les.1940.1.A1_at	BG630347	TC22797	803	333	427	0.41	0.53	Transcribed locus, weakly similar to NP_001046378.1 [Oryza sativa (japonica cultivar-group)]
Les.1179.1.A1_at	BG629888	TC24291	241	100	180	0.41	0.75	Transcribed locus
LesAffx.39072.1.S1_at	BI211118	TC22430	1594	661	918	0.41	0.58	Transcribed locus, weakly similar to NP_172552.2 protein [Arabidopsis thaliana]
Les.5198.1.S1_at	BT013491.1		448	186	367	0.41	0.82	Lycopersicon esculentum clone 132175F, mRNA sequence
Les.3748.1.S1_at	AB023388.1	TC22967	1360	563	668	0.41	0.49	acid phosphatase
Les.3094.3.S1_at	BG125458	TC23596	1412	583	420	0.41	0.3	Transcribed locus, weakly similar to NP_001041542.1 P450, family 51, subfamily A, polypeptide 1 [Gallus gallus]
Les.3733.1.S1_at	AF096776.1	TC21836	791	326	599	0.41	0.76	expansin
Les.3771.1.S1_at	Y14387.1	TC22876	191	78	119	0.41	0.62	lycopene epsilon-cyclase
Les.2529.2.S1_at	BI421300	TC22334	624	257	481	0.41	0.77	Transcribed locus, weakly similar to NP_001041768.1 [Oryza sativa (japonica cultivar-group)]
Les.206.1.S1_at	AW625821	TC23716	1292	530	1725	0.41	1.34	Transcribed locus, moderately similar to NP_001046132.1 [Oryza sativa (japonica cultivar-group)]
Les.3034.1.S1_at	BG631511	TC22683	1900	779	1399	0.41	0.74	Lycopersicon esculentum clone 114419R, mRNA sequence
Les.5223.1.S1_at	BT013527.1	TC23738	1467	600	1178	0.41	0.8	Lycopersicon esculentum clone 132245R, mRNA sequence
LesAffx.68607.1.S1_at	BM409831	TC23658	1132	461	564	0.41	0.5	Transcribed locus, weakly similar to NP_001049586.1 [Oryza sativa (japonica cultivar-group)]
Les.4097.1.A1_at	AF022018.1	TC22516	568	231	202	0.41	0.36	IAA7 protein
LesAffx.56.16.A1_at	BG643188	AI89840	359	146	174	0.41	0.48	Transcribed locus, moderately similar to NP_001059779.1 [Oryza sativa (japonica cultivar-group)]
Les.3214.2.S1_at	BF050423	TC21991	587	239	191	0.41	0.32	Transcribed locus, strongly similar to NP_001064609.1 [Oryza sativa (japonica cultivar-group)]
Les.4089.1.S1_at	BG642986	TC22862	870	353	626	0.41	0.72	Transcribed locus
LesAffx.30937.1.A1_at	AJ785090		575	234	191	0.41	0.33	---
Les.2168.1.S1_at	BT013274.1	TC23964	2082	846	344	0.41	0.17	Lycopersicon esculentum clone 134851R, mRNA sequence
Les.3016.1.S1_at	BT012757.1	TC24030	815	330	122	0.4	0.15	Lycopersicon esculentum clone 113701F, mRNA sequence

Les.3216.3.S1_at	AW649713	TC22776	491	198	229	0.4	0.47	Transcribed locus, moderately similar to NP_001043347.1 [Oryza sativa (japonica cultivar-group)]
Les.4097.1.S1_at	AF022018.1	TC22516	1611	650	655	0.4	0.41	IAA7 protein
Les.4564.1.S1_at	BT013856.1	TC21760	2870	1155	1916	0.4	0.67	Lycopersicon esculentum clone 132816F, mRNA sequence
LesAffx.65678.1.S1_at	AW442323	AW22314	727	292	296	0.4	0.41	Transcribed locus, weakly similar to XP_001091707.1 interleukin-1 receptor-associated kinase 4 [Macaca mulatta]
Les.5791.1.S1_at	BT014572.1	TC22816	794	319	373	0.4	0.47	Lycopersicon esculentum clone 134027R, mRNA sequence
Les.3247.1.S1_at	BT013622.1	TC22319	2639	1057	1295	0.4	0.49	Lycopersicon esculentum clone 132423F, mRNA sequence
Les.3636.1.S1_at	Y11268.1	TC23558	357	143	388	0.4	1.09	endo-1,4-beta-D-glucanase
LesAffx.24799.2.S1_at	BI209670	TC24255	472	189	327	0.4	0.69	Transcribed locus, weakly similar to NP_001045764.1 [Oryza sativa (japonica cultivar-group)]
LesAffx.30938.1.S1_at	AJ785088	TC22113	391	156	127	0.4	0.32	Transcribed locus, weakly similar to NP_001042177.1 [Oryza sativa (japonica cultivar-group)]
Les.3019.1.S1_at	BI926148	TC23144	415	165	199	0.4	0.48	Transcribed locus, weakly similar to NP_065956.1 dehydrogenase 14 (all-trans and 9-cis) [Homo sapiens]
Les.5214.1.S1_at	BT013515.1	DB71633	1065	423	648	0.4	0.61	Lycopersicon esculentum clone 132232R, mRNA sequence
Les.3279.2.S1_at	BI933014	TC21783	2940	1166	1240	0.4	0.42	Transcribed locus, weakly similar to NP_001042478.1 [Oryza sativa (japonica cultivar-group)]
Les.5670.1.S1_at	BT014359.1	TC22878	185	73	152	0.4	0.82	Lycopersicon esculentum clone 133637R, mRNA sequence
LesAffx.17924.1.S1_at	BG124217	TC24101	891	353	435	0.4	0.49	Transcribed locus, weakly similar to NP_001049241.1 [Oryza sativa (japonica cultivar-group)]
LesAffx.48876.1.S1_at	AW622727	AW62272	48	19	43	0.4	0.89	---
LesAffx.64206.1.S1_at	AW034232	TC21738	2480	981	1230	0.4	0.5	Transcribed locus, weakly similar to NP_001041979.1 [Oryza sativa (japonica cultivar-group)]
Les.790.1.A1_at	BG627458	TC23127	413	163	120	0.4	0.29	Transcribed locus, weakly similar to NP_001052896.1 [Oryza sativa (japonica cultivar-group)]
LesAffx.56237.1.S1_at	AW218110	TC22223	213	84	214	0.39	1.01	Transcribed locus, weakly similar to NP_001041856.1 [Oryza sativa (japonica cultivar-group)]
Les.394.1.A1_at	BG627056	TC23820	698	275	281	0.39	0.4	Transcribed locus
Les.720.1.A1_at	BG631868	TC24279	2556	1005	994	0.39	0.39	Transcribed locus, weakly similar to NP_001042731.1 [Oryza sativa (japonica cultivar-group)]
LesAffx.58110.1.S1_at	BE435276	BE43527	1191	469	422	0.39	0.35	---
Les.2923.1.S1_at	BI925625	TC23094	4746	1865	2978	0.39	0.63	Transcribed locus, weakly similar to NP_001044052.1 [Oryza sativa (japonica cultivar-group)]
Les.2141.1.A1_at	BG734555	BG73455	191	75	86	0.39	0.45	Transcribed locus
Les.3569.1.S1_at	AF059487.1	TC21833	1553	608	1055	0.39	0.68	expansin A3

Les.1977.2.S1_at	AW930109	TC21826	834	326	270	0.39	0.32	Transcribed locus, weakly similar to XP_001097638.1 similar to CG32699-PA [Macaca mulatta]
Les.2529.1.A1_at	BG631274	TC24469	1087	424	784	0.39	0.72	Transcribed locus, weakly similar to NP_001041768.1 [Oryza sativa (japonica cultivar-group)]
Les.4962.1.S1_at	BI204454	TC22450	778	303	702	0.39	0.9	Lycopersicon esculentum clone 114228R, mRNA sequence
Les.3330.2.S1_at	BE458823	TC24355	1703	664	698	0.39	0.41	Transcribed locus, weakly similar to NP_001044764.1 [Oryza sativa (japonica cultivar-group)]
Les.5290.1.S1_at	BT013644.1	TC23296	2617	1019	1603	0.39	0.61	Lycopersicon esculentum clone 132449R, mRNA sequence
LesAffx.66226.1.S1_at	BI926952	TC22735	282	110	319	0.39	1.13	Transcribed locus, weakly similar to NP_001041821.1 [Oryza sativa (japonica cultivar-group)]
Les.1835.1.S1_at	BG629491	TC23603	787	306	442	0.39	0.56	Lycopersicon esculentum clone 132743F, mRNA sequence
LesAffx.71563.1.S1_at	AW930851	TC22892	429	167	188	0.39	0.44	---
Les.828.1.A1_at	BG627663	TC24124	384	149	91	0.39	0.24	Transcribed locus
Les.2545.1.S1_at	BT014360.1	TC22148	3725	1440	2894	0.39	0.78	Lycopersicon esculentum clone 133646R, mRNA sequence
Les.4259.1.S1_at	BG629037	TC24424	3428	1323	1204	0.39	0.35	Transcribed locus, weakly similar to XP_001082599.1 cysteine and glycine-rich protein 2 [Macaca mulatta]
Les.3216.2.S1_at	BG129507	TC22776	2130	821	942	0.39	0.44	Transcribed locus, moderately similar to NP_001043347.1 [Oryza sativa (japonica cultivar-group)]
Les.5419.1.S1_at	BT013877.1	TC23973	1085	418	779	0.39	0.72	Lycopersicon esculentum clone 132857F, mRNA sequence
Les.2547.1.A1_at	BG627349	TC23877	318	122	365	0.38	1.15	Transcribed locus, weakly similar to NP_001041979.1 [Oryza sativa (japonica cultivar-group)]
Les.1988.1.A1_at	BG630760	BG62625	2201	846	698	0.38	0.32	Transcribed locus
Les.4613.1.S1_at	BT013526.1		4698	1803	3213	0.38	0.68	Lycopersicon esculentum clone 132244R, mRNA sequence
Les.4007.1.S1_at	BG629658	TC22991	2626	1007	1283	0.38	0.49	33 kDa oxygen-evolving protein
Les.2626.1.S1_at	BG735008	TC22245	1281	490	1223	0.38	0.95	Lycopersicon esculentum clone 132901F, mRNA sequence
Les.1851.1.A1_at	BG629607	BG62960	934	357	606	0.38	0.65	Transcribed locus
Les.3330.3.A1_at	BG629064	TC24355	1804	686	768	0.38	0.43	Transcribed locus, weakly similar to NP_001044764.1 [Oryza sativa (japonica cultivar-group)]
Les.3500.1.S1_at	AF043085.1	TC21717	750	283	358	0.38	0.48	ethylene receptor homolog
Les.3065.1.S1_at	BI929558	TC22543	310	117	74	0.38	0.24	Transcribed locus, weakly similar to NP_001042793.1 [Oryza sativa (japonica cultivar-group)]
Les.2325.1.S1_at	BG625896	TC22401	271	102	139	0.38	0.51	yfe37 protein
Les.1212.1.S1_at	AY584532.1	GO37246	6864	2577	3489	0.38	0.51	DWARF1/DIMINUTO
Les.3017.1.A1_at	BG629826	AI78132	1113	417	828	0.37	0.74	Transcribed locus, weakly similar to XP_001084411.1 aquaporin 1 [Macaca mulatta]

Les.3216.1.S1_at	AW930422	TC22776	2629	984	1066	0.37	0.41	Transcribed locus, moderately similar to NP_001043347.1 [Oryza sativa (japonica cultivar-group)]
Les.1465.1.S1_at	BG631822	TC23566	1045	391	234	0.37	0.22	Lycopersicon esculentum clone 132131F, mRNA sequence
LesAffx.53474.1.S1_at	BG133264	TC22918	1281	475	509	0.37	0.4	Transcribed locus, weakly similar to NP_001042043.1 [Oryza sativa (japonica cultivar-group)]
LesAffx.5776.1.S1_at	BG127982	BG12798	146	54	66	0.37	0.45	phytochromobilin synthase
Les.3122.1.S1_a_at	BT013364.1	TC21726	108	40	96	0.37	0.89	pectin esterase
LesAffx.770.1.S1_at	BI210904	BI21090	177	66	99	0.37	0.56	---
Les.1784.1.S1_at	BI931777	TC24140	2175	803	554	0.37	0.25	Transcribed locus, weakly similar to XP_001118335.1 similar to intestinal facilitative glucose transporter 7 [Macaca mulatta]
Les.4008.1.S1_at	BG630151	TC21741	4876	1801	2552	0.37	0.52	xyloglucan endotransglycosylase/hydrolase 16 protein
Les.4170.1.A1_at	BG628936	BG62893	9	3	8	0.37	0.91	Transcribed locus
Les.5312.1.S1_at	BT013697.1	TC22735	2479	913	357	0.37	0.14	Lycopersicon esculentum clone 132539F, mRNA sequence
LesAffx.56.16.S1_at	BG643188	AI89840	2285	840	1468	0.37	0.64	Transcribed locus, moderately similar to NP_001059779.1 [Oryza sativa (japonica cultivar-group)]
LesAffx.286.9.S1_at	CK716206	BP89354	2786	1023	885	0.37	0.32	---
Les.5333.1.S1_at	BT013726.1	TC22354	1478	542	993	0.37	0.67	Lycopersicon esculentum clone 132585F, mRNA sequence
LesAffx.66966.1.S1_at	BG135649	TC22051	317	116	174	0.37	0.55	Transcribed locus, weakly similar to XP_001102266.1 alpha-N-acetylgalactosaminidase [Macaca mulatta]
Les.3312.2.S1_at	BI932707	TC21843	4904	1790	2843	0.37	0.58	Transcribed locus, weakly similar to NP_001043993.1 [Oryza sativa (japonica cultivar-group)]
LesAffx.53061.1.S1_at	BI208217	TC23775	392	143	251	0.36	0.64	Transcribed locus, weakly similar to NP_001059549.1 [Oryza sativa (japonica cultivar-group)]
Les.3019.2.S1_at	BG128957	BI93132	493	179	225	0.36	0.46	Transcribed locus, weakly similar to NP_065956.1 dehydrogenase 14 (all-trans and 9-cis) [Homo sapiens]
LesAffx.58019.1.S1_at	BI929233	TC22171	233	84	208	0.36	0.89	Transcribed locus, weakly similar to NP_001052012.1 [Oryza sativa (japonica cultivar-group)]
Les.1197.2.A1_at	BG630003	TC23124	2960	1070	1176	0.36	0.4	Transcribed locus, weakly similar to XP_001090911.1 LanC lantibiotic synthetase component C-like 2 isoform 2 [Macaca mulatta]
Les.5150.1.S1_at	BT013389.1	AW92832	548	197	177	0.36	0.32	Lycopersicon esculentum clone 135570F, mRNA sequence
Les.3299.1.S1_at	BI933484	TC23916	258	93	59	0.36	0.23	Arginase 2
Les.2831.1.S1_at	BG627219	TC22500	3936	1415	2031	0.36	0.52	Lycopersicon esculentum clone 132363R, mRNA sequence
Les.5120.1.S1_at	BT013322.1	TC21818	657	235	222	0.36	0.34	Lycopersicon esculentum clone 135034F, mRNA sequence
LesAffx.14321.1.S1_at	AW737808	AW73780	176	63	51	0.36	0.29	---
Les.1557.1.A1_at	BG626732	TC24262	2121	758	812	0.36	0.38	Transcribed locus
Les.3055.1.S1_at	AJ002590.1	TC21769	453	161	357	0.36	0.79	CycD3;3 protein

Les.4008.2.S1_at	BG643663	TC21741	4486	1595	2258	0.36	0.5	xyloglucan endotransglycosylase/hydrolase 16 protein
Les.181.1.A1_at	BG630947	TC23820	242	86	95	0.35	0.39	Transcribed locus, weakly similar to NP_001043252.1 [Oryza sativa (japonica cultivar-group)]
LesAffx.105.1.S1_at	CK715483	TC22543	70	25	59	0.35	0.84	Transcribed locus, weakly similar to NP_001052215.1 [Oryza sativa (japonica cultivar-group)]
Les.1959.1.A1_at	BG630503	TC23129	74	26	28	0.35	0.38	Transcribed locus
Les.1654.1.A1_at	BG627748	TC23489	2541	897	1011	0.35	0.4	Transcribed locus, weakly similar to NP_001042315.1 [Oryza sativa (japonica cultivar-group)]
LesAffx.71503.1.S1_at	AW648310	TC22254	961	338	538	0.35	0.56	Transcribed locus, weakly similar to NP_001044300.1 [Oryza sativa (japonica cultivar-group)]
Les.4696.1.S1_at	BT014502.1	TC22932	1936	679	772	0.35	0.4	Lycopersicon esculentum clone 133876F, mRNA sequence
Les.1258.1.S1_at	AY240230.1	TC21731	2255	790	467	0.35	0.21	gamma-aminobutyrate transaminase subunit precursor isozyme 2
Les.1042.1.A1_at	BG629025	TC22972	134	47	119	0.35	0.89	Transcribed locus, weakly similar to NP_001042141.1 [Oryza sativa (japonica cultivar-group)]
Les.778.1.A1_at	BG627417	TC23561	486	169	322	0.35	0.66	Transcribed locus
Les.4444.1.S1_s_at	BI207010	TC22444	235	81	186	0.34	0.79	CycD3;3 protein
LesAffx.69264.1.S1_at	BF113833	TC23903	1648	566	1212	0.34	0.74	Transcribed locus, weakly similar to NP_001047317.1 [Oryza sativa (japonica cultivar-group)]
Les.6.1.S1_at	AF261141.1	TC22389	939	322	322	0.34	0.34	hypothetical LOC543656
Les.3409.1.A1_at	BG735108	GO37597	2962	1016	915	0.34	0.31	Transcribed locus, weakly similar to NP_001044764.1 [Oryza sativa (japonica cultivar-group)]
Les.5832.1.S1_at	BT014414.1	TC22401	2216	760	463	0.34	0.21	Lycopersicon esculentum clone 133722R, mRNA sequence
LesAffx.65028.1.S1_at	BG128587	TC22082	2314	790	1172	0.34	0.51	Transcribed locus, weakly similar to NP_001054271.1 [Oryza sativa (japonica cultivar-group)]
Les.305.3.S1_at	AW930072	TC23404	1365	462	386	0.34	0.28	Transcribed locus, weakly similar to XP_001100725.1 similar to eukaryotic translation initiation factor 2C, 2 [Macaca mulatta]
Les.1197.1.S1_at	BI925087	TC22471	858	290	273	0.34	0.32	Transcribed locus, weakly similar to XP_001090911.1 LanC lantibiotic synthetase component C-like 2 isoform 2 [Macaca mulatta]
LesAffx.10497.2.A1_at	AI897776	TC24435	2131	716	765	0.34	0.36	Transcribed locus, weakly similar to NP_001042618.1 [Oryza sativa (japonica cultivar-group)]
Les.305.2.S1_at	CD002775	TC23749	2555	856	776	0.34	0.3	Transcribed locus, weakly similar to XP_001100725.1 similar to eukaryotic translation initiation factor 2C, 2 [Macaca mulatta]
Les.3017.3.S1_at	BE462343	TC21898	760	254	526	0.33	0.69	Transcribed locus, weakly similar to XP_001084411.1 aquaporin 1 [Macaca mulatta]
LesAffx.10279.1.S1_at	CK720579	TC23575	356	119	178	0.33	0.5	Transcribed locus, weakly similar to NP_001043142.1 [Oryza sativa (japonica cultivar-group)]

Les.4473.1.S1_at	AJ606077.1	TC22116	586	195	285	0.33	0.49	spe4 protein
Les.3123.1.S1_at	BT013406.1	TC23016	2549	846	917	0.33	0.36	Lycopersicon esculentum clone 135721R, mRNA sequence
Les.369.1.S1_at	AF059489.1	TC21922	1890	622	1420	0.33	0.75	expansin
Les.263.1.S1_at	M98466.1	TC21722	3864	1268	4277	0.33	1.11	polygalacturonase isoenzyme 1 beta subunit
Les.4835.1.S1_at	BT012722.1	TC24498	370	121	149	0.33	0.4	Lycopersicon esculentum clone 113649F, mRNA sequence
Les.4987.1.S1_at	BT013055.1	TC23511	2308	756	1346	0.33	0.58	Lycopersicon esculentum clone 114311F, mRNA sequence
Les.174.1.A1_at	BG627544	TC24015	365	119	92	0.33	0.25	Transcribed locus, moderately similar to NP_001045188.1 [Oryza sativa (japonica cultivar-group)]
Les.70.1.S1_at	AF000141.1	NP00014	2600	848	1027	0.33	0.39	class I knotted-like homeodomain protein
LesAffx.41518.1.S1_at	AW930080	TC23094	265	86	140	0.32	0.53	Transcribed locus, weakly similar to NP_001050851.1 [Oryza sativa (japonica cultivar-group)]
Les.4731.1.S1_at	BT013577.1	TC23297	2609	845	1921	0.32	0.74	Lycopersicon esculentum clone 132322R, mRNA sequence
Les.2316.2.A1_at	BG626036	TC22940	3248	1050	172	0.32	0.05	Transcribed locus, weakly similar to NP_001044252.1 [Oryza sativa (japonica cultivar-group)]
Les.3409.2.S1_at	BF050508	TC21920	1653	532	440	0.32	0.27	Transcribed locus, weakly similar to NP_001044764.1 [Oryza sativa (japonica cultivar-group)]
Les.1108.1.A1_at	BG629434	TC22374	419	135	467	0.32	1.11	Transcribed locus, weakly similar to NP_001042402.1 [Oryza sativa (japonica cultivar-group)]
LesAffx.65984.1.S1_at	BG125327	GO37430	2600	833	791	0.32	0.3	Transcribed locus, weakly similar to NP_001044052.1 [Oryza sativa (japonica cultivar-group)]
Les.252.1.S1_at	U35643.1	DB68734	2965	945	1343	0.32	0.45	Alpha-dioxygenase 2
LesAffx.10279.1.A1_at	CK720579	TC23575	515	163	256	0.32	0.5	Transcribed locus, weakly similar to NP_001043142.1 [Oryza sativa (japonica cultivar-group)]
Les.4738.1.S1_at	BT013480.1	GO37558	2215	702	1325	0.32	0.6	Lycopersicon esculentum clone 132163F, mRNA sequence
Les.3630.1.S1_at	U50985.1	TC21720	224	71	204	0.32	0.91	pectin esterase
Les.2923.2.S1_at	BI935057	TC23094	2412	763	1225	0.32	0.51	Transcribed locus, weakly similar to NP_001044052.1 [Oryza sativa (japonica cultivar-group)]
Les.513.1.S1_at	BG626001	TC22866	301	95	68	0.32	0.23	Lycopersicon esculentum clone 132360F, mRNA sequence
Les.2091.1.S1_at	BT013457.1	TC22626	893	282	438	0.32	0.49	Lycopersicon esculentum clone 132113R, mRNA sequence
LesAffx.66226.2.S1_at	BI205336	TC22735	802	253	929	0.32	1.16	Transcribed locus, weakly similar to NP_001041821.1 [Oryza sativa (japonica cultivar-group)]
Les.204.1.S1_at	L41126.1	TC21759	2599	816	1150	0.31	0.44	ADP-glucose pyrophosphorylase small subunit
Les.1376.2.A1_at	BG629093	TC23229	729	228	420	0.31	0.58	Transcribed locus, weakly similar to NP_001043392.1 [Oryza sativa (japonica cultivar-group)]
Les.1376.1.A1_at	BG631198	TC23229	2283	715	1191	0.31	0.52	Transcribed locus, weakly similar to NP_001043392.1 [Oryza sativa (japonica cultivar-group)]

Les.3303.1.S1_at	BT012987.1	TC23470	1909	591	689	0.31	0.36	Lycopersicon esculentum clone 114205R, mRNA sequence
Les.4334.3.A1_at	BG735063	TC22597	127	39	66	0.31	0.52	Transcribed locus, weakly similar to XP_001109838.1 ribulose-5-phosphate-3-epimerase isoform 5 [Macaca mulatta]
Les.642.1.S1_at	BG626672	BG62667	112	35	46	0.31	0.41	Lycopersicon esculentum clone 132200F, mRNA sequence
LesAffx.61000.1.S1_at	BM410987	TC23050	1059	324	287	0.31	0.27	Transcribed locus, weakly similar to NP_001045184.1 [Oryza sativa (japonica cultivar-group)]
Les.4312.2.S1_at	BG626083	TC22795	3678	1114	680	0.3	0.18	CDNA, clone: FC02DB04, HTC in fruit
Les.406.1.A1_at	BG629308	TC24082	933	282	564	0.3	0.6	Transcribed locus, weakly similar to NP_196850.1 binding [Arabidopsis thaliana]
Les.4405.1.A1_at	BG630861	TC24428	1572	476	216	0.3	0.14	Transcribed locus, moderately similar to NP_001047599.1 [Oryza sativa (japonica cultivar-group)]
LesAffx.39973.1.S1_at	BI932177	DV10487	278	84	124	0.3	0.45	Transcribed locus, weakly similar to NP_001044052.1 [Oryza sativa (japonica cultivar-group)]
Les.3065.2.A1_at	BG630869	TC22543	538	160	92	0.3	0.17	Transcribed locus, weakly similar to NP_001042793.1 [Oryza sativa (japonica cultivar-group)]
LesAffx.70738.1.A1_at	BG630069	TC23199	1053	310	302	0.29	0.29	Transcribed locus, weakly similar to XP_001101267.1 similar to myeloblastosis proto-oncogene product isoform 2 [Macaca mulatta]
Les.2092.2.S1_at	BI203427	BG63176	1338	393	620	0.29	0.46	Transcribed locus, weakly similar to XP_001111095.1 aldolase B isoform 5 [Macaca mulatta]
Les.1205.2.S1_at	BM536081	TC21911	690	202	100	0.29	0.14	Transcribed locus, weakly similar to NP_001059455.1 [Oryza sativa (japonica cultivar-group)]
LesAffx.13252.1.S1_at	BI929109	TC22202	2857	831	1463	0.29	0.51	Transcribed locus, weakly similar to NP_001042395.1 [Oryza sativa (japonica cultivar-group)]
Les.852.1.A1_at	BG627744	TC24030	88	25	20	0.29	0.23	Transcribed locus
Les.3322.1.S1_at	BI935557	TC22083	608	175	69	0.29	0.11	Transcribed locus, moderately similar to NP_001047599.1 [Oryza sativa (japonica cultivar-group)]
LesAffx.13252.2.A1_at	BG124820	TC22202	519	149	200	0.29	0.38	Transcribed locus, weakly similar to NP_001042395.1 [Oryza sativa (japonica cultivar-group)]
LesAffx.41330.1.A1_at	CK574967	TC24064	205	59	90	0.29	0.44	Transcribed locus, weakly similar to NP_001067668.1 [Oryza sativa (japonica cultivar-group)]
Les.3322.3.S1_at	AW443800	BP90402	852	244	118	0.29	0.14	Transcribed locus, moderately similar to NP_001047599.1 [Oryza sativa (japonica cultivar-group)]
Les.5161.1.S1_at	BT013412.1	AI77756	289	83	115	0.29	0.4	Lycopersicon esculentum clone 132049R, mRNA sequence
LesAffx.41330.2.S1_at	AI899559	TC24064	324	92	128	0.29	0.4	Transcribed locus, weakly similar to NP_001067668.1 [Oryza sativa (japonica cultivar-group)]
Les.3064.1.S1_at	U81033.1	TC21721	1463	418	596	0.29	0.41	ADP-glucose pyrophosphorylase large subunit

LesAffx.70270.1.S1_at	AI773931	TC23665	3342	953	1469	0.29	0.44	Transcribed locus, moderately similar to NP_001046164.1 [Oryza sativa (japonica cultivar-group)]
LesAffx.64757.1.A1_at	AI490042	TC22570	292	83	102	0.28	0.35	Transcribed locus
Les.547.1.S1_at	AW648331	TC22628	296	84	305	0.28	1.03	Transcribed locus
Les.2316.1.S1_at	AW623527	TC22940	3123	881	146	0.28	0.05	Transcribed locus, weakly similar to NP_001044252.1 [Oryza sativa (japonica cultivar-group)]
Les.360.1.S1_at	M86724.1	TC23986	4174	1174	5063	0.28	1.21	alcohol dehydrogenase
Les.1205.1.A1_at	BG630057	TC21911	471	132	115	0.28	0.24	Transcribed locus, weakly similar to NP_001059455.1 [Oryza sativa (japonica cultivar-group)]
LesAffx.885.1.S1_at	BI205930	BI20593	352	98	130	0.28	0.37	---
Les.2092.1.S1_at	BG626759	TC22657	2338	651	1349	0.28	0.58	Transcribed locus, weakly similar to XP_001111095.1 aldolase B isoform 5 [Macaca mulatta]
Les.4794.1.S1_at	BT014116.1	TC23742	2907	806	886	0.28	0.3	Lycopersicon esculentum clone 133221F, mRNA sequence
Les.3972.1.S1_at	AJ243340.1	TC22219	4144	1136	4381	0.27	1.06	expansin9
Les.4136.1.S1_at	AJ538329.1	NP59756	1016	278	145	0.27	0.14	wuschel protein
Les.1205.3.S1_at	BM410805	TC21911	1136	310	145	0.27	0.13	Transcribed locus, weakly similar to NP_001059455.1 [Oryza sativa (japonica cultivar-group)]
LesAffx.48215.1.S1_at	AW945008	TC23968	2359	642	893	0.27	0.38	Transcribed locus, weakly similar to NP_001043647.1 [Oryza sativa (japonica cultivar-group)]
Les.1348.1.A1_at	BG631005	TC22489	1419	386	607	0.27	0.43	Transcribed locus, weakly similar to NP_199517.1 protein [Arabidopsis thaliana]
LesAffx.4981.1.S1_at	AW623022	TC22429	3443	928	715	0.27	0.21	Transcribed locus, weakly similar to NP_001041979.1 [Oryza sativa (japonica cultivar-group)]
Les.4397.2.A1_at	AW218741	TC23761	671	179	251	0.27	0.37	CDNA, clone: FC25AG12, HTC in fruit
LesAffx.61034.1.S1_at	BG643132	TC21983	1159	308	728	0.27	0.63	Transcribed locus, weakly similar to NP_001043558.1 [Oryza sativa (japonica cultivar-group)]
Les.5850.1.S1_at	BT014189.1	TC21894	350	92	164	0.26	0.47	Lycopersicon esculentum clone 133366R, mRNA sequence
Les.876.1.A1_at	BG627344	TC22377	179	46	45	0.26	0.25	Transcribed locus, weakly similar to NP_001051973.1 [Oryza sativa (japonica cultivar-group)]
Les.3069.1.S1_at	BG630752	TC21938	695	175	447	0.25	0.64	Lycopersicon esculentum clone 132988F, mRNA sequence
LesAffx.66974.1.S1_at	BG135595	TC23429	106	26	125	0.25	1.19	Transcribed locus, weakly similar to NP_001046313.1 [Oryza sativa (japonica cultivar-group)]
Les.4397.1.S1_at	BG127578	TC23761	263	65	101	0.25	0.38	CDNA, clone: FC25AG12, HTC in fruit
Les.2474.1.S1_at	BG791226	TC23985	839	204	211	0.24	0.25	Transcribed locus, weakly similar to XP_001108186.1 similar to 24-dehydrocholesterol reductase precursor [Macaca mulatta]
LesAffx.64757.1.S1_at	AI490042	TC22570	464	112	182	0.24	0.39	Transcribed locus

Les.2474.2.A1_at	BG735266	TC23985	1462	354	398	0.24	0.27	Transcribed locus, weakly similar to XP_001108186.1 similar to 24-dehydrocholesterol reductase precursor [Macaca mulatta]
LesAffx.53591.1.S1_at	AI899018	TC22292	122	29	38	0.24	0.31	Transcribed locus, weakly similar to XP_001101267.1 similar to myeloblastosis proto-oncogene product isoform 2 [Macaca mulatta]
Les.5918.1.S1_at	AY224079.1	TC21718	143	34	57	0.24	0.4	ferric-chelate reductase
Les.3519.1.S1_at	AJ002589.1	TC23298	577	138	350	0.24	0.61	CycD3;2 protein
Les.1951.1.A1_at	BG630440	TC23845	5340	1270	2134	0.24	0.4	Transcribed locus, weakly similar to NP_188563.1 binding [Arabidopsis thaliana]
LesAffx.13252.2.S1_at	BG124820	TC22202	2033	482	916	0.24	0.45	Transcribed locus, weakly similar to NP_001042395.1 [Oryza sativa (japonica cultivar-group)]
LesAffx.56547.2.S1_at	BI207815	TC22569	1323	312	231	0.24	0.17	Transcribed locus, weakly similar to NP_001042475.1 [Oryza sativa (japonica cultivar-group)]
LesAffx.46085.1.S1_at	AW928724	TC22038	411	96	80	0.23	0.2	Transcribed locus, weakly similar to XP_001114240.1 cytochrome P450, family 21, subfamily A, polypeptide 2 isoform 3 [Macaca mulatta]
Les.15.1.S1_at	X98929.1	TC21718	3016	705	2083	0.23	0.69	SBT1 protein
LesAffx.44609.1.S1_at	BG631285	TC23469	1392	322	940	0.23	0.68	Transcribed locus, weakly similar to NP_187615.2 factor [Arabidopsis thaliana]
Les.5941.1.A1_at	CK715706	TC22893	1396	322	728	0.23	0.52	Transcribed locus, weakly similar to NP_001042315.1 [Oryza sativa (japonica cultivar-group)]
Les.1549.1.A1_at	BG626692	TC23631	1106	253	392	0.23	0.35	Transcribed locus
LesAffx.16472.1.S1_at	AI774397	TC24204	227	52	154	0.23	0.68	---
Les.68.2.S1_a_at	BT013727.1	TC21796	2320	524	1106	0.23	0.48	endo-beta-1,4-D-glucanase
Les.4749.1.S1_at	BT013546.1	AI77484	554	124	254	0.22	0.46	Lycopersicon esculentum clone 132267F, mRNA sequence
Les.3792.1.S1_s_at	AF249329.1	GO37419	2871	631	611	0.22	0.21	Lycopersicon esculentum putative cytochrome P450
Les.857.1.S1_at	BT013838.1	TC22565	693	151	159	0.22	0.23	Lycopersicon esculentum clone 132795F, mRNA sequence
LesAffx.1251.1.S1_at	BG125851	BG12585	266	58	222	0.22	0.83	---
Les.4710.1.S1_at	BT014526.1	TC24079	94	20	17	0.22	0.18	Lycopersicon esculentum clone 133940F, mRNA sequence
Les.1714.1.A1_at	BG628281	TC24400	471	100	299	0.21	0.63	Transcribed locus
Les.33.1.S1_at	AF148934.1	TC22018	2943	614	2034	0.21	0.69	phantastica
Les.4065.1.A1_at	L24019.1		66	14	22	0.21	0.33	Lycopersicon esculentum (DB166) meloidogyne-induced giant cell protein mRNA, 3' end
Les.3372.1.S1_at	BG631141	TC23249	2722	556	577	0.2	0.21	Lycopersicon esculentum clone 132813R, mRNA sequence
Les.9.1.S1_at	AB010992.1	TC22813	1634	324	323	0.2	0.2	3b-hydroxylase
LesAffx.23349.1.S1_at	AI782014	TC21920	1000	193	764	0.19	0.76	ADP/ATP translocator
Les.1614.1.A1_at	BG628742	TC23793	554	102	209	0.18	0.38	Transcribed locus, moderately similar to NP_001049792.1 [Oryza sativa (japonica cultivar-group)]
Les.4938.1.S1_at	BT012940.1	TC22310	2255	399	643	0.18	0.29	Lycopersicon esculentum clone 114108R, mRNA sequence

LesAffx.9038.1.S1_at	BI930643	TC22956	957	164	161	0.17	0.17	Transcribed locus, weakly similar to NP_001042297.1 [Oryza sativa (japonica cultivar-group)]
Les.5664.1.S1_at	BT014348.1	AW03195	881	146	1364	0.17	1.55	Lycopersicon esculentum clone 133623F, mRNA sequence
LesAffx.56.4.S1_at	BG128707	TC22262	421	62	296	0.15	0.7	Transcribed locus, weakly similar to XP_001087943.1 similar to carboxypeptidase, vitellogenic-like isoform 4 [Macaca mulatta]
Les.4594.1.S1_at	BT012739.1	TC23704	3669	513	1376	0.14	0.38	Lycopersicon esculentum clone 113677R, mRNA sequence
LesAffx.3099.1.S1_at	CK574995	GO37548	5135	577	611	0.11	0.12	Transcribed locus, moderately similar to NP_001044625.1 [Oryza sativa (japonica cultivar-group)]
