# Supplementary material for: Transcriptome Analysis of Tomato Flower Pedicel Tissues Reveals Abscission Zone-Specific Modulation of Key Meristem Activity Genes
Source: PLoS One. 2013 Feb 4;8(2):e55238. doi: 10.1371/journal.pone.0055238 (PMC3563536; doi:10.1371/journal.pone.0055238)
Supplement: Table S4 — Differentially expressed genes in the basal portion after 3 h or 6 h ethylene treatment. (PDF) [file pone.0055238.s006.pdf]

Table S4. Differentially expressed genes in the basal portion after 3 h and/or 6 h ethylene treatment.

Affymetrix Probe Set ID	GenBank Accession	Tomato Gene Index	0BP	3BP	6BP	3BP/0BP	6BP/0BP	GenBank Annotation
Les.3646.1.S1_at	U70481.1	TC21735	190	1814	1522	9.57	8.03	abscission polygalacturonase
LesAffx.56389.1.S1_at	AW034707	TC23934	168	1459	1257	8.67	7.47	Transcribed locus, weakly similar to NP_001033715.2 F synthase [Sus scrofa]
Les.4885.1.S1_at	BT012835.1	TC24138	559	4064	3024	7.27	5.41	Lycopersicon esculentum clone 113896R, mRNA sequence
Les.3665.1.S1_at	U23053.1	TC21734	9	58	75	6.79	8.79	polygalacturonase precursor
Les.132.1.S1_at	X58885.1	TC21743	1017	6766	7754	6.65	7.63	ethylene-forming enzyme
Les.3712.1.S1_at	AF154003.1	TC24028	443	2198	1780	4.96	4.02	pirin
Les.3647.1.S1_at	U70480.1	TC21734	89	414	361	4.68	4.07	abscission polygalacturonase
Les.113.1.S1_at	CN384955	TC23130	940	4379	3468	4.66	3.69	CDNA, clone: FC02BG03, HTC in fruit
LesAffx.31873.1.S1_at	BI928574	BI92857	108	432	957	4.02	8.9	---
LesAffx.13365.1.S1_at	BM411110	TC21920	588	2335	765	3.97	1.3	Transcribed locus, weakly similar to NP_001066487.1 [Oryza sativa (japonica cultivar-group)]
LesAffx.70406.1.S1_at	AW036249	TC22360	103	398	610	3.87	5.92	Transcribed locus, weakly similar to XP_001114236.1 branched chain aminotransferase 2, mitochondrial isoform 2 [Macaca mulatta]
Les.3818.1.S1_at	AF502085.1	TC21752	90	331	388	3.69	4.33	ethylene responsive element binding protein
LesAffx.64582.2.A1_at	AI490624	TC24338	107	377	199	3.54	1.87	---
LesAffx.64582.1.S1_at	AI894448	TC24338	243	834	441	3.43	1.81	---
Les.244.2.S1_a_at	AI485696	TC22935	341	1145	1009	3.36	2.96	Calmodulin
LesAffx.54536.1.S1_at	BM409044	TC23408	233	779	630	3.34	2.71	Transcribed locus, weakly similar to NP_001046834.1 [Oryza sativa (japonica cultivar-group)]
LesAffx.63659.1.S1_at	BI921813	TC22794	389	1272	1078	3.27	2.77	Transcribed locus, weakly similar to NP_001047062.1 [Oryza sativa (japonica cultivar-group)]
LesAffx.33607.1.S1_s_at	AW223067	TC23890	463	1480	1331	3.2	2.88	Calmodulin
LesAffx.50112.1.S1_at	AI898985	TC23361	532	1676	1458	3.15	2.74	Transcribed locus, weakly similar to NP_001047062.1 [Oryza sativa (japonica cultivar-group)]
Les.2084.1.S1_at	BF097539	TC22764	518	1632	1251	3.15	2.41	Transcribed locus, weakly similar to NP_001041763.1 [Oryza sativa (japonica cultivar-group)]
LesAffx.33607.2.S1_s_at	AI775872	TC23890	1215	3815	3399	3.14	2.8	Calmodulin
LesAffx.56301.1.S1_at	AI895341	TC22182	1312	4090	4843	3.12	3.69	Transcribed locus, weakly similar to NP_001048145.1 [Oryza sativa (japonica cultivar-group)]
Les.5915.1.S1_at	AY344539.1	TC22182	23	71	148	3.09	6.41	alpha-DOX1
Les.1524.1.A1_at	BG626458	BG62645	471	1443	964	3.07	2.05	Transcribed locus
LesAffx.69349.2.S1_at	AW623862	AW62386	243	739	749	3.04	3.09	Transcribed locus, weakly similar to NP_001043801.1 [Oryza sativa (japonica cultivar-group)]

LesAffx.63739.1.S1_at	AW033134	TC21965	167	502	292	3	1.74	Transcribed locus, weakly similar to NP_001043665.1 [Oryza sativa (japonica cultivar-group)]
LesAffx.61149.1.S1_at	BE460847	TC24084	284	845	1294	2.97	4.55	Transcribed locus, weakly similar to NP_001042177.1 [Oryza sativa (japonica cultivar-group)]
Les.4287.1.S1_at	CB751564	TC21784	1204	3536	5184	2.94	4.31	Transcribed locus, weakly similar to XP_001094505.1 proteasome 26S non-ATPase subunit 3 isoform 4 [Macaca mulatta]
Les.2899.1.S1_at	BG628131	TC22810	3975	11581	7784	2.91	1.96	Transcribed locus, weakly similar to XP_001108862.1 cathepsin H isoform 2 [Macaca mulatta]
LesAffx.60582.1.S1_at	AI899413	TC23540	128	372	195	2.9	1.52	Transcribed locus, weakly similar to NP_001048083.1 [Oryza sativa (japonica cultivar-group)]
LesAffx.52437.1.S1_at	BI925563	TC23341	257	738	266	2.87	1.04	Transcribed locus, weakly similar to NP_001044627.1 [Oryza sativa (japonica cultivar-group)]
LesAffx.58502.1.S1_at	BM412364	TC22738	635	1781	1087	2.81	1.71	Transcribed locus, weakly similar to NP_001042057.1 [Oryza sativa (japonica cultivar-group)]
LesAffx.8850.1.S1_at	AW034398	TC22181	118	330	1018	2.8	8.65	Subtilisin-like protease
Les.423.1.S1_at	BM409421	TC22953	998	2794	1641	2.8	1.64	CDNA, clone: FC09CF07, HTC in fruit
Les.1046.1.S1_at	X72730.1	TC22785	359	1003	575	2.79	1.6	(ERT 10) ripening-related mRNA
Les.244.2.S1_at	AI485696	TC22935	161	448	298	2.79	1.85	Calmodulin
LesAffx.71660.2.S1_at	AW218025	TC24516	1	3	4	2.73	3.25	---
LesAffx.22583.1.S1_at	AI484022	TC23670	319	868	758	2.73	2.38	Transcribed locus, weakly similar to NP_001044974.1 [Oryza sativa (japonica cultivar-group)]
LesAffx.68306.1.S1_at	BE461086	TC23074	144	391	131	2.72	0.91	Transcribed locus, weakly similar to NP_001055605.1 [Oryza sativa (japonica cultivar-group)]
Les.4341.1.A1_at	BG626075	TC23787	4	11	9	2.72	2.22	Transcribed locus
LesAffx.62138.1.S1_at	AW737374	TC24364	107	288	251	2.69	2.34	---
Les.4527.1.S1_at	AJ635323.1	TC22837	44	118	97	2.69	2.22	polyphenol oxidase A
LesAffx.57163.2.S1_at	BI421649	TC22802	621	1652	210	2.66	0.34	Transcribed locus, weakly similar to NP_001044707.1 [Oryza sativa (japonica cultivar-group)]
Les.3693.1.S1_at	AF426174.1	TC22813	38	100	92	2.65	2.43	blind
Les.3645.1.S1_at	U54770.1	NP924378	926	2443	2252	2.64	2.43	6-deoxocastasterone oxidase
LesAffx.62232.1.S1_at	AW649113	TC23914	60	159	166	2.64	2.74	Transcribed locus, weakly similar to NP_001048167.1 [Oryza sativa (japonica cultivar-group)]
LesAffx.70522.1.S1_at	BI423015	TC23029	480	1264	1366	2.63	2.85	Transcribed locus, weakly similar to NP_001055496.1 [Oryza sativa (japonica cultivar-group)]
Les.3545.1.S1_at	Y14809.1	TC22310	1073	2804	1229	2.61	1.15	beta-carotene hydroxylase

LesAffx.50687.1.S1_at	AI773710	TC22384	514	1337	1403	2.6	2.73	Transcribed locus, weakly similar to NP_001046905.1 [Oryza sativa (japonica cultivar-group)]
Les.702.1.S1_at	BG627003	TC21772	956	2486	1111	2.6	1.16	Lycopersicon esculentum clone 134862F, mRNA sequence
Les.1115.1.A1_at	BG629468	BG62946	12	31	28	2.54	2.29	Transcribed locus
Les.4034.1.S1_at	AF204789.1	BW68739	7	16	9	2.51	1.44	Lycopersicon esculentum ripening regulated protein (DDTFR20) mRNA, partial sequence
Les.4307.1.S1_at	AY257487.1	TC21819	3580	8972	12193	2.51	3.41	PR5-like protein
Les.3610.1.S1_at	BG629712	TC23477	53	133	238	2.5	4.47	glycine rich protein
Les.5051.1.S1_at	BT013182.1	TC22934	195	488	512	2.5	2.63	Lycopersicon esculentum clone 134258F, mRNA sequence
Les.3619.1.S1_at	J04099.1	TC23203	2446	6027	8095	2.46	3.31	proteinase inhibitor I
LesAffx.4116.1.S1_at	BI933542	TC23165	101	247	225	2.45	2.24	Transcribed locus, weakly similar to NP_001047564.1 [Oryza sativa (japonica cultivar-group)]
LesAffx.3467.1.S1_at	CN384781	TC23179	397	974	1287	2.45	3.24	Transcribed locus, weakly similar to NP_001047645.1 [Oryza sativa (japonica cultivar-group)]
Les.5126.1.S1_at	BT013336.1	TC22310	307	751	565	2.45	1.84	Lycopersicon esculentum clone 135126R, mRNA sequence
Les.4335.2.S1_at	AW931003	TC22074	980	2390	985	2.44	1.01	Pathogenesis-related protein P2
LesAffx.20450.1.S1_at	AW223514	TC21776	2363	5757	5293	2.44	2.24	Transcribed locus, weakly similar to NP_001042868.1 [Oryza sativa (japonica cultivar-group)]
LesAffx.51158.2.S1_at	AI898095	TC23014	30	73	62	2.42	2.06	Transcribed locus, weakly similar to NP_001048008.1 [Oryza sativa (japonica cultivar-group)]
LesAffx.66953.1.S1_at	AW651552	TC23604	1494	3608	4184	2.41	2.8	Transcribed locus, weakly similar to NP_001042903.1 [Oryza sativa (japonica cultivar-group)]
Les.4022.1.S1_at	X94946.1	TC21814	368	889	433	2.41	1.18	proteinase inhibitor II
LesAffx.3467.1.A1_at	CN384781	TC23179	185	441	569	2.38	3.07	Transcribed locus, weakly similar to NP_001047645.1 [Oryza sativa (japonica cultivar-group)]
LesAffx.66354.1.S1_at	AI773917	TC22427	168	399	287	2.37	1.71	Transcribed locus, weakly similar to NP_001057807.1 [Oryza sativa (japonica cultivar-group)]
Les.5152.1.S1_at	BT013393.1	TC24469	462	1088	794	2.36	1.72	Lycopersicon esculentum clone 135647R, mRNA sequence
Les.4443.1.A1_s_at	BG630528	TC21730	1261	2893	2623	2.29	2.08	6-deoxocastasterone oxidase
LesAffx.37563.1.S1_at	Z70216.1	TC23549	215	489	535	2.28	2.49	---
LesAffx.71623.1.S1_at	AW035747	TC24095	1496	3381	2761	2.26	1.85	Transcribed locus, weakly similar to NP_001041763.1 [Oryza sativa (japonica cultivar-group)]
LesAffx.51158.1.S1_at	AI897068	TC23014	34	77	60	2.24	1.74	Transcribed locus, weakly similar to NP_001048008.1 [Oryza sativa (japonica cultivar-group)]
Les.5146.1.S1_at	BT013382.1	AW64874	156	349	253	2.23	1.62	Lycopersicon esculentum clone 135514R, mRNA sequence

LesAffx.67592.1.S1_at	AW093105	TC22664	166	368	270	2.22	1.63	Transcribed locus, weakly similar to NP_061135.1 (Asp-Glu-Ala-Asp) box polypeptide 43 [Homo sapiens]
Les.2899.2.S1_at	BM536145	TC22810	1702	3733	2893	2.19	1.7	Transcribed locus, weakly similar to XP_001108862.1 cathepsin H isoform 2 [Macaca mulatta]
Les.3716.1.S1_at	AJ277944.1	TC22806	428	938	585	2.19	1.36	SANT/MYB domain protein
LesAffx.64741.1.S1_at	AI775317	TC22074	1003	2183	2060	2.18	2.05	Transcribed locus, weakly similar to XP_001103467.1 similar to 3-hydroxyisobutyryl-Coenzyme A hydrolase isoform 1 [Macaca mulatta]
LesAffx.24637.1.S1_at	CN550618	TC22796	166	361	710	2.17	4.26	Transcribed locus, weakly similar to NP_001049089.1 [Oryza sativa (japonica cultivar-group)]
LesAffx.15921.1.S1_at	BM412286	TC23758	608	1316	1384	2.17	2.28	Transcribed locus, weakly similar to NP_001057315.1 [Oryza sativa (japonica cultivar-group)]
LesAffx.56070.1.S1_at	BF113259	TC23846	416	895	757	2.15	1.82	Transcribed locus, weakly similar to NP_001043915.1 [Oryza sativa (japonica cultivar-group)]
LesAffx.49488.1.S1_at	AI895632	TC23120	329	709	336	2.15	1.02	---
LesAffx.69998.1.S1_at	BI203963	TC23259	79	168	160	2.14	2.02	Transcribed locus, moderately similar to NP_001048806.1 [Oryza sativa (japonica cultivar-group)]
LesAffx.124.1.S1_at	AW443936	TC23232	177	377	339	2.14	1.92	CDNA, clone: FC20CD10, HTC in fruit
Les.2988.1.S1_at	CK715255	TC22567	1991	4202	2243	2.11	1.13	Sucrose transporter
Les.2353.1.A1_at	BG629059	TC23606	18	38	29	2.1	1.58	Transcribed locus
Les.1431.1.A1_at	BG631562	TC23902	376	791	896	2.1	2.38	Transcribed locus, moderately similar to NP_001043710.1 [Oryza sativa (japonica cultivar-group)]
Les.3455.1.S1_at	AF243040.1	NP33401	5	11	9	2.09	1.75	receptor-like protein kinase 3
LesAffx.70722.1.S1_at	AW030137	TC22649	485	1011	370	2.09	0.76	Transcribed locus, moderately similar to NP_001045420.1 [Oryza sativa (japonica cultivar-group)]
Les.2667.2.S1_at	AW621251	TC22610	2065	4303	3517	2.08	1.7	Transcribed locus, weakly similar to NP_001042221.1 [Oryza sativa (japonica cultivar-group)]
Les.4895.1.S1_at	BT012856.1	TC23595	2087	4345	2000	2.08	0.96	Lycopersicon esculentum clone 113942F, mRNA sequence
Les.5091.1.S1_at	BT013255.1	TC22888	341	709	476	2.08	1.4	Lycopersicon esculentum clone 134773R, mRNA sequence
Les.3752.1.S1_at	AW036285	NP16439	597	1239	674	2.08	1.13	CDNA, clone: FC23CF12, HTC in fruit
LesAffx.71065.1.S1_at	BM413117	TC22472	1421	2935	2297	2.07	1.62	Transcribed locus, weakly similar to NP_001042582.1 [Oryza sativa (japonica cultivar-group)]
Les.934.1.S1_at	BG628248	GO37331	1599	3292	2000	2.06	1.25	Cell wall protein
LesAffx.71606.1.S1_s_at	AI896002	TC24027	3258	6700	6087	2.06	1.87	Lycopersicon esculentum clone 114334F, mRNA sequence
Les.28.2.S1_a_at	AF092654.1	TC23972	61	125	69	2.05	1.13	NRT2;1 protein
Les.59.1.S1_at	AF014809.1	TC22468	1736	3552	4040	2.05	2.33	proline transporter 2
Les.3289.1.S1_at	AY240229.1	TC21835	3049	6225	4919	2.04	1.61	gamma-aminobutyrate transaminase subunit precursor isozyme 1

LesAffx.70414.1.S1_at	AW034339	TC21936	1694	3454	2654	2.04	1.57	Transcribed locus, weakly similar to NP_001045308.1 [Oryza sativa (japonica cultivar-group)]
LesAffx.9910.1.S1_at	CN385590	TC22147	733	1494	1880	2.04	2.56	Transcribed locus, weakly similar to NP_001046094.1 [Oryza sativa (japonica cultivar-group)]
LesAffx.62131.1.S1_at	BE437087	TC23252	1649	3342	1770	2.03	1.07	Transcribed locus, weakly similar to XP_001081745.1 similar to threonine aldolase 1 [Rattus norvegicus]
Les.796.1.A1_at	BG627506	TC22633	675	1365	901	2.02	1.33	Transcribed locus, weakly similar to NP_001043676.1 [Oryza sativa (japonica cultivar-group)]
Les.5130.1.S1_at	BT013342.1	TC23433	274	552	714	2.02	2.61	Lycopersicon esculentum clone 135145F, mRNA sequence
Les.3986.1.S1_at	AF454634.1	TC22373	8	16	14	2.01	1.75	Lycopersicon esculentum cytochrome P450 CYP74C3
LesAffx.64990.1.S1_at	BI422984	TC21791	542	1083	885	2	1.63	Transcribed locus, weakly similar to NP_001051696.1 [Oryza sativa (japonica cultivar-group)]
LesAffx.63489.1.S1_at	BI921137	TC22082	555	1110	2146	2	3.87	Transcribed locus, weakly similar to XP_001117063.1 transmembrane BAX inhibitor motif containing 4 isoform 1 [Macaca mulatta]
			2	4	6	2	3.15	---
Les.5916.1.S1_at	AJ715788.1	TC23545	748	1494	1528	2	2.04	anaerobic basic leucine zipper protein
Les.5177.1.S1_at	BT013446.1	TC24190	28	25	254	0.91	9.21	Lycopersicon esculentum clone 132101F, mRNA sequence
Les.5442.1.S1_at	BT013931.1	TC22814	266	514	1981	1.94	7.46	Lycopersicon esculentum clone 132929R, mRNA sequence
Les.129.1.S1_at	AF317515.1	TC21823	122	239	864	1.96	7.06	divinyl ether synthase
LesAffx.52594.1.S1_at	AI895802	TC22372	40	75	256	1.86	6.34	Transcribed locus, weakly similar to NP_001047179.1 [Oryza sativa (japonica cultivar-group)]
LesAffx.3455.1.S1_at	BI421254	TC23177	149	262	697	1.76	4.67	Transcribed locus, weakly similar to NP_001042297.1 [Oryza sativa (japonica cultivar-group)]
LesAffx.3455.2.S1_at	CN385030	TC23177	307	524	1393	1.71	4.53	Transcribed locus, weakly similar to NP_001042297.1 [Oryza sativa (japonica cultivar-group)]
Les.54.1.S1_at	AW218809	TC22203	170	297	747	1.75	4.4	PR-1a1 protein
LesAffx.26180.1.S1_at	BG132890	BG13289	159	295	660	1.86	4.15	---
Les.4317.1.S1_at	AW625684	TC23953	1266	2194	5165	1.73	4.08	asparagine synthetase
LesAffx.59842.1.S1_at	BI205718	TC21823	45	34	174	0.75	3.89	Transcribed locus, weakly similar to NP_001042297.1 [Oryza sativa (japonica cultivar-group)]
Les.5934.1.S1_at	AI895164	TC21861	264	406	1020	1.53	3.86	Transcribed locus, weakly similar to NP_001047927.1 [Oryza sativa (japonica cultivar-group)]
Les.3735.1.S1_at	AY007561.1	TC22594	338	476	1271	1.41	3.76	hypothetical LOC543816
LesAffx.33082.1.S1_at	BG133383	TC23204	25	37	92	1.5	3.73	---
Les.2591.1.S1_at	M80608.1		2433	3930	9023	1.62	3.71	beta-1,3-glucanase
Les.1350.1.A1_at	BG631017	TC24319	93	82	342	0.88	3.68	Transcribed locus

LesAffx.8720.1.S1_at	BI921446	BI92223	214	406	742	1.9	3.47	Transcribed locus, weakly similar to NP_663449.2 P450-like [Mus musculus]
LesAffx.3455.2.A1_at	CN385030	TC23177	43	58	149	1.34	3.47	Transcribed locus, weakly similar to NP_001042297.1 [Oryza sativa (japonica cultivar-group)]
Les.1.1.S1_at	AF154421.1	TC21715	1857	2580	6436	1.39	3.47	beta-galactosidase
Les.4693.1.S1_at	M69247.1	TC23231	600	952	2061	1.59	3.44	pathogenesis-related protein P4
LesAffx.8720.2.S1_at	AI895030	TC22019	371	690	1229	1.86	3.31	Transcribed locus, weakly similar to NP_663449.2 P450-like [Mus musculus]
Les.5917.1.S1_at	AJ715790.1	TC21740	1327	2449	4228	1.85	3.19	1-aminocyclopropane-1-carboxylate oxidase
Les.1741.1.A1_at	BG628577	TC24082	2	4	6	1.87	3.16	Transcribed locus, weakly similar to NP_001042587.1 [Oryza sativa (japonica cultivar-group)]
LesAffx.43198.1.S1_at	BF113993	BF11399	163	295	511	1.81	3.13	Transcribed locus, weakly similar to NP_001045064.1 [Oryza sativa (japonica cultivar-group)]
Les.3707.1.S1_at	AF022013.1	TC24298	56	29	170	0.51	3.04	IAA2 protein
LesAffx.59592.1.S1_at	BI210118	TC22019	21	12	64	0.56	3.03	Transcribed locus, weakly similar to NP_065956.1 dehydrogenase 14 (all-trans and 9-cis) [Homo sapiens]
LesAffx.59622.1.S1_at	BI210866	TC22863	341	406	1018	1.19	2.99	Transcribed locus, weakly similar to NP_565922.1 (EARLY FLOWERING 4) [Arabidopsis thaliana]
LesAffx.68092.1.S1_at	BE459012	TC22815	176	218	523	1.23	2.96	Transcribed locus, weakly similar to NP_001044783.1 [Oryza sativa (japonica cultivar-group)]
Les.5884.1.S1_at	CK716273	TC21763	1065	1944	3134	1.83	2.94	Transcribed locus, weakly similar to NP_173228.1 inhibitor [Arabidopsis thaliana]
Les.3491.1.S1_at	AF077339.1	TC23081	118	159	346	1.35	2.94	endo-1,4-beta-glucanase
Les.3042.2.S1_at	AW934591	BE46335	34	62	97	1.85	2.88	Transcribed locus, weakly similar to NP_001062055.1 [Oryza sativa (japonica cultivar-group)]
Les.3683.1.S1_at	AY093595.1	TC24086	716	1335	2054	1.86	2.87	PR-5x
Les.3625.1.S1_at	L22188.1	TC23715	289	200	785	0.69	2.72	RSI-1 protein
Les.3706.1.S1_at	AF022014.1	TC21953	228	179	620	0.79	2.72	IAA3 protein
Les.3575.1.S1_at	U89256.1	TC22509	99	156	267	1.57	2.7	Pti5
Les.3583.1.A1_at	Y15846.1	TC22445	210	216	562	1.03	2.67	TSI-1 protein
LesAffx.17387.1.S1_at	BM534931	BM53493	379	597	1012	1.58	2.67	---
LesAffx.22100.1.S1_at	AW216933	TC22078	155	267	410	1.73	2.65	Transcribed locus, weakly similar to XP_001061027.1 similar to Heat shock factor protein 1 (HSF 1) (Heat shock transcription factor 1) (HSTF 1) [Rattus norvegicus]
LesAffx.24042.1.S1_at	CN385197	DB67876	696	1089	1832	1.56	2.63	Transcribed locus, weakly similar to NP_663449.2 P450-like [Mus musculus]
LesAffx.59625.1.S1_at	BI204607	TC22866	184	172	484	0.94	2.63	Transcribed locus, weakly similar to NP_001049721.1 [Oryza sativa (japonica cultivar-group)]
Les.5597.1.S1_at	BT014226.1	AW09450	46	40	120	0.87	2.63	Lycopersicon esculentum clone 133421F, mRNA sequence
LesAffx.32607.1.S1_at	BI206284	TC23179	13	19	34	1.45	2.62	---

LesAffx.56802.1.S1_at	BI423210	TC21932	859	1572	2248	1.83	2.62	Transcribed locus, weakly similar to NP_001044900.1 [Oryza sativa (japonica cultivar-group)]
Les.3669.1.S1_at	L19762.1	TC21717	229	290	589	1.27	2.57	sucrose synthase
LesAffx.3253.1.S1_at	BM412445	TC21903	123	144	315	1.17	2.56	Transcribed locus, weakly similar to XP_001091435.1 similar to cytochrome P450, family 26, subfamily A, polypeptide 1 isoform 1 isoform 3 [Macaca mulatta]
LesAffx.59769.1.S1_at	BI203677	TC23621	798	1439	2042	1.8	2.56	---
LesAffx.52595.1.S1_at	AI778688	TC22841	478	897	1218	1.88	2.55	Transcribed locus, weakly similar to XP_001103792.1 ERO1-like isoform 1 [Macaca mulatta]
Les.3408.1.S2_at	Y08804.1	TC21800	37	48	92	1.31	2.5	PR protein
LesAffx.3554.1.A1_at	CK720570	CK72057	19	19	47	1.02	2.49	---
Les.3396.2.S1_at	AW221386	TC22284	1822	3009	4514	1.65	2.48	Transcribed locus, weakly similar to NP_001042402.1 [Oryza sativa (japonica cultivar-group)]
LesAffx.31514.1.S1_at	BG127453	TC22656	795	1155	1954	1.45	2.46	Transcribed locus, weakly similar to NP_001041856.1 [Oryza sativa (japonica cultivar-group)]
Les.3465.1.S1_at	AY079426.1	TC21718	189	357	463	1.89	2.46	ethylene receptor-like protein
LesAffx.44395.1.S1_at	BM409907	TC22503	757	637	1856	0.84	2.45	CDNA, clone: FC04BC05, HTC in fruit
Les.63.1.S1_at	AF049900.1	TC22864	20	19	50	0.93	2.43	gibberellin 20-oxidase-3
Les.3591.1.S1_at	CN385613	FS19591	517	674	1250	1.31	2.42	Partial mRNA (clone SENU1)
Les.3706.1.A1_at	AF022014.1	TC21953	107	69	258	0.64	2.41	IAA3 protein
Les.4346.1.S1_at	AF020390.2	TC21717	606	689	1442	1.14	2.38	ss-galactosidase
Les.4718.1.S1_at	BT013487.1	BI92390	124	79	295	0.63	2.37	Lycopersicon esculentum clone 132171F, mRNA sequence
LesAffx.15953.1.S1_at	AW033344	TC23158	77	84	181	1.09	2.36	Transcribed locus, weakly similar to NP_181965.1 protein [Arabidopsis thaliana]
Les.4966.1.S1_at	BT013015.1	BF17648	144	127	339	0.88	2.35	Lycopersicon esculentum clone 114253R, mRNA sequence
Les.3700.1.S1_at	AY026343.1	TC22149	1055	1090	2478	1.03	2.35	Lycopersicon esculentum non-symbiotic hemoglobin class 1 (Glb1)
Les.4888.1.S1_at	BI927238	TC24096	532	903	1245	1.7	2.34	Lycopersicon esculentum clone 113905R, mRNA sequence
LesAffx.37916.1.S1_at	BM536108	TC23806	90	125	209	1.39	2.33	Transcribed locus, weakly similar to NP_001048479.1 [Oryza sativa (japonica cultivar-group)]
Les.3707.1.A1_at	AF022013.1	TC24298	29	22	66	0.76	2.28	IAA2 protein
Les.3597.1.S1_at	S40549.1	TC22411	637	639	1454	1	2.28	deoxyuridine triphosphatase
Les.3673.1.S1_at	M80604.1		851	1187	1936	1.39	2.27	beta-1,3-glucanase
LesAffx.64333.1.S1_at	CN384866	TC21750	2976	2409	6744	0.81	2.27	Transcribed locus, weakly similar to NP_565383.1 protein [Arabidopsis thaliana]
LesAffx.62690.1.S1_at	BI206793	TC22770	449	764	1004	1.7	2.24	Transcribed locus, weakly similar to NP_001043439.1 [Oryza sativa (japonica cultivar-group)]
LesAffx.62570.2.S1_at	CD003040	TC24016	189	370	421	1.96	2.23	Transcribed locus, weakly similar to NP_001044685.1 [Oryza sativa (japonica cultivar-group)]

LesAffx.62690.2.S1_at	CD003021	CD00302	882	1447	1953	1.64	2.21	Transcribed locus, weakly similar to NP_001043439.1 [Oryza sativa (japonica cultivar-group)]
Les.3663.1.S1_at	X55193.1	TC22989	258	193	569	0.75	2.2	9612 protein
Les.909.1.A1_at	BG628078	TC23979	14	19	31	1.37	2.2	Transcribed locus
Les.5339.1.S1_at	BT013735.1	TC23231	658	947	1434	1.44	2.18	Lycopersicon esculentum clone 132595F, mRNA sequence
Les.1568.1.A1_at	BG626849	TC24275	18	28	40	1.51	2.18	Transcribed locus
Les.977.1.A1_at	BG628563	TC24335	3	6	7	1.84	2.17	Transcribed locus
LesAffx.68294.1.S1_at	BM408981	TC22580	279	162	603	0.58	2.17	Transcribed locus, weakly similar to NP_001046934.1 [Oryza sativa (japonica cultivar-group)]
Les.5855.1.S1_at	BT014384.1	TC22983	1306	2416	2823	1.85	2.16	Lycopersicon esculentum clone 133681F, mRNA sequence
Les.2026.1.S1_at	BF051105	TC22543	616	893	1326	1.45	2.15	Transcribed locus, weakly similar to NP_001047542.1 [Oryza sativa (japonica cultivar-group)]
LesAffx.44422.1.S1_at	AW441968	TC21789	388	634	831	1.63	2.14	Transcribed locus, weakly similar to NP_001041874.1 [Oryza sativa (japonica cultivar-group)]
LesAffx.3059.1.S1_at	AI776626	AI77662	25	48	52	1.94	2.13	---
LesAffx.15986.1.S1_at	CK715617	TC23538	67	107	142	1.61	2.13	Transcribed locus, weakly similar to XP_001116334.1 myo-inositol oxygenase [Macaca mulatta]
Les.1606.1.S1_at	BG627189	TC23013	1498	1601	3188	1.07	2.13	Lycopersicon esculentum clone 114287R, mRNA sequence
Les.2643.1.S1_at	BG128842	TC23690	238	284	503	1.2	2.12	Transcribed locus
LesAffx.1695.1.S1_at	BE435747	TC21794	101	115	210	1.14	2.09	Transcribed locus, weakly similar to NP_001042242.1 [Oryza sativa (japonica cultivar-group)]
LesAffx.735.1.S1_at	AW217158	TC22685	232	451	485	1.94	2.09	Transcribed locus, weakly similar to NP_001042221.1 [Oryza sativa (japonica cultivar-group)]
LesAffx.3554.1.S1_at	CK720570	CK72057	16	20	33	1.26	2.09	---
Les.288.1.S1_at	BM410927	TC23438	63	91	132	1.44	2.08	NAC domain protein
LesAffx.52960.1.S1_at	BI207845	TC23617	286	460	593	1.61	2.07	---
LesAffx.24696.1.S1_at	AW034874	DB70637	105	55	216	0.52	2.05	Transcribed locus, weakly similar to NP_001043378.1 [Oryza sativa (japonica cultivar-group)]
LesAffx.56137.1.A1_at	AI490099	TC22599	47	55	97	1.15	2.04	Transcribed locus, weakly similar to NP_001041909.1 [Oryza sativa (japonica cultivar-group)]
LesAffx.18686.1.S1_at	BI203812	TC22685	52	77	107	1.48	2.04	Transcribed locus, weakly similar to NP_001043492.1 [Oryza sativa (japonica cultivar-group)]
LesAffx.62690.2.A1_at	CD003021	CD00302	263	406	534	1.54	2.03	Transcribed locus, weakly similar to NP_001043439.1 [Oryza sativa (japonica cultivar-group)]
Les.220.1.S1_at	CK716059	TC21926	2468	3955	5008	1.6	2.03	Transcribed locus, weakly similar to NP_001055699.1 [Oryza sativa (japonica cultivar-group)]

LesAffx.69783.1.S1_at	AW220917	TC23448	1140	2235	2307	1.96	2.02	Transcribed locus, weakly similar to NP_001067067.1 [Oryza sativa (japonica cultivar-group)]
Les.797.1.S1_at	X92854.1	TC24488	721	1070	1454	1.48	2.02	Exon 1 of AMT1 gene
Les.5567.1.S1_at	BT014164.1	TC22700	629	558	1263	0.89	2.01	Lycopersicon esculentum clone 133286F, mRNA sequence
LesAffx.60369.1.S1_at	BG130091	TC22835	1091	1444	2189	1.32	2.01	Transcribed locus, moderately similar to NP_001064827.1 [Oryza sativa (japonica cultivar-group)]
Les.2137.1.S1_at	AY359965.1	TC21714	1033	1480	2072	1.43	2.01	EIX receptor 1
LesAffx.38395.2.S1_at	AI488594	AI48859	197	250	395	1.27	2	---
Les.2832.1.S1_at	CN384480	TC21748	1542	1268	3086	0.82	2	peroxidase
Les.4493.1.S1_at	BG734807	TC22690	3276	2959	1653	0.9	0.5	ADP/ATP translocator
Les.4488.1.S1_at	BG628643	TC22827	6203	5929	3128	0.96	0.5	threonine deaminase
Les.5493.1.S1_at	BT014016.1	AW62178	420	408	211	0.97	0.5	Lycopersicon esculentum clone 133078F, mRNA sequence
Les.2024.2.S1_at	BG642995	AI48435	1207	914	607	0.76	0.5	Transcribed locus, weakly similar to XP_001105201.1 fructose-1,6-bisphosphatase 1 isoform 1 [Macaca mulatta]
LesAffx.68320.1.S1_at	BM535633	ES89343	1170	1234	588	1.05	0.5	Transcribed locus, weakly similar to NP_001065587.1 [Oryza sativa (japonica cultivar-group)]
LesAffx.16884.1.S1_at	BI421819	TC21771	7226	6474	3632	0.9	0.5	Metallothionein-like protein
Les.3085.1.S1_at	BM413323	TC22204	1583	1529	793	0.97	0.5	Transcribed locus, weakly similar to NP_001042186.1 [Oryza sativa (japonica cultivar-group)]
Les.1603.1.A1_at	BG630194	TC23278	213	147	107	0.69	0.5	Transcribed locus, weakly similar to NP_001043493.1 [Oryza sativa (japonica cultivar-group)]
Les.3409.1.A1_at	BG735108	GO37597	4196	2804	2095	0.67	0.5	Transcribed locus, weakly similar to NP_001044764.1 [Oryza sativa (japonica cultivar-group)]
Les.3594.1.S1_at	Z47980.1	TC21807	3702	2322	1842	0.63	0.5	hypothetical LOC544160
LesAffx.3394.1.S1_at	BT013543.1	TC23598	2019	1303	1002	0.65	0.5	---
LesAffx.66600.1.S1_at	AW040689	TC23173	1918	1709	952	0.89	0.5	Transcribed locus, moderately similar to NP_001045167.1 [Oryza sativa (japonica cultivar-group)]
Les.4459.1.S1_s_at	X99148.1	ES89373	2038	1245	1010	0.61	0.5	hypothetical LOC544160 /// arabinogalactan
Les.3983.1.S1_at	U20591.1	TC23477	65	50	32	0.77	0.49	flower-specific gamma-thionin-like protein/acidic protein precursor
LesAffx.53966.1.S1_at	AY026037.1	TC23466	2000	1783	987	0.89	0.49	---
Les.3550.1.S1_at	AF096251.1	TC24195	363	361	179	0.99	0.49	ethylene-responsive heat shock protein cognate 70
Les.3175.1.S1_at	BG626608	TC23958	3901	2879	1908	0.74	0.49	Lycopersicon esculentum clone 132976F, mRNA sequence
LesAffx.29801.1.S1_at	AI489456	TC23187	4574	5597	2234	1.22	0.49	Transcribed locus, weakly similar to NP_001042186.1 [Oryza sativa (japonica cultivar-group)]
Les.608.1.S1_at	BG628276	TC21796	6221	5262	3033	0.85	0.49	Lycopersicon esculentum clone 133776F, mRNA sequence
Les.3123.1.S1_at	BT013406.1	TC23016	3147	2078	1531	0.66	0.49	Lycopersicon esculentum clone 135721R, mRNA sequence

LesAffx.55629.1.S1_at	BM412122	TC22287	391	451	190	1.15	0.49	Transcribed locus, weakly similar to NP_001044052.1 [Oryza sativa (japonica cultivar-group)]
Les.2060.1.A1_at	BG631361	BG63136	7	9	3	1.37	0.49	Transcribed locus, weakly similar to NP_001054945.1 [Oryza sativa (japonica cultivar-group)]
LesAffx.62950.1.S1_at	AI490601	TC23296	549	577	266	1.05	0.48	Transcribed locus, weakly similar to XP_001104086.1 similar to UDP-galactose-4-epimerase isoform 2 [Macaca mulatta]
Les.3593.1.S1_at	BT012763.1	TC23407	3718	3180	1800	0.86	0.48	TAS14 peptide (AA 1-130)
LesAffx.65029.1.S1_at	AJ785063	TC22116	1483	1465	718	0.99	0.48	Transcribed locus, weakly similar to NP_001058029.1 [Oryza sativa (japonica cultivar-group)]
Les.2620.1.S1_at	BG631075	AW44264	1185	965	573	0.81	0.48	Transcribed locus, weakly similar to NP_001044402.1 [Oryza sativa (japonica cultivar-group)]
Les.3180.2.S1_at	AI775197	TC22419	367	267	177	0.73	0.48	Transcribed locus, weakly similar to NP_001048006.1 [Oryza sativa (japonica cultivar-group)]
Les.3664.1.S1_at	U20590.1	TC22651	965	583	464	0.6	0.48	endo-1,4-beta-glucanase precursor
LesAffx.64062.1.S1_at	AW040768	TC22080	114	84	55	0.73	0.48	Transcribed locus, weakly similar to NP_001042589.1 [Oryza sativa (japonica cultivar-group)]
LesAffx.30938.1.S1_at	AJ785088	TC22113	430	259	204	0.6	0.47	Transcribed locus, weakly similar to NP_001042177.1 [Oryza sativa (japonica cultivar-group)]
Les.2909.1.S1_at	AW223174	TC24474	2329	1636	1106	0.7	0.47	Transcribed locus, weakly similar to NP_001041799.1 [Oryza sativa (japonica cultivar-group)]
LesAffx.64062.2.S1_at	BG129043	TC22887	717	793	340	1.11	0.47	Transcribed locus, moderately similar to NP_001044143.1 [Oryza sativa (japonica cultivar-group)]
Les.641.1.S1_at	BG626664	TC23339	1329	689	630	0.52	0.47	Lycopersicon esculentum clone 132316F, mRNA sequence
Les.5096.1.S1_at	BT013271.1	TC22616	159	316	75	1.99	0.47	Lycopersicon esculentum clone 134844R, mRNA sequence
Les.513.1.S1_at	BG626001	TC22866	374	240	177	0.64	0.47	Lycopersicon esculentum clone 132360F, mRNA sequence
LesAffx.66331.1.S1_at	BI421673	TC21932	196	222	93	1.13	0.47	Transcribed locus, weakly similar to NP_001056463.1 [Oryza sativa (japonica cultivar-group)]
Les.1596.1.A1_at	BG627098	TC23373	2237	2269	1054	1.01	0.47	Transcribed locus, moderately similar to NP_001051136.1 [Oryza sativa (japonica cultivar-group)]
Les.3140.1.S1_at	BG628034	TC24291	2856	2987	1341	1.05	0.47	Transcribed locus, weakly similar to XP_001088834.1 acetyl-Coenzyme A acyltransferase 1 isoform 2 [Macaca mulatta]
Les.3234.1.A1_at	BG735047	TC23423	4021	4469	1887	1.11	0.47	Metallocarboxypeptidase inhibitor
LesAffx.30938.1.A1_at	AJ785088	TC22113	186	114	87	0.61	0.47	Transcribed locus, weakly similar to NP_001042177.1 [Oryza sativa (japonica cultivar-group)]
LesAffx.65029.1.A1_at	AJ785063	TC22116	576	482	270	0.84	0.47	Transcribed locus, weakly similar to NP_001058029.1 [Oryza sativa (japonica cultivar-group)]

LesAffx.66600.1.A1_at	AW036163	TC23173	870	722	406	0.83	0.47	Transcribed locus, moderately similar to NP_001045167.1 [Oryza sativa (japonica cultivar-group)]
Les.4944.1.S1_at	BT012955.1	TC22653	130	82	61	0.63	0.47	Lycopersicon esculentum clone 114142R, mRNA sequence
Les.3062.2.S1_at	AI782456	TC23728	717	503	334	0.7	0.47	Transcribed locus, weakly similar to NP_001043493.1 [Oryza sativa (japonica cultivar-group)]
LesAffx.41703.1.S1_at	BG352060	TC23987	267	246	124	0.92	0.47	Transcribed locus, moderately similar to NP_001062462.1 [Oryza sativa (japonica cultivar-group)]
LesAffx.38499.1.A1_at	BI209699	TC22324	572	331	266	0.58	0.47	Transcribed locus, moderately similar to NP_001044017.1 [Oryza sativa (japonica cultivar-group)]
Les.2418.1.S1_at	BG735381	BG73538	357	374	166	1.05	0.46	Transcribed locus
Les.2703.1.S1_at	BG735318	TC23293	2018	1114	936	0.55	0.46	Lycopersicon esculentum clone 132109R, mRNA sequence
Les.4849.1.S1_at	BT012755.1	TC21877	1190	1118	550	0.94	0.46	Lycopersicon esculentum clone 113699R, mRNA sequence
LesAffx.46085.1.S1_at	AW928724	TC22038	276	147	127	0.53	0.46	Transcribed locus, weakly similar to XP_001114240.1 cytochrome P450, family 21, subfamily A, polypeptide 2 isoform 3 [Macaca mulatta]
Les.490.1.A1_at	BG625891	TC22945	315	252	145	0.8	0.46	Transcribed locus, weakly similar to XP_001108145.1 similar to nucleotide binding protein-like [Macaca mulatta]
Les.5437.1.S1_at	CK715969	TC24035	1259	849	579	0.67	0.46	Lycopersicon esculentum clone 132916F, mRNA sequence
LesAffx.41333.2.S1_at	AW934014	TC23167	295	266	136	0.9	0.46	Transcribed locus, weakly similar to XP_001111662.1 protein phosphatase 1B isoform 6 [Macaca mulatta]
LesAffx.41703.1.A1_at	BG352060	TC23987	1183	1239	543	1.05	0.46	Transcribed locus, moderately similar to NP_001062462.1 [Oryza sativa (japonica cultivar-group)]
Les.4492.1.S1_at	BG628012	TC21878	674	488	309	0.72	0.46	Transcribed locus, weakly similar to XP_001111095.1 aldolase B isoform 5 [Macaca mulatta]
Les.2934.2.S1_at	BG128445	TC22376	399	338	183	0.85	0.46	Transcribed locus, weakly similar to NP_001046132.1 [Oryza sativa (japonica cultivar-group)]
Les.3266.3.S1_at	BI935106	TC22577	1982	2079	904	1.05	0.46	Transcribed locus, moderately similar to NP_001047794.1 [Oryza sativa (japonica cultivar-group)]
LesAffx.16291.2.S1_at	AW040554	TC24065	540	473	245	0.88	0.45	Transcribed locus, moderately similar to NP_001050499.1 [Oryza sativa (japonica cultivar-group)]
Les.3180.3.A1_at	BG734557	TC23323	1277	941	580	0.74	0.45	Transcribed locus, weakly similar to NP_001048006.1 [Oryza sativa (japonica cultivar-group)]
Les.3372.1.S1_at	BG631141	TC23249	2066	1064	935	0.52	0.45	Lycopersicon esculentum clone 132813R, mRNA sequence
Les.3924.1.S1_at	BE433308	TC23702	5683	5622	2559	0.99	0.45	Hypothetical protein LOC778199
LesAffx.8708.1.S1_at	AW033860	AW03386	82	54	37	0.65	0.45	---
Les.314.1.A1_at	CK715040	TC22072	1712	1137	769	0.66	0.45	Transcribed locus, weakly similar to XP_001084411.1 aquaporin 1 [Macaca mulatta]
LesAffx.23510.1.S1_at	AI772286	TC23430	102	82	46	0.8	0.45	Transcribed locus

Les.4492.2.S1_at	CK714954	TC22356	4903	3832	2196	0.78	0.45	Transcribed locus, weakly similar to XP_001111095.1 aldolase B isoform 5 [Macaca mulatta]
Les.3210.2.A1_at	AJ320060	TC22662	1665	1737	742	1.04	0.45	Transcribed locus
Les.3113.1.S1_at	BG131753	TC21889	329	315	146	0.96	0.44	Transcribed locus, weakly similar to XP_001101334.1 sterol-C4-methyl oxidase-like isoform 1 [Macaca mulatta]
LesAffx.26471.2.S1_at	AW218197	TC22883	36	20	16	0.55	0.44	Transcribed locus, weakly similar to NP_001054088.1 [Oryza sativa (japonica cultivar-group)]
Les.2964.2.S1_at	BI923596	TC22145	308	350	136	1.13	0.44	Transcribed locus, weakly similar to NP_001042324.1 [Oryza sativa (japonica cultivar-group)]
Les.3119.1.A1_at	AW037265	TC23253	585	631	259	1.08	0.44	Transcribed locus, weakly similar to XP_001094505.1 proteasome 26S non-ATPase subunit 3 isoform 4 [Macaca mulatta]
Les.1123.1.A1_at	BG629518	BG62951	50	34	22	0.69	0.44	Transcribed locus
Les.4259.1.S1_at	BG629037	TC24424	4419	3194	1954	0.72	0.44	Transcribed locus, weakly similar to XP_001082599.1 cysteine and glycine-rich protein 2 [Macaca mulatta]
Les.672.1.S1_at	BM536270	TC22195	695	498	306	0.72	0.44	Transcribed locus, weakly similar to NP_001042227.1 [Oryza sativa (japonica cultivar-group)]
Les.2809.1.S1_at	BT014540.1	TC24080	956	870	417	0.91	0.44	Lycopersicon esculentum clone 133955F, mRNA sequence
LesAffx.18330.1.S1_at	BG129699	TC24342	789	641	344	0.81	0.44	Transcribed locus, weakly similar to NP_001046074.1 [Oryza sativa (japonica cultivar-group)]
Les.70.1.S1_at	AF000141.1	NP00014	1980	1011	857	0.51	0.43	class I knotted-like homeodomain protein
Les.3210.1.S1_at	AI483637	TC22662	1167	1308	503	1.12	0.43	Transcribed locus
Les.1157.1.A1_at	BG629717	BG62971	101	83	43	0.82	0.43	Transcribed locus
LesAffx.29801.1.A1_at	AI489456	TC23187	302	310	129	1.03	0.43	Transcribed locus, weakly similar to NP_001042186.1 [Oryza sativa (japonica cultivar-group)]
Les.3330.3.A1_at	BG629064	TC24355	3175	1761	1357	0.55	0.43	Transcribed locus, weakly similar to NP_001044764.1 [Oryza sativa (japonica cultivar-group)]
Les.3409.2.S1_at	BF050508	TC21920	2577	1466	1099	0.57	0.43	Transcribed locus, weakly similar to NP_001044764.1 [Oryza sativa (japonica cultivar-group)]
Les.3166.1.S1_at	AI775188	TC23957	129	124	55	0.96	0.42	Transcribed locus, weakly similar to NP_001047732.1 [Oryza sativa (japonica cultivar-group)]
Les.110.1.S1_at	AF154423.1	TC22330	1588	871	673	0.55	0.42	TBG5 protein
LesAffx.64540.1.S1_at	AW441832	TC22052	1279	1349	541	1.05	0.42	Transcribed locus, moderately similar to NP_001053805.1 [Oryza sativa (japonica cultivar-group)]
LesAffx.21101.1.S1_at	BI924446	TC21935	153	108	64	0.71	0.42	Transcribed locus, weakly similar to NP_001047732.1 [Oryza sativa (japonica cultivar-group)]
Les.3741.1.S1_at	AF146691.1	TC23195	1940	2543	815	1.31	0.42	Eli3 protein

Les.3113.2.S1_at	AI780108	FS20183	295	271	123	0.92	0.42	Transcribed locus, weakly similar to XP_001101334.1 sterol-C4-methyl oxidase-like isoform 1 [Macaca mulatta]
Les.3741.1.A1_at	AF146691.1	TC23195	816	936	339	1.15	0.42	Eli3 protein
Les.1923.1.S1_at	BG630193	TC23497	334	268	138	0.8	0.41	Transcribed locus, moderately similar to NP_001066340.1 [Oryza sativa (japonica cultivar-group)]
Les.3119.3.S1_at	AW092353	TC23253	687	753	284	1.1	0.41	Transcribed locus, weakly similar to XP_001094505.1 proteasome 26S non-ATPase subunit 3 isoform 4 [Macaca mulatta]
Les.1936.1.S1_at	BG626997	TC21753	1899	2626	783	1.38	0.41	Lycopersicon esculentum clone 133556R, mRNA sequence
LesAffx.14110.2.S1_at	AW932597	TC23949	1232	806	507	0.65	0.41	Transcribed locus, weakly similar to NP_001042660.1 [Oryza sativa (japonica cultivar-group)]
Les.3119.2.S1_at	BI927792	TC23253	389	413	160	1.06	0.41	Transcribed locus, weakly similar to XP_001094505.1 proteasome 26S non-ATPase subunit 3 isoform 4 [Macaca mulatta]
Les.3330.2.S1_at	BE458823	TC24355	3030	1675	1239	0.55	0.41	Transcribed locus, weakly similar to NP_001044764.1 [Oryza sativa (japonica cultivar-group)]
LesAffx.14110.2.A1_at	AW932597	TC23949	197	111	80	0.56	0.41	Transcribed locus, weakly similar to NP_001042660.1 [Oryza sativa (japonica cultivar-group)]
Les.2415.2.S1_at	BE431455	TC23842	1246	875	508	0.7	0.41	Transcribed locus, moderately similar to NP_001061906.1 [Oryza sativa (japonica cultivar-group)]
Les.424.2.S1_at	AI776305	TC22847	2404	2043	979	0.85	0.41	Transcribed locus, weakly similar to NP_001041744.1 [Oryza sativa (japonica cultivar-group)]
LesAffx.37588.1.S1_at	BG124246	TC21902	84	76	34	0.9	0.41	Transcribed locus, weakly similar to NP_001046132.1 [Oryza sativa (japonica cultivar-group)]
Les.1478.1.S1_at	BG631500	TC23304	1377	1168	559	0.85	0.41	Lycopersicon esculentum clone 114252R, mRNA sequence
Les.3668.1.S1_at	U09026.1	TC21838	963	763	391	0.79	0.41	lipoxygenase
Les.2131.2.S1_at	BE433043	TC22249	2227	2245	902	1.01	0.4	Transcribed locus, weakly similar to NP_001050262.1 [Oryza sativa (japonica cultivar-group)]
Les.4412.1.A1_at	BG627732	TC23503	1188	776	481	0.65	0.4	Transcribed locus, weakly similar to NP_001042986.1 [Oryza sativa (japonica cultivar-group)]
Les.1784.1.S1_at	BI931777	TC24140	2189	1361	884	0.62	0.4	Transcribed locus, weakly similar to XP_001118335.1 similar to intestinal facilitative glucose transporter 7 [Macaca mulatta]
LesAffx.21904.1.S1_at	BM536146	TC23302	340	262	137	0.77	0.4	Transcribed locus, weakly similar to NP_001048363.1 [Oryza sativa (japonica cultivar-group)]
LesAffx.34986.1.A1_at	CK715664	TC23304	1182	925	476	0.78	0.4	Transcribed locus, weakly similar to NP_001048569.1 [Oryza sativa (japonica cultivar-group)]
Les.424.1.S1_at	BG628349	TC22847	2534	2327	1017	0.92	0.4	Transcribed locus, weakly similar to NP_001041744.1 [Oryza sativa (japonica cultivar-group)]

Les.5864.1.S1_at	U20596.1	TC23921	7593	6045	3047	0.8	0.4	hypothetical LOC544002
Les.4008.1.S1_a_at	BG630151	TC21741	5134	3170	2042	0.62	0.4	xyloglucan endotransglycosylase/hydrolase 16 protein
Les.3995.1.S1_at	AW443014	TC21771	3091	2024	1226	0.65	0.4	methionine rich arabinogalactan
Les.2038.1.A1_at	BG631023	TC23348	889	741	351	0.83	0.4	Transcribed locus, weakly similar to NP_199407.1 ester hydrolase/ hydrolase, acting on ester bonds [Arabidopsis thaliana]
LesAffx.81.2.S1_at	AW648752	TC22482	3290	2900	1300	0.88	0.39	Transcribed locus, weakly similar to XP_001090175.1 hypothetical protein LOC51507 isoform 2 [Macaca mulatta]
Les.2415.3.A1_at	BG735370	TC23842	1780	1341	701	0.75	0.39	Transcribed locus, moderately similar to NP_001061906.1 [Oryza sativa (japonica cultivar-group)]
LesAffx.20929.1.S1_at	BI206813	TC23621	308	207	121	0.67	0.39	Transcribed locus, weakly similar to NP_001051650.1 [Oryza sativa (japonica cultivar-group)]
Les.4359.1.S1_at	BE462878	TC23326	427	273	167	0.64	0.39	Pathogenesis-related protein P2
Les.2063.1.A1_at	BG631371	TC24473	63	47	24	0.75	0.39	Transcribed locus
Les.3330.1.S1_at	AW650121	TC24355	1650	937	644	0.57	0.39	Transcribed locus, weakly similar to NP_001044764.1 [Oryza sativa (japonica cultivar-group)]
Les.314.2.S1_at	AW217816	BG64268	1773	1073	690	0.61	0.39	Transcribed locus, weakly similar to XP_001084411.1 aquaporin 1 [Macaca mulatta]
Les.861.1.A1_at	BG627791	TC23388	229	238	89	1.04	0.39	Transcribed locus, moderately similar to NP_001065502.1 [Oryza sativa (japonica cultivar-group)]
Les.349.1.S1_at	CN385641	TC23560	279	259	108	0.93	0.39	Transcribed locus
Les.953.2.A1_at	BG628368	TC22566	18	19	7	1.03	0.39	Transcribed locus, weakly similar to XP_001113968.1 similar to solute carrier family 35, member E1 [Macaca mulatta]
Les.790.1.A1_at	BG627458	TC23127	241	163	93	0.68	0.39	Transcribed locus, weakly similar to NP_001052896.1 [Oryza sativa (japonica cultivar-group)]
Les.4835.1.S1_at	BT012722.1	TC24498	444	255	171	0.58	0.39	Lycopersicon esculentum clone 113649F, mRNA sequence
Les.3273.1.S1_at	BG627786	TC24518	918	1171	353	1.28	0.39	proline rich protein
Les.2131.1.A1_at	BG627099	TC22249	2151	2123	825	0.99	0.38	Transcribed locus, weakly similar to NP_001050262.1 [Oryza sativa (japonica cultivar-group)]
Les.3210.2.A1_a_at	AJ320060	TC22662	634	630	243	0.99	0.38	Transcribed locus
Les.2933.1.S1_at	BT012764.1	TC23454	2322	1680	889	0.72	0.38	Lycopersicon esculentum clone 113710R, mRNA sequence
Les.4522.1.S1_at	AY497477.1	TC23773	2638	2835	1009	1.07	0.38	xyloglucan endotransglucosylase-hydrolase XTH6
Les.3073.1.S1_at	AJ785415	TC21760	436	443	166	1.02	0.38	Fruit-ripening protein
Les.3322.2.S1_at	AW091831	TC22662	756	455	288	0.6	0.38	Transcribed locus, moderately similar to NP_001047599.1 [Oryza sativa (japonica cultivar-group)]
Les.4617.1.S1_at	BT014212.1	TC23312	1620	1409	617	0.87	0.38	Lycopersicon esculentum clone 133389F, mRNA sequence
Les.4291.2.S1_at	AW649205	TC22682	3119	2421	1185	0.78	0.38	Transcribed locus, weakly similar to NP_001044127.1 [Oryza sativa (japonica cultivar-group)]

Les.4291.1.S1_at	BI934076	TC22682	2435	1926	921	0.79	0.38	Transcribed locus, weakly similar to NP_001044127.1 [Oryza sativa (japonica cultivar-group)]
Les.2971.2.A1_at	BG629234	TC23191	5983	6106	2236	1.02	0.37	Wound-induced proteinase inhibitor I prepropeptide
Les.375.1.A1_at	BG734771	TC22710	1350	1093	503	0.81	0.37	Transcribed locus, weakly similar to NP_565660.1 protein [Arabidopsis thaliana]
Les.2964.3.A1_at	BG626300	TC23599	998	871	371	0.87	0.37	Transcribed locus, weakly similar to NP_001042324.1 [Oryza sativa (japonica cultivar-group)]
Les.2174.1.S1_at	CN384833	TC22746	175	99	65	0.56	0.37	Transcribed locus
Les.1784.2.A1_at	BG628995	TC24140	223	136	82	0.61	0.37	Transcribed locus, weakly similar to XP_001118335.1 similar to intestinal facilitative glucose transporter 7 [Macaca mulatta]
Les.3742.1.S1_at	AF146690.1	TC22347	1098	681	406	0.62	0.37	Pto-responsive gene 1 protein
Les.4492.3.S1_at	CK715138	TC22356	2764	1890	1021	0.68	0.37	Transcribed locus, weakly similar to XP_001111095.1 aldolase B isoform 5 [Macaca mulatta]
LesAffx.37344.1.S1_at	AI897449	TC21881	700	497	259	0.71	0.37	Transcribed locus, weakly similar to NP_001042390.1 [Oryza sativa (japonica cultivar-group)]
LesAffx.34986.1.S1_at	CK715664	TC23304	576	399	212	0.69	0.37	Transcribed locus, weakly similar to NP_001048569.1 [Oryza sativa (japonica cultivar-group)]
Les.840.1.A1_at	BG629718	TC24411	847	904	310	1.07	0.37	Transcribed locus
LesAffx.70136.2.S1_at	AI899680	TC23538	373	268	136	0.72	0.36	Transcribed locus, weakly similar to NP_001044898.1 [Oryza sativa (japonica cultivar-group)]
Les.5017.1.S1_at	BT013110.1	TC23613	280	256	102	0.91	0.36	Lycopersicon esculentum clone 114397F, mRNA sequence
LesAffx.3537.1.S1_at	AJ784726	TC24068	463	334	168	0.72	0.36	---
Les.1562.1.S1_at	BG629310	TC23006	446	232	161	0.52	0.36	Lycopersicon esculentum clone 133516F, mRNA sequence
LesAffx.3537.1.A1_at	AJ784726	TC24068	2229	1885	799	0.85	0.36	---
Les.1198.1.A1_at	BG630006	TC22356	151	103	54	0.68	0.36	Transcribed locus, weakly similar to XP_001111095.1 aldolase B isoform 5 [Macaca mulatta]
Les.2792.1.A1_at	BG629283	BG62927	390	393	139	1.01	0.36	Transcribed locus
Les.4975.1.S1_at	BT013032.1	TC23130	632	628	224	0.99	0.35	Lycopersicon esculentum clone 114280F, mRNA sequence
Les.5832.1.S1_at	BT014414.1	TC22401	2091	1147	742	0.55	0.35	Lycopersicon esculentum clone 133722R, mRNA sequence
Les.4886.1.S1_at	BT012836.1	BE44961	336	261	119	0.78	0.35	Lycopersicon esculentum clone 113902F, mRNA sequence
Les.4359.2.A1_at	BG630798	TC23326	468	275	166	0.59	0.35	Pathogenesis-related protein P2
Les.799.1.A1_at	BG627521	BG62752	1103	758	387	0.69	0.35	Transcribed locus, weakly similar to NP_001052094.1 [Oryza sativa (japonica cultivar-group)]
LesAffx.37344.2.S1_at	BG131634	TC21881	1341	897	468	0.67	0.35	Transcribed locus, weakly similar to NP_001042390.1 [Oryza sativa (japonica cultivar-group)]
Les.4982.1.S1_at	BT013044.1	AW62120	293	157	102	0.54	0.35	Lycopersicon esculentum clone 114294R, mRNA sequence

LesAffx.57163.3.A1_at	AI486803	TC22802	290	569	101	1.96	0.35	Transcribed locus, weakly similar to NP_001042186.1 [Oryza sativa (japonica cultivar-group)]
LesAffx.69295.1.S1_at	BI933687	TC23040	437	304	151	0.69	0.35	Transcribed locus, weakly similar to NP_001045746.1 [Oryza sativa (japonica cultivar-group)]
Les.4428.2.S1_at	CK575032	TC21729	1975	2231	682	1.13	0.35	carbonic anhydrase
Les.4974.1.S1_at	BT013031.1	TC23280	755	810	261	1.07	0.35	Lycopersicon esculentum clone 114279F, mRNA sequence
Les.810.1.S1_at	BG627575	TC22972	780	556	268	0.71	0.34	Lycopersicon esculentum clone 133744F, mRNA sequence
Les.4312.2.S1_at	BG626083	TC22795	6347	3463	2182	0.55	0.34	CDNA, clone: FC02DB04, HTC in fruit
Les.2173.1.A1_at	BG631444	TC23409	682	654	234	0.96	0.34	Transcribed locus, weakly similar to NP_001044532.1 [Oryza sativa (japonica cultivar-group)]
Les.4345.1.S1_at	BG629758	TC23353	8774	7472	3008	0.85	0.34	Lycopersicon esculentum clone 133385R, mRNA sequence
Les.828.1.A1_at	BG627663	TC24124	298	193	102	0.65	0.34	Transcribed locus
Les.4181.1.A1_at	BG630015	TC22910	149	127	51	0.85	0.34	Transcribed locus
LesAffx.37212.1.S1_at	AI491145	TC23069	633	446	214	0.7	0.34	---
LesAffx.13481.2.S1_at	BI921546	TC22621	1698	1469	566	0.87	0.33	Transcribed locus, weakly similar to NP_001044052.1 [Oryza sativa (japonica cultivar-group)]
Les.3266.2.S1_at	BI931445	TC23669	650	643	216	0.99	0.33	Transcribed locus, moderately similar to NP_001047794.1 [Oryza sativa (japonica cultivar-group)]
LesAffx.13481.2.A1_at	BI921546	TC22621	2836	3094	942	1.09	0.33	Transcribed locus, weakly similar to NP_001044052.1 [Oryza sativa (japonica cultivar-group)]
LesAffx.81.1.S1_at	BG135419	TC23216	1209	1053	400	0.87	0.33	Transcribed locus, weakly similar to XP_001090175.1 hypothetical protein LOC51507 isoform 2 [Macaca mulatta]
Les.5017.1.A1_at	BT013110.1	TC23613	826	848	270	1.03	0.33	Lycopersicon esculentum clone 114397F, mRNA sequence
Les.4008.2.S1_at	BG643663	TC21741	3548	1806	1148	0.51	0.32	xyloglucan endotransglycosylase/hydrolase 16 protein
Les.2457.1.A1_at	BG630077	TC22085	2283	1899	738	0.83	0.32	Transcribed locus
Les.4008.1.S1_at	BG630151	TC21741	3873	2084	1250	0.54	0.32	xyloglucan endotransglycosylase/hydrolase 16 protein
LesAffx.57251.1.S1_at	BI208760	TC22155	249	149	80	0.6	0.32	Transcribed locus, weakly similar to NP_001043814.1 [Oryza sativa (japonica cultivar-group)]
LesAffx.13481.1.S1_at	BG134320	TC22621	2025	2174	648	1.07	0.32	Transcribed locus, weakly similar to NP_001044052.1 [Oryza sativa (japonica cultivar-group)]
Les.5157.1.S1_at	BT013405.1	TC23882	4803	4206	1529	0.88	0.32	Lycopersicon esculentum clone 135719R, mRNA sequence
Les.4428.1.S1_a_at	AW443158	DB69734	707	823	222	1.16	0.31	carbonic anhydrase
LesAffx.69609.1.S1_at	AW929283	TC23404	208	147	65	0.71	0.31	Transcribed locus, weakly similar to NP_001043990.1 [Oryza sativa (japonica cultivar-group)]
Les.2964.1.A1_at	BG627802	TC23599	1413	1110	442	0.79	0.31	Transcribed locus, weakly similar to NP_001042324.1 [Oryza sativa (japonica cultivar-group)]

Les.144.1.S1_at	Z75519.1	TC22816	608	869	190	1.43	0.31	Partial mRNA (clone SEND32)
Les.327.1.S1_at	BT014378.1	TC22961	4422	4496	1377	1.02	0.31	Lycopersicon esculentum clone 133667F, mRNA sequence
Les.3128.2.S1_s_at	BI423262	TC23347	471	512	145	1.09	0.31	Lycopersicon esculentum clone 114279F, mRNA sequence
Les.4508.2.S1_s_at	AW623685	TC23882	5220	4769	1603	0.91	0.31	Lycopersicon esculentum clone 135719R, mRNA sequence
LesAffx.33796.2.S1_at	AW650353	TC23406	1963	1086	595	0.55	0.3	---
Les.3718.1.S1_at	AF384374.1	TC21922	2821	2954	853	1.05	0.3	allene oxide cyclase
Les.2504.1.A1_at	BG628687	TC23404	483	368	146	0.76	0.3	Transcribed locus, weakly similar to NP_001043990.1 [Oryza sativa (japonica cultivar-group)]
LesAffx.56547.2.S1_at	BI207815	TC22569	773	448	232	0.58	0.3	Transcribed locus, weakly similar to NP_001042475.1 [Oryza sativa (japonica cultivar-group)]
LesAffx.56221.1.S1_at	AI894713	TC24290	112	138	34	1.23	0.3	Transcribed locus, weakly similar to NP_001042068.1 [Oryza sativa (japonica cultivar-group)]
Les.3515.1.S1_at	AF242849.1	TC21891	1107	1224	330	1.11	0.3	wound-inducible carboxypeptidase
Les.3286.1.S1_at	BT013249.1	TC23425	5271	5601	1557	1.06	0.3	Lycopersicon esculentum inducible plastid-lipid associated protein (CHRD)
LesAffx.37212.1.A1_at	AI491145	TC23069	192	104	56	0.54	0.29	---
LesAffx.1.1.S1_at	AY656838.1	TC21732	2243	2378	653	1.06	0.29	arginase 2
Les.3376.1.A1_at	BG630058	TC21732	2956	2942	859	1	0.29	CONSTANS interacting protein 1
LesAffx.71535.1.S1_at	AW040299	TC23102	277	300	79	1.08	0.29	Transcribed locus, weakly similar to NP_001049750.1 [Oryza sativa (japonica cultivar-group)]
Les.24.1.S1_at	AF191823.1	TC21735	410	262	116	0.64	0.28	alpha-galactosidase
Les.4953.1.S1_at	BT012976.1	TC23170	1282	897	361	0.7	0.28	Lycopersicon esculentum clone 114187R, mRNA sequence
Les.3065.2.A1_at	BG630869	TC22543	941	480	263	0.51	0.28	Transcribed locus, weakly similar to NP_001042793.1 [Oryza sativa (japonica cultivar-group)]
Les.4345.4.A1_x_at	BG631293	TC22725	2058	1449	569	0.7	0.28	Pathogenesis-related protein P2
Les.4345.3.S1_x_at	AI775226	TC22725	5727	4434	1582	0.77	0.28	Pathogenesis-related protein P2
Les.1219.1.A1_at	BG630155	TC23499	161	201	45	1.25	0.28	Transcribed locus, weakly similar to NP_001047376.1 [Oryza sativa (japonica cultivar-group)]
Les.3376.2.S1_at	AW932459	TC21732	1317	1274	361	0.97	0.27	CONSTANS interacting protein 1
Les.1258.1.S1_at	AY240230.1	TC21731	2797	1685	767	0.6	0.27	gamma-aminobutyrate transaminase subunit precursor isozyme 2
Les.4258.1.A1_at	BG626687	TC23571	793	604	217	0.76	0.27	Transcribed locus, weakly similar to XP_001104139.1 similar to aarF domain containing kinase 1 isoform 2 [Macaca mulatta]
Les.2971.1.S1_at	AI781668	TC23191	2987	3170	806	1.06	0.27	Wound-induced proteinase inhibitor I prepropeptide
Les.3299.2.A1_s_at	BG626957	TC21732	1838	1968	495	1.07	0.27	arginase 2
Les.4258.3.S1_at	BG128812	TC23571	492	355	132	0.72	0.27	Transcribed locus, weakly similar to XP_001104139.1 similar to aarF domain containing kinase 1 isoform 2 [Macaca mulatta]
Les.2168.1.S1_at	BT013274.1	TC23964	3447	2528	914	0.73	0.27	Lycopersicon esculentum clone 134851R, mRNA sequence

Les.4345.4.A1_at	BG631293	TC22725	2387	1550	631	0.65	0.26	Pathogenesis-related protein P2
Les.4508.1.S1_s_at	AI780576	BG12438	4722	3993	1220	0.85	0.26	Lycopersicon esculentum clone 135719R, mRNA sequence
Les.1832.1.A1_at	BG629451	TC23307	1172	1556	301	1.33	0.26	Transcribed locus, moderately similar to NP_001062542.1 [Oryza sativa (japonica cultivar-group)]
Les.3297.1.S1_at	BG630303	TC21776	5073	3472	1277	0.68	0.25	Transcribed locus, weakly similar to NP_001043493.1 [Oryza sativa (japonica cultivar-group)]
Les.4345.2.A1_x_at	AI781554	TC23670	6562	5221	1622	0.8	0.25	Pathogenesis-related protein P2
Les.3297.2.S1_at	AI773077	TC23571	1395	840	345	0.6	0.25	Transcribed locus, weakly similar to NP_001043493.1 [Oryza sativa (japonica cultivar-group)]
LesAffx.10650.1.S1_at	AW036686	TC23252	1928	1945	476	1.01	0.25	CDNA, clone: FC20BC08, HTC in fruit
Les.2012.1.S1_at	BG627650	TC23373	258	189	62	0.73	0.24	Myo-inositol-1-phosphate synthase
Les.174.1.A1_at	BG627544	TC24015	109	68	26	0.62	0.24	Transcribed locus, moderately similar to NP_001045188.1 [Oryza sativa (japonica cultivar-group)]
Les.1314.1.S1_at	BG630759	TC24293	344	387	81	1.12	0.24	Transcribed locus
Les.3054.1.S1_at	AI773693	TC23429	4567	3441	1063	0.75	0.23	Lycopersicon esculentum clone 135718R, mRNA sequence
Les.4428.1.S1_at	AW443158	DB69734	457	551	105	1.2	0.23	carbonic anhydrase
Les.233.1.S1_at	M17558.1	TC23200	4325	3370	987	0.78	0.23	chlorophyll a/b-binding protein precursor
Les.4345.2.A1_a_at	AI781554	TC23670	5915	4475	1332	0.76	0.23	Lycopersicon esculentum clone 133385R, mRNA sequence
Les.4766.1.S1_at	CN385384	TC23006	3456	2381	776	0.69	0.22	Lycopersicon esculentum clone 133946F, mRNA sequence
Les.84.1.S1_at	U50151.1	TC21723	4421	4639	987	1.05	0.22	leucine aminopeptidase
Les.4868.1.S1_at	BT012795.1	TC22912	167	156	37	0.94	0.22	Lycopersicon esculentum clone 113794R, mRNA sequence
Les.3070.1.S1_at	BG126941	TC22028	3102	2930	658	0.94	0.21	Transcribed locus, weakly similar to NP_001042269.1 [Oryza sativa (japonica cultivar-group)]
Les.3070.2.A1_at	BG629293	TC22028	4745	4233	1003	0.89	0.21	Transcribed locus, weakly similar to NP_001042269.1 [Oryza sativa (japonica cultivar-group)]
Les.4281.3.S1_at	AI771846	TC21921	179	186	38	1.04	0.21	Transcribed locus, weakly similar to XP_001110787.1 ectonucleoside triphosphate diphosphohydrolase 3 [Macaca mulatta]
Les.4988.1.S1_at	BT013056.1	TC22946	2992	2643	617	0.88	0.21	Lycopersicon esculentum clone 114312R, mRNA sequence
LesAffx.10091.1.S1_at	AI487223	TC23911	233	170	48	0.73	0.21	Transcribed locus, weakly similar to NP_173851.1 [Arabidopsis thaliana]
Les.4281.1.A1_at	BG626715	TC21921	1554	1651	319	1.06	0.21	Transcribed locus, weakly similar to XP_001110787.1 ectonucleoside triphosphate diphosphohydrolase 3 [Macaca mulatta]
Les.4281.2.S1_at	BI926015	DB69892	932	1089	189	1.17	0.2	Transcribed locus, weakly similar to XP_001110787.1 ectonucleoside triphosphate diphosphohydrolase 3 [Macaca mulatta]
Les.4923.1.S1_at	BT012912.1	TC22925	566	759	113	1.34	0.2	Lycopersicon esculentum clone 114030R, mRNA sequence
Les.4405.1.A1_at	BG630861	TC24428	1573	832	302	0.53	0.19	Transcribed locus, moderately similar to NP_001047599.1 [Oryza sativa (japonica cultivar-group)]

LesAffx.65984.1.S1_at	BG125327	GO37430	2430	1237	462	0.51	0.19	Transcribed locus, weakly similar to NP_001044052.1 [Oryza sativa (japonica cultivar-group)]
Les.3322.3.S1_at	AW443800	BP90402	850	445	159	0.52	0.19	Transcribed locus, moderately similar to NP_001047599.1 [Oryza sativa (japonica cultivar-group)]
Les.3016.1.S1_at	BT012757.1	TC24030	1306	828	200	0.63	0.15	Lycopersicon esculentum clone 113701F, mRNA sequence
Les.2316.2.A1_at	BG626036	TC22940	3550	2137	522	0.6	0.15	Transcribed locus, weakly similar to NP_001044252.1 [Oryza sativa (japonica cultivar-group)]
Les.2316.1.S1_at	AW623527	TC22940	3402	2037	458	0.6	0.13	Transcribed locus, weakly similar to NP_001044252.1 [Oryza sativa (japonica cultivar-group)]
Les.147.1.S1_at	BG629070	TC22473	419	392	53	0.93	0.13	chlorophyll a/b-binding protein precursor
LesAffx.29797.1.S1_at	AJ784483	TC23412	497	391	58	0.79	0.12	Transcribed locus, weakly similar to NP_001042863.1 [Oryza sativa (japonica cultivar-group)]
Les.5201.1.S1_at	BT013496.1	FS19239	451	228	384	0.5	0.85	Lycopersicon esculentum clone 132182F, mRNA sequence
LesAffx.53474.1.S1_at	BG133264	TC22918	1208	609	654	0.5	0.54	Transcribed locus, weakly similar to NP_001042043.1 [Oryza sativa (japonica cultivar-group)]
Les.3017.3.S1_at	BE462343	TC21898	465	234	378	0.5	0.81	Transcribed locus, weakly similar to XP_001084411.1 aquaporin 1 [Macaca mulatta]
LesAffx.59952.1.S1_at	BF114023	TC23767	10	5	9	0.5	0.88	Transcribed locus, moderately similar to NP_001047491.1 [Oryza sativa (japonica cultivar-group)]
Les.5263.1.S1_at	BT013600.1	TC22756	284	143	208	0.5	0.73	Lycopersicon esculentum clone 132364F, mRNA sequence
LesAffx.66226.2.S1_at	BI205336	TC22735	438	220	335	0.5	0.76	Transcribed locus, weakly similar to NP_001041821.1 [Oryza sativa (japonica cultivar-group)]
LesAffx.9975.1.A1_at	AI487405	AI48740	51	26	34	0.5	0.66	---
Les.3701.1.S1_at	AF022022.1	TC24440	35	18	70	0.5	1.98	IAA11 protein
Les.34.1.S1_at	AF050496.1	TC21713	472	236	283	0.5	0.6	Ca2+-ATPase
Les.2626.1.S1_at	BG735008	TC22245	3727	1860	1994	0.5	0.54	Lycopersicon esculentum clone 132901F, mRNA sequence
Les.3094.3.S1_at	BG125458	TC23596	1688	842	608	0.5	0.36	Transcribed locus, weakly similar to NP_001041542.1 P450, family 51, subfamily A, polypeptide 1 [Gallus gallus]
Les.876.1.A1_at	BG627344	TC22377	215	107	85	0.5	0.4	Transcribed locus, weakly similar to NP_001051973.1 [Oryza sativa (japonica cultivar-group)]
LesAffx.47070.1.S1_at	AW651406	AW65140	45	22	39	0.5	0.88	Transcribed locus, moderately similar to NP_001042375.1 [Oryza sativa (japonica cultivar-group)]
Les.1654.1.A1_at	BG627748	TC23489	2715	1349	1705	0.5	0.63	Transcribed locus, weakly similar to NP_001042315.1 [Oryza sativa (japonica cultivar-group)]
LesAffx.54718.1.S1_at	BM411779	TC22617	804	399	315	0.5	0.39	Transcribed locus, moderately similar to NP_001044227.1 [Oryza sativa (japonica cultivar-group)]

LesAffx.71388.1.S1_at	AW034384	TC22565	237	118	93	0.5	0.39	Transcribed locus, weakly similar to NP_001042342.1 [Oryza sativa (japonica cultivar-group)]
Les.355.1.A1_at	BG627591	TC23931	599	297	263	0.5	0.44	Transcribed locus
LesAffx.65695.2.A1_at	AI484733	TC23451	319	158	223	0.49	0.7	Transcribed locus, moderately similar to NP_001047304.1 [Oryza sativa (japonica cultivar-group)]
Les.1079.3.A1_at	BG626088	GO37592	70	35	34	0.49	0.48	Transcribed locus, weakly similar to NP_001043162.1 [Oryza sativa (japonica cultivar-group)]
Les.3322.1.S1_at	BI935557	TC22083	595	293	112	0.49	0.19	Transcribed locus, moderately similar to NP_001047599.1 [Oryza sativa (japonica cultivar-group)]
Les.5290.1.S1_at	BT013644.1	TC23296	2487	1225	2254	0.49	0.91	Lycopersicon esculentum clone 132449R, mRNA sequence
Les.46.1.S1_at	X60760.1	NP00035	238	117	202	0.49	0.85	TDR8 protein
LesAffx.7472.2.S1_at	AW651303	TC23954	287	141	84	0.49	0.29	Transcribed locus, weakly similar to NP_001043730.1 [Oryza sativa (japonica cultivar-group)]
Les.1197.1.S1_at	BI925087	TC22471	542	266	218	0.49	0.4	Transcribed locus, weakly similar to XP_001090911.1 LanC lantibiotic synthetase component C-like 2 isoform 2 [Macaca mulatta]
Les.2092.1.S1_at	BG626759	TC22657	2326	1141	2102	0.49	0.9	Transcribed locus, weakly similar to XP_001111095.1 aldolase B isoform 5 [Macaca mulatta]
LesAffx.66458.1.A1_at	CK720557	TC23194	77	38	47	0.49	0.61	---
Les.2068.1.A1_at	BG631401	TC24040	454	222	320	0.49	0.71	Transcribed locus, weakly similar to NP_001045667.1 [Oryza sativa (japonica cultivar-group)]
Les.1376.2.A1_at	BG629093	TC23229	475	232	379	0.49	0.8	Transcribed locus, weakly similar to NP_001043392.1 [Oryza sativa (japonica cultivar-group)]
Les.3792.1.S1_s_at	AF249329.1	GO37419	1758	858	270	0.49	0.15	Lycopersicon esculentum putative cytochrome P450
Les.1248.2.A1_at	BT012749.1	TC21728	865	422	435	0.49	0.5	GDP-mannose pyrophosphorylase
Les.4707.1.S1_at	BT012714.1	TC23557	567	276	396	0.49	0.7	Lycopersicon esculentum clone 113596R, mRNA sequence
Les.4896.1.S1_at	BT012857.1	TC23109	2404	1172	933	0.49	0.39	Lycopersicon esculentum clone 113944R, mRNA sequence
LesAffx.41330.2.S1_at	AI899559	TC24064	499	243	217	0.49	0.43	Transcribed locus, weakly similar to NP_001067668.1 [Oryza sativa (japonica cultivar-group)]
LesAffx.71503.1.S1_at	AW648310	TC22254	1050	511	898	0.49	0.85	Transcribed locus, weakly similar to NP_001044300.1 [Oryza sativa (japonica cultivar-group)]
Les.5312.1.S1_at	BT013697.1	TC22735	4469	2175	742	0.49	0.17	Lycopersicon esculentum clone 132539F, mRNA sequence
Les.3017.1.A1_at	BG629826	AI78132	735	358	611	0.49	0.83	Transcribed locus, weakly similar to XP_001084411.1 aquaporin 1 [Macaca mulatta]
Les.3506.1.S1_at	AF365930.1	TC21713	883	429	1004	0.49	1.14	verticillium wilt disease resistance protein Ve2
Les.305.3.S1_at	AW930072	TC23404	1000	486	566	0.49	0.57	Transcribed locus, weakly similar to XP_001100725.1 similar to eukaryotic translation initiation factor 2C, 2 [Macaca mulatta]
Les.4493.3.S1_at	CN385077	TC23105	195	95	84	0.49	0.43	ADP/ATP translocator

LesAffx.24799.1.S1_at	CK715756	TC23394	625	303	402	0.48	0.64	Transcribed locus, weakly similar to NP_001045764.1 [Oryza sativa (japonica cultivar-group)]
Les.1108.1.A1_at	BG629434	TC22374	492	238	302	0.48	0.61	Transcribed locus, weakly similar to NP_001042402.1 [Oryza sativa (japonica cultivar-group)]
Les.4397.1.S1_at	BG127578	TC23761	268	129	170	0.48	0.63	CDNA, clone: FC25AG12, HTC in fruit
Les.4397.2.A1_at	AW218741	TC23761	693	334	427	0.48	0.62	CDNA, clone: FC25AG12, HTC in fruit
Les.1205.2.S1_at	BM536081	TC21911	688	331	180	0.48	0.26	Transcribed locus, weakly similar to NP_001059455.1 [Oryza sativa (japonica cultivar-group)]
LesAffx.41330.1.A1_at	CK574967	TC24064	326	157	138	0.48	0.42	Transcribed locus, weakly similar to NP_001067668.1 [Oryza sativa (japonica cultivar-group)]
LesAffx.64675.2.A1_at	AJ784544	TC21939	207	100	127	0.48	0.61	Transcribed locus, weakly similar to NP_001052406.1 [Oryza sativa (japonica cultivar-group)]
Les.1465.1.S1_at	BG631822	TC23566	1249	601	491	0.48	0.39	Lycopersicon esculentum clone 132131F, mRNA sequence
Les.1320.1.A1_at	BG630795	BG63079	25	12	14	0.48	0.58	Transcribed locus
Les.5352.1.S1_at	BT013754.1	TC23917	1980	946	1078	0.48	0.54	Lycopersicon esculentum clone 132620F, mRNA sequence
Les.2219.1.A1_at	BG735134	TC23582	768	365	391	0.47	0.51	Transcribed locus, weakly similar to NP_568624.1 protein [Arabidopsis thaliana]
Les.1197.2.A1_at	BG630003	TC23124	2179	1029	943	0.47	0.43	Transcribed locus, weakly similar to XP_001090911.1 LanC lantibiotic synthetase component C-like 2 isoform 2 [Macaca mulatta]
LesAffx.44609.1.S1_at	BG631285	TC23469	498	235	657	0.47	1.32	Transcribed locus, weakly similar to NP_187615.2 factor [Arabidopsis thaliana]
Les.1857.1.A1_at	BG629691	TC23786	90	43	86	0.47	0.96	Transcribed locus, weakly similar to NP_001060003.1 [Oryza sativa (japonica cultivar-group)]
Les.4050.1.A1_at	L26982.1		394	185	221	0.47	0.56	Lycopersicon esculentum (DB173) mRNA fragment
LesAffx.17150.1.A1_at	AW039265	TC23237	262	123	129	0.47	0.49	Nitrite reductase
LesAffx.67767.1.S1_at	BF096648	TC22922	185	87	261	0.47	1.41	Transcribed locus, weakly similar to NP_568700.1 protein [Arabidopsis thaliana]
LesAffx.46046.1.S1_at	BI208410	TC24515	280	131	420	0.47	1.5	---
Les.1205.3.S1_at	BM410805	TC21911	1109	520	289	0.47	0.26	Transcribed locus, weakly similar to NP_001059455.1 [Oryza sativa (japonica cultivar-group)]
LesAffx.41518.1.S1_at	AW930080	TC23094	250	116	149	0.47	0.59	Transcribed locus, weakly similar to NP_001050851.1 [Oryza sativa (japonica cultivar-group)]
LesAffx.71563.1.S1_at	AW930851	TC22892	491	228	294	0.46	0.6	---
Les.2474.2.A1_at	BG735266	TC23985	1861	861	503	0.46	0.27	Transcribed locus, weakly similar to XP_001108186.1 similar to 24-dehydrocholesterol reductase precursor [Macaca mulatta]
Les.3519.1.S1_at	AJ002589.1	TC23298	302	139	163	0.46	0.54	CycD3;2 protein
Les.68.2.S1_a_at	BT013727.1	TC21796	2136	988	906	0.46	0.42	endo-beta-1,4-D-glucanase
Les.1821.1.A1_at	BG629348	BG62934	341	157	201	0.46	0.59	Transcribed locus

Les.181.1.A1_at	BG630947	TC23820	325	149	177	0.46	0.55	Transcribed locus, weakly similar to NP_001043252.1 [Oryza sativa (japonica cultivar-group)]
Les.4979.1.S1_at	BT013040.1	TC21739	457	210	300	0.46	0.66	Lycopersicon esculentum clone 114290R, mRNA sequence
LesAffx.885.1.S1_at	BI205930	BI20593	262	121	145	0.46	0.55	---
Les.1376.1.A1_at	BG631198	TC23229	1540	706	1125	0.46	0.73	Transcribed locus, weakly similar to NP_001043392.1 [Oryza sativa (japonica cultivar-group)]
LesAffx.26471.3.A1_at	AW218200	TC22883	289	132	152	0.46	0.53	Transcribed locus, weakly similar to NP_001054088.1 [Oryza sativa (japonica cultivar-group)]
Les.1179.1.A1_at	BG629888	TC24291	211	96	183	0.46	0.87	Transcribed locus
LesAffx.24799.2.S1_at	BI209670	TC24255	1037	473	653	0.46	0.63	Transcribed locus, weakly similar to NP_001045764.1 [Oryza sativa (japonica cultivar-group)]
LesAffx.3570.4.S1_at	AW624180	TC22612	1125	512	886	0.46	0.79	Ribosomal protein L2
LesAffx.46550.1.S1_at	BG134870	BG13487	127	58	130	0.46	1.02	---
LesAffx.30807.1.S1_at	AW624957	AW62495	1352	615	1013	0.45	0.75	Transcribed locus, weakly similar to XP_001116629.1 similar to fatty acid desaturase 2 isoform 2 [Macaca mulatta]
Les.3477.1.S1_at	AF230198.2	TC23205	112	51	41	0.45	0.37	hypothetical LOC543641
Les.857.1.S1_at	BT013838.1	TC22565	575	260	244	0.45	0.42	Lycopersicon esculentum clone 132795F, mRNA sequence
Les.3198.1.S1_at	L13654.1	TC22193	232	105	80	0.45	0.35	Lycopersicon esculentum peroxidase (TPX1)
Les.3065.1.S1_at	BI929558	TC22543	618	279	191	0.45	0.31	Transcribed locus, weakly similar to NP_001042793.1 [Oryza sativa (japonica cultivar-group)]
Les.2688.1.S1_at	AJ560646.1	TC21917	431	194	578	0.45	1.34	expansin11
Les.5327.1.S1_at	BT013719.1	TC24178	1067	476	630	0.45	0.59	Lycopersicon esculentum clone 132567F, mRNA sequence
Les.360.1.S1_at	M86724.1	TC23986	2417	1075	3862	0.44	1.6	alcohol dehydrogenase
Les.3312.2.S1_at	BI932707	TC21843	1807	802	1307	0.44	0.72	Transcribed locus, weakly similar to NP_001043993.1 [Oryza sativa (japonica cultivar-group)]
Les.4050.1.S1_at	L26982.1		409	182	229	0.44	0.56	Lycopersicon esculentum (DB173) mRNA fragment
Les.2014.1.A1_at	BG629750	TC23266	2443	1081	1800	0.44	0.74	Transcribed locus, weakly similar to NP_001046281.1 [Oryza sativa (japonica cultivar-group)]
Les.5161.1.S1_at	BT013412.1	AI77756	331	146	204	0.44	0.61	Lycopersicon esculentum clone 132049R, mRNA sequence
LesAffx.5088.1.S1_at	BI206366	BI20636	63	28	33	0.44	0.52	---
Les.5579.1.S1_at	BT014190.1	TC23962	970	425	961	0.44	0.99	Lycopersicon esculentum clone 133367R, mRNA sequence
Les.5150.1.S1_at	BT013389.1	AW92832	538	233	288	0.43	0.53	Lycopersicon esculentum clone 135570F, mRNA sequence
Les.5233.1.S1_at	CN384566	EG55296	258	111	245	0.43	0.95	Lycopersicon esculentum clone 132260F, mRNA sequence
LesAffx.4981.1.S1_at	AW623022	TC22429	4746	2048	1251	0.43	0.26	Transcribed locus, weakly similar to NP_001041979.1 [Oryza sativa (japonica cultivar-group)]

Les.2474.1.S1_at	BG791226	TC23985	996	430	284	0.43	0.29	Transcribed locus, weakly similar to XP_001108186.1 similar to 24-dehydrocholesterol reductase precursor [Macaca mulatta]
LesAffx.62070.1.S1_at	BI924487	TC23266	2797	1201	2107	0.43	0.75	Transcribed locus, weakly similar to NP_001046281.1 [Oryza sativa (japonica cultivar-group)]
LesAffx.58227.1.S1_at	CK714930	CK71493	351	150	230	0.43	0.66	---
LesAffx.66879.1.A1_at	BG631312	TC22769	1037	442	548	0.43	0.53	CDNA, clone: FC04CE09, HTC in fruit
LesAffx.58110.1.S1_at	BE435276	BE43527	1314	559	590	0.43	0.45	---
LesAffx.61034.1.S1_at	BG643132	TC21983	1515	644	1233	0.42	0.81	Transcribed locus, weakly similar to NP_001043558.1 [Oryza sativa (japonica cultivar-group)]
Les.4962.1.S1_at	BI204454	TC22450	929	394	798	0.42	0.86	Lycopersicon esculentum clone 114228R, mRNA sequence
LesAffx.61000.1.S1_at	BM410987	TC23050	1288	543	600	0.42	0.47	Transcribed locus, weakly similar to NP_001045184.1 [Oryza sativa (japonica cultivar-group)]
LesAffx.67767.1.A1_at	AI486022	TC22922	152	64	155	0.42	1.02	Transcribed locus, weakly similar to NP_568700.1 protein [Arabidopsis thaliana]
Les.4710.1.S1_at	BT014526.1	TC24079	67	28	23	0.42	0.34	Lycopersicon esculentum clone 133940F, mRNA sequence
Les.252.1.S1_at	U35643.1	DB68734	2438	1021	1852	0.42	0.76	Alpha-dioxygenase 2
LesAffx.56.16.A1_at	BG643188	AI89840	234	98	150	0.42	0.64	Transcribed locus, moderately similar to NP_001059779.1 [Oryza sativa (japonica cultivar-group)]
Les.2092.2.S1_at	BI203427	BG63176	1322	550	1088	0.42	0.82	Transcribed locus, weakly similar to XP_001111095.1 aldolase B isoform 5 [Macaca mulatta]
LesAffx.24799.2.A1_at	BI209670	TC24255	483	199	284	0.41	0.59	Transcribed locus, weakly similar to NP_001045764.1 [Oryza sativa (japonica cultivar-group)]
Les.3064.1.S1_at	U81033.1	TC21721	1637	674	963	0.41	0.59	ADP-glucose pyrophosphorylase large subunit
LesAffx.23349.1.S1_at	AI782014	TC21920	887	361	1050	0.41	1.18	ADP/ATP translocator
Les.2091.1.S1_at	BT013457.1	TC22626	621	252	502	0.41	0.81	Lycopersicon esculentum clone 132113R, mRNA sequence
LesAffx.46815.2.S1_at	BI929852	TC22829	2607	1058	1179	0.41	0.45	Hypothetical LOC544002
Les.5918.1.S1_at	AY224079.1	TC21718	376	152	281	0.41	0.75	ferric-chelate reductase
LesAffx.9038.1.S1_at	BI930643	TC22956	946	381	272	0.4	0.29	Transcribed locus, weakly similar to NP_001042297.1 [Oryza sativa (japonica cultivar-group)]
LesAffx.41774.1.S1_at	AW031192	TC22848	915	368	635	0.4	0.69	Transcribed locus, weakly similar to NP_001045466.1 [Oryza sativa (japonica cultivar-group)]
Les.2539.1.A1_at	BG628858	TC24275	119	47	95	0.4	0.8	Transcribed locus
LesAffx.58019.1.S1_at	BI929233	TC22171	269	106	136	0.4	0.5	Transcribed locus, weakly similar to NP_001052012.1 [Oryza sativa (japonica cultivar-group)]
LesAffx.33890.1.S1_at	BG134582	TC22584	190	75	108	0.39	0.57	Transcribed locus, weakly similar to NP_001067602.1 [Oryza sativa (japonica cultivar-group)]
			43	17	24	0.39	0.57	---

Les.5850.1.S1_at	BT014189.1	TC21894	438	170	224	0.39	0.51	Lycopersicon esculentum clone 133366R, mRNA sequence
LesAffx.4617.1.A1_at	BG735509	TC23088	864	335	478	0.39	0.55	Transcribed locus, weakly similar to NP_001041735.1 [Oryza sativa (japonica cultivar-group)]
LesAffx.58110.1.A1_at	AJ785349	BE43527	312	121	126	0.39	0.4	---
Les.263.1.S1_at	M98466.1	TC21722	2490	963	1824	0.39	0.73	polygalacturonase isoenzyme 1 beta subunit
LesAffx.17924.1.S1_at	BG124217	TC24101	1606	619	803	0.39	0.5	Transcribed locus, weakly similar to NP_001049241.1 [Oryza sativa (japonica cultivar-group)]
LesAffx.71563.1.A1_at	AI487345	TC22892	380	146	191	0.38	0.5	---
LesAffx.16472.1.S1_at	AI774397	TC24204	125	48	116	0.38	0.93	---
LesAffx.46815.1.S1_at	BI934723	TC21911	935	357	394	0.38	0.42	Hypothetical LOC544002
Les.642.1.S1_at	BG626672	BG62667	135	51	48	0.38	0.35	Lycopersicon esculentum clone 132200F, mRNA sequence
LesAffx.62334.1.S1_at	AW647879	AW64787	29	11	25	0.38	0.86	---
LesAffx.37636.1.S1_at	CN384813	EG55389	60	22	43	0.37	0.72	---
LesAffx.53591.1.S1_at	AI899018	TC22292	242	90	36	0.37	0.15	Transcribed locus, weakly similar to XP_001101267.1 similar to myeloblastosis proto-oncogene product isoform 2 [Macaca mulatta]
Les.1549.1.A1_at	BG626692	TC23631	811	301	431	0.37	0.53	Transcribed locus
LesAffx.30937.1.A1_at	AJ785090		285	105	152	0.37	0.53	---
LesAffx.64757.1.S1_at	AI490042	TC22570	1101	403	624	0.37	0.57	Transcribed locus
LesAffx.6110.1.S1_at	BF098450	TC22721	141	51	46	0.36	0.33	---
LesAffx.63209.1.S1_at	AW092854	AW09285	265	95	385	0.36	1.45	---
LesAffx.10497.2.A1_at	AI897776	TC24435	4369	1559	2285	0.36	0.52	Transcribed locus, weakly similar to NP_001042618.1 [Oryza sativa (japonica cultivar-group)]
Les.369.1.S1_at	AF059489.1	TC21922	2377	836	2268	0.35	0.95	expansin
Les.6.1.S1_at	AF261141.1	TC22389	1181	414	512	0.35	0.43	hypothetical LOC543656
Les.1205.1.A1_at	BG630057	TC21911	934	325	278	0.35	0.3	Transcribed locus, weakly similar to NP_001059455.1 [Oryza sativa (japonica cultivar-group)]
Les.4529.1.S1_at	AY497478.1	TC21903	1468	508	281	0.35	0.19	xyloglucan endotransglucosylase-hydrolase XTH7
LesAffx.71035.1.S1_at	BI207404	TC23510	796	276	824	0.35	1.03	Transcribed locus, weakly similar to NP_179716.1 protein [Arabidopsis thaliana]
Les.3552.1.A1_at	AF081021.1	TC24327	57	19	30	0.34	0.53	hypoxia-induced protein 242
Les.4549.1.S1_at	BT013085.1	TC24365	182	62	73	0.34	0.4	Lycopersicon esculentum clone 114349R, mRNA sequence
LesAffx.10497.2.S1_at	AI897776	TC24435	853	288	373	0.34	0.44	Transcribed locus, weakly similar to NP_001042618.1 [Oryza sativa (japonica cultivar-group)]
Les.4938.1.S1_at	BT012940.1	TC22310	628	212	247	0.34	0.39	Lycopersicon esculentum clone 114108R, mRNA sequence
Les.4749.1.S1_at	BT013546.1	AI77484	842	280	597	0.33	0.71	Lycopersicon esculentum clone 132267F, mRNA sequence
LesAffx.64757.1.A1_at	AI490042	TC22570	683	227	375	0.33	0.55	Transcribed locus

LesAffx.41816.1.S1_at	AW649474	TC22094	300	99	229	0.33	0.76	Transcribed locus, weakly similar to XP_001102886.1 hexosaminidase B isoform 1 [Macaca mulatta]
LesAffx.3099.1.S1_at	CK574995	GO37548	5316	1733	1247	0.33	0.23	Transcribed locus, moderately similar to NP_001044625.1 [Oryza sativa (japonica cultivar-group)]
LesAffx.56.4.S1_at	BG128707	TC22262	311	100	139	0.32	0.45	Transcribed locus, weakly similar to XP_001087943.1 similar to carboxypeptidase, vitellogenic-like isoform 4 [Macaca mulatta]
Les.1959.1.A1_at	BG630503	TC23129	139	45	71	0.32	0.51	Transcribed locus
Les.4731.1.S1_at	BT013577.1	TC23297	3148	952	2661	0.3	0.85	Lycopersicon esculentum clone 132322R, mRNA sequence
Les.2325.1.S1_at	BG625896	TC22401	834	249	337	0.3	0.4	yfe37 protein
Les.1714.1.A1_at	BG628281	TC24400	550	164	263	0.3	0.48	Transcribed locus
Les.5664.1.S1_at	BT014348.1	AW03195	525	156	665	0.3	1.27	Lycopersicon esculentum clone 133623F, mRNA sequence
Les.852.1.A1_at	BG627744	TC24030	117	35	34	0.3	0.29	Transcribed locus
Les.1614.1.A1_at	BG628742	TC23793	386	112	157	0.29	0.41	Transcribed locus, moderately similar to NP_001049792.1 [Oryza sativa (japonica cultivar-group)]
Les.4594.1.S1_at	BT012739.1	TC23704	2671	757	1627	0.28	0.61	Lycopersicon esculentum clone 113677R, mRNA sequence
Les.4473.1.S1_at	AJ606077.1	TC22116	301	85	364	0.28	1.21	spe4 protein
LesAffx.13252.1.S1_at	BI929109	TC22202	1288	359	362	0.28	0.28	Transcribed locus, weakly similar to NP_001042395.1 [Oryza sativa (japonica cultivar-group)]
Les.3069.1.S1_at	BG630752	TC21938	830	228	531	0.27	0.64	Lycopersicon esculentum clone 132988F, mRNA sequence
Les.3733.1.S1_at	AF096776.1	TC21836	933	245	569	0.26	0.61	expansin
LesAffx.70738.1.A1_at	BG630069	TC23199	488	120	122	0.25	0.25	Transcribed locus, weakly similar to XP_001101267.1 similar to myeloblastosis proto-oncogene product isoform 2 [Macaca mulatta]
LesAffx.13252.2.S1_at	BG124820	TC22202	800	196	186	0.25	0.23	Transcribed locus, weakly similar to NP_001042395.1 [Oryza sativa (japonica cultivar-group)]
LesAffx.13252.2.A1_at	BG124820	TC22202	237	57	64	0.24	0.27	Transcribed locus, weakly similar to NP_001042395.1 [Oryza sativa (japonica cultivar-group)]
Les.5941.1.A1_at	CK715706	TC22893	1477	351	1097	0.24	0.74	Transcribed locus, weakly similar to NP_001042315.1 [Oryza sativa (japonica cultivar-group)]
Les.9.1.S1_at	AB010992.1	TC22813	1614	368	482	0.23	0.3	3b-hydroxylase
Les.406.1.A1_at	BG629308	TC24082	1036	204	297	0.2	0.29	Transcribed locus, weakly similar to NP_196850.1 binding [Arabidopsis thaliana]
LesAffx.1251.1.S1_at	BG125851	BG12585	307	51	204	0.17	0.66	---
