# Supplementary material for: Transcriptome Analysis of Tomato Flower Pedicel Tissues Reveals Abscission Zone-Specific Modulation of Key Meristem Activity Genes
Source: PLoS One. 2013 Feb 4;8(2):e55238. doi: 10.1371/journal.pone.0055238 (PMC3563536; doi:10.1371/journal.pone.0055238)
Supplement: Table S5 — Differentially expressed genes in the apical portion after 3 h or 6 h of ethylene treatment. (PDF) [file pone.0055238.s007.pdf]

Table S5. Differentially expressed genes in the apical portion after 3 h and/or 6 h of ethylene treatment.

Affymetrix Probe Set ID	GenBank Accession	Tomato Gene Index	0AP	3AP	6AP	3AP/0AP	6AP/0AP	GenBank Annotation
Les.4885.1.S1_at	BT012835.1	TC24138	136	2376	1202	17.48	8.84	Lycopersicon esculentum clone 113896R, mRNA sequence
Les.113.1.S1_at	CN384955	TC23130	443	4256	6495	9.6	14.64	CDNA, clone: FC02BG03, HTC in fruit
LesAffx.56389.1.S1_at	AW034707	TC23934	81	666	2651	8.21	32.65	Transcribed locus, weakly similar to NP_001033715.2 F synthase [Sus scrofa]
Les.3465.1.S1_at	AY079426.1	TC21718	129	927	799	7.18	6.19	ethylene receptor-like protein
LesAffx.56301.1.S1_at	AI895341	TC22182	597	3810	2471	6.38	4.14	Transcribed locus, weakly similar to NP_001048145.1 [Oryza sativa (japonica cultivar-group)]
Les.3712.1.S1_at	AF154003.1	TC24028	565	3223	3549	5.7	6.28	pirin
LesAffx.54536.1.S1_at	BM409044	TC23408	128	721	703	5.66	5.51	Transcribed locus, weakly similar to NP_001046834.1 [Oryza sativa (japonica cultivar-group)]
LesAffx.13365.1.S1_at	BM411110	TC21920	542	2989	778	5.51	1.43	Transcribed locus, weakly similar to NP_001066487.1 [Oryza sativa (japonica cultivar-group)]
LesAffx.24637.1.S1_at	CN550618	TC22796	276	1413	4587	5.12	16.63	Transcribed locus, weakly similar to NP_001049089.1 [Oryza sativa (japonica cultivar-group)]
LesAffx.66953.1.S1_at	AW651552	TC23604	508	2527	2910	4.97	5.72	Transcribed locus, weakly similar to NP_001042903.1 [Oryza sativa (japonica cultivar-group)]
Les.3645.1.S1_at	U54770.1	NP924378	668	3317	6227	4.96	9.32	6-deoxocastasterone oxidase
LesAffx.70406.1.S1_at	AW036249	TC22360	109	539	1056	4.93	9.66	Transcribed locus, weakly similar to XP_001114236.1 branched chain aminotransferase 2, mitochondrial isoform 2 [Macaca mulatta]
Les.2899.1.S1_at	BG628131	TC22810	2384	11063	8998	4.64	3.77	Transcribed locus, weakly similar to XP_001108862.1 cathepsin H isoform 2 [Macaca mulatta]
LesAffx.69349.2.S1_at	AW623862	AW62386	214	955	1041	4.47	4.87	Transcribed locus, weakly similar to NP_001043801.1 [Oryza sativa (japonica cultivar-group)]
Les.4443.1.A1_s_at	BG630528	TC21730	928	4136	6638	4.46	7.16	6-deoxocastasterone oxidase
Les.4287.1.S1_at	CB751564	TC21784	1045	4642	6634	4.44	6.35	Transcribed locus, weakly similar to XP_001094505.1 proteasome 26S non-ATPase subunit 3 isoform 4 [Macaca mulatta]
LesAffx.63659.1.S1_at	BI921813	TC22794	380	1633	2849	4.3	7.51	Transcribed locus, weakly similar to NP_001047062.1 [Oryza sativa (japonica cultivar-group)]
LesAffx.50112.1.S1_at	AI898985	TC23361	524	2248	3667	4.29	7	Transcribed locus, weakly similar to NP_001047062.1 [Oryza sativa (japonica cultivar-group)]
Les.132.1.S1_at	X58885.1	TC21743	2652	11261	11802	4.25	4.45	ethylene-forming enzyme
LesAffx.9910.1.S1_at	CN385590	TC22147	394	1669	2163	4.23	5.49	Transcribed locus, weakly similar to NP_001046094.1 [Oryza sativa (japonica cultivar-group)]

Les.2084.1.S1_at	BF097539	TC22764	372	1539	1802	4.14	4.84	Transcribed locus, weakly similar to NP_001041763.1 [Oryza sativa (japonica cultivar-group)]
LesAffx.15921.1.S1_at	BM412286	TC23758	473	1900	1903	4.02	4.03	Transcribed locus, weakly similar to NP_001057315.1 [Oryza sativa (japonica cultivar-group)]
LesAffx.64582.2.A1_at	AI490624	TC24338	54	215	141	3.96	2.59	---
Les.1524.1.A1_at	BG626458	BG62645	322	1275	1097	3.96	3.41	Transcribed locus
LesAffx.37563.1.S1_at	Z70216.1	TC23549	61	237	103	3.89	1.69	---
Les.2899.2.S1_at	BM536145	TC22810	992	3859	3060	3.89	3.08	Transcribed locus, weakly similar to XP_001108862.1 cathepsin H isoform 2 [Macaca mulatta]
Les.5442.1.S1_at	BT013931.1	TC22814	385	1497	2777	3.88	7.21	Lycopersicon esculentum clone 132929R, mRNA sequence
Les.4055.1.S1_at	BM536050	TC23122	112	427	436	3.8	3.89	Lycopersicon esculentum (DB222) meloidogyne-induced giant cell protein mRNA, 3' end
Les.4307.1.S1_at	AY257487.1	TC21819	2647	9970	10398	3.77	3.93	PR5-like protein
Les.5126.1.S1_at	BT013336.1	TC22310	240	894	1809	3.73	7.54	Lycopersicon esculentum clone 135126R, mRNA sequence
LesAffx.58502.1.S1_at	BM412364	TC22738	421	1567	886	3.72	2.1	Transcribed locus, weakly similar to NP_001042057.1 [Oryza sativa (japonica cultivar-group)]
Les.244.2.S1_a_at	AI485696	TC22935	298	1096	1049	3.68	3.53	Calmodulin
Les.3683.1.S1_at	AY093595.1	TC24086	261	956	1187	3.66	4.54	PR-5x
LesAffx.64582.1.S1_at	AI894448	TC24338	141	510	349	3.62	2.48	---
LesAffx.33607.1.S1_s_at	AW223067	TC23890	410	1475	1444	3.6	3.52	Calmodulin
Les.423.1.S1_at	BM409421	TC22953	976	3493	3886	3.58	3.98	CDNA, clone: FC09CF07, HTC in fruit
LesAffx.63489.1.S1_at	BI921137	TC22082	654	2341	879	3.58	1.34	Transcribed locus, weakly similar to XP_001117063.1 transmembrane BAX inhibitor motif containing 4 isoform 1 [Macaca mulatta]
LesAffx.33607.2.S1_s_at	AI775872	TC23890	1015	3632	3683	3.58	3.63	Calmodulin
Les.1046.1.S1_at	X72730.1	TC22785	315	1122	1067	3.56	3.39	(ERT 10) ripening-related mRNA
LesAffx.22583.1.S1_at	AI484022	TC23670	216	755	725	3.5	3.36	Transcribed locus, weakly similar to NP_001044974.1 [Oryza sativa (japonica cultivar-group)]
Les.796.1.A1_at	BG627506	TC22633	145	501	994	3.44	6.84	Transcribed locus, weakly similar to NP_001043676.1 [Oryza sativa (japonica cultivar-group)]
LesAffx.57163.2.S1_at	BI421649	TC22802	114	388	64	3.39	0.56	Transcribed locus, weakly similar to NP_001044707.1 [Oryza sativa (japonica cultivar-group)]
Les.2591.1.S1_at	M80608.1		1087	3649	6411	3.36	5.9	beta-1,3-glucanase
Les.4223.1.S1_at	AY034148.1	TC21729	363	1169	1344	3.22	3.7	alternative oxidase 1a
LesAffx.3467.1.S1_at	CN384781	TC23179	554	1699	1930	3.07	3.48	Transcribed locus, weakly similar to NP_001047645.1 [Oryza sativa (japonica cultivar-group)]

LesAffx.63739.1.S1_at	AW033134	TC21965	104	317	395	3.04	3.79	Transcribed locus, weakly similar to NP_001043665.1 [Oryza sativa (japonica cultivar-group)]
Les.4999.1.A1_at	BT013077.1	TC23341	299	907	781	3.04	2.62	Lycopersicon esculentum clone 114334F, mRNA sequence
LesAffx.30544.1.S1_at	AI489986	TC24194	1128	3404	3828	3.02	3.39	---
LesAffx.30544.1.A1_at	AI489986	TC24194	427	1283	1388	3	3.25	---
LesAffx.68306.1.S1_at	BE461086	TC23074	108	324	137	3	1.27	Transcribed locus, weakly similar to NP_001055605.1 [Oryza sativa (japonica cultivar-group)]
LesAffx.47221.1.S1_at	AW033464	AW03346	1056	3158	3833	2.99	3.63	Transcribed locus, weakly similar to NP_001041994.1 [Oryza sativa (japonica cultivar-group)]
Les.4483.1.S1_at	AY498713.1	TC21812	605	1802	1519	2.98	2.51	NAC domain protein
LesAffx.59769.1.S1_at	BI203677	TC23621	804	2387	3826	2.97	4.76	---
LesAffx.64741.1.S1_at	AI775317	TC22074	1088	3210	3433	2.95	3.16	Transcribed locus, weakly similar to XP_001103467.1 similar to 3-hydroxyisobutyryl-Coenzyme A hydrolase isoform 1 [Macaca mulatta]
LesAffx.56596.1.S1_at	AW036395	AW03639	44	129	114	2.95	2.61	---
Les.3668.1.S1_at	U09026.1	TC21838	89	261	497	2.95	5.61	lipoxygenase
LesAffx.71606.1.S1_s_at	AI896002	TC24027	1348	3965	3500	2.94	2.6	Lycopersicon esculentum clone 114334F, mRNA sequence
LesAffx.61149.1.S1_at	BE460847	TC24084	262	763	1473	2.91	5.61	Transcribed locus, weakly similar to NP_001042177.1 [Oryza sativa (japonica cultivar-group)]
LesAffx.43198.1.S1_at	BF113993	BF11399	134	388	463	2.9	3.45	Transcribed locus, weakly similar to NP_001045064.1 [Oryza sativa (japonica cultivar-group)]
LesAffx.124.1.S1_at	AW443936	TC23232	41	118	139	2.89	3.4	CDNA, clone: FC20CD10, HTC in fruit
Les.3646.1.S1_at	U70481.1	TC21735	14	41	173	2.89	12.26	abscission polygalacturonase
LesAffx.47179.1.S1_at	BM411655	TC24073	653	1884	970	2.89	1.49	Transcribed locus, weakly similar to NP_010015.2 serine/threonine protein kinase most similar to cyclic nucleotide-dependent protein kinase subfamily and the protein kinase C subfamily; Kin82p [Saccharomyces cerevisiae]
Les.3752.1.S1_at	AW036285	NP16439	87	250	141	2.87	1.62	CDNA, clone: FC23CF12, HTC in fruit
Les.3383.1.S1_at	BI208081	TC22532	745	2134	1169	2.86	1.57	Transcribed locus, weakly similar to XP_001118353.1 similar to olfactory specific medium-chain acyl CoA synthetase [Macaca mulatta]
Les.2137.1.S1_at	AY359965.1	TC21714	732	2096	2411	2.86	3.29	EIX receptor 1
LesAffx.3467.1.A1_at	CN384781	TC23179	257	729	776	2.84	3.02	Transcribed locus, weakly similar to NP_001047645.1 [Oryza sativa (japonica cultivar-group)]
Les.702.1.S1_at	BG627003	TC21772	1100	3118	1998	2.84	1.82	Lycopersicon esculentum clone 134862F, mRNA sequence
Les.217.1.S1_at	AF243180.1	TC22069	499	1404	1814	2.81	3.63	dicyanin
LesAffx.70522.1.S1_at	BI423015	TC23029	414	1144	1269	2.76	3.07	Transcribed locus, weakly similar to NP_001055496.1 [Oryza sativa (japonica cultivar-group)]
Les.5091.1.S1_at	BT013255.1	TC22888	203	557	401	2.74	1.98	Lycopersicon esculentum clone 134773R, mRNA sequence

Les.3619.1.S1_at	J04099.1	TC23203	2564	6966	8264	2.72	3.22	proteinase inhibitor I
Les.3735.1.S1_at	AY007561.1	TC22594	166	450	672	2.72	4.05	hypothetical LOC543816
LesAffx.64990.1.S1_at	BI422984	TC21791	441	1188	1001	2.7	2.27	Transcribed locus, weakly similar to NP_001051696.1 [Oryza sativa (japonica cultivar-group)]
Les.3486.1.S1_at	AF416289.1	TC22056	910	2449	3009	2.69	3.31	auxin-regulated protein
LesAffx.56802.1.S1_at	BI423210	TC21932	894	2401	4008	2.69	4.48	Transcribed locus, weakly similar to NP_001044900.1 [Oryza sativa (japonica cultivar-group)]
LesAffx.20450.1.S1_at	AW223514	TC21776	2151	5730	4550	2.66	2.12	Transcribed locus, weakly similar to NP_001042868.1 [Oryza sativa (japonica cultivar-group)]
Les.2212.1.A1_at	BG735110	BG73511	117	310	266	2.66	2.28	Lycopersicon esculentum clone 133400R, mRNA sequence
LesAffx.57163.3.A1_at	AI486803	TC22802	61	164	46	2.66	0.75	Transcribed locus, weakly similar to NP_001042186.1 [Oryza sativa (japonica cultivar-group)]
LesAffx.17387.1.S1_at	BM534931	BM53493	259	682	1245	2.63	4.8	---
LesAffx.66354.1.S1_at	AI773917	TC22427	65	169	118	2.62	1.83	Transcribed locus, weakly similar to NP_001057807.1 [Oryza sativa (japonica cultivar-group)]
LesAffx.69783.1.S1_at	AW220917	TC23448	1210	3165	3428	2.61	2.83	Transcribed locus, weakly similar to NP_001067067.1 [Oryza sativa (japonica cultivar-group)]
Les.244.2.S1_at	AI485696	TC22935	134	348	375	2.6	2.8	Calmodulin
Les.91.1.S1_at	Z97215.1	TC22836	549	1422	1461	2.59	2.66	nine-cis-epoxycarotenoid dioxygenase
Les.3545.1.S1_at	Y14809.1	TC22310	985	2541	1684	2.58	1.71	beta-carotene hydroxylase
Les.4264.1.A1_s_at	L24012.1	TC22590	206	526	455	2.56	2.21	gamma-aminobutyrate transaminase subunit precursor isozyme 3
Les.31.1.S1_s_at	AF110518.1	NP00011	711	1816	1198	2.55	1.68	ethylene-inducible CTR1-like protein kinase
Les.2667.2.S1_at	AW621251	TC22610	1562	3987	4038	2.55	2.58	Transcribed locus, weakly similar to NP_001042221.1 [Oryza sativa (japonica cultivar-group)]
Les.3818.1.S1_at	AF502085.1	TC21752	39	99	123	2.53	3.15	ethylene responsive element binding protein
LesAffx.58497.1.S1_at	BM411367	TC23480	40	100	141	2.53	3.54	---
Les.3667.1.S1_at	U13054.1	TC21851	733	1853	4922	2.53	6.72	endo-1,4-beta-glucanase precursor
Les.4335.2.S1_at	AW931003	TC22074	683	1721	1288	2.52	1.89	Pathogenesis-related protein P2
Les.5339.1.S1_at	BT013735.1	TC23231	682	1705	2841	2.5	4.17	Lycopersicon esculentum clone 132595F, mRNA sequence
Les.4999.1.S1_at	BT013077.1	TC23341	1978	4895	4582	2.47	2.32	Lycopersicon esculentum clone 114334F, mRNA sequence
LesAffx.22026.1.S1_at	AW218437	TC23971	877	2148	2112	2.45	2.41	Transcribed locus, weakly similar to NP_001043479.1 [Oryza sativa (japonica cultivar-group)]
Les.2560.1.S1_at	CN384809	TC22909	2172	5319	6149	2.45	2.83	gene product (aa 1-295)
LesAffx.3299.1.S1_at	AI486708	TC23796	178	435	185	2.44	1.04	Transcribed locus, weakly similar to NP_194461.1 factor/ zinc ion binding [Arabidopsis thaliana]

LesAffx.56070.1.S1_at	BF113259	TC23846	300	733	631	2.44	2.1	Transcribed locus, weakly similar to NP_001043915.1 [Oryza sativa (japonica cultivar-group)]
Les.2344.1.A1_at	BG631466	TC23883	5	13	10	2.44	1.91	Transcribed locus
LesAffx.65156.1.A1_at	CD003450	TC23971	21	50	75	2.44	3.64	Transcribed locus
Les.1020.1.A1_at	BG628781	TC23783	340	829	746	2.44	2.19	Transcribed locus, weakly similar to XP_001108540.1 similar to phosphatidylinositol-3-phosphate/phosphatidylinositol 5-kinase, type III isoform 2 isoform 4 [Macaca mulatta]
LesAffx.29757.2.S1_at	AI898522	TC23303	1062	2578	4035	2.43	3.8	Transcribed locus, moderately similar to NP_001062087.1 [Oryza sativa (japonica cultivar-group)]
Les.5530.1.S1_at	BT014086.1	TC23075	637	1539	1987	2.42	3.12	Lycopersicon esculentum clone 133190R, mRNA sequence
LesAffx.47116.1.S1_at	AW224087	TC23403	1031	2487	2725	2.41	2.64	Transcribed locus, weakly similar to XP_001089495.1 cystinosis, nephropathic isoform 1 [Macaca mulatta]
Les.546.1.A1_at	AY157063.1	TC22025	747	1797	2131	2.41	2.85	WRKY transcription factor IId-4
Les.1617.3.A1_s_at	BG627478	TC21950	104	250	153	2.41	1.47	Lycopersicon esculentum clone 114145F, mRNA sequence
LesAffx.42260.1.S1_at	AW441621	AW44162	3	6	4	2.4	1.71	---
Les.5855.1.S1_at	BT014384.1	TC22983	1523	3651	4905	2.4	3.22	Lycopersicon esculentum clone 133681F, mRNA sequence
Les.1899.1.A1_at	BG630030	TC24035	11	27	21	2.39	1.82	Transcribed locus
LesAffx.62420.1.S1_at	AW218614	TC22371	401	955	894	2.38	2.23	Transcribed locus, weakly similar to NP_001042177.1 [Oryza sativa (japonica cultivar-group)]
LesAffx.29757.2.A1_at	AI898522	TC23303	148	352	489	2.38	3.31	Transcribed locus, moderately similar to NP_001062087.1 [Oryza sativa (japonica cultivar-group)]
LesAffx.71623.1.S1_at	AW035747	TC24095	1918	4554	5064	2.37	2.64	Transcribed locus, weakly similar to NP_001041763.1 [Oryza sativa (japonica cultivar-group)]
LesAffx.32607.1.S1_at	BI206284	TC23179	13	30	19	2.37	1.5	---
LesAffx.22297.1.S1_at	AW041000	TC21723	539	1280	1460	2.37	2.71	CYP710A7 mRNA for sterol 22-desaturase
LesAffx.33082.1.S1_at	BG133383	TC23204	22	51	145	2.36	6.7	---
LesAffx.51158.2.S1_at	AI898095	TC23014	415	979	1399	2.36	3.37	Transcribed locus, weakly similar to NP_001048008.1 [Oryza sativa (japonica cultivar-group)]
LesAffx.40648.1.S1_at	BE461455	TC24357	606	1429	1392	2.36	2.3	Transcribed locus, weakly similar to NP_001043479.1 [Oryza sativa (japonica cultivar-group)]
LesAffx.6125.1.S1_at	CK468687	TC22217	171	403	299	2.36	1.74	Transcribed locus, weakly similar to NP_001042057.1 [Oryza sativa (japonica cultivar-group)]
LesAffx.4116.1.S1_at	BI933542	TC23165	173	408	756	2.35	4.36	Transcribed locus, weakly similar to NP_001047564.1 [Oryza sativa (japonica cultivar-group)]
LesAffx.60006.1.S1_at	BG643009	TC22674	134	313	763	2.34	5.7	Transcribed locus, weakly similar to NP_001055441.1 [Oryza sativa (japonica cultivar-group)]

Les.909.1.A1_at	BG628078	TC23979	21	50	100	2.33	4.69	Transcribed locus
Les.5130.1.S1_at	BT013342.1	TC23433	272	633	818	2.33	3.01	Lycopersicon esculentum clone 135145F, mRNA sequence
Les.4233.2.A1_at	BG628148	TC23601	252	586	774	2.32	3.07	Iron superoxide dismutase
Les.5672.1.S1_at	BT014363.1	TC23335	50	116	77	2.32	1.55	Lycopersicon esculentum clone 133649F, mRNA sequence
LesAffx.62232.1.S1_at	AW649113	TC23914	39	91	129	2.32	3.27	Transcribed locus, weakly similar to NP_001048167.1 [Oryza sativa (japonica cultivar-group)]
Les.1431.1.A1_at	BG631562	TC23902	298	690	1181	2.32	3.97	Transcribed locus, moderately similar to NP_001043710.1 [Oryza sativa (japonica cultivar-group)]
Les.4233.1.S1_at	BM412148	TC23601	155	360	461	2.32	2.97	Iron superoxide dismutase
Les.1826.1.A1_at	BG629397	TC23561	173	401	636	2.31	3.67	Transcribed locus
LesAffx.51158.1.S1_at	AI897068	TC23014	446	1028	1367	2.3	3.07	Transcribed locus, weakly similar to NP_001048008.1 [Oryza sativa (japonica cultivar-group)]
LesAffx.3299.1.A1_at	BG130520	TC22413	335	771	345	2.3	1.03	Transcribed locus, weakly similar to NP_194461.1 factor/ zinc ion binding [Arabidopsis thaliana]
LesAffx.52595.1.S1_at	AI778688	TC22841	462	1061	1078	2.3	2.33	Transcribed locus, weakly similar to XP_001103792.1 ERO1-like isoform 1 [Macaca mulatta]
Les.5091.1.A1_at	BT013255.1	TC22888	126	286	204	2.27	1.62	Lycopersicon esculentum clone 134773R, mRNA sequence
Les.3396.2.S1_at	AW221386	TC22284	2157	4885	9418	2.27	4.37	Transcribed locus, weakly similar to NP_001042402.1 [Oryza sativa (japonica cultivar-group)]
LesAffx.60693.1.S1_at	BI210284	TC23596	449	1016	1185	2.26	2.64	---
LesAffx.52960.1.S1_at	BI207845	TC23617	313	706	1160	2.25	3.7	---
Les.1020.1.A1_a_at	BG628781	TC23783	593	1333	1217	2.25	2.05	Transcribed locus, weakly similar to XP_001108540.1 similar to phosphatidylinositol-3-phosphate/phosphatidylinositol 5-kinase, type III isoform 2 isoform 4 [Macaca mulatta]
Les.4067.1.S1_at	AY240231.1	TC22590	1250	2810	2459	2.25	1.97	gamma-aminobutyrate transaminase subunit precursor isozyme 3
Les.2256.1.S1_at	BT014302.1	GO37635	105	235	616	2.25	5.87	Lycopersicon esculentum clone 133553F, mRNA sequence
Les.4022.1.S1_at	X94946.1	TC21814	88	196	315	2.24	3.59	proteinase inhibitor II
Les.638.1.S1_at	BT012771.1	TC21816	3065	6853	7472	2.24	2.44	Lycopersicon esculentum clone 113749F, mRNA sequence
LesAffx.51158.2.A1_at	AI898095	TC23014	230	513	650	2.23	2.83	Transcribed locus, weakly similar to NP_001048008.1 [Oryza sativa (japonica cultivar-group)]
Les.220.1.S1_at	CK716059	TC21926	1275	2835	3988	2.22	3.13	Transcribed locus, weakly similar to NP_001055699.1 [Oryza sativa (japonica cultivar-group)]
LesAffx.65156.1.S1_at	CD003450	TC23971	132	292	378	2.22	2.87	Transcribed locus
Les.5859.1.S1_at	BT013818.1	TC22390	1856	4118	4291	2.22	2.31	Lycopersicon esculentum clone 132739F, mRNA sequence
LesAffx.295.2.S1_at	AI772896	TC22228	1021	2259	2965	2.21	2.91	Transcribed locus, weakly similar to NP_001042227.1 [Oryza sativa (japonica cultivar-group)]

Les.4264.1.S1_at	L24012.1	TC22590	227	502	418	2.21	1.84	Lycopersicon esculentum (DB149) meloidogyne-induced giant cell protein mRNA, 3' end
Les.3543.1.S1_at	AJ011914.1	TC23261	145	319	215	2.2	1.49	THY5 protein
Les.4946.1.S1_at	BT012957.1	TC22395	110	243	163	2.2	1.48	Lycopersicon esculentum clone 114145F, mRNA sequence
LesAffx.3379.1.A1_at	AW039492	TC24415	624	1368	1830	2.19	2.93	Transcribed locus, moderately similar to XP_001096794.1 similar to methylcrotonoyl-Coenzyme A carboxylase 2 (beta) isoform 2 [Macaca mulatta]
Les.1764.2.A1_at	BG628826	TC23284	17	37	27	2.18	1.58	Transcribed locus, weakly similar to NP_001056202.1 [Oryza sativa (japonica cultivar-group)]
Les.4264.1.A1_at	L24012.1	TC22590	21	45	33	2.18	1.58	Lycopersicon esculentum (DB149) meloidogyne-induced giant cell protein mRNA, 3' end
Les.2988.1.S1_at	CK715255	TC22567	1399	3039	1795	2.17	1.28	Sucrose transporter
LesAffx.36837.1.S1_at	AI780027	TC23516	108	233	371	2.17	3.44	Transcribed locus, weakly similar to NP_001051963.1 [Oryza sativa (japonica cultivar-group)]
LesAffx.295.1.S1_at	BM408538	TC22228	552	1192	1361	2.16	2.46	Transcribed locus, weakly similar to NP_001042227.1 [Oryza sativa (japonica cultivar-group)]
Les.5916.1.S1_at	AJ715788.1	TC23545	442	955	1140	2.16	2.58	anaerobic basic leucine zipper protein
Les.5064.1.S1_at	BT013211.1	AI78070	712	1534	1082	2.15	1.52	Lycopersicon esculentum clone 134421R, mRNA sequence
LesAffx.71065.1.S1_at	BM413117	TC22472	1042	2241	2379	2.15	2.28	Transcribed locus, weakly similar to NP_001042582.1 [Oryza sativa (japonica cultivar-group)]
LesAffx.735.1.S1_at	AW217158	TC22685	157	338	408	2.15	2.6	Transcribed locus, weakly similar to NP_001042221.1 [Oryza sativa (japonica cultivar-group)]
Les.1189.1.A1_at	BG629967	BG62996	3	7	5	2.15	1.68	Transcribed locus, weakly similar to NP_001062206.1 [Oryza sativa (japonica cultivar-group)]
Les.4948.1.S1_at	BT012962.1	TC23041	413	886	1559	2.15	3.78	Lycopersicon esculentum clone 114152R, mRNA sequence
LesAffx.25133.1.S1_at	BI925723	TC23184	446	949	1197	2.13	2.68	Transcribed locus, weakly similar to NP_001055158.1 [Oryza sativa (japonica cultivar-group)]
Les.5395.1.S1_at	BT013835.1	TC23018	1445	3072	2770	2.13	1.92	Lycopersicon esculentum clone 132792R, mRNA sequence
LesAffx.62131.1.S1_at	BE437087	TC23252	787	1673	1027	2.12	1.3	Transcribed locus, weakly similar to XP_001081745.1 similar to threonine aldolase 1 [Rattus norvegicus]
LesAffx.12060.1.A1_at	BI204528	TC24300	153	325	360	2.12	2.36	---
Les.3396.1.A1_at	AW224542	TC22094	1485	3154	4930	2.12	3.32	Transcribed locus, weakly similar to NP_001042402.1 [Oryza sativa (japonica cultivar-group)]
LesAffx.57303.1.S1_at	AW625293	TC22186	490	1037	1501	2.12	3.07	Transcribed locus, weakly similar to NP_198885.1 protein [Arabidopsis thaliana]
Les.4040.1.A1_at	X83420.1	AW09681	373	791	1312	2.12	3.51	Alu I-fragment
LesAffx.50687.1.S1_at	AI773710	TC22384	386	817	1249	2.11	3.23	Transcribed locus, weakly similar to NP_001046905.1 [Oryza sativa (japonica cultivar-group)]

Les.3289.1.S1_at	AY240229.1	TC21835	2566	5391	5937	2.1	2.31	gamma-aminobutyrate transaminase subunit precursor isozyme 1
LesAffx.70414.1.S1_at	AW034339	TC21936	1533	3213	2717	2.1	1.77	Transcribed locus, weakly similar to NP_001045308.1 [Oryza sativa (japonica cultivar-group)]
Les.4317.1.S1_at	AW625684	TC23953	950	1990	6525	2.09	6.87	asparagine synthetase
Les.4888.1.S1_at	BI927238	TC24096	554	1159	1136	2.09	2.05	Lycopersicon esculentum clone 113905R, mRNA sequence
LesAffx.51554.1.S1_at	AW093474	GO37601	696	1452	816	2.09	1.17	---
LesAffx.68459.2.S1_at	BM411992	TC22747	105	219	452	2.08	4.29	Transcribed locus, weakly similar to NP_001051596.1 [Oryza sativa (japonica cultivar-group)]
Les.5848.2.S1_at	BM535694	TC22461	155	322	211	2.08	1.36	Transcribed locus, weakly similar to XP_001121814.1 similar to CG9009-PA [Apis mellifera]
LesAffx.66163.1.S1_at	AW222774	TC22788	404	839	857	2.08	2.12	Transcribed locus, weakly similar to NP_001046543.1 [Oryza sativa (japonica cultivar-group)]
Les.2667.3.S1_at	BE433811	TC23595	684	1416	1291	2.07	1.89	Transcribed locus, weakly similar to NP_001042221.1 [Oryza sativa (japonica cultivar-group)]
LesAffx.26180.1.S1_at	BG132890	BG13289	272	557	1188	2.05	4.36	---
LesAffx.57010.1.S1_at	AI898096	TC21847	336	685	769	2.04	2.29	Transcribed locus, weakly similar to NP_001041759.1 [Oryza sativa (japonica cultivar-group)]
LesAffx.31022.1.A1_at	AI897508	TC23408	2932	5980	5520	2.04	1.88	Transcribed locus, weakly similar to NP_001053420.1 [Oryza sativa (japonica cultivar-group)]
Les.3869.1.S1_at	BG626913	TC24136	254	518	776	2.04	3.05	Lycopersicon esculentum clone 133624F, mRNA sequence
Les.2353.1.A1_at	BG629059	TC23606	18	36	24	2.03	1.36	Transcribed locus
Les.5152.1.S1_at	BT013393.1	TC24469	250	508	747	2.03	2.99	Lycopersicon esculentum clone 135647R, mRNA sequence
LesAffx.52437.1.S1_at	BI925563	TC23341	345	699	474	2.03	1.38	Transcribed locus, weakly similar to NP_001044627.1 [Oryza sativa (japonica cultivar-group)]
Les.3526.1.S1_a_at	AF198259.1	TC22267	474	951	1064	2.01	2.24	calmodulin-binding diacylglycerol kinase
Les.5958.1.S1_at	CN384731	TC23315	469	940	1308	2	2.79	Transcribed locus, weakly similar to NP_998286.2 kinase CKM3 [Danio rerio]
LesAffx.60722.1.S1_at	AW092522	TC22954	515	1028	1068	2	2.07	Transcribed locus, weakly similar to NP_001046772.1 [Oryza sativa (japonica cultivar-group)]
LesAffx.60242.1.S1_at	BI207558	TC23660	318	635	746	2	2.34	Transcribed locus, moderately similar to NP_001058564.1 [Oryza sativa (japonica cultivar-group)]
Les.5177.1.S1_at	BT013446.1	TC24190	28	43	910	1.54	32.93	Lycopersicon esculentum clone 132101F, mRNA sequence
LesAffx.59842.1.S1_at	BI205718	TC21823	104	128	1997	1.23	19.21	Transcribed locus, weakly similar to NP_001042297.1 [Oryza sativa (japonica cultivar-group)]
LesAffx.8850.1.S1_at	AW034398	TC22181	52	81	667	1.56	12.85	Subtilisin-like protease
Les.1.1.S1_at	AF154421.1	TC21715	917	1503	7080	1.64	7.72	beta-galactosidase
Les.63.1.S1_at	AF049900.1	TC22864	47	35	320	0.74	6.81	gibberellin 20-oxidase-3

Les.4346.1.S1_at	AF020390.2	TC21717	376	505	2547	1.34	6.77	ss-galactosidase
LesAffx.56137.1.A1_at	AI490099	TC22599	81	136	472	1.67	5.79	Transcribed locus, weakly similar to NP_001041909.1 [Oryza sativa (japonica cultivar-group)]
LesAffx.56137.1.S1_at	AI490099	TC22599	353	570	2015	1.62	5.71	Transcribed locus, weakly similar to NP_001041909.1 [Oryza sativa (japonica cultivar-group)]
LesAffx.17773.1.S1_at	BF113622	DB69166	652	827	3604	1.27	5.52	Transcribed locus, weakly similar to NP_001045544.1 [Oryza sativa (japonica cultivar-group)]
Les.4026.1.S1_at	AY155579.1	TC21731	566	787	2833	1.39	5	xyloglucan-specific fungal endoglucanase inhibitor protein precursor
LesAffx.3455.2.S1_at	CN385030	TC23177	35	63	168	1.81	4.83	Transcribed locus, weakly similar to NP_001042297.1 [Oryza sativa (japonica cultivar-group)]
LesAffx.34771.1.S1_at	AW930136	AW93013	572	958	2729	1.68	4.77	---
Les.5884.1.S1_at	CK716273	TC21763	1114	2204	5316	1.98	4.77	Transcribed locus, weakly similar to NP_173228.1 inhibitor [Arabidopsis thaliana]
Les.3232.1.A1_at	BG627277	TC21879	55	66	258	1.2	4.71	Transcribed locus, weakly similar to NP_001043730.1 [Oryza sativa (japonica cultivar-group)]
LesAffx.8720.1.S1_at	BI921446	BI92223	107	164	501	1.53	4.66	Transcribed locus, weakly similar to NP_663449.2 P450-like [Mus musculus]
Les.3610.1.S1_at	BG629712	TC23477	96	165	444	1.72	4.62	glycine rich protein
Les.3575.1.S1_at	U89256.1	TC22509	45	83	207	1.85	4.62	Pti5
Les.4693.1.S1_at	M69247.1	TC23231	95	161	441	1.68	4.61	pathogenesis-related protein P4
Les.3476.1.S1_at	AF179246.1	TC21724	23	15	103	0.67	4.53	1-aminocyclopropane-1-carboxylate synthase 5
LesAffx.65135.1.S1_at	CK468698	TC23660	19	23	82	1.26	4.4	Transcribed locus
LesAffx.62906.1.S1_at	BI923447	TC23590	84	161	367	1.91	4.36	Transcribed locus, weakly similar to NP_065266.1 translocase [Mus musculus]
Les.1945.1.A1_at	BG630386	BG63038	318	500	1351	1.57	4.25	Transcribed locus
LesAffx.62549.1.S1_at	AW649200	TC24034	383	533	1622	1.39	4.24	Transcribed locus, weakly similar to NP_001042875.1 [Oryza sativa (japonica cultivar-group)]
LesAffx.8720.2.S1_at	AI895030	TC22019	201	314	848	1.56	4.22	Transcribed locus, weakly similar to NP_663449.2 P450-like [Mus musculus]
Les.5051.1.S1_at	BT013182.1	TC22934	132	260	546	1.97	4.14	Lycopersicon esculentum clone 134258F, mRNA sequence
LesAffx.68047.1.S1_at	BM535639	TC21800	62	70	255	1.13	4.12	Transcribed locus, weakly similar to NP_001043730.1 [Oryza sativa (japonica cultivar-group)]
LesAffx.68092.1.S1_at	BE459012	TC22815	176	224	712	1.27	4.03	Transcribed locus, weakly similar to NP_001044783.1 [Oryza sativa (japonica cultivar-group)]
Les.1350.1.A1_at	BG631017	TC24319	109	94	434	0.86	3.98	Transcribed locus
Les.5711.1.S1_at	BT014429.1	TC21831	199	302	776	1.52	3.9	Lycopersicon esculentum clone 133747F, mRNA sequence
Les.559.1.A1_at	BG626246	TC22601	217	380	841	1.75	3.88	Transcribed locus, weakly similar to NP_001043106.1 [Oryza sativa (japonica cultivar-group)]
Les.5759.1.S1_at	BM956714	TC22993	305	422	1181	1.38	3.88	Lycopersicon esculentum clone 133897F, mRNA sequence
Les.3693.1.S1_at	AF426174.1	TC22813	15	18	57	1.21	3.82	blind

Les.5934.1.S1_at	AI895164	TC21861	166	207	602	1.25	3.62	Transcribed locus, weakly similar to NP_001047927.1 [Oryza sativa (japonica cultivar-group)]
Les.3706.1.S1_at	AF022014.1	TC21953	333	453	1192	1.36	3.57	IAA3 protein
LesAffx.56237.1.S1_at	AW218110	TC22223	183	175	649	0.96	3.54	Transcribed locus, weakly similar to NP_001041856.1 [Oryza sativa (japonica cultivar-group)]
LesAffx.57437.1.S1_at	BI928709	TC21886	428	566	1500	1.32	3.51	Transcribed locus, weakly similar to NP_001042998.1 [Oryza sativa (japonica cultivar-group)]
LesAffx.3253.1.S1_at	BM412445	TC21903	69	115	240	1.67	3.5	Transcribed locus, weakly similar to XP_001091435.1 similar to cytochrome P450, family 26, subfamily A, polypeptide 1 isoform 1 isoform 3 [Macaca mulatta]
Les.3632.1.S1_at	U37840.1	TC21802	1594	2394	5434	1.5	3.41	lipoxygenase
Les.3583.1.A1_at	Y15846.1	TC22445	122	155	414	1.27	3.39	TSI-1 protein
Les.4040.1.S1_at	X83420.1	AW09681	1816	3562	6100	1.96	3.36	Alu I-fragment
Les.3706.1.A1_at	AF022014.1	TC21953	119	166	399	1.39	3.35	IAA3 protein
LesAffx.24042.1.S1_at	CN385197	DB67876	391	555	1310	1.42	3.35	Transcribed locus, weakly similar to NP_663449.2 P450-like [Mus musculus]
LesAffx.59592.1.S1_at	BI210118	TC22019	22	16	72	0.72	3.31	Transcribed locus, weakly similar to NP_065956.1 dehydrogenase 14 (all-trans and 9-cis) [Homo sapiens]
LesAffx.58326.1.A1_at	CD002272	TC23714	461	717	1523	1.55	3.3	Transcribed locus, weakly similar to NP_001042998.1 [Oryza sativa (japonica cultivar-group)]
LesAffx.59622.1.S1_at	BI210866	TC22863	344	368	1122	1.07	3.27	Transcribed locus, weakly similar to NP_565922.1 (EARLY FLOWERING 4) [Arabidopsis thaliana]
LesAffx.15986.1.S1_at	CK715617	TC23538	43	54	138	1.26	3.25	Transcribed locus, weakly similar to XP_001116334.1 myo-inositol oxygenase [Macaca mulatta]
LesAffx.15353.1.S1_at	BM967389	BM96738	57	62	184	1.1	3.24	Transcribed locus, weakly similar to NP_001046337.1 [Oryza sativa (japonica cultivar-group)]
LesAffx.62670.1.S1_at	BF113967	TC22120	790	1051	2489	1.33	3.15	Transcribed locus, weakly similar to NP_565423.1 protein [Arabidopsis thaliana]
LesAffx.3455.1.S1_at	BI421254	TC23177	22	37	70	1.68	3.14	Transcribed locus, weakly similar to NP_001042297.1 [Oryza sativa (japonica cultivar-group)]
Les.3042.2.S1_at	AW934591	BE46335	59	84	184	1.44	3.14	Transcribed locus, weakly similar to NP_001062055.1 [Oryza sativa (japonica cultivar-group)]
LesAffx.44422.1.S1_at	AW441968	TC21789	475	865	1485	1.82	3.13	Transcribed locus, weakly similar to NP_001041874.1 [Oryza sativa (japonica cultivar-group)]
Les.54.1.S1_at	AW218809	TC22203	97	141	302	1.46	3.12	PR-1a1 protein
LesAffx.62690.1.S1_at	BI206793	TC22770	516	829	1562	1.61	3.03	Transcribed locus, weakly similar to NP_001043439.1 [Oryza sativa (japonica cultivar-group)]
Les.4625.1.S1_at	BT013972.1	AW03556	1310	2326	3957	1.77	3.02	Lycopersicon esculentum clone 133016F, mRNA sequence
Les.3707.1.S1_at	AF022013.1	TC24298	85	54	254	0.64	3	IAA2 protein

LesAffx.56797.1.S1_at	BG127803	AW21639	313	361	937	1.15	2.99	Transcribed locus, weakly similar to NP_001041856.1 [Oryza sativa (japonica cultivar-group)]
LesAffx.17017.1.S1_at	CN384671	TC24170	188	367	558	1.96	2.97	Transcribed locus, weakly similar to NP_186978.1 protein [Arabidopsis thaliana]
Les.2341.1.A1_at	BG734949	BG73494	23	24	68	1.02	2.93	Transcribed locus
Les.1044.1.A1_at	BG629030	TC23609	194	207	563	1.07	2.91	Transcribed locus
LesAffx.51274.1.A1_at	CN385340	TC22133	42	80	122	1.92	2.91	Transcribed locus, moderately similar to NP_001044266.1 [Oryza sativa (japonica cultivar-group)]
Les.50.1.S1_at	X79338.1	TC22030	550	682	1595	1.24	2.9	ribonuclease
			3	4	8	1.62	2.89	---
Les.3415.3.S1_at	AI779314	TC24218	1568	1287	4507	0.82	2.87	CDNA, clone: FC24DE01, HTC in fruit
Les.2708.1.S1_at	BG734625	TC21907	2283	4124	6556	1.81	2.87	Transcribed locus, moderately similar to NP_001044464.1 [Oryza sativa (japonica cultivar-group)]
LesAffx.51274.1.S1_at	CN385340	TC22133	155	299	444	1.93	2.87	Transcribed locus, moderately similar to NP_001044266.1 [Oryza sativa (japonica cultivar-group)]
LesAffx.344.10.S1_at	AI773570	AI77357	1123	1806	3195	1.61	2.84	Transcribed locus, weakly similar to XP_001089325.1 similar to cellular repressor of E1A-stimulated genes [Macaca mulatta]
Les.2026.1.S1_at	BF051105	TC22543	673	922	1908	1.37	2.83	Transcribed locus, weakly similar to NP_001047542.1 [Oryza sativa (japonica cultivar-group)]
Les.1397.1.A1_at	BG631327	BG63132	914	761	2567	0.83	2.81	Transcribed locus, weakly similar to NP_001042152.1 [Oryza sativa (japonica cultivar-group)]
Les.2026.2.A1_at	BG631061	TC22543	305	439	850	1.44	2.79	Transcribed locus, weakly similar to NP_001047542.1 [Oryza sativa (japonica cultivar-group)]
LesAffx.41862.1.S1_at	AW650745	TC22822	305	356	836	1.17	2.74	Transcribed locus, weakly similar to NP_001042874.1 [Oryza sativa (japonica cultivar-group)]
LesAffx.62690.2.S1_at	CD003021	CD00302	992	1489	2704	1.5	2.73	Transcribed locus, weakly similar to NP_001043439.1 [Oryza sativa (japonica cultivar-group)]
LesAffx.56797.2.S1_at	BI922633	TC22427	533	633	1432	1.19	2.68	Transcribed locus, weakly similar to NP_001041856.1 [Oryza sativa (japonica cultivar-group)]
LesAffx.3244.2.S1_at	CD002790	TC22222	438	644	1172	1.47	2.67	Transcribed locus, weakly similar to NP_001045327.1 [Oryza sativa (japonica cultivar-group)]
Les.129.1.S1_at	AF317515.1	TC21823	38	68	101	1.81	2.67	divinyl ether synthase
LesAffx.64333.1.S1_at	CN384866	TC21750	2898	2047	7730	0.71	2.67	Transcribed locus, weakly similar to NP_565383.1 protein [Arabidopsis thaliana]
Les.2859.2.A1_at	BG735561	TC21767	784	1347	2084	1.72	2.66	Transcribed locus, weakly similar to NP_001042083.1 [Oryza sativa (japonica cultivar-group)]
Les.4254.1.S1_at	BG631257	TC24002	2565	4444	6818	1.73	2.66	Transcribed locus, moderately similar to NP_001061342.1 [Oryza sativa (japonica cultivar-group)]

LesAffx.269.2.S1_at	AW039256	TC22480	1115	2216	2959	1.99	2.66	Transcribed locus, weakly similar to XP_001082734.1 ATP-binding cassette, sub-family B, member 10 [Macaca mulatta]
Les.4222.1.S1_at	AY034149.1	TC21756	130	235	344	1.8	2.65	alternative oxidase 1b
Les.2759.1.S1_at	AW096587	TC22503	2598	4037	6845	1.55	2.64	Transcribed locus, strongly similar to NP_001042468.1 [Oryza sativa (japonica cultivar-group)]
LesAffx.69647.2.S1_at	AI774131	TC24417	326	345	855	1.06	2.62	---
LesAffx.62690.2.A1_at	CD003021	CD00302	270	422	701	1.56	2.6	Transcribed locus, weakly similar to NP_001043439.1 [Oryza sativa (japonica cultivar-group)]
Les.5567.1.S1_at	BT014164.1	TC22700	989	1080	2554	1.09	2.58	Lycopersicon esculentum clone 133286F, mRNA sequence
LesAffx.61848.1.S1_at	BG131606	TC23542	1030	1414	2653	1.37	2.58	Transcribed locus, weakly similar to XP_526649.2 similar to cytochrome P450 [Pan troglodytes]
Les.45.1.S1_at	X70787.1	TC21849	2529	4573	6493	1.81	2.57	pathogenesis-related protein PR P23
LesAffx.38740.1.S1_at	BM412691	TC23535	518	582	1326	1.12	2.56	Transcribed locus, weakly similar to XP_001104086.1 similar to UDP-galactose-4-epimerase isoform 2 [Macaca mulatta]
Les.1510.1.S1_at	AW030786	TC23573	918	1743	2341	1.9	2.55	Transcribed locus, weakly similar to XP_001106861.1 similar to sphingosine-1-phosphate lyase 1 [Macaca mulatta]
Les.104.1.S1_at	AJ243455.1	TC23544	28	34	72	1.18	2.55	cyclin B2
Les.5654.1.S1_at	BT014325.1	TC22622	1449	2827	3682	1.95	2.54	Lycopersicon esculentum clone 133598F, mRNA sequence
Les.2759.2.A1_at	BG629504	TC22503	1339	2118	3377	1.58	2.52	Transcribed locus, strongly similar to NP_001042468.1 [Oryza sativa (japonica cultivar-group)]
Les.5065.1.S1_at	BT013212.1	TC22270	1314	1495	3299	1.14	2.51	Lycopersicon esculentum clone 134422F, mRNA sequence
Les.293.1.S1_at	AY007560.1	TC21902	519	610	1300	1.18	2.5	hypothetical LOC543815
LesAffx.22100.1.S1_at	AW216933	TC22078	119	219	298	1.84	2.5	Transcribed locus, weakly similar to XP_001061027.1 similar to Heat shock factor protein 1 (HSF 1) (Heat shock transcription factor 1) (HSTF 1) [Rattus norvegicus]
LesAffx.62138.1.S1_at	AW737374	TC24364	110	195	273	1.77	2.48	---
LesAffx.23309.1.S1_at	AI777449	AI77744	330	393	816	1.19	2.48	---
Les.2859.1.S1_at	BG134254	TC21767	347	580	857	1.67	2.47	Transcribed locus, weakly similar to NP_001042083.1 [Oryza sativa (japonica cultivar-group)]
LesAffx.37916.1.S1_at	BM536108	TC23806	92	156	227	1.7	2.46	Transcribed locus, weakly similar to NP_001048479.1 [Oryza sativa (japonica cultivar-group)]
Les.5044.1.S1_at	BT013168.1	TC22109	1078	1736	2645	1.61	2.45	Lycopersicon esculentum clone 134231R, mRNA sequence
LesAffx.51285.1.S1_at	CN385118	TC24451	85	106	207	1.25	2.45	---
Les.4912.1.S1_at	BT012886.1	TC22592	773	1183	1887	1.53	2.44	Lycopersicon esculentum clone 113990R, mRNA sequence
			1	2	2	1.67	2.44	---
Les.2708.2.S1_at	AW218185	TC21907	1814	3174	4423	1.75	2.44	Transcribed locus, moderately similar to NP_001044464.1 [Oryza sativa (japonica cultivar-group)]

LesAffx.52594.1.S1_at	AI895802	TC22372	28	36	68	1.27	2.42	Transcribed locus, weakly similar to NP_001047179.1 [Oryza sativa (japonica cultivar-group)]
Les.23.1.S1_at	AF011555.1	TC22224	1767	3428	4264	1.94	2.41	jasmonic acid 2
Les.5591.1.S1_at	BT014216.1	TC23430	1555	2185	3697	1.4	2.38	Lycopersicon esculentum clone 133394F, mRNA sequence
Les.4803.1.S1_at	BT013418.1	TC21851	125	235	296	1.88	2.37	Lycopersicon esculentum clone 132056R, mRNA sequence
LesAffx.3244.1.S1_at	BI934720	TC22222	844	1116	1998	1.32	2.37	Transcribed locus, weakly similar to NP_001045327.1 [Oryza sativa (japonica cultivar-group)]
Les.1369.1.A1_at	BG631144	BG63114	32	47	77	1.46	2.37	Transcribed locus
Les.1568.1.A1_at	BG626849	TC24275	45	74	107	1.64	2.36	Transcribed locus
Les.5499.1.S1_at	BT014028.1	TC21914	1842	3303	4346	1.79	2.36	Lycopersicon esculentum clone 133090F, mRNA sequence
Les.1610.1.S1_at	BG735104	TC23584	1892	3085	4457	1.63	2.36	Proline dehydrogenase
Les.4523.1.S1_at	AY497475.1	TC21776	893	1700	2101	1.9	2.35	xyloglucan endotransglucosylase-hydrolase XTH5
Les.582.1.A1_at	BG628493	TC24387	163	225	381	1.38	2.34	Transcribed locus
Les.471.1.S1_at	AJ270956.1	TC22981	785	1042	1837	1.33	2.34	Lycopersicon esculentum partial mRNA for subtilisin-like protein, clone mo5-3C11/1
Les.5264.1.S1_at	BT013603.1	TC23455	1455	1432	3387	0.98	2.33	Lycopersicon esculentum clone 132370R, mRNA sequence
LesAffx.51285.1.A1_at	CN385118	TC24451	37	51	86	1.38	2.33	---
Les.1650.1.A1_at	BG627719	TC24168	1393	1918	3228	1.38	2.32	Transcribed locus, weakly similar to XP_001110187.1 similar to peroxisomal short-chain alcohol dehydrogenase isoform 7 [Macaca mulatta]
Les.1287.2.S1_at	AW621773	TC22852	810	713	1860	0.88	2.3	CDNA, clone: FC07DG10, HTC in fruit
LesAffx.64505.1.S1_at	AI894738	TC22840	863	1286	1980	1.49	2.29	Transcribed locus, weakly similar to NP_001041763.1 [Oryza sativa (japonica cultivar-group)]
LesAffx.51483.1.A1_at	CK348350	CK34835	10	16	23	1.56	2.28	---
Les.2123.2.S1_at	BM413006	TC23290	1500	2056	3412	1.37	2.28	Transcribed locus, weakly similar to XP_001109429.1 similar to hect domain and RLD 2 [Macaca mulatta]
Les.3710.1.S1_at	AJ312131.1	TC21745	154	290	350	1.88	2.27	alternaria stem canker resistance protein
LesAffx.36979.1.S1_at	AI775450	TC22380	820	1240	1861	1.51	2.27	Transcribed locus, weakly similar to XP_001104766.1 calmodulin-like 5 [Macaca mulatta]
Les.5597.1.S1_at	BT014226.1	AW09450	40	49	92	1.21	2.27	Lycopersicon esculentum clone 133421F, mRNA sequence
LesAffx.67017.1.S1_at	AW032581	TC22724	37	56	84	1.52	2.27	Transcribed locus, moderately similar to NP_001046101.1 [Oryza sativa (japonica cultivar-group)]
Les.5778.1.S1_at	BT014550.1	NP924383	1792	3458	4050	1.93	2.26	(Sodium/potassium)/proton exchanger 3
LesAffx.69647.1.S1_at	AW223794	TC24165	557	568	1257	1.02	2.26	Transcribed locus, weakly similar to NP_001043992.1 [Oryza sativa (japonica cultivar-group)]
LesAffx.38103.1.S1_at	BM410411	TC24474	409	525	918	1.29	2.25	---
LesAffx.45130.1.S1_at	BI933466	BI93346	26	38	59	1.45	2.24	---
Les.3665.1.S1_at	U23053.1	TC21734	6	5	13	0.86	2.24	polygalacturonase precursor

LesAffx.69872.1.S1_at	CN385882	TC23745	127	139	282	1.1	2.23	Transcribed locus
Les.4217.1.S1_at	Z48736.1	BI20478	4673	2933	10392	0.63	2.22	Cyp-3 gene for pre-pro-cysteine proteinase
LesAffx.60831.1.S1_at	AW928514	TC22802	56	53	124	0.95	2.22	Transcribed locus, weakly similar to NP_001042342.1 [Oryza sativa (japonica cultivar-group)]
Les.5410.1.S1_at	BT013860.1	TC22889	1955	2502	4321	1.28	2.21	Lycopersicon esculentum clone 132840F, mRNA sequence
Les.3611.1.S1_at	BI933029	TC21908	1316	1956	2907	1.49	2.21	biotin-containing subunit of methylcrotonyl-CoA carboxylase
Les.1243.1.A1_at	BG630316	TC23603	416	739	917	1.78	2.2	Transcribed locus
Les.150.1.S1_at	BT013117.1	AW65015	477	619	1049	1.3	2.2	Lycopersicon esculentum clone 114407R, mRNA sequence
LesAffx.8337.1.S1_at	AW094574	TC22963	85	134	187	1.57	2.2	Transcribed locus, weakly similar to NP_001051197.1 [Oryza sativa (japonica cultivar-group)]
Les.3071.3.S1_at	BI921398	TC23091	564	771	1237	1.37	2.19	Transcribed locus, weakly similar to XP_231524.4 similar to aminoadipate-semialdehyde synthase precursor [Rattus norvegicus]
LesAffx.11542.1.S1_at	BI922302	TC21973	75	100	164	1.34	2.18	Transcribed locus, weakly similar to NP_001044352.1 [Oryza sativa (japonica cultivar-group)]
Les.2569.1.S1_at	BI421923	TC22983	2681	5124	5844	1.91	2.18	Transcribed locus, weakly similar to NP_001041763.1 [Oryza sativa (japonica cultivar-group)]
LesAffx.1695.1.S1_at	BE435747	TC21794	92	97	200	1.06	2.18	Transcribed locus, weakly similar to NP_001042242.1 [Oryza sativa (japonica cultivar-group)]
Les.73.1.S1_at	AF022873.1	TC21721	2362	3311	5107	1.4	2.16	hypothetical LOC544136
Les.3935.1.S1_at	BT013403.1	TC21790	4181	7241	9035	1.73	2.16	Lycopersicon esculentum clone 135717R, mRNA sequence
Les.1961.1.A1_at	BG630514	BG63051	6	9	13	1.57	2.16	Transcribed locus
Les.4959.1.S1_at	BT012990.1	TC24411	271	339	584	1.25	2.16	Lycopersicon esculentum clone 114210R, mRNA sequence
Les.59.1.S1_at	AF014809.1	TC22468	3311	6201	7145	1.87	2.16	proline transporter 2
LesAffx.62570.2.S1_at	CD003040	TC24016	137	216	295	1.57	2.15	Transcribed locus, weakly similar to NP_001044685.1 [Oryza sativa (japonica cultivar-group)]
LesAffx.65515.1.S1_at	BM411507	TC23812	78	112	166	1.44	2.14	Transcribed locus, weakly similar to NP_001046014.1 [Oryza sativa (japonica cultivar-group)]
LesAffx.26054.1.S1_at	BG135770	TC21811	94	178	201	1.88	2.13	CDNA, clone: FC09AA01, HTC in fruit
LesAffx.70064.1.S1_at	BE435236	DB72318	419	514	892	1.23	2.13	Transcribed locus, weakly similar to NP_001041976.1 [Oryza sativa (japonica cultivar-group)]
Les.4356.3.S1_at	BE432137	TC23318	855	1374	1818	1.61	2.13	Cytosolic ascorbate peroxidase 2
LesAffx.70885.1.S1_at	BI422537	TC22623	529	800	1123	1.51	2.12	Transcribed locus, weakly similar to NP_001042660.1 [Oryza sativa (japonica cultivar-group)]
			6	8	14	1.22	2.12	---
LesAffx.64505.2.S1_at	AW032569	TC21897	2531	3819	5368	1.51	2.12	Transcribed locus, weakly similar to NP_001041763.1 [Oryza sativa (japonica cultivar-group)]

Les.471.1.A1_at	AJ270956.1	TC22981	279	364	591	1.31	2.12	Lycopersicon esculentum partial mRNA for subtilisin-like protein, clone mo5-3C11/1
Les.3403.2.S1_at	BI925376	TC22472	1151	1895	2434	1.65	2.11	Transcribed locus, weakly similar to NP_001046332.1 [Oryza sativa (japonica cultivar-group)]
LesAffx.18872.1.S1_at	BI204261	BI20426	355	528	748	1.49	2.11	---
Les.3042.1.S1_at	BI926173	TC23899	31	39	64	1.26	2.1	Transcribed locus, weakly similar to NP_001062055.1 [Oryza sativa (japonica cultivar-group)]
Les.4038.1.S1_at	AY081908.1	TC21781	192	351	402	1.83	2.09	Lycopersicon esculentum N-hydroxycinnamoyl-CoA:tyramine N-hydroxycinnamoyl transferase THT1-4 (THT1-4) pseudogene
LesAffx.68054.1.S1_at	AI898501	TC22471	262	264	548	1.01	2.09	Transcribed locus, weakly similar to NP_001049859.1 [Oryza sativa (japonica cultivar-group)]
LesAffx.64980.1.S1_at	CN384849	TC22156	49	69	103	1.41	2.09	CDNA, clone: FC09BG05, HTC in fruit
LesAffx.70873.1.S1_at	BG124340	TC23574	315	488	658	1.55	2.09	Transcribed locus, weakly similar to NP_201445.1 protein [Arabidopsis thaliana]
LesAffx.50480.1.S1_at	AI780229	TC21985	447	698	927	1.56	2.08	Transcribed locus, weakly similar to NP_001044365.1 [Oryza sativa (japonica cultivar-group)]
LesAffx.35587.1.S1_at	AW442015	TC23327	3113	3695	6454	1.19	2.07	Transcribed locus, weakly similar to XP_001092047.1 legumain [Macaca mulatta]
LesAffx.3253.2.S1_at	AW034203	TC22113	65	83	134	1.29	2.07	Transcribed locus, weakly similar to XP_001091435.1 similar to cytochrome P450, family 26, subfamily A, polypeptide 1 isoform 1 isoform 3 [Macaca mulatta]
LesAffx.54411.1.S1_at	CD003054	DB70399	123	147	254	1.2	2.06	Transcribed locus, weakly similar to NP_001047524.1 [Oryza sativa (japonica cultivar-group)]
Les.4356.2.S1_at	BM410801	TC23318	1416	2225	2920	1.57	2.06	Cytosolic ascorbate peroxidase 2
LesAffx.68107.1.S1_at	AI485479	TC23454	126	173	260	1.37	2.06	Transcribed locus, moderately similar to NP_001048067.1 [Oryza sativa (japonica cultivar-group)]
Les.4934.1.S1_at	BT012932.1	TC22421	169	298	348	1.76	2.06	Lycopersicon esculentum clone 114095R, mRNA sequence
LesAffx.71532.1.S1_at	AI772343	TC22607	1340	2621	2750	1.96	2.05	Transcribed locus, weakly similar to NP_001041930.1 [Oryza sativa (japonica cultivar-group)]
Les.5104.1.S1_at	BT013286.1	TC21728	1380	1949	2830	1.41	2.05	arginase 1
Les.450.1.S1_at	AJ441250.2	TC23501	390	755	799	1.94	2.05	p27KIP1-related-protein 2
Les.4355.1.S1_at	BG128350	TC23169	345	536	706	1.55	2.05	Ferredoxin-I
Les.5917.1.S1_at	AJ715790.1	TC21740	1850	2836	3780	1.53	2.04	1-aminocyclopropane-1-carboxylate oxidase
Les.7.1.S1_at	BI921853	TC21823	2474	3217	5043	1.3	2.04	homogentisate 1,2-dioxygenase
LesAffx.50270.1.S1_at	BI422773	TC22961	896	1139	1823	1.27	2.03	Transcribed locus, weakly similar to XP_001098383.1 chromosome 20 open reading frame 3 isoform 1 [Macaca mulatta]
Les.3388.2.S1_at	BM535881	TC23309	87	118	177	1.35	2.02	Transcribed locus, weakly similar to NP_001042227.1 [Oryza sativa (japonica cultivar-group)]
LesAffx.49103.1.A1_at	AW224716	TC22913	1060	1614	2142	1.52	2.02	Transcribed locus, weakly similar to NP_065615.1 acid (GABA(A)) receptor-associated protein-like 1 [Mus musculus]

Les.1061.1.A1_at	AI488184	TC23906	69	91	139	1.32	2.02	Transcribed locus, weakly similar to NP_568700.1 protein [Arabidopsis thaliana]
Les.411.1.S1_at	BI204543	TC23306	6150	11713	12396	1.9	2.02	Transcribed locus, weakly similar to NP_001046222.1 [Oryza sativa (japonica cultivar-group)]
Les.3499.1.S1_at	AF387785.1	TC21887	483	515	971	1.07	2.01	leucine-zipper transcription factor
LesAffx.63935.1.S1_at	AW442721	TC23203	117	168	234	1.45	2.01	Transcribed locus, weakly similar to XP_001098000.1 matrix metalloproteinase 8 [Macaca mulatta]
Les.170.1.S1_at	AY026344.1	TC22471	267	299	536	1.12	2.01	non-symbiotic hemoglobin class 2
LesAffx.50270.2.S1_at	BI205699	TC22751	710	891	1425	1.26	2.01	Transcribed locus, weakly similar to XP_001098383.1 chromosome 20 open reading frame 3 isoform 1 [Macaca mulatta]
LesAffx.34425.1.S1_at	BE354536	TC23085	253	400	506	1.58	2	Transcribed locus, weakly similar to NP_001044062.1 [Oryza sativa (japonica cultivar-group)]
Les.4526.1.S1_at	AY534757.1	TC21751	2391	2938	4790	1.23	2	protein phosphatase 2C
Les.2544.1.A1_at	BG629373	TC23776	835	1595	1669	1.91	2	Transcribed locus, weakly similar to NP_001042177.1 [Oryza sativa (japonica cultivar-group)]
Les.5621.1.S1_at	BT014267.1		243	302	486	1.24	2	Lycopersicon esculentum clone 133492F, mRNA sequence
Les.4300.1.A1_at	BG628954	TC21888	5624	4431	2831	0.79	0.5	Transcribed locus, weakly similar to NP_001043243.1 [Oryza sativa (japonica cultivar-group)]
Les.5510.1.S1_at	BT014047.1	TC22069	289	245	146	0.85	0.5	Lycopersicon esculentum clone 133144F, mRNA sequence
LesAffx.60168.2.S1_at	BI204956	TC22806	184	110	93	0.6	0.5	Transcribed locus, weakly similar to XP_001104442.1 similar to chromatin modifying protein 2B isoform 3 [Macaca mulatta]
LesAffx.59336.1.S1_at	BI203548	TC21953	179	129	90	0.72	0.5	Transcribed locus, weakly similar to NP_001044161.1 [Oryza sativa (japonica cultivar-group)]
Les.5493.1.S1_at	BT014016.1	AW62178	299	194	150	0.65	0.5	Lycopersicon esculentum clone 133078F, mRNA sequence
Les.4083.1.S1_at	BG735070	TC22596	8653	6378	4334	0.74	0.5	Lycopersicon esculentum clone 132756F, mRNA sequence
Les.2409.1.S1_at	BG735325	TC23979	1988	1218	994	0.61	0.5	Lycopersicon esculentum clone 133918F, mRNA sequence
LesAffx.65060.1.A1_at	CK716180	DV10448	1036	719	517	0.69	0.5	---
Les.3618.1.S1_at	U20592.1	TC21795	8791	7195	4382	0.82	0.5	hypothetical LOC544001
LesAffx.62950.1.S1_at	AI490601	TC23296	339	321	169	0.95	0.5	Transcribed locus, weakly similar to XP_001104086.1 similar to UDP-galactose-4-epimerase isoform 2 [Macaca mulatta]
Les.3748.1.S1_at	AB023388.1	TC22967	2170	1260	1080	0.58	0.5	acid phosphatase
LesAffx.10095.1.A1_at	AA824679	TC23852	2562	2026	1275	0.79	0.5	---
Les.2265.1.S1_at	BG735394	TC24162	440	343	218	0.78	0.5	Lycopersicon esculentum clone 133691F, mRNA sequence
LesAffx.44490.1.S1_at	AJ785174	TC22044	1664	964	826	0.58	0.5	Transcribed locus, weakly similar to NP_177118.1 factor [Arabidopsis thaliana]
Les.110.1.S1_at	AF154423.1	TC22330	989	631	490	0.64	0.5	TBG5 protein
Les.1394.1.A1_at	BG631315	AI77731	2486	1733	1232	0.7	0.5	Transcribed locus, strongly similar to NP_054477.1 II protein D1 [Nicotiana tabacum]

LesAffx.70144.1.S1_at	BI422600	TC22131	1251	979	619	0.78	0.5	Transcribed locus, moderately similar to NP_001067589.1 [Oryza sativa (japonica cultivar-group)]
LesAffx.66226.2.S1_at	BI205336	TC22735	412	217	204	0.53	0.49	Transcribed locus, weakly similar to NP_001041821.1 [Oryza sativa (japonica cultivar-group)]
Les.5560.1.S1_at	BT014148.1	TC22942	1599	1116	791	0.7	0.49	Lycopersicon esculentum clone 133267F, mRNA sequence
LesAffx.55337.1.S1_at	BM410261	TC22580	1196	1140	591	0.95	0.49	Transcribed locus, weakly similar to NP_001043235.1 [Oryza sativa (japonica cultivar-group)]
Les.3168.1.S1_at	BT013505.1	TC23152	913	726	451	0.8	0.49	INS-1P protein
Les.3732.1.A1_at	AF308936.1	NP28032	20	12	10	0.62	0.49	endo-beta-1,4-D-glucanase
Les.224.1.A1_at	AJ277561.1	TC21885	952	567	469	0.6	0.49	gts1 protein
Les.3290.1.S1_at	BG123573	TC22022	1029	668	506	0.65	0.49	Transcribed locus, weakly similar to XP_001093616.1 glutamic-pyruvate transaminase (alanine aminotransferase) isoform 1 [Macaca mulatta]
Les.3290.3.S1_at	BI933219	BG12945	1168	770	574	0.66	0.49	Transcribed locus, weakly similar to XP_001093616.1 glutamic-pyruvate transaminase (alanine aminotransferase) isoform 1 [Macaca mulatta]
Les.5933.1.S1_at	BG123740	TC23593	849	470	417	0.55	0.49	CDNA, clone: FC08AD03, HTC in fruit
LesAffx.71003.1.S1_at	AI779846	TC22050	614	607	301	0.99	0.49	Transcribed locus, weakly similar to NP_001054553.1 [Oryza sativa (japonica cultivar-group)]
Les.3640.1.S1_at	X95098.1	TC21838	39	21	19	0.54	0.49	ammonium transporter
Les.1997.1.S1_at	BE354113	TC22008	1163	631	569	0.54	0.49	Transcribed locus, weakly similar to NP_001737.1 precursor [Homo sapiens]
Les.3621.1.S1_at	K03290.1	BG63088	1946	1675	950	0.86	0.49	wound-induced proteinase inhibitor I prepropeptide
Les.4003.1.S1_at	BG631258	TC22516	2476	1737	1209	0.7	0.49	Lycopersicon esculentum clone 113690F, mRNA sequence
LesAffx.34986.1.S1_at	CK715664	TC23304	348	286	169	0.82	0.49	Transcribed locus, weakly similar to NP_001048569.1 [Oryza sativa (japonica cultivar-group)]
Les.3718.1.S1_at	AF384374.1	TC21922	1450	1469	705	1.01	0.49	allene oxide cyclase
Les.2944.1.A1_s_at	BG628979	TC21934	1185	907	574	0.77	0.48	Lycopersicon esculentum (DB208) meloidogyne-induced giant cell protein mRNA, 3' end
Les.2964.3.A1_at	BG626300	TC23599	630	573	305	0.91	0.48	Transcribed locus, weakly similar to NP_001042324.1 [Oryza sativa (japonica cultivar-group)]
LesAffx.64583.1.S1_at	BM409701	TC22829	539	493	260	0.91	0.48	Transcribed locus, weakly similar to XP_573107.2 hypothetical protein [Rattus norvegicus]
Les.3047.1.S1_at	BT013001.1	TC21910	210	119	101	0.57	0.48	Expansin18
Les.3477.1.S1_at	AF230198.2	TC23205	71	42	34	0.59	0.48	hypothetical LOC543641
LesAffx.69296.1.S1_at	AW092917	TC22654	2842	2221	1369	0.78	0.48	Transcribed locus, weakly similar to NP_001045764.1 [Oryza sativa (japonica cultivar-group)]
Les.3127.1.S1_at	AW092318	TC22823	3397	3033	1631	0.89	0.48	Transcribed locus, weakly similar to XP_001096387.1 similar to serine hydroxymethyltransferase 1 (soluble) isoform 1 [Macaca mulatta]

LesAffx.41703.1.A1_at	BG352060	TC23987	919	899	440	0.98	0.48	Transcribed locus, moderately similar to NP_001062462.1 [Oryza sativa (japonica cultivar-group)]
LesAffx.44490.1.A1_at	AJ785174	TC22044	1203	686	575	0.57	0.48	Transcribed locus, weakly similar to NP_177118.1 factor [Arabidopsis thaliana]
Les.4097.1.S1_at	AF022018.1	TC22516	2150	1268	1026	0.59	0.48	IAA7 protein
Les.4097.1.A1_at	AF022018.1	TC22516	679	403	323	0.59	0.48	IAA7 protein
LesAffx.3918.1.S1_at	BM410706	TC21958	1737	2154	827	1.24	0.48	Transcribed locus, weakly similar to NP_001047111.1 [Oryza sativa (japonica cultivar-group)]
Les.4849.1.S1_at	BT012755.1	TC21877	1196	1028	569	0.86	0.48	Lycopersicon esculentum clone 113699R, mRNA sequence
Les.840.1.A1_at	BG629718	TC24411	314	350	149	1.12	0.47	Transcribed locus
Les.3681.1.S1_at	AF466378.1	TC23533	884	517	418	0.58	0.47	hypothetical LOC543709
LesAffx.8708.1.S1_at	AW033860	AW03386	82	68	39	0.83	0.47	---
Les.3579.1.S1_at	Z68309.1	TC23498	264	165	124	0.63	0.47	Metallothionein-like protein
Les.1997.3.A1_at	BG630825	TC24117	985	516	464	0.52	0.47	Transcribed locus, weakly similar to NP_001737.1 precursor [Homo sapiens]
LesAffx.1084.1.S1_at	BG133220	TC23413	296	175	140	0.59	0.47	Transcribed locus, weakly similar to XP_001085031.1 similar to Werner syndrome protein [Macaca mulatta]
Les.1248.2.A1_at	BT012749.1	TC21728	613	344	288	0.56	0.47	GDP-mannose pyrophosphorylase
Les.3234.1.A1_at	BG735047	TC23423	2784	2650	1309	0.95	0.47	Metallocarboxypeptidase inhibitor
LesAffx.71535.1.S1_at	AW040299	TC23102	120	134	56	1.11	0.47	Transcribed locus, weakly similar to NP_001049750.1 [Oryza sativa (japonica cultivar-group)]
Les.4300.2.S1_at	AW217635	TC21888	12153	8997	5696	0.74	0.47	Transcribed locus, weakly similar to NP_001043243.1 [Oryza sativa (japonica cultivar-group)]
Les.490.1.A1_at	BG625891	TC22945	302	247	142	0.82	0.47	Transcribed locus, weakly similar to XP_001108145.1 similar to nucleotide binding protein-like [Macaca mulatta]
Les.4301.1.S1_at	BG128693	TC21928	3293	1880	1543	0.57	0.47	pre-plastocyanin (AA -64 to 106)
LesAffx.42825.1.S1_at	BI924900	TC23069	511	262	239	0.51	0.47	Transcribed locus, weakly similar to NP_001046792.1 [Oryza sativa (japonica cultivar-group)]
Les.1310.1.S1_at	BT013430.1	TC22042	2094	1352	976	0.65	0.47	Lycopersicon esculentum clone 132070F, mRNA sequence
LesAffx.7472.2.S1_at	AW651303	TC23954	132	77	61	0.58	0.47	Transcribed locus, weakly similar to NP_001043730.1 [Oryza sativa (japonica cultivar-group)]
Les.3266.2.S1_at	BI931445	TC23669	170	221	79	1.3	0.47	Transcribed locus, moderately similar to NP_001047794.1 [Oryza sativa (japonica cultivar-group)]
Les.2946.2.S1_at	BG130169	TC22071	3709	3559	1722	0.96	0.46	CDNA, clone: FC16CC05, HTC in fruit
Les.1936.1.S1_at	BG626997	TC21753	859	1077	399	1.25	0.46	Lycopersicon esculentum clone 133556R, mRNA sequence
Les.2063.1.A1_at	BG631371	TC24473	44	35	21	0.79	0.46	Transcribed locus
Les.2917.1.S1_at	BM412347	TC22799	425	252	196	0.59	0.46	Transcribed locus
Les.1157.1.A1_at	BG629717	BG62971	110	102	51	0.93	0.46	Transcribed locus

Les.1481.1.A1_at	BG625940	BG62594	55	52	26	0.94	0.46	Transcribed locus, weakly similar to NP_001052936.1 [Oryza sativa (japonica cultivar-group)]
LesAffx.65060.1.S1_at	CK716180	DV10448	502	326	231	0.65	0.46	---
Les.2038.1.A1_at	BG631023	TC23348	570	532	262	0.93	0.46	Transcribed locus, weakly similar to NP_199407.1 ester hydrolase/ hydrolase, acting on ester bonds [Arabidopsis thaliana]
Les.3214.1.S1_at	BG631018	TC21991	1105	616	507	0.56	0.46	Transcribed locus, strongly similar to NP_001064609.1 [Oryza sativa (japonica cultivar-group)]
LesAffx.55629.1.S1_at	BM412122	TC22287	484	425	222	0.88	0.46	Transcribed locus, weakly similar to NP_001044052.1 [Oryza sativa (japonica cultivar-group)]
Les.672.1.S1_at	BM536270	TC22195	330	202	151	0.61	0.46	Transcribed locus, weakly similar to NP_001042227.1 [Oryza sativa (japonica cultivar-group)]
Les.2944.3.S1_at	BE462526	TC21934	1451	980	663	0.67	0.46	Pto kinase interactor 1
Les.4492.2.S1_at	CK714954	TC22356	4075	2595	1862	0.64	0.46	Transcribed locus, weakly similar to XP_001111095.1 aldolase B isoform 5 [Macaca mulatta]
Les.2131.2.S1_at	BE433043	TC22249	2075	2069	947	1	0.46	Transcribed locus, weakly similar to NP_001050262.1 [Oryza sativa (japonica cultivar-group)]
Les.5523.1.S1_at	BT014071.1	TC21750	1899	1453	864	0.77	0.45	Lycopersicon esculentum clone 133171F, mRNA sequence
Les.3127.1.S1_a_at	AW092318	TC22823	2789	2542	1268	0.91	0.45	Transcribed locus, weakly similar to XP_001096387.1 similar to serine hydroxymethyltransferase 1 (soluble) isoform 1 [Macaca mulatta]
LesAffx.68547.1.S1_at	AW442497	TC22270	1339	808	609	0.6	0.45	Transcribed locus, weakly similar to NP_001043047.1 [Oryza sativa (japonica cultivar-group)]
Les.3174.1.S1_at	BT012701.1	TC22568	718	725	326	1.01	0.45	Lycopersicon esculentum clone 113575F, mRNA sequence
Les.3127.3.S1_at	AW932976	AW09372	2843	2554	1288	0.9	0.45	Transcribed locus, weakly similar to XP_001096387.1 similar to serine hydroxymethyltransferase 1 (soluble) isoform 1 [Macaca mulatta]
LesAffx.286.9.S1_at	CK716206	BP89354	3018	1783	1366	0.59	0.45	---
Les.2131.1.A1_at	BG627099	TC22249	2010	2120	905	1.05	0.45	Transcribed locus, weakly similar to NP_001050262.1 [Oryza sativa (japonica cultivar-group)]
LesAffx.17157.1.A1_at	CK715404	CK71540	331	211	149	0.64	0.45	---
LesAffx.45315.4.S1_at	AW622607	TC23315	3339	2218	1502	0.66	0.45	Transcribed locus, weakly similar to XP_001105252.1 similar to NAD(P)H:quinone oxidoreductase type 3, polypeptide A2 [Macaca mulatta]
Les.4975.1.S1_at	BT013032.1	TC23130	219	215	98	0.98	0.45	Lycopersicon esculentum clone 114280F, mRNA sequence
Les.2809.1.S1_at	BT014540.1	TC24080	660	525	296	0.8	0.45	Lycopersicon esculentum clone 133955F, mRNA sequence
LesAffx.66331.1.S1_at	BI421673	TC21932	195	211	88	1.08	0.45	Transcribed locus, weakly similar to NP_001056463.1 [Oryza sativa (japonica cultivar-group)]
Les.5738.1.S1_at	BT014475.1	TC23739	647	339	290	0.52	0.45	Lycopersicon esculentum clone 133825F, mRNA sequence

LesAffx.1276.1.S1_at	BG130074	TC23044	2035	1506	911	0.74	0.45	Transcribed locus, weakly similar to NP_001043284.1 [Oryza sativa (japonica cultivar-group)]
Les.2024.1.A1_at	BG631036	TC23275	1119	833	499	0.74	0.45	Transcribed locus, weakly similar to XP_001105201.1 fructose-1,6-bisphosphatase 1 isoform 1 [Macaca mulatta]
Les.4980.1.S1_at	BT013041.1	TC21982	818	432	365	0.53	0.45	Lycopersicon esculentum clone 114291R, mRNA sequence
Les.740.1.A1_at	BG627220	TC24320	61	35	27	0.58	0.44	Transcribed locus
Les.3594.1.S1_at	Z47980.1	TC21807	2402	1294	1068	0.54	0.44	hypothetical LOC544160
Les.2478.1.S1_a_at	AI780323	TC21757	12076	7738	5359	0.64	0.44	Pathogenesis-related protein P2
LesAffx.70088.1.S1_at	BI924873	TC22069	961	714	423	0.74	0.44	Transcribed locus, weakly similar to NP_565556.1 protein [Arabidopsis thaliana]
Les.3740.1.S1_at	X73986.1	TC24046	879	566	386	0.64	0.44	cathepsin D inhibitor protein
Les.4520.1.S1_at	BT013422.1	GO37596	6935	3680	3042	0.53	0.44	Lycopersicon esculentum clone 132061R, mRNA sequence
Les.827.1.S1_at	BG626882	TC23903	2925	1784	1281	0.61	0.44	TBG6 protein
Les.4099.1.S1_at	AY187634.1	TC21751	572	504	250	0.88	0.44	phosphoenolpyruvate carboxylase kinase 2
Les.2971.2.A1_at	BG629234	TC23191	2748	2599	1199	0.95	0.44	Wound-induced proteinase inhibitor I prepropeptide
Les.1784.2.A1_at	BG628995	TC24140	295	180	129	0.61	0.44	Transcribed locus, weakly similar to XP_001118335.1 similar to intestinal facilitative glucose transporter 7 [Macaca mulatta]
Les.4488.1.S1_at	BG628643	TC22827	2818	2991	1228	1.06	0.44	threonine deaminase
LesAffx.70112.1.S1_at	AW092445	TC22090	443	298	193	0.67	0.44	Transcribed locus, moderately similar to NP_001044346.1 [Oryza sativa (japonica cultivar-group)]
Les.4300.3.S1_at	BI932691	TC21888	11720	8379	5105	0.71	0.44	Transcribed locus, weakly similar to NP_001043243.1 [Oryza sativa (japonica cultivar-group)]
LesAffx.10095.1.S1_at	AA824679	TC23852	933	651	406	0.7	0.44	---
LesAffx.66600.1.A1_at	AW036163	TC23173	882	703	382	0.8	0.43	Transcribed locus, moderately similar to NP_001045167.1 [Oryza sativa (japonica cultivar-group)]
LesAffx.30938.1.S1_at	AJ785088	TC22113	644	366	279	0.57	0.43	Transcribed locus, weakly similar to NP_001042177.1 [Oryza sativa (japonica cultivar-group)]
LesAffx.1276.2.S1_at	AI484083	TC23044	3102	2313	1342	0.75	0.43	Transcribed locus, weakly similar to NP_001043284.1 [Oryza sativa (japonica cultivar-group)]
Les.2856.3.S1_at	BG130913	TC22367	638	528	276	0.83	0.43	Transcribed locus, weakly similar to NP_001043401.1 [Oryza sativa (japonica cultivar-group)]
Les.2095.1.A1_at	BG627157	TC23577	223	180	96	0.8	0.43	Transcribed locus
Les.2418.1.S1_at	BG735381	BG73538	228	244	98	1.07	0.43	Transcribed locus
LesAffx.58110.1.A1_at	AJ785349	BE43527	235	122	101	0.52	0.43	---
LesAffx.5822.1.S1_at	BG644005	TC24068	260	157	112	0.6	0.43	Transcribed locus, weakly similar to NP_001054370.1 [Oryza sativa (japonica cultivar-group)]
Les.4493.1.S1_at	BG734807	TC22690	2875	2105	1238	0.73	0.43	ADP/ATP translocator

LesAffx.21101.1.S1_at	BI924446	TC21935	148	94	64	0.64	0.43	Transcribed locus, weakly similar to NP_001047732.1 [Oryza sativa (japonica cultivar-group)]
Les.438.1.S1_at	AF011556.1	TC22758	1139	599	488	0.53	0.43	jasmonic acid 1
Les.5715.1.S1_at	BT014438.1	TC23114	692	393	296	0.57	0.43	Lycopersicon esculentum clone 133760F, mRNA sequence
Les.376.1.S1_at	BG627516	TC24390	7392	5286	3157	0.72	0.43	ribulose-1,5-bisphosphate carboxylase, small subunit precursor
Les.349.1.S1_at	CN385641	TC23560	174	142	74	0.81	0.43	Transcribed locus
Les.5817.1.S1_at	AJ785525		626	349	267	0.56	0.43	Lycopersicon esculentum clone 134124R, mRNA sequence
Les.1596.1.A1_at	BG627098	TC23373	2374	2004	1011	0.84	0.43	Transcribed locus, moderately similar to NP_001051136.1 [Oryza sativa (japonica cultivar-group)]
Les.3664.1.S1_at	U20590.1	TC22651	420	261	179	0.62	0.43	endo-1,4-beta-glucanase precursor
Les.314.1.A1_at	CK715040	TC22072	1491	893	633	0.6	0.42	Transcribed locus, weakly similar to XP_001084411.1 aquaporin 1 [Macaca mulatta]
LesAffx.17150.1.A1_at	AW039265	TC23237	418	238	178	0.57	0.42	Nitrite reductase
Les.3119.2.S1_at	BI927792	TC23253	312	245	132	0.79	0.42	Transcribed locus, weakly similar to XP_001094505.1 proteasome 26S non-ATPase subunit 3 isoform 4 [Macaca mulatta]
Les.4359.2.A1_at	BG630798	TC23326	130	75	55	0.58	0.42	Pathogenesis-related protein P2
Les.4886.1.S1_at	BT012836.1	BE44961	137	96	58	0.7	0.42	Lycopersicon esculentum clone 113902F, mRNA sequence
LesAffx.25097.1.S1_at	BI926331	TC22738	394	613	167	1.56	0.42	Transcribed locus, weakly similar to NP_194461.1 factor/ zinc ion binding [Arabidopsis thaliana]
LesAffx.63209.1.S1_at	AW092854	AW09285	608	328	256	0.54	0.42	---
Les.3085.1.S1_at	BM413323	TC22204	948	795	399	0.84	0.42	Transcribed locus, weakly similar to NP_001042186.1 [Oryza sativa (japonica cultivar-group)]
LesAffx.53053.1.S1_at	BI211180	TC22538	1252	1249	525	1	0.42	Transcribed locus, weakly similar to NP_001041735.1 [Oryza sativa (japonica cultivar-group)]
Les.3322.2.S1_at	AW091831	TC22662	706	413	295	0.58	0.42	Transcribed locus, moderately similar to NP_001047599.1 [Oryza sativa (japonica cultivar-group)]
Les.2299.1.A1_at	BG734604	TC23692	396	296	165	0.75	0.42	Transcribed locus, weakly similar to NP_001042472.1 [Oryza sativa (japonica cultivar-group)]
Les.1018.1.A1_at	BG628791	BG62879	276	169	115	0.61	0.42	Transcribed locus
LesAffx.41477.1.S1_at	AI782352	TC22420	1839	1491	763	0.81	0.41	Transcribed locus, weakly similar to NP_001041763.1 [Oryza sativa (japonica cultivar-group)]
LesAffx.37588.1.S1_at	BG124246	TC21902	72	53	30	0.73	0.41	Transcribed locus, weakly similar to NP_001046132.1 [Oryza sativa (japonica cultivar-group)]
Les.2888.1.S1_at	AI491181	TC22589	3139	2455	1285	0.78	0.41	Lycopersicon esculentum clone 113533R, mRNA sequence
LesAffx.68876.1.S1_at	BE436967	TC24391	4035	2556	1649	0.63	0.41	Transcribed locus, weakly similar to XP_001105188.1 similar to 5,10-methylenetetrahydrofolate reductase isoform 3 [Macaca mulatta]
LesAffx.48588.1.S1_at	AW650462	AW65046	361	290	147	0.8	0.41	---

LesAffx.21904.1.S1_at	BM536146	TC23302	310	194	126	0.63	0.41	Transcribed locus, weakly similar to NP_001048363.1 [Oryza sativa (japonica cultivar-group)]
LesAffx.68320.1.S1_at	BM535633	ES89343	914	788	370	0.86	0.4	Transcribed locus, weakly similar to NP_001065587.1 [Oryza sativa (japonica cultivar-group)]
Les.3214.2.S1_at	BF050423	TC21991	724	396	292	0.55	0.4	Transcribed locus, strongly similar to NP_001064609.1 [Oryza sativa (japonica cultivar-group)]
Les.3070.1.S1_at	BG126941	TC22028	443	467	179	1.05	0.4	Transcribed locus, weakly similar to NP_001042269.1 [Oryza sativa (japonica cultivar-group)]
Les.3297.3.S1_at	BM410357	TC22153	249	144	100	0.58	0.4	Transcribed locus, weakly similar to NP_001043493.1 [Oryza sativa (japonica cultivar-group)]
Les.3119.3.S1_at	AW092353	TC23253	551	474	222	0.86	0.4	Transcribed locus, weakly similar to XP_001094505.1 proteasome 26S non-ATPase subunit 3 isoform 4 [Macaca mulatta]
Les.608.1.S1_at	BG628276	TC21796	5774	4080	2324	0.71	0.4	Lycopersicon esculentum clone 133776F, mRNA sequence
LesAffx.64171.2.S1_at	AI779060	TC23115	1233	875	495	0.71	0.4	Transcribed locus, moderately similar to NP_001041918.1 [Oryza sativa (japonica cultivar-group)]
Les.2934.2.S1_at	BG128445	TC22376	291	208	116	0.72	0.4	Transcribed locus, weakly similar to NP_001046132.1 [Oryza sativa (japonica cultivar-group)]
Les.2703.1.S1_at	BG735318	TC23293	2046	1108	815	0.54	0.4	Lycopersicon esculentum clone 132109R, mRNA sequence
LesAffx.41477.2.S1_at	BI924534	TC22420	1782	1426	710	0.8	0.4	Transcribed locus, weakly similar to NP_001041763.1 [Oryza sativa (japonica cultivar-group)]
LesAffx.64062.1.S1_at	AW040768	TC22080	133	92	53	0.69	0.4	Transcribed locus, weakly similar to NP_001042589.1 [Oryza sativa (japonica cultivar-group)]
LesAffx.38304.1.S1_at	AI782644	TC21999	44	33	18	0.73	0.4	Transcribed locus, weakly similar to NP_172098.1 (DELTA 9 DESATURASE 1); iron ion binding / oxidoreductase/ stearoyl-CoA 9-desaturase [Arabidopsis thaliana]
Les.2024.2.S1_at	BG642995	AI48435	1558	925	619	0.59	0.4	Transcribed locus, weakly similar to XP_001105201.1 fructose-1,6-bisphosphatase 1 isoform 1 [Macaca mulatta]
Les.641.1.S1_at	BG626664	TC23339	1533	834	608	0.54	0.4	Lycopersicon esculentum clone 132316F, mRNA sequence
Les.84.1.S1_at	U50151.1	TC21723	968	1150	384	1.19	0.4	leucine aminopeptidase
Les.1968.1.A1_at	BG628724	TC23891	512	470	203	0.92	0.4	Transcribed locus, weakly similar to NP_001065587.1 [Oryza sativa (japonica cultivar-group)]
LesAffx.29801.1.S1_at	AI489456	TC23187	5026	5603	1985	1.11	0.39	Transcribed locus, weakly similar to NP_001042186.1 [Oryza sativa (japonica cultivar-group)]
LesAffx.64171.1.S1_at	AW622064	TC23115	1424	996	561	0.7	0.39	Transcribed locus, moderately similar to NP_001041918.1 [Oryza sativa (japonica cultivar-group)]
Les.3073.1.S1_at	AJ785415	TC21760	297	227	117	0.77	0.39	Fruit-ripening protein
Les.3741.1.S1_at	AF146691.1	TC23195	942	1160	370	1.23	0.39	Eli3 protein

Les.3180.1.S1_at	AI775150	DB70250	340	214	133	0.63	0.39	Transcribed locus, weakly similar to NP_001048006.1 [Oryza sativa (japonica cultivar-group)]
Les.3175.1.S1_at	BG626608	TC23958	3659	2252	1429	0.62	0.39	Lycopersicon esculentum clone 132976F, mRNA sequence
Les.614.1.A1_at	BG626522	BG62652	394	283	154	0.72	0.39	Transcribed locus
Les.1198.1.A1_at	BG630006	TC22356	125	87	49	0.7	0.39	Transcribed locus, weakly similar to XP_001111095.1 aldolase B isoform 5 [Macaca mulatta]
LesAffx.66600.1.S1_at	AW040689	TC23173	2302	1709	895	0.74	0.39	Transcribed locus, moderately similar to NP_001045167.1 [Oryza sativa (japonica cultivar-group)]
Les.2971.1.S1_at	AI781668	TC23191	1083	972	419	0.9	0.39	Wound-induced proteinase inhibitor I prepropeptide
LesAffx.81.2.S1_at	AW648752	TC22482	3212	2441	1244	0.76	0.39	Transcribed locus, weakly similar to XP_001090175.1 hypothetical protein LOC51507 isoform 2 [Macaca mulatta]
Les.4259.1.S1_at	BG629037	TC24424	5274	2855	2042	0.54	0.39	Transcribed locus, weakly similar to XP_001082599.1 cysteine and glycine-rich protein 2 [Macaca mulatta]
Les.3286.1.S1_at	BT013249.1	TC23425	2555	2905	989	1.14	0.39	Lycopersicon esculentum inducible plastid-lipid associated protein (CHRD)
LesAffx.64540.1.S1_at	AW441832	TC22052	1305	1254	500	0.96	0.38	Transcribed locus, moderately similar to NP_001053805.1 [Oryza sativa (japonica cultivar-group)]
LesAffx.30938.1.A1_at	AJ785088	TC22113	274	143	105	0.52	0.38	Transcribed locus, weakly similar to NP_001042177.1 [Oryza sativa (japonica cultivar-group)]
Les.3017.2.S1_at	AW738744	TC23031	2032	1434	774	0.71	0.38	Transcribed locus, weakly similar to XP_001084411.1 aquaporin 1 [Macaca mulatta]
LesAffx.71016.1.S1_at	BI923152	TC24047	935	610	356	0.65	0.38	Transcribed locus, weakly similar to NP_001052012.1 [Oryza sativa (japonica cultivar-group)]
Les.2964.1.A1_at	BG627802	TC23599	931	758	354	0.81	0.38	Transcribed locus, weakly similar to NP_001042324.1 [Oryza sativa (japonica cultivar-group)]
Les.4371.1.A1_s_at	X72731.1	TC21794	3961	2120	1507	0.54	0.38	(ERT 13) ripening-related mRNA
Les.5617.1.S1_at	BT014260.1	TC21794	6494	3390	2447	0.52	0.38	CDNA, clone: FC07DG10, HTC in fruit /// Lycopersicon esculentum clone 133464R, mRNA sequence
Les.5017.1.S1_at	BT013110.1	TC23613	405	295	153	0.73	0.38	Lycopersicon esculentum clone 114397F, mRNA sequence
Les.1478.1.S1_at	BG631500	TC23304	1117	932	419	0.83	0.37	Lycopersicon esculentum clone 114252R, mRNA sequence
Les.2173.1.A1_at	BG631444	TC23409	255	206	95	0.81	0.37	Transcribed locus, weakly similar to NP_001044532.1 [Oryza sativa (japonica cultivar-group)]
LesAffx.53857.2.S1_at	AI776010	TC23441	121	104	45	0.86	0.37	---
Les.3119.1.A1_at	AW037265	TC23253	527	433	196	0.82	0.37	Transcribed locus, weakly similar to XP_001094505.1 proteasome 26S non-ATPase subunit 3 isoform 4 [Macaca mulatta]
Les.5017.1.A1_at	BT013110.1	TC23613	1233	907	458	0.74	0.37	Lycopersicon esculentum clone 114397F, mRNA sequence
Les.4727.1.S1_at	AI776392		2720	2259	1003	0.83	0.37	Lycopersicon esculentum clone 133381R, mRNA sequence

Les.3180.2.S1_at	AI775197	TC22419	305	204	112	0.67	0.37	Transcribed locus, weakly similar to NP_001048006.1 [Oryza sativa (japonica cultivar-group)]
Les.355.1.A1_at	BG627591	TC23931	641	369	235	0.58	0.37	Transcribed locus
LesAffx.29801.1.A1_at	AI489456	TC23187	287	325	105	1.13	0.37	Transcribed locus, weakly similar to NP_001042186.1 [Oryza sativa (japonica cultivar-group)]
Les.314.2.S1_at	AW217816	BG64268	1377	780	504	0.57	0.37	Transcribed locus, weakly similar to XP_001084411.1 aquaporin 1 [Macaca mulatta]
LesAffx.14110.2.A1_at	AW932597	TC23949	214	153	78	0.72	0.36	Transcribed locus, weakly similar to NP_001042660.1 [Oryza sativa (japonica cultivar-group)]
LesAffx.13481.2.S1_at	BI921546	TC22621	1155	1193	421	1.03	0.36	Transcribed locus, weakly similar to NP_001044052.1 [Oryza sativa (japonica cultivar-group)]
Les.3180.3.A1_at	BG734557	TC23323	1153	737	417	0.64	0.36	Transcribed locus, weakly similar to NP_001048006.1 [Oryza sativa (japonica cultivar-group)]
LesAffx.13481.2.A1_at	BI921546	TC22621	2060	2202	738	1.07	0.36	Transcribed locus, weakly similar to NP_001044052.1 [Oryza sativa (japonica cultivar-group)]
LesAffx.13481.1.S1_at	BG134320	TC22621	1467	1619	519	1.1	0.35	Transcribed locus, weakly similar to NP_001044052.1 [Oryza sativa (japonica cultivar-group)]
Les.1079.3.A1_at	BG626088	GO37592	58	29	20	0.51	0.35	Transcribed locus, weakly similar to NP_001043162.1 [Oryza sativa (japonica cultivar-group)]
Les.4492.3.S1_at	CK715138	TC22356	2021	1067	711	0.53	0.35	Transcribed locus, weakly similar to XP_001111095.1 aldolase B isoform 5 [Macaca mulatta]
Les.3593.1.S1_at	BT012763.1	TC23407	2036	1602	714	0.79	0.35	TAS14 peptide (AA 1-130)
LesAffx.81.1.S1_at	BG135419	TC23216	1169	868	407	0.74	0.35	Transcribed locus, weakly similar to XP_001090175.1 hypothetical protein LOC51507 isoform 2 [Macaca mulatta]
Les.695.1.A1_at	BG626949	TC23503	693	451	240	0.65	0.35	Transcribed locus, weakly similar to NP_001042986.1 [Oryza sativa (japonica cultivar-group)]
LesAffx.1.1.S1_at	AY656838.1	TC21732	755	939	259	1.24	0.34	arginase 2
LesAffx.18330.1.S1_at	BG129699	TC24342	975	588	333	0.6	0.34	Transcribed locus, weakly similar to NP_001046074.1 [Oryza sativa (japonica cultivar-group)]
Les.5318.1.S1_at	BT013703.1	TC24128	4198	2583	1434	0.62	0.34	Lycopersicon esculentum clone 132545F, mRNA sequence
Les.506.1.A1_at	BG625957	TC22672	220	169	75	0.77	0.34	Transcribed locus
LesAffx.53857.1.S1_at	AI773690	TC23441	170	129	58	0.76	0.34	Transcribed locus, weakly similar to NP_001043047.1 [Oryza sativa (japonica cultivar-group)]
LesAffx.14110.2.S1_at	AW932597	TC23949	1775	1121	595	0.63	0.33	Transcribed locus, weakly similar to NP_001042660.1 [Oryza sativa (japonica cultivar-group)]
LesAffx.69295.1.S1_at	BI933687	TC23040	453	240	151	0.53	0.33	Transcribed locus, weakly similar to NP_001045746.1 [Oryza sativa (japonica cultivar-group)]

Les.424.1.S1_at	BG628349	TC22847	2115	1578	704	0.75	0.33	Transcribed locus, weakly similar to NP_001041744.1 [Oryza sativa (japonica cultivar-group)]
Les.3741.1.A1_at	AF146691.1	TC23195	435	458	141	1.05	0.32	Eli3 protein
Les.424.2.S1_at	AI776305	TC22847	1867	1362	602	0.73	0.32	Transcribed locus, weakly similar to NP_001041744.1 [Oryza sativa (japonica cultivar-group)]
Les.1923.1.S1_at	BG630193	TC23497	402	286	129	0.71	0.32	Transcribed locus, moderately similar to NP_001066340.1 [Oryza sativa (japonica cultivar-group)]
LesAffx.47070.1.S1_at	AW651406	AW65140	38	24	12	0.64	0.32	Transcribed locus, moderately similar to NP_001042375.1 [Oryza sativa (japonica cultivar-group)]
Les.4617.1.S1_at	BT014212.1	TC23312	1489	1145	478	0.77	0.32	Lycopersicon esculentum clone 133389F, mRNA sequence
LesAffx.3537.1.S1_at	AJ784726	TC24068	374	223	118	0.6	0.32	---
Les.4181.1.A1_at	BG630015	TC22910	185	110	58	0.6	0.31	Transcribed locus
Les.2457.1.A1_at	BG630077	TC22085	1433	1197	446	0.84	0.31	Transcribed locus
Les.5864.1.S1_at	U20596.1	TC23921	3526	2022	1096	0.57	0.31	hypothetical LOC544002
Les.228.1.S1_a_at	CK714929	TC23208	10238	7297	3165	0.71	0.31	hypothetical LOC543672
Les.2415.2.S1_at	BE431455	TC23842	1362	769	417	0.56	0.31	Transcribed locus, moderately similar to NP_001061906.1 [Oryza sativa (japonica cultivar-group)]
Les.4953.1.S1_at	BT012976.1	TC23170	800	407	244	0.51	0.31	Lycopersicon esculentum clone 114187R, mRNA sequence
Les.3983.1.S1_at	U20591.1	TC23477	104	68	31	0.65	0.3	flower-specific gamma-thionin-like protein/acidic protein precursor
Les.3299.2.A1_s_at	BG626957	TC21732	606	799	181	1.32	0.3	arginase 2
Les.3217.2.S1_at	CK575028	TC24389	7031	6638	2094	0.94	0.3	Ribulose-1,5-bisphosphate carboxylase, small subunit precursor
Les.4412.1.A1_at	BG627732	TC23503	1315	682	385	0.52	0.29	Transcribed locus, weakly similar to NP_001042986.1 [Oryza sativa (japonica cultivar-group)]
Les.4522.1.S1_at	AY497477.1	TC23773	980	1072	283	1.09	0.29	xyloglucan endotransglucosylase-hydrolase XTH6
LesAffx.3537.1.A1_at	AJ784726	TC24068	2166	1160	611	0.54	0.28	---
Les.228.1.S1_at	CK714929	TC23208	6131	4239	1724	0.69	0.28	hypothetical LOC543672
Les.810.1.S1_at	BG627575	TC22972	542	309	152	0.57	0.28	Lycopersicon esculentum clone 133744F, mRNA sequence
Les.2415.3.A1_at	BG735370	TC23842	2172	1186	605	0.55	0.28	Transcribed locus, moderately similar to NP_001061906.1 [Oryza sativa (japonica cultivar-group)]
Les.4428.1.S1_at	AW443158	DB69734	178	222	49	1.25	0.27	carbonic anhydrase
Les.4345.4.A1_x_at	BG631293	TC22725	1975	1177	533	0.6	0.27	Pathogenesis-related protein P2
Les.2316.2.A1_at	BG626036	TC22940	1125	801	302	0.71	0.27	Transcribed locus, weakly similar to NP_001044252.1 [Oryza sativa (japonica cultivar-group)]
Les.2933.1.S1_at	BT012764.1	TC23454	2715	1543	722	0.57	0.27	Lycopersicon esculentum clone 113710R, mRNA sequence
Les.5157.1.S1_at	BT013405.1	TC23882	3739	2430	987	0.65	0.26	Lycopersicon esculentum clone 135719R, mRNA sequence

Les.2316.1.S1_at	AW623527	TC22940	977	628	257	0.64	0.26	Transcribed locus, weakly similar to NP_001044252.1 [Oryza sativa (japonica cultivar-group)]
LesAffx.70136.2.S1_at	AI899680	TC23538	396	248	104	0.63	0.26	Transcribed locus, weakly similar to NP_001044898.1 [Oryza sativa (japonica cultivar-group)]
LesAffx.56221.1.S1_at	AI894713	TC24290	152	144	38	0.95	0.25	Transcribed locus, weakly similar to NP_001042068.1 [Oryza sativa (japonica cultivar-group)]
LesAffx.57251.1.S1_at	BI208760	TC22155	60	35	15	0.58	0.25	Transcribed locus, weakly similar to NP_001043814.1 [Oryza sativa (japonica cultivar-group)]
Les.4974.1.S1_at	BT013031.1	TC23280	871	702	215	0.81	0.25	Lycopersicon esculentum clone 114279F, mRNA sequence
Les.4258.1.A1_at	BG626687	TC23571	869	660	214	0.76	0.25	Transcribed locus, weakly similar to XP_001104139.1 similar to aarF domain containing kinase 1 isoform 2 [Macaca mulatta]
Les.1314.1.S1_at	BG630759	TC24293	281	344	68	1.22	0.24	Transcribed locus
Les.4345.4.A1_at	BG631293	TC22725	2337	1342	563	0.57	0.24	Pathogenesis-related protein P2
Les.1832.1.A1_at	BG629451	TC23307	1317	1472	310	1.12	0.24	Transcribed locus, moderately similar to NP_001062542.1 [Oryza sativa (japonica cultivar-group)]
Les.233.1.S1_at	M17558.1	TC23200	3068	1602	718	0.52	0.23	chlorophyll a/b-binding protein precursor
Les.3070.2.A1_at	BG629293	TC22028	790	788	182	1	0.23	Transcribed locus, weakly similar to NP_001042269.1 [Oryza sativa (japonica cultivar-group)]
Les.3924.1.S1_at	BE433308	TC23702	5583	4652	1283	0.83	0.23	Hypothetical protein LOC778199
Les.4508.2.S1_s_at	AW623685	TC23882	4124	2767	944	0.67	0.23	Lycopersicon esculentum clone 135719R, mRNA sequence
Les.4345.3.S1_x_at	AI775226	TC22725	4099	2181	937	0.53	0.23	Pathogenesis-related protein P2
Les.4258.3.S1_at	BG128812	TC23571	482	345	110	0.72	0.23	Transcribed locus, weakly similar to XP_001104139.1 similar to aarF domain containing kinase 1 isoform 2 [Macaca mulatta]
LesAffx.69609.1.S1_at	AW929283	TC23404	211	220	47	1.04	0.22	Transcribed locus, weakly similar to NP_001043990.1 [Oryza sativa (japonica cultivar-group)]
Les.3128.2.S1_s_at	BI423262	TC23347	523	426	114	0.81	0.22	Lycopersicon esculentum clone 114279F, mRNA sequence
Les.1219.1.A1_at	BG630155	TC23499	242	189	53	0.78	0.22	Transcribed locus, weakly similar to NP_001047376.1 [Oryza sativa (japonica cultivar-group)]
Les.147.1.S1_at	BG629070	TC22473	162	157	34	0.97	0.21	chlorophyll a/b-binding protein precursor
Les.4508.1.S1_s_at	AI780576	BG12438	3489	2137	702	0.61	0.2	Lycopersicon esculentum clone 135719R, mRNA sequence
Les.4923.1.S1_at	BT012912.1	TC22925	547	747	110	1.37	0.2	Lycopersicon esculentum clone 114030R, mRNA sequence
Les.3376.1.A1_at	BG630058	TC21732	3840	3331	758	0.87	0.2	CONSTANS interacting protein 1
Les.4868.1.S1_at	BT012795.1	TC22912	188	104	34	0.55	0.18	Lycopersicon esculentum clone 113794R, mRNA sequence
Les.327.1.S1_at	BT014378.1	TC22961	3584	3365	653	0.94	0.18	Lycopersicon esculentum clone 133667F, mRNA sequence
Les.3054.1.S1_at	AI773693	TC23429	4281	2208	763	0.52	0.18	Lycopersicon esculentum clone 135718R, mRNA sequence
Les.3376.2.S1_at	AW932459	TC21732	1772	1445	311	0.82	0.18	CONSTANS interacting protein 1

Les.4281.2.S1_at	BI926015	DB69892	703	477	113	0.68	0.16	Transcribed locus, weakly similar to XP_001110787.1 ectonucleoside triphosphate diphosphohydrolase 3 [Macaca mulatta]
Les.4281.1.A1_at	BG626715	TC21921	1277	899	202	0.7	0.16	Transcribed locus, weakly similar to XP_001110787.1 ectonucleoside triphosphate diphosphohydrolase 3 [Macaca mulatta]
Les.4281.3.S1_at	AI771846	TC21921	145	77	19	0.53	0.13	Transcribed locus, weakly similar to XP_001110787.1 ectonucleoside triphosphate diphosphohydrolase 3 [Macaca mulatta]
Les.2504.1.A1_at	BG628687	TC23404	576	612	59	1.06	0.1	Transcribed locus, weakly similar to NP_001043990.1 [Oryza sativa (japonica cultivar-group)]
Les.4988.1.S1_at	BT013056.1	TC22946	3222	1848	263	0.57	0.08	Lycopersicon esculentum clone 114312R, mRNA sequence
Les.4345.1.S1_at	BG629758	TC23353	5903	2979	1334	0.5	0.23	Lycopersicon esculentum clone 133385R, mRNA sequence
LesAffx.3438.1.A1_at	CN385709	TC23123	1375	694	948	0.5	0.69	Transcribed locus, weakly similar to XP_001092746.1 similar to HTPAP protein [Macaca mulatta]
LesAffx.69264.1.S1_at	BF113833	TC23903	1540	775	1364	0.5	0.89	Transcribed locus, weakly similar to NP_001047317.1 [Oryza sativa (japonica cultivar-group)]
Les.5201.1.S1_at	BT013496.1	FS19239	465	234	337	0.5	0.72	Lycopersicon esculentum clone 132182F, mRNA sequence
Les.2119.1.A1_s_at	BG626940	FS19239	1178	592	865	0.5	0.73	Lycopersicon esculentum clone 132182F, mRNA sequence
Les.5382.1.S1_at	BT013811.1	AI48888	2026	1018	1122	0.5	0.55	Lycopersicon esculentum clone 132732F, mRNA sequence
Les.842.2.S1_a_at	AI896617	TC23080	1619	813	1111	0.5	0.69	Transcribed locus, weakly similar to NP_001042177.1 [Oryza sativa (japonica cultivar-group)]
Les.4615.1.S1_at	BE462687	TC22537	3675	1844	3664	0.5	1	Lycopersicon esculentum clone 113657R, mRNA sequence
Les.1212.1.S1_at	AY584532.1	GO37246	6785	3400	4341	0.5	0.64	DWARF1/DIMINUTO
Les.1721.1.A1_at	BG628370	TC22831	398	199	265	0.5	0.67	Transcribed locus, weakly similar to NP_001065760.1 [Oryza sativa (japonica cultivar-group)]
LesAffx.70738.1.A1_at	BG630069	TC23199	1263	633	482	0.5	0.38	Transcribed locus, weakly similar to XP_001101267.1 similar to myeloblastosis proto-oncogene product isoform 2 [Macaca mulatta]
LesAffx.37212.1.A1_at	AI491145	TC23069	172	86	42	0.5	0.24	---
LesAffx.62349.1.S1_at	BI207106	TC22646	1973	985	1984	0.5	1.01	Transcribed locus, weakly similar to NP_001045429.1 [Oryza sativa (japonica cultivar-group)]
LesAffx.60159.1.S1_at	BG134596	TC23159	67	34	31	0.5	0.47	---
Les.2305.1.S1_at	AW622769	TC22654	3476	1735	1568	0.5	0.45	Transcribed locus, weakly similar to NP_001044221.1 [Oryza sativa (japonica cultivar-group)]
Les.1784.1.S1_at	BI931777	TC24140	2664	1327	1199	0.5	0.45	Transcribed locus, weakly similar to XP_001118335.1 similar to intestinal facilitative glucose transporter 7 [Macaca mulatta]
Les.3297.2.S1_at	AI773077	TC23571	820	407	191	0.5	0.23	Transcribed locus, weakly similar to NP_001043493.1 [Oryza sativa (japonica cultivar-group)]

LesAffx.20929.1.S1_at	BI206813	TC23621	416	206	138	0.5	0.33	Transcribed locus, weakly similar to NP_001051650.1 [Oryza sativa (japonica cultivar-group)]
LesAffx.58110.1.S1_at	BE435276	BE43527	1150	570	530	0.5	0.46	---
Les.3123.1.S1_at	BT013406.1	TC23016	2937	1452	1105	0.49	0.38	Lycopersicon esculentum clone 135721R, mRNA sequence
LesAffx.49935.1.S1_at	BF097557	TC23282	945	467	965	0.49	1.02	Transcribed locus, moderately similar to NP_001042088.1 [Oryza sativa (japonica cultivar-group)]
LesAffx.66879.1.A1_at	BG631312	TC22769	1426	704	1154	0.49	0.81	CDNA, clone: FC04CE09, HTC in fruit
Les.1721.2.S1_at	BF113580	TC22831	141	69	93	0.49	0.66	Transcribed locus, weakly similar to NP_001065760.1 [Oryza sativa (japonica cultivar-group)]
Les.3702.1.A1_at	AJ785373	TC23512	34	17	22	0.49	0.66	IAA10 protein
Les.4345.2.A1_x_at	AI781554	TC23670	4258	2099	889	0.49	0.21	Pathogenesis-related protein P2
Les.3742.1.S1_at	AF146690.1	TC22347	1195	588	444	0.49	0.37	Pto-responsive gene 1 protein
LesAffx.39973.1.S1_at	BI932177	DV10487	334	165	221	0.49	0.66	Transcribed locus, weakly similar to NP_001044052.1 [Oryza sativa (japonica cultivar-group)]
Les.5290.1.S1_at	BT013644.1	TC23296	2951	1445	2268	0.49	0.77	Lycopersicon esculentum clone 132449R, mRNA sequence
Les.2909.2.S1_at	BM413099	TC22288	2771	1357	970	0.49	0.35	Transcribed locus, weakly similar to NP_001041799.1 [Oryza sativa (japonica cultivar-group)]
Les.822.1.A1_at	BG629497	BG73456	839	410	422	0.49	0.5	Transcribed locus
Les.790.1.A1_at	BG627458	TC23127	293	143	133	0.49	0.45	Transcribed locus, weakly similar to NP_001052896.1 [Oryza sativa (japonica cultivar-group)]
Les.4769.1.S1_at	BT012708.1	TC21756	1314	640	967	0.49	0.74	Lycopersicon esculentum clone 113586R, mRNA sequence
LesAffx.24696.1.S1_at	AW034874	DB70637	106	52	244	0.49	2.31	Transcribed locus, weakly similar to NP_001043378.1 [Oryza sativa (japonica cultivar-group)]
Les.3479.1.S1_at	AF347614.1	TC22134	2571	1252	1850	0.49	0.72	sulfate transporter 2
Les.5670.1.S1_at	BT014359.1	TC22878	229	111	255	0.49	1.11	Lycopersicon esculentum clone 133637R, mRNA sequence
LesAffx.37212.1.S1_at	AI491145	TC23069	699	340	165	0.49	0.24	---
Les.4345.2.A1_a_at	AI781554	TC23670	3718	1802	725	0.48	0.2	Lycopersicon esculentum clone 133385R, mRNA sequence
Les.5745.1.S1_at	BT014484.1	TC22393	876	425	285	0.48	0.33	Lycopersicon esculentum clone 133838F, mRNA sequence
Les.2286.1.S1_at	BT013195.1	TC22726	2309	1119	1128	0.48	0.49	Lycopersicon esculentum clone 134347F, mRNA sequence
Les.2168.1.S1_at	BT013274.1	TC23964	3890	1884	465	0.48	0.12	Lycopersicon esculentum clone 134851R, mRNA sequence
Les.2659.1.S1_at	BE458733	TC23900	274	133	211	0.48	0.77	Transcribed locus, weakly similar to NP_001053382.1 [Oryza sativa (japonica cultivar-group)]
Les.1348.1.A1_at	BG631005	TC22489	1072	518	499	0.48	0.47	Transcribed locus, weakly similar to NP_199517.1 protein [Arabidopsis thaliana]
Les.5075.1.S1_at	BT013228.1	TC21844	1615	780	1112	0.48	0.69	Lycopersicon esculentum clone 134470F, mRNA sequence
Les.4992.1.S1_at	BT013060.1	TC23556	1607	776	703	0.48	0.44	Lycopersicon esculentum clone 114316R, mRNA sequence
Les.173.1.S1_at	AY725511.1	TC21987	2098	1013	862	0.48	0.41	LePIP1 protein

Les.5437.1.S1_at	CK715969	TC24035	1136	548	352	0.48	0.31	Lycopersicon esculentum clone 132916F, mRNA sequence
Les.5221.1.S1_at	BT013523.1	TC24473	551	265	515	0.48	0.93	Lycopersicon esculentum clone 132241R, mRNA sequence
Les.252.1.S1_at	U35643.1	DB68734	4907	2364	3795	0.48	0.77	Alpha-dioxygenase 2
Les.2012.1.S1_at	BG627650	TC23373	237	114	75	0.48	0.32	Myo-inositol-1-phosphate synthase
Les.4899.1.S1_at	BT012863.1		43	20	33	0.48	0.76	Lycopersicon esculentum clone 113951F, mRNA sequence
Les.2831.1.S1_at	BG627219	TC22500	3062	1470	2660	0.48	0.87	Lycopersicon esculentum clone 132363R, mRNA sequence
LesAffx.66705.1.S1_at	BI207021	TC23871	1777	852	867	0.48	0.49	Transcribed locus, moderately similar to NP_197266.1 protein [Arabidopsis thaliana]
LesAffx.35363.1.S1_at	AW621326	TC23283	1478	708	809	0.48	0.55	Transcribed locus, weakly similar to NP_001046337.1 [Oryza sativa (japonica cultivar-group)]
Les.1192.2.S1_at	BG643122	TC23222	942	450	744	0.48	0.79	Transcribed locus
LesAffx.885.1.S1_at	BI205930	BI20593	563	269	296	0.48	0.52	---
Les.2174.1.S1_at	CN384833	TC22746	171	82	60	0.48	0.35	Transcribed locus
Les.4929.1.S1_at	BT012922.1	TC22857	85	41	40	0.48	0.47	Lycopersicon esculentum clone 114085R, mRNA sequence
LesAffx.57293.1.S1_at	AI779530	AI77953	101	48	23	0.47	0.23	---
Les.2909.1.S1_at	AW223174	TC24474	1808	855	633	0.47	0.35	Transcribed locus, weakly similar to NP_001041799.1 [Oryza sativa (japonica cultivar-group)]
Les.4982.1.S1_at	BT013044.1	AW62120	247	117	112	0.47	0.45	Lycopersicon esculentum clone 114294R, mRNA sequence
Les.204.1.S1_at	L41126.1	TC21759	2818	1329	1106	0.47	0.39	ADP-glucose pyrophosphorylase small subunit
Les.2121.1.A1_at	AY292201.1	TC22352	1470	692	821	0.47	0.56	defense-signaling glycopeptide hormone precursor
Les.3630.1.S1_at	U50985.1	TC21720	82	39	58	0.47	0.71	pectin esterase
Les.3995.1.S1_at	AW443014	TC21771	2367	1111	548	0.47	0.23	methionine rich arabinogalactan
LesAffx.32563.1.S1_at	AI778206	TC22260	1389	652	895	0.47	0.64	Transcribed locus, weakly similar to NP_001053075.1 [Oryza sativa (japonica cultivar-group)]
Les.5161.1.S1_at	BT013412.1	AI77756	262	123	126	0.47	0.48	Lycopersicon esculentum clone 132049R, mRNA sequence
Les.1988.1.A1_at	BG630760	BG62625	1761	824	736	0.47	0.42	Transcribed locus
Les.4794.1.S1_at	BT014116.1	TC23742	2619	1221	1093	0.47	0.42	Lycopersicon esculentum clone 133221F, mRNA sequence
Les.109.1.S1_at	AF154424.1	TC22392	2621	1221	1625	0.47	0.62	TBG6 protein
Les.4707.1.S1_at	BT012714.1	TC23557	344	160	163	0.47	0.47	Lycopersicon esculentum clone 113596R, mRNA sequence
Les.2923.2.S1_at	BI935057	TC23094	2678	1245	1238	0.46	0.46	Transcribed locus, weakly similar to NP_001044052.1 [Oryza sativa (japonica cultivar-group)]
Les.1603.1.A1_at	BG630194	TC23278	214	99	91	0.46	0.43	Transcribed locus, weakly similar to NP_001043493.1 [Oryza sativa (japonica cultivar-group)]
Les.3034.1.S1_at	BG631511	TC22683	4169	1935	3335	0.46	0.8	Lycopersicon esculentum clone 114419R, mRNA sequence
Les.4696.1.S1_at	BT014502.1	TC22932	3043	1409	1421	0.46	0.47	Lycopersicon esculentum clone 133876F, mRNA sequence
LesAffx.47885.1.S1_at	CN384848	TC23827	474	219	342	0.46	0.72	Transcribed locus, weakly similar to XP_001121814.1 similar to CG9009-PA [Apis mellifera]

LesAffx.67948.1.S1_s_at	BE458482	TC22485	3995	1847	2284	0.46	0.57	IAA6 protein
Les.1465.1.S1_at	BG631822	TC23566	1273	588	345	0.46	0.27	Lycopersicon esculentum clone 132131F, mRNA sequence
Les.33.1.S1_at	AF148934.1	TC22018	2400	1107	1610	0.46	0.67	phantastica
Les.1557.1.A1_at	BG626732	TC24262	1307	603	712	0.46	0.55	Transcribed locus
Les.5559.1.S1_at	BT014147.1	BE34444	714	329	448	0.46	0.63	Lycopersicon esculentum clone 133266F, mRNA sequence
Les.4089.1.S1_at	BG642986	TC22862	1304	600	816	0.46	0.63	Transcribed locus
Les.4731.1.S1_at	BT013577.1	TC23297	3010	1383	3240	0.46	1.08	Lycopersicon esculentum clone 132322R, mRNA sequence
Les.1192.1.A1_at	BG627061	TC23222	999	459	737	0.46	0.74	Transcribed locus
LesAffx.46085.1.S1_at	AW928724	TC22038	179	82	79	0.46	0.44	Transcribed locus, weakly similar to XP_001114240.1 cytochrome P450, family 21, subfamily A, polypeptide 2 isoform 3 [Macaca mulatta]
Les.5791.1.S1_at	BT014572.1	TC22816	936	429	473	0.46	0.51	Lycopersicon esculentum clone 134027R, mRNA sequence
Les.4007.1.S1_at	BG629658	TC22991	3245	1486	1662	0.46	0.51	33 kDa oxygen-evolving protein
LesAffx.41774.1.S1_at	AW031192	TC22848	1817	832	963	0.46	0.53	Transcribed locus, weakly similar to NP_001045466.1 [Oryza sativa (japonica cultivar-group)]
Les.5309.1.S1_at	BT013691.1	TC24062	610	279	498	0.46	0.82	Lycopersicon esculentum clone 132529R, mRNA sequence
LesAffx.71503.1.S1_at	AW648310	TC22254	1764	805	1529	0.46	0.87	Transcribed locus, weakly similar to NP_001044300.1 [Oryza sativa (japonica cultivar-group)]
Les.1654.1.A1_at	BG627748	TC23489	3560	1621	1753	0.46	0.49	Transcribed locus, weakly similar to NP_001042315.1 [Oryza sativa (japonica cultivar-group)]
LesAffx.53506.1.S1_at	BI206456	TC22555	383	174	415	0.45	1.08	Transcribed locus, weakly similar to XP_001113078.1 similar to protein tyrosine phosphatase-like (proline instead of catalytic arginine), member b [Macaca mulatta]
Les.2474.2.A1_at	BG735266	TC23985	816	369	375	0.45	0.46	Transcribed locus, weakly similar to XP_001108186.1 similar to 24-dehydrocholesterol reductase precursor [Macaca mulatta]
Les.70.1.S1_at	AF000141.1	NP00014	1683	760	680	0.45	0.4	class I knotted-like homeodomain protein
Les.3016.1.S1_at	BT012757.1	TC24030	1853	837	268	0.45	0.14	Lycopersicon esculentum clone 113701F, mRNA sequence
Les.5198.1.S1_at	BT013491.1		311	140	124	0.45	0.4	Lycopersicon esculentum clone 132175F, mRNA sequence
LesAffx.71388.1.S1_at	AW034384	TC22565	375	168	285	0.45	0.76	Transcribed locus, weakly similar to NP_001042342.1 [Oryza sativa (japonica cultivar-group)]
LesAffx.24167.1.S1_at	CK715596	TC23845	108	48	87	0.45	0.81	Transcribed locus
LesAffx.26471.2.S1_at	AW218197	TC22883	108	48	60	0.45	0.55	Transcribed locus, weakly similar to NP_001054088.1 [Oryza sativa (japonica cultivar-group)]
Les.4766.1.S1_at	CN385384	TC23006	1457	652	213	0.45	0.15	Lycopersicon esculentum clone 133946F, mRNA sequence
Les.1857.1.A1_at	BG629691	TC23786	91	41	61	0.45	0.67	Transcribed locus, weakly similar to NP_001060003.1 [Oryza sativa (japonica cultivar-group)]
Les.1959.1.A1_at	BG630503	TC23129	171	76	51	0.45	0.3	Transcribed locus

Les.2547.1.A1_at	BG627349	TC23877	299	133	205	0.45	0.69	Transcribed locus, weakly similar to NP_001041979.1 [Oryza sativa (japonica cultivar-group)]
Les.3198.1.S1_at	L13654.1	TC22193	55	24	24	0.45	0.44	Lycopersicon esculentum peroxidase (TPX1)
Les.3303.1.S1_at	BT012987.1	TC23470	1889	840	1083	0.44	0.57	Lycopersicon esculentum clone 114205R, mRNA sequence
Les.208.1.S1_at	U44386.1	TC22299	2359	1048	1277	0.44	0.54	heat shock protein
Les.3297.1.S1_at	BG630303	TC21776	3104	1377	638	0.44	0.21	Transcribed locus, weakly similar to NP_001043493.1 [Oryza sativa (japonica cultivar-group)]
Les.4718.1.S1_at	BT013487.1	BI92390	181	80	199	0.44	1.1	Lycopersicon esculentum clone 132171F, mRNA sequence
Les.3322.3.S1_at	AW443800	BP90402	781	344	174	0.44	0.22	Transcribed locus, moderately similar to NP_001047599.1 [Oryza sativa (japonica cultivar-group)]
Les.4962.1.S1_at	BI204454	TC22450	1416	623	1003	0.44	0.71	Lycopersicon esculentum clone 114228R, mRNA sequence
Les.1108.1.A1_at	BG629434	TC22374	350	154	137	0.44	0.39	Transcribed locus, weakly similar to NP_001042402.1 [Oryza sativa (japonica cultivar-group)]
Les.256.1.S1_s_at	AF022017.1	TC22485	3008	1322	1637	0.44	0.54	IAA6 protein
LesAffx.26471.3.A1_at	AW218200	TC22883	578	254	326	0.44	0.56	Transcribed locus, weakly similar to NP_001054088.1 [Oryza sativa (japonica cultivar-group)]
Les.4334.3.A1_at	BG735063	TC22597	238	104	100	0.44	0.42	Transcribed locus, weakly similar to XP_001109838.1 ribulose-5-phosphate-3-epimerase isoform 5 [Macaca mulatta]
Les.4277.1.A1_at	BG628255	TC24221	3215	1408	1665	0.44	0.52	Transcribed locus, moderately similar to NP_001048891.1 [Oryza sativa (japonica cultivar-group)]
Les.5865.1.S1_at	BT013275.1	TC23540	2209	966	3530	0.44	1.6	Lycopersicon esculentum clone 134853R, mRNA sequence
Les.4703.1.S1_at	BT012803.1	TC23339	4704	2050	1964	0.44	0.42	Lycopersicon esculentum clone 113819F, mRNA sequence
LesAffx.10497.2.S1_at	AI897776	TC24435	393	171	158	0.43	0.4	Transcribed locus, weakly similar to NP_001042618.1 [Oryza sativa (japonica cultivar-group)]
Les.5952.1.S1_at	AY568716.1	TC22867	1531	664	531	0.43	0.35	acyl carrier protein
Les.4405.1.A1_at	BG630861	TC24428	1457	630	337	0.43	0.23	Transcribed locus, moderately similar to NP_001047599.1 [Oryza sativa (japonica cultivar-group)]
LesAffx.70270.1.S1_at	AI773931	TC23665	4305	1858	2320	0.43	0.54	Transcribed locus, moderately similar to NP_001046164.1 [Oryza sativa (japonica cultivar-group)]
LesAffx.9367.1.S1_at	CN385420	TC22317	638	275	170	0.43	0.27	Transcribed locus, weakly similar to NP_001049918.1 [Oryza sativa (japonica cultivar-group)]
LesAffx.10091.1.S1_at	AI487223	TC23911	156	67	41	0.43	0.26	Transcribed locus, weakly similar to NP_173851.1 [Arabidopsis thaliana]
Les.2659.2.A1_at	BG626591	TC23900	312	133	210	0.43	0.67	Transcribed locus, weakly similar to NP_001053382.1 [Oryza sativa (japonica cultivar-group)]
LesAffx.10650.1.S1_at	AW036686	TC23252	1610	683	314	0.42	0.2	CDNA, clone: FC20BC08, HTC in fruit
LesAffx.3572.1.S1_at	CK715858	DV10479	1723	731	829	0.42	0.48	---

LesAffx.53474.1.S1_at	BG133264	TC22918	1539	652	620	0.42	0.4	Transcribed locus, weakly similar to NP_001042043.1 [Oryza sativa (japonica cultivar-group)]
Les.4277.3.S1_at	BG643121	TC24221	2649	1121	1322	0.42	0.5	Transcribed locus, moderately similar to NP_001048891.1 [Oryza sativa (japonica cultivar-group)]
LesAffx.5088.1.S1_at	BI206366	BI20636	45	19	24	0.42	0.53	---
Les.1871.2.A1_at	BG629735	TC21764	1823	770	1403	0.42	0.77	Transcribed locus, weakly similar to NP_001044653.1 [Oryza sativa (japonica cultivar-group)]
Les.2092.2.S1_at	BI203427	BG63176	1563	659	1151	0.42	0.74	Transcribed locus, weakly similar to XP_001111095.1 aldolase B isoform 5 [Macaca mulatta]
LesAffx.29797.1.S1_at	AJ784483	TC23412	1762	743	154	0.42	0.09	Transcribed locus, weakly similar to NP_001042863.1 [Oryza sativa (japonica cultivar-group)]
LesAffx.56.6.S1_at	BM536076	TC23980	150	63	169	0.42	1.13	Transcribed locus, weakly similar to NP_004311.1 Ca++ transporting, fast twitch 1 isoform b [Homo sapiens]
Les.3409.1.A1_at	BG735108	GO37597	3684	1549	1014	0.42	0.28	Transcribed locus, weakly similar to NP_001044764.1 [Oryza sativa (japonica cultivar-group)]
Les.119.1.S1_at	AF218774.1	TC21978	1243	521	483	0.42	0.39	Aqp2 protein
Les.3595.1.S1_at	L76632.1	TC21885	3432	1438	2086	0.42	0.61	osmotin-like protein
Les.1549.1.A1_at	BG626692	TC23631	864	362	579	0.42	0.67	Transcribed locus
Les.4008.1.S1_a_at	BG630151	TC21741	4135	1730	2005	0.42	0.48	xyloglucan endotransglycosylase/hydrolase 16 protein
Les.1179.1.A1_at	BG629888	TC24291	461	193	336	0.42	0.73	Transcribed locus
Les.1821.1.A1_at	BG629348	BG62934	342	143	132	0.42	0.39	Transcribed locus
Les.3312.2.S1_at	BI932707	TC21843	2541	1054	1238	0.41	0.49	Transcribed locus, weakly similar to NP_001043993.1 [Oryza sativa (japonica cultivar-group)]
Les.5327.1.S1_at	BT013719.1	TC24178	931	384	356	0.41	0.38	Lycopersicon esculentum clone 132567F, mRNA sequence
LesAffx.3570.4.S1_at	AW624180	TC22612	1017	418	498	0.41	0.49	Ribosomal protein L2
LesAffx.61000.1.S1_at	BM410987	TC23050	850	349	372	0.41	0.44	Transcribed locus, weakly similar to NP_001045184.1 [Oryza sativa (japonica cultivar-group)]
Les.3064.1.S1_at	U81033.1	TC21721	2277	934	812	0.41	0.36	ADP-glucose pyrophosphorylase large subunit
LesAffx.6110.1.S1_at	BF098450	TC22721	79	32	49	0.41	0.62	---
LesAffx.26471.3.S1_at	AW218200	TC22883	2567	1048	1456	0.41	0.57	Transcribed locus, weakly similar to NP_001054088.1 [Oryza sativa (japonica cultivar-group)]
Les.1951.1.A1_at	BG630440	TC23845	4344	1772	2057	0.41	0.47	Transcribed locus, weakly similar to NP_188563.1 binding [Arabidopsis thaliana]
LesAffx.9007.1.S1_at	CN385923	GO37540	1257	512	1147	0.41	0.91	Transcribed locus, weakly similar to NP_001042158.1 [Oryza sativa (japonica cultivar-group)]
Les.1871.1.S1_at	CN385866	TC21764	2611	1060	2116	0.41	0.81	Transcribed locus, weakly similar to NP_001044653.1 [Oryza sativa (japonica cultivar-group)]

Les.2092.1.S1_at	BG626759	TC22657	3052	1238	2521	0.41	0.83	Transcribed locus, weakly similar to XP_001111095.1 aldolase B isoform 5 [Macaca mulatta]
Les.174.1.A1_at	BG627544	TC24015	289	117	42	0.41	0.15	Transcribed locus, moderately similar to NP_001045188.1 [Oryza sativa (japonica cultivar-group)]
LesAffx.24799.2.A1_at	BI209670	TC24255	404	163	146	0.4	0.36	Transcribed locus, weakly similar to NP_001045764.1 [Oryza sativa (japonica cultivar-group)]
Les.4835.1.S1_at	BT012722.1	TC24498	451	182	174	0.4	0.39	Lycopersicon esculentum clone 113649F, mRNA sequence
Les.4896.1.S1_at	BT012857.1	TC23109	2174	875	916	0.4	0.42	Lycopersicon esculentum clone 113944R, mRNA sequence
Les.5263.1.S1_at	BT013600.1	TC22756	223	90	107	0.4	0.48	Lycopersicon esculentum clone 132364F, mRNA sequence
Les.4277.2.S1_at	AW623709	TC21859	2273	912	1088	0.4	0.48	Transcribed locus, moderately similar to NP_001048891.1 [Oryza sativa (japonica cultivar-group)]
Les.24.1.S1_at	AF191823.1	TC21735	474	190	73	0.4	0.15	alpha-galactosidase
Les.3372.1.S1_at	BG631141	TC23249	1332	533	595	0.4	0.45	Lycopersicon esculentum clone 132813R, mRNA sequence
LesAffx.41816.1.S1_at	AW649474	TC22094	284	114	147	0.4	0.52	Transcribed locus, weakly similar to XP_001102886.1 hexosaminidase B isoform 1 [Macaca mulatta]
Les.1205.2.S1_at	BM536081	TC21911	805	321	93	0.4	0.12	Transcribed locus, weakly similar to NP_001059455.1 [Oryza sativa (japonica cultivar-group)]
LesAffx.37636.1.S1_at	CN384813	EG55389	90	36	39	0.4	0.44	---
Les.3094.3.S1_at	BG125458	TC23596	3360	1337	991	0.4	0.29	Transcribed locus, weakly similar to NP_001041542.1 P450, family 51, subfamily A, polypeptide 1 [Gallus gallus]
Les.3330.3.A1_at	BG629064	TC24355	2943	1169	1032	0.4	0.35	Transcribed locus, weakly similar to NP_001044764.1 [Oryza sativa (japonica cultivar-group)]
Les.1871.2.A1_a_at	BG629735	TC21764	952	378	616	0.4	0.65	Transcribed locus, weakly similar to NP_001044653.1 [Oryza sativa (japonica cultivar-group)]
Les.3701.1.S1_at	AF022022.1	TC24440	69	27	49	0.4	0.7	IAA11 protein
Les.4312.2.S1_at	BG626083	TC22795	6555	2588	741	0.39	0.11	CDNA, clone: FC02DB04, HTC in fruit
LesAffx.770.1.S1_at	BI210904	BI21090	239	94	156	0.39	0.65	---
Les.4738.1.S1_at	BT013480.1	GO37558	1878	737	1199	0.39	0.64	Lycopersicon esculentum clone 132163F, mRNA sequence
LesAffx.24799.2.S1_at	BI209670	TC24255	986	386	346	0.39	0.35	Transcribed locus, weakly similar to NP_001045764.1 [Oryza sativa (japonica cultivar-group)]
Les.4334.1.A1_at	BG626518	TC24039	2115	828	895	0.39	0.42	Transcribed locus, weakly similar to XP_001109838.1 ribulose-5-phosphate-3-epimerase isoform 5 [Macaca mulatta]
LesAffx.54718.1.S1_at	BM411779	TC22617	644	252	216	0.39	0.33	Transcribed locus, moderately similar to NP_001044227.1 [Oryza sativa (japonica cultivar-group)]
LesAffx.62334.1.S1_at	AW647879	AW64787	38	15	12	0.39	0.33	---

LesAffx.65028.1.S1_at	BG128587	TC22082	3341	1298	1290	0.39	0.39	Transcribed locus, weakly similar to NP_001054271.1 [Oryza sativa (japonica cultivar-group)]
Les.3322.1.S1_at	BI935557	TC22083	525	204	112	0.39	0.21	Transcribed locus, moderately similar to NP_001047599.1 [Oryza sativa (japonica cultivar-group)]
Les.4008.1.S1_at	BG630151	TC21741	2619	1015	1220	0.39	0.47	xyloglucan endotransglycosylase/hydrolase 16 protein
LesAffx.46550.1.S1_at	BG134870	BG13487	155	59	72	0.38	0.46	---
Les.4008.2.S1_at	BG643663	TC21741	2263	867	1039	0.38	0.46	xyloglucan endotransglycosylase/hydrolase 16 protein
LesAffx.48215.1.S1_at	AW945008	TC23968	1835	703	1102	0.38	0.6	Transcribed locus, weakly similar to NP_001043647.1 [Oryza sativa (japonica cultivar-group)]
Les.5312.1.S1_at	BT013697.1	TC22735	2597	991	656	0.38	0.25	Lycopersicon esculentum clone 132539F, mRNA sequence
LesAffx.24096.1.S1_at	AJ785329	TC23598	345	131	158	0.38	0.46	---
Les.4334.2.S1_at	BI928683	TC22597	1421	539	570	0.38	0.4	Transcribed locus, weakly similar to XP_001109838.1 ribulose-5-phosphate-3-epimerase isoform 5 [Macaca mulatta]
Les.3055.1.S1_at	AJ002590.1	TC21769	375	142	183	0.38	0.49	CycD3;3 protein
LesAffx.30807.1.S1_at	AW624957	AW62495	1784	668	638	0.37	0.36	Transcribed locus, weakly similar to XP_001116629.1 similar to fatty acid desaturase 2 isoform 2 [Macaca mulatta]
Les.4444.1.S1_s_at	BI207010	TC22444	190	71	75	0.37	0.4	CycD3;3 protein
Les.1205.3.S1_at	BM410805	TC21911	1376	508	149	0.37	0.11	Transcribed locus, weakly similar to NP_001059455.1 [Oryza sativa (japonica cultivar-group)]
Les.1197.2.A1_at	BG630003	TC23124	2356	867	1007	0.37	0.43	Transcribed locus, weakly similar to XP_001090911.1 LanC lantibiotic synthetase component C-like 2 isoform 2 [Macaca mulatta]
Les.1197.1.S1_at	BI925087	TC22471	550	201	213	0.37	0.39	Transcribed locus, weakly similar to XP_001090911.1 LanC lantibiotic synthetase component C-like 2 isoform 2 [Macaca mulatta]
LesAffx.24799.1.S1_at	CK715756	TC23394	612	224	202	0.37	0.33	Transcribed locus, weakly similar to NP_001045764.1 [Oryza sativa (japonica cultivar-group)]
LesAffx.44609.1.S1_at	BG631285	TC23469	1162	423	804	0.36	0.69	Transcribed locus, weakly similar to NP_187615.2 factor [Arabidopsis thaliana]
LesAffx.56547.2.S1_at	BI207815	TC22569	254	92	159	0.36	0.62	Transcribed locus, weakly similar to NP_001042475.1 [Oryza sativa (japonica cultivar-group)]
Les.5579.1.S1_at	BT014190.1	TC23962	769	278	241	0.36	0.31	Lycopersicon esculentum clone 133367R, mRNA sequence
LesAffx.105.1.S1_at	CK715483	TC22543	185	66	136	0.36	0.74	Transcribed locus, weakly similar to NP_001052215.1 [Oryza sativa (japonica cultivar-group)]
LesAffx.17924.1.S1_at	BG124217	TC24101	1449	514	535	0.36	0.37	Transcribed locus, weakly similar to NP_001049241.1 [Oryza sativa (japonica cultivar-group)]
Les.3330.1.S1_at	AW650121	TC24355	1469	521	512	0.35	0.35	Transcribed locus, weakly similar to NP_001044764.1 [Oryza sativa (japonica cultivar-group)]
Les.4473.1.S1_at	AJ606077.1	TC22116	1478	524	780	0.35	0.53	spe4 protein

Les.1808.1.A1_at	BG629204		9	3	7	0.35	0.72	Transcribed locus
Les.369.1.S1_at	AF059489.1	TC21922	4053	1421	1975	0.35	0.49	expansin
LesAffx.14321.1.S1_at	AW737808	AW73780	110	39	43	0.35	0.39	---
Les.6.1.S1_at	AF261141.1	TC22389	1804	625	337	0.35	0.19	hypothetical LOC543656
LesAffx.26471.1.S1_at	BG126065	TC22883	1932	668	972	0.35	0.5	Transcribed locus, weakly similar to NP_001054088.1 [Oryza sativa (japonica cultivar-group)]
Les.4987.1.S1_at	BT013055.1	TC23511	2295	791	1406	0.34	0.61	Lycopersicon esculentum clone 114311F, mRNA sequence
Les.876.1.A1_at	BG627344	TC22377	218	75	77	0.34	0.35	Transcribed locus, weakly similar to NP_001051973.1 [Oryza sativa (japonica cultivar-group)]
Les.852.1.A1_at	BG627744	TC24030	56	19	17	0.34	0.3	Transcribed locus
Les.4749.1.S1_at	BT013546.1	AI77484	1052	357	371	0.34	0.35	Lycopersicon esculentum clone 132267F, mRNA sequence
Les.3409.2.S1_at	BF050508	TC21920	1957	664	470	0.34	0.24	Transcribed locus, weakly similar to NP_001044764.1 [Oryza sativa (japonica cultivar-group)]
Les.3065.2.A1_at	BG630869	TC22543	1472	495	202	0.34	0.14	Transcribed locus, weakly similar to NP_001042793.1 [Oryza sativa (japonica cultivar-group)]
Les.4549.1.S1_at	BT013085.1	TC24365	152	51	53	0.34	0.35	Lycopersicon esculentum clone 114349R, mRNA sequence
LesAffx.33890.1.S1_at	BG134582	TC22584	160	54	44	0.33	0.27	Transcribed locus, weakly similar to NP_001067602.1 [Oryza sativa (japonica cultivar-group)]
Les.3330.2.S1_at	BE458823	TC24355	2809	939	922	0.33	0.33	Transcribed locus, weakly similar to NP_001044764.1 [Oryza sativa (japonica cultivar-group)]
LesAffx.67767.1.A1_at	AI486022	TC22922	254	85	271	0.33	1.07	Transcribed locus, weakly similar to NP_568700.1 protein [Arabidopsis thaliana]
Les.4938.1.S1_at	BT012940.1	TC22310	134	45	47	0.33	0.35	Lycopersicon esculentum clone 114108R, mRNA sequence
Les.3792.1.S1_s_at	AF249329.1	GO37419	707	234	126	0.33	0.18	Lycopersicon esculentum putative cytochrome P450
Les.479.1.S1_at	AY509122.1	TC22844	418	138	156	0.33	0.37	arachidonic acid-induced DEA1
LesAffx.9038.1.S1_at	BI930643	TC22956	345	113	165	0.33	0.48	Transcribed locus, weakly similar to NP_001042297.1 [Oryza sativa (japonica cultivar-group)]
LesAffx.41518.1.S1_at	AW930080	TC23094	461	151	177	0.33	0.39	Transcribed locus, weakly similar to NP_001050851.1 [Oryza sativa (japonica cultivar-group)]
LesAffx.37344.1.S1_at	AI897449	TC21881	472	154	103	0.33	0.22	Transcribed locus, weakly similar to NP_001042390.1 [Oryza sativa (japonica cultivar-group)]
LesAffx.48876.1.S1_at	AW622727	AW62272	97	32	76	0.32	0.78	---
Les.5832.1.S1_at	BT014414.1	TC22401	1538	494	287	0.32	0.19	Lycopersicon esculentum clone 133722R, mRNA sequence
Les.4529.1.S1_at	AY497478.1	TC21903	542	174	100	0.32	0.18	xyloglucan endotransglucosylase-hydrolase XTH7
LesAffx.16472.1.S1_at	AI774397	TC24204	424	135	192	0.32	0.45	---
Les.2626.1.S1_at	BG735008	TC22245	2277	725	651	0.32	0.29	Lycopersicon esculentum clone 132901F, mRNA sequence
Les.5850.1.S1_at	BT014189.1	TC21894	526	167	325	0.32	0.62	Lycopersicon esculentum clone 133366R, mRNA sequence

Les.4493.3.S1_at	CN385077	TC23105	595	189	197	0.32	0.33	ADP/ATP translocator
Les.1042.1.A1_at	BG629025	TC22972	280	89	302	0.32	1.08	Transcribed locus, weakly similar to NP_001042141.1 [Oryza sativa (japonica cultivar-group)]
Les.642.1.S1_at	BG626672	BG62667	229	72	59	0.32	0.26	Lycopersicon esculentum clone 132200F, mRNA sequence
LesAffx.41330.1.A1_at	CK574967	TC24064	366	115	120	0.31	0.33	Transcribed locus, weakly similar to NP_001067668.1 [Oryza sativa (japonica cultivar-group)]
Les.2529.1.A1_at	BG631274	TC24469	1575	489	971	0.31	0.62	Transcribed locus, weakly similar to NP_001041768.1 [Oryza sativa (japonica cultivar-group)]
Les.10.1.S1_at	AB010991.1	TC21912	299	92	117	0.31	0.39	3b-hydroxylase
Les.1205.1.A1_at	BG630057	TC21911	926	283	107	0.31	0.12	Transcribed locus, weakly similar to NP_001059455.1 [Oryza sativa (japonica cultivar-group)]
Les.2529.2.S1_at	BI421300	TC22334	823	250	537	0.3	0.65	Transcribed locus, weakly similar to NP_001041768.1 [Oryza sativa (japonica cultivar-group)]
LesAffx.10497.2.A1_at	AI897776	TC24435	2587	785	710	0.3	0.27	Transcribed locus, weakly similar to NP_001042618.1 [Oryza sativa (japonica cultivar-group)]
Les.3065.1.S1_at	BI929558	TC22543	965	289	140	0.3	0.15	Transcribed locus, weakly similar to NP_001042793.1 [Oryza sativa (japonica cultivar-group)]
Les.1562.1.S1_at	BG629310	TC23006	502	150	158	0.3	0.32	Lycopersicon esculentum clone 133516F, mRNA sequence
Les.4065.1.A1_at	L24019.1		42	12	14	0.3	0.33	Lycopersicon esculentum (DB166) meloidogyne-induced giant cell protein mRNA, 3' end
LesAffx.41330.2.S1_at	AI899559	TC24064	548	162	174	0.3	0.32	Transcribed locus, weakly similar to NP_001067668.1 [Oryza sativa (japonica cultivar-group)]
LesAffx.4617.1.A1_at	BG735509	TC23088	874	258	220	0.3	0.25	Transcribed locus, weakly similar to NP_001041735.1 [Oryza sativa (japonica cultivar-group)]
Les.68.2.S1_a_at	BT013727.1	TC21796	3596	1063	828	0.3	0.23	endo-beta-1,4-D-glucanase
Les.3017.3.S1_at	BE462343	TC21898	994	293	476	0.29	0.48	Transcribed locus, weakly similar to XP_001084411.1 aquaporin 1 [Macaca mulatta]
Les.3017.1.A1_at	BG629826	AI78132	1583	463	806	0.29	0.51	Transcribed locus, weakly similar to XP_001084411.1 aquaporin 1 [Macaca mulatta]
Les.3636.1.S1_at	Y11268.1	TC23558	372	108	268	0.29	0.72	endo-1,4-beta-D-glucanase
LesAffx.61034.1.S1_at	BG643132	TC21983	2125	619	1062	0.29	0.5	Transcribed locus, weakly similar to NP_001043558.1 [Oryza sativa (japonica cultivar-group)]
LesAffx.71035.1.S1_at	BI207404	TC23510	1293	376	448	0.29	0.35	Transcribed locus, weakly similar to NP_179716.1 protein [Arabidopsis thaliana]
LesAffx.4981.1.S1_at	AW623022	TC22429	5373	1556	1114	0.29	0.21	Transcribed locus, weakly similar to NP_001041979.1 [Oryza sativa (japonica cultivar-group)]
Les.5918.1.S1_at	AY224079.1	TC21718	116	34	33	0.29	0.29	ferric-chelate reductase
Les.2325.1.S1_at	BG625896	TC22401	336	97	93	0.29	0.28	yfe37 protein
LesAffx.67767.1.S1_at	BF096648	TC22922	466	134	579	0.29	1.24	Transcribed locus, weakly similar to NP_568700.1 protein [Arabidopsis thaliana]

Les.2688.1.S1_at	AJ560646.1	TC21917	561	159	192	0.28	0.34	expansin11
LesAffx.46815.1.S1_at	BI934723	TC21911	669	186	169	0.28	0.25	Hypothetical LOC544002
LesAffx.58019.1.S1_at	BI929233	TC22171	575	159	429	0.28	0.75	Transcribed locus, weakly similar to NP_001052012.1 [Oryza sativa (japonica cultivar-group)]
LesAffx.37344.2.S1_at	BG131634	TC21881	920	245	157	0.27	0.17	Transcribed locus, weakly similar to NP_001042390.1 [Oryza sativa (japonica cultivar-group)]
LesAffx.65984.1.S1_at	BG125327	GO37430	2532	675	176	0.27	0.07	Transcribed locus, weakly similar to NP_001044052.1 [Oryza sativa (japonica cultivar-group)]
LesAffx.71006.1.S1_at	BI203873	TC22346	31	8	9	0.26	0.28	Transcribed locus, weakly similar to NP_001043243.1 [Oryza sativa (japonica cultivar-group)]
LesAffx.46815.2.S1_at	BI929852	TC22829	2008	528	491	0.26	0.24	Hypothetical LOC544002
Les.4594.1.S1_at	BT012739.1	TC23704	3408	893	1824	0.26	0.54	Lycopersicon esculentum clone 113677R, mRNA sequence
Les.406.1.A1_at	BG629308	TC24082	891	233	317	0.26	0.36	Transcribed locus, weakly similar to NP_196850.1 binding [Arabidopsis thaliana]
Les.1320.1.A1_at	BG630795	BG63079	45	11	11	0.25	0.24	Transcribed locus
Les.5941.1.A1_at	CK715706	TC22893	2580	642	1747	0.25	0.68	Transcribed locus, weakly similar to NP_001042315.1 [Oryza sativa (japonica cultivar-group)]
LesAffx.64757.1.A1_at	AI490042	TC22570	547	135	249	0.25	0.45	Transcribed locus
LesAffx.64757.1.S1_at	AI490042	TC22570	999	245	432	0.25	0.43	Transcribed locus
LesAffx.13252.2.A1_at	BG124820	TC22202	356	77	65	0.22	0.18	Transcribed locus, weakly similar to NP_001042395.1 [Oryza sativa (japonica cultivar-group)]
Les.3069.1.S1_at	BG630752	TC21938	1004	214	555	0.21	0.55	Lycopersicon esculentum clone 132988F, mRNA sequence
LesAffx.23349.1.S1_at	AI782014	TC21920	2010	423	2066	0.21	1.03	ADP/ATP translocator
Les.360.1.S1_at	M86724.1	TC23986	2155	449	1480	0.21	0.69	alcohol dehydrogenase
Les.1714.1.A1_at	BG628281	TC24400	677	140	505	0.21	0.75	Transcribed locus
Les.3733.1.S1_at	AF096776.1	TC21836	2244	457	146	0.2	0.06	expansin
Les.9.1.S1_at	AB010992.1	TC22813	4788	968	704	0.2	0.15	3b-hydroxylase
LesAffx.56.4.S1_at	BG128707	TC22262	219	44	53	0.2	0.24	Transcribed locus, weakly similar to XP_001087943.1 similar to carboxypeptidase, vitellogenic-like isoform 4 [Macaca mulatta]
Les.1614.1.A1_at	BG628742	TC23793	432	86	192	0.2	0.45	Transcribed locus, moderately similar to NP_001049792.1 [Oryza sativa (japonica cultivar-group)]
LesAffx.13252.1.S1_at	BI929109	TC22202	2304	431	403	0.19	0.18	Transcribed locus, weakly similar to NP_001042395.1 [Oryza sativa (japonica cultivar-group)]
Les.4397.2.A1_at	AW218741	TC23761	1155	213	357	0.18	0.31	CDNA, clone: FC25AG12, HTC in fruit
Les.4397.1.S1_at	BG127578	TC23761	478	85	147	0.18	0.31	CDNA, clone: FC25AG12, HTC in fruit
Les.5664.1.S1_at	BT014348.1	AW03195	848	148	437	0.17	0.51	Lycopersicon esculentum clone 133623F, mRNA sequence
Les.263.1.S1_at	M98466.1	TC21722	2396	412	629	0.17	0.26	polygalacturonase isoenzyme 1 beta subunit

LesAffx.13252.2.S1_at	BG124820	TC22202	1590	237	242	0.15	0.15	Transcribed locus, weakly similar to NP_001042395.1 [Oryza sativa (japonica cultivar-group)]
LesAffx.53591.1.S1_at	AI899018	TC22292	390	55	33	0.14	0.08	Transcribed locus, weakly similar to XP_001101267.1 similar to myeloblastosis proto-oncogene product isoform 2 [Macaca mulatta]
LesAffx.3099.1.S1_at	CK574995	GO37548	6180	844	861	0.14	0.14	Transcribed locus, moderately similar to NP_001044625.1 [Oryza sativa (japonica cultivar-group)]
Les.857.1.S1_at	BT013838.1	TC22565	1835	243	366	0.13	0.2	Lycopersicon esculentum clone 132795F, mRNA sequence
Les.4710.1.S1_at	BT014526.1	TC24079	370	34	33	0.09	0.09	Lycopersicon esculentum clone 133940F, mRNA sequence
LesAffx.1251.1.S1_at	BG125851	BG12585	516	46	82	0.09	0.16	---
