# Supplementary material for: Transcriptome Analysis of Tomato Flower Pedicel Tissues Reveals Abscission Zone-Specific Modulation of Key Meristem Activity Genes
Source: PLoS One. 2013 Feb 4;8(2):e55238. doi: 10.1371/journal.pone.0055238 (PMC3563536; doi:10.1371/journal.pone.0055238)
Supplement: Table S6 — MapMan classification of differentially expressed non-redundant genes in abscising abscission zone. (PDF) [file pone.0055238.s008.pdf]

Table S6. *MapMan classification of differentially expressed non-redundant genes in the abscising AZ.*

Affy_probe	GB Acession	At homolog	Tomato Gene Index	Cluster	3h: 0h	6h:0h	MapMan code	Mapman category	Arabidopsis annotation
LesAffx.64741.1.S1_at	AI775317	AT3G60510.2	TC22074	I	1.46	1.19	13.2.6.3	amino acid metabolism.degradation.aromatic aa.tryptophan	(at3g60510): enoyl-CoA hydratase/isomerase family protein
Les.5433.1.S1_at	BT013909.1	AT4G14430.1	TC22191	III	1.27	1.24	13.2.6.3	amino acid metabolism.degradation.aromatic aa.tryptophan	(at4g14430): enoyl-CoA hydratase/isomerase family protein
LesAffx.11659.1.A1_at	BI422229	AT1G12050.1	TC24331	V	-0.65	-1.64	13.2.6.2	amino acid metabolism.degradation.aromatic aa.tyrosine	(at1g12050): fumarylacetoacetase, putative
LesAffx.67643.1.S1_at	BI926164	AT3G16150.1	TC22219	I	1.7	0.06	13.2.3.1.1	amino acid metabolism.degradation.aspartate family.asparagine.L-asparaginase	(at3g16150): L-asparaginase, putative / L-asparagine amidohydrolase, putative
LesAffx.3379.1.A1_at	AW039492	AT4G34030.1	TC24415	I	0.99	0.62	13.2.4.4	amino acid metabolism.degradation.branched-chain group.leucine	(at4g34030): MCCB (3-METHYLCROTONYL-COA CARBOXYLASE); biotin carboxylase
Les.4803.1.S1_at	BT013418.1	AT5G38710.1	TC21851	I	1.09	0.6	13.2.2.2	amino acid metabolism.degradation.glutamate family.proline	(at5g38710): proline oxidase, putative / osmotic stress-responsive proline dehydrogenase, putative
LesAffx.51554.1.A1_at	AW093474	AT1G08630.2	GO37601	I	1.07	-0.65	13.2.5.2	amino acid metabolism.degradation.serine-glycine-cysteine group.glycine	(at1g08630): THA1 (THREONINE ALDOLASE 1); aldehyde-lyase/ threonine aldolase
LesAffx.62131.1.S1_at	BE437087	AT1G08630.4	TC23252	I	1.08	-0.46	13.2.5.2	amino acid metabolism.degradation.serine-glycine-cysteine group.glycine	(at1g08630): THA1 (THREONINE ALDOLASE 1); aldehyde-lyase/ threonine aldolase
LesAffx.41477.1.S1_at	AI782352	AT1G68010.1	TC22420	V	-0.71	-1.76	13.2.5.2	amino acid metabolism.degradation.serine-glycine-cysteine group.glycine	(at1g68010): HPR (Hydroxypyruvate reductase); NAD binding / cofactor binding / oxidoreductase, acting on the CH-OH group of donors, NAD or NADP as acceptor

LesAffx.10955.1.S1_at	BI422058	AT5G22630.1	TC22366	I	1.14	0.13	13.1.6.3.1	amino acid metabolism.synthesis.aromatic aa.phenylalanine.arogenate dehydratase / prephenate dehydratase	(at5g22630): prephenate dehydratase family protein
LesAffx.66354.1.S1_at	AI773917	AT5G34930.1	TC22427	I	2.72	1.36	13.1.6.4.1	amino acid metabolism.synthesis.aromatic aa.tyrosine.arogenate dehydrogenase \& prephenate dehydrogenase	(at5g34930): arogenate dehydrogenase
Les.4317.1.S1_at	AW625684	AT3G47340.1	TC23953	III	0.86	1.61	13.1.3.1.1	amino acid metabolism.synthesis.aspartate family.asparagine.asparagine synthetase	(at3g47340): ASN1 (DARK INDUCIBLE 6)
LesAffx.9260.1.S1_at	AI781857	AT5G52100.1	DB68638	VI	-0.93	-1.1	13.1.3.5.2	amino acid metabolism.synthesis.aspartate family.lysine.dihydrodipicolinate reductase	(at5g52100): dihydrodipicolinate reductase family protein
Les.1054.1.S1_at	BM412386	AT3G22740.1	TC21841	I	1.09	0.61	13.1.3.4.12	amino acid metabolism.synthesis.aspartate family.methionine.homocysteine S-methyltransferase	(at3g22740): HMT3 (Homocysteine S-methyltransferase 3); homocysteine S-methyltransferase
Les.4488.1.S1_at	BG628643	AT3G10050.1	TC22827	V	-0.19	-2.05	13.1.4.5.1	amino acid metabolism.synthesis.branched chain group.isoleucine specific.threonine ammonia-lyase	(at3g10050): OMR1 (L-O-METHYLTHREONINE RESISTANT 1); threonine ammonia-lyase
LesAffx.7894.1.S1_at	AW933852	AT5G19550.1	TC23795	II	0.13	1.36	13.1.1.2.1	amino acid metabolism.synthesis.central amino acid metabolism.aspartate.aspartate aminotransferase	(at5g19550): ASP2 (ASPARTATE AMINOTRANSFERASE 2)
Les.3289.1.S1_at	AY240229.1	AT3G22200.1	TC21835	I	1.01	0.67	13.1.1.1.2	amino acid metabolism.synthesis.central amino acid metabolism.GABA.GABA transaminase	(at3g22200): POP2 (POLLEN-PISTIL INCOMPATIBILITY 2); 4-aminobutyrate transaminase

Les.4067.1.S1_at	AY240231.1	AT3G22200.1	TC22590	I	1.11	0.49	13.1.1.1.2	amino acid metabolism.synthesis.central amino acid metabolism.GABA.GABA transaminase	(at3g22200): POP2 (POLLEN-PISTIL INCOMPATIBILITY 2); 4-aminobutyrate transaminase
Les.1258.1.S1_at	AY240230.1	AT3G22200.1	TC21731	VI	-1.51	-2.27	13.1.1.1.2	amino acid metabolism.synthesis.central amino acid metabolism.GABA.GABA transaminase	(at3g22200): POP2 (POLLEN-PISTIL INCOMPATIBILITY 2); 4-aminobutyrate transaminase
Les.3018.1.S1_at	BT013902.1	AT3G61440.1	TC22606	I	1.14	0.91	13.1.5.3.1	amino acid metabolism.synthesis.serine-glycine-cysteine group.cysteine.OASTL	(at3g61440): ATCYSC1 (BETA-SUBSTITUTED ALA SYNTHASE 3;1); L-3-cyanoalanine synthase/ cysteine synthase
Les.3290.1.S1_at	BG123573	AT1G70580.4	TC22022	VI	-1.08	-1.41	13.1.5.2.2	amino acid metabolism.synthesis.serine-glycine-cysteine group.glycine.glycine transaminase	(at1g70580): AOAT2 (ALANINE-2-OXOGLUTARATE AMINOTRANSFERASE 2); alanine transaminase
Les.2703.1.S1_at	BG735318	AT1G17745.1	TC23293	VI	-0.91	-1.11	13.1.5.1.1	amino acid metabolism.synthesis.serine-glycine-cysteine group.serine.phosphoglycerate dehydrogenase	(at1g17745): PGDH (3-PHOSPHOGLYCERATE DEHYDROGENASE); phosphoglycerate dehydrogenase
Les.1665.1.S1_at	AW623646	AT1G80160.1	DB71244	I	1.81	1.06	24.2	Biodegradation of Xenobiotics.lactoylglutathione lyase	(at1g80160): lactoylglutathione lyase family protein / glyoxalase I family protein
LesAffx.9007.1.S1_at	CN385923	AT1G80160.1	GO37540	IV	-1.22	-0.69	24.2	Biodegradation of Xenobiotics.lactoylglutathione lyase	(at1g80160): lactoylglutathione lyase family protein / glyoxalase I family protein
Les.3610.1.S1_at	BG629712	AT4G13340.1	TC23477	II	1.05	3.31	10.5.3	cell wall.cell wall proteins.LRR	(at4g13340): leucine-rich repeat family protein / extensin family protein
Les.2946.2.S1_at	BG130169	AT3G22800.1	TC22071	V	-0.24	-1.27	10.5.3	cell wall.cell wall proteins.LRR	(at3g22800): leucine-rich repeat family protein / extensin family protein
Les.3330.1.S1_at	AW650121	AT4G12730.1	TC24355	VI	-1.2	-1.12	10.5.1	cell wall.cell wall proteins.AGPs	(at4g12730): FLA2 (FLA2)
LesAffx.70270.1.S1_at	AI773931	AT5G22740.1	TC23665	IV	-1.81	-1.19	10.2	cell wall.cellulose synthesis	(at5g22740): ATCSLA02 (Cellulose synthase-like A2); transferase, transferring glycosyl groups

Les.2316.1.S1_at	AW623527	AT4G24000.1	TC22940	V	-1.83	-4.42	10.2.1	cell wall.cellulose synthesis.cellulose synthase	(at4g24000): ATCSLG2 (Cellulose synthase-like G2); transferase/ transferase, transferring glycosyl groups
Les.4520.1.S1_at	BT013422.1	AT3G02210.1	GO37596	VI	-1.14	-0.98	10.2.2	cell wall.cellulose synthesis.COBRA	(at3g02210): phytochelatin synthetase family protein / COBRA cell expansion protein COBL3
Les.4549.1.S1_at	BT013085.1	AT2G32990.1	TC24365	VI	-1.21	-1.31	10.6.1	cell wall.degradation.cellulases and beta -1,4-glucanases	(at2g32990): glycosyl hydrolase family 9 protein
Les.3663.1.S1_at	X55193.1	AT3G27400.1	TC22989	II	-0.82	1.41	10.6.3	cell wall.degradation.pectate lyases and polygalacturonases	(at3g27400): pectate lyase family protein
Les.4707.1.S1_at	BT012714.1	AT4G24780.1	TC23557	II	-1	1.57	10.6.3	cell wall.degradation.pectate lyases and polygalacturonases	(at4g24780): pectate lyase family protein
Les.3646.1.S1_at	U70481.1	AT3G59850.1	TC21735	III	3.06	3.29	10.6.3	cell wall.degradation.pectate lyases and polygalacturonases	(at3g59850): polygalacturonase, putative / pectinase, putative
Les.3647.1.S1_at	U70480.1	AT3G59850.1	TC21734	III	4.91	5.38	10.6.3	cell wall.degradation.pectate lyases and polygalacturonases	(at3g59850): polygalacturonase, putative / pectinase, putative
Les.3665.1.S1_at	U23053.1	AT3G59850.1	TC21734	III	5.41	7.31	10.6.3	cell wall.degradation.pectate lyases and polygalacturonases	(at3g59850): polygalacturonase, putative / pectinase, putative
LesAffx.13252.1.S1_at	BI929109	AT1G67830.1	TC22202	IV	-1.78	-0.97	10.6.2	cell wall.degradation.mannan-xylose-arabinose-fucose	(at1g67830): ATFXG1 (ALPHA-FUCOSIDASE 1); alpha-L-fucosidase/ carboxylic ester hydrolase
Les.263.1.S1_at	M98466.1	AT1G70370.1	TC21722	IV	-1.61	0.15	10.6.3	cell wall.degradation.pectate lyases and polygalacturonases	(at1g70370): BURP domain-containing protein / polygalacturonase, putative
LesAffx.1276.1.S1_at	BG130074	AT5G48900.1	TC23044	V	-0.55	-1.24	10.6.3	cell wall.degradation.pectate lyases and polygalacturonases	(at5g48900): pectate lyase family protein
Les.857.1.S1_at	BT013838.1	AT3G61490.2	TC22565	VI	-2.2	-2.13	10.6.3	cell wall.degradation.pectate lyases and polygalacturonases	(at3g61490): glycoside hydrolase family 28 protein / polygalacturonase (pectinase) family protein
LesAffx.56547.2.S1_at	BI207815	AT5G06870.1	TC22569	VI	-2.08	-2.52	10.6.3	cell wall.degradation.pectate lyases and polygalacturonases	(at5g06870): PGIP2 (POLYGALACTURONASE INHIBITING PROTEIN 2); protein binding
Les.191.1.S1_at	AF548376.1	AT2G39700.1	TC21771	II	-0.56	2.18	10.7	cell wall.modification	(at2g39700): ATEXPA4 (ARABIDOPSIS THALIANA EXPANSIN A4)
Les.2688.1.S1_at	AJ560646.1	AT5G39270.1	TC21917	II	1.08	3.02	10.7	cell wall.modification	MULTIPLE HITS: (at5g39270,at5g39290). at5g39270: ATEXPA22 (ARABIDOPSIS THALIANA EXPANSIN A22) at5g39290: ATEXP26 (ARABIDOPSIS THALIANA EXPANSIN A26)

Les.369.1.S1_at	AF059489.1	AT1G26770.2	TC21922	IV	-1.6	-0.41	10.7	cell wall.modification	(at1g26770): ATEXPA10 (ARABIDOPSIS THALIANA EXPANSIN A10)
Les.3972.1.S1_at	AJ243340.1	AT2G28950.1	TC22219	IV	-1.87	0.08	10.7	cell wall.modification	(at2g28950): ATEXPA6 (ARABIDOPSIS THALIANA EXPANSIN A6)
Les.3733.1.S1_at	AF096776.1	AT2G40610.1	TC21836	IV	-1.28	-0.4	10.7	cell wall.modification	(at2g40610): ATEXPA8 (ARABIDOPSIS THALIANA EXPANSIN A8)
Les.3569.1.S1_at	AF059487.1	AT3G03220.1	TC21833	IV	-1.35	-0.56	10.7	cell wall.modification	(at3g03220): ATEXPA13 (ARABIDOPSIS THALIANA EXPANSIN A13)
Les.4353.1.S1_at	AY497476.1	AT5G57560.1	TC21758	I	2.55	1.93	10.7	cell wall.modification	XTH (at5g57560): TCH4 (TOUCH 4); hydrolase, acting on glycosyl bonds
Les.429.1.S1_at	X82684.1	AT3G23730.1	TC22396	I	2.65	1.72	10.7	cell wall.modification	XTH (at3g23730): xyloglucan:xyloglucosyl transferase, putative / xyloglucan endotransglycosylase, putative / endo-xyloglucan transferase, putative
Les.4530.1.S1_at	AY497479.1	AT3G23730.1	TC21992	III	2.36	3.61	10.7	cell wall.modification	XTH (at3g23730): xyloglucan:xyloglucosyl transferase, putative / xyloglucan endotransglycosylase, putative / endo-xyloglucan transferase, putative
Les.4523.1.S1_at	AY497475.1	AT4G18990.1	TC21776	III	1.25	1.21	10.7	cell wall.modification	XTH (at4g18990): xyloglucan:xyloglucosyl transferase, putative / xyloglucan endotransglycosylase, putative / endo-xyloglucan transferase, putative
LesAffx.24799.1.S1_at	CK715756	AT1G11545.1	TC23394	IV	-1.2	-0.55	10.7	cell wall.modification	XTH (at1g11545): xyloglucan:xyloglucosyl transferase, putative / xyloglucan endotransglycosylase, putative / endo-xyloglucan transferase, putative
LesAffx.69296.1.A1_at	AW092917	AT2G36870.1	TC22654	V	-0.52	-1.65	10.7	cell wall.modification	XTH (at2g36870): xyloglucan:xyloglucosyl transferase, putative / xyloglucan endotransglycosylase, putative / endo-xyloglucan transferase, putative
Les.4522.1.S1_at	AY497477.1	AT3G44990.1	TC23773	V	-0.35	-2.57	10.7	cell wall.modification	XTH (at3g44990): XTR8 (xyloglucan:xyloglucosyl transferase 8); hydrolase, acting on glycosyl bonds

Les.4529.1.S1_at	AY497478.1	AT4G37800.1	TC21903	VI	-1.22	-1.92	10.7	cell wall.modification	XTH (at4g37800): xyloglucan:xyloglucosyl transferase, putative / xyloglucan endotransglycosylase, putative / endo-xyloglucan transferase, putative
Les.3122.1.S1_a_at	BT013364.1	AT3G43270.1	TC21726	IV	-1.43	-0.18	10.8.1	cell wall.pectin*esterases.PME	(at3g43270): pectinesterase family protein
Les.67.1.S1_at	U49330.1	AT3G14310.1	TC21724	IV	-1.25	-0.46	10.8.1	cell wall.pectin*esterases.PME	(at3g14310): ATPME3 (Arabidopsis thaliana pectin methylesterase 3)
LesAffx.10497.2.A1_at	AI897776	AT3G49220.1	TC24435	VI	-1.57	-1.48	10.8.1	cell wall.pectin*esterases.PME	(at3g49220): pectinesterase family protein
Les.1248.2.A1_at	BT012749.1	AT2G39770.1	TC21728	VI	-0.89	-1.23	10.1.1.1	cell wall.precursor synthesis.NDP sugar pyrophosphorylase.GDP mannose	(at2g39770): CYT1 (CYTOKINESIS DEFECTIVE 1); nucleotidyltransferase
Les.103.1.S1_at	AJ243451.1	AT1G44110.1	TC22759	V	-0.5	-1.13	31.3	cell.cycle	(at1g44110): CYCA1;1 (CYCLIN A1;1); cyclin-dependent protein kinase regulator
LesAffx.69295.1.S1_at	BI933687	AT2G45080.1	TC23040	V	-1.15	-2.24	31.3	cell.cycle	(at2g45080): CYCP3;1 (cyclin p3;1); cyclin-dependent protein kinase
Les.3055.1.S1_at	AJ002590.1	AT3G50070.1	TC21769	IV	-1.49	-0.34	31.3	cell.cycle	(at3g50070): CYCD3;3 (CYCLIN D3;3); cyclin-dependent protein kinase
Les.3519.1.S1_at	AJ002589.1	AT3G50070.1	TC23298	IV	-2.06	-0.72	31.3	cell.cycle	(at3g50070): CYCD3;3 (CYCLIN D3;3); cyclin-dependent protein kinase
Les.4444.1.S1_s_at	BI207010	AT5G67260.1	TC22444	IV	-1.54	-0.34	31.3	cell.cycle	(at5g67260): CYCD3;2 (CYCLIN D3;2); cyclin-dependent protein kinase
LesAffx.38145.1.A1_at	AI487713	AT5G10490.2	BM40940	V	-0.31	-1.08	31.2.5	cell.division.plastid	(at5g10490): MSL2 (MSCS-LIKE 2)
LesAffx.1001.1.S1_at	BM535807	AT2G18280.2	TC22526	I	1.3	0.85	31.1	cell.organisation	(at2g18280): AtTLP2 (TUBBY LIKE PROTEIN 2); phosphoric diester hydrolase/ transcription factor
Les.5161.1.S1_at	BT013412.1	AT2G37080.1	AI77756	VI	-1.81	-1.34	31.1	cell.organisation	(at2g37080): myosin heavy chain-related
Les.1514.1.S1_at	BG626292	AT2G35490.1	TC23484	V	-0.51	-1.09	31.1	cell.organisation	(at2g35490): plastid-lipid associated protein PAP, putative
LesAffx.64540.1.S1_at	AW441832	AT5G19940.1	TC22052	V	0.02	-1.06	31.1	cell.organisation	(at5g19940): plastid-lipid associated protein PAP-related / fibrillin-related
LesAffx.37916.1.S1_at	BM536108	AT1G09155.1	TC23806	III	1.03	1.58	31.1	cell.organisation	(at1g09155): ATPP2-B15 (Phloem protein 2-B15)
Les.4564.1.S1_at	BT013856.1	AT5G55230.1	TC21760	IV	-1.31	-0.58	31.1	cell.organisation	(at5g55230): ATMAP65-1 (MICROTUBULE-ASSOCIATED PROTEINS 65-1); microtubule binding

LesAffx.71000.1.S1_at	BF096685	AT5G50375.1	TC23035	IV	-1.05	-0.61	31.4	cell.vesicle transport	(at5g50375): CPI1 (CYCLOPROPYL ISOMERASE)
Les.1044.1.A1_at	BG629030	AT2G43360.1	TC23609	II	-0.11	1.5	18.6.1	Co-factor and vitamine metabolism.biotin.biotin synthase	(at2g43360): BIO2 (BIOTIN AUXOTROPH 2); biotin synthase
LesAffx.66163.1.S1_at	AW222774	AT3G16190.1	TC22788	III	1.08	1.18	18.13	Co-factor and vitamine metabolism.isochorismatase	(at3g16190): isochorismatase hydrolase family protein
LesAffx.3099.1.S1_at	CK574995	AT5G54770.1	GO37548	VI	-3.15	-3.07	18.2	Co-factor and vitamine metabolism.thiamine	(at5g54770): THI1 (THIAZOLE REQUIRING)
Les.4594.1.S1_at	BT012739.1	AT2G29630.1	TC23704	IV	-2.84	-1.41	18.2	Co-factor and vitamine metabolism.thiamine	(at2g29630): thiamine biosynthesis family protein / thiC family protein
Les.2212.1.A1_at	BG735110	AT5G13790.1	BG73511	I	1.33	0.61	33.99	development.unspecified	TF (at5g13790): AGL15 (AGAMOUS-LIKE 15); DNA binding / transcription factor
Les.4483.1.S1_at	AY498713.1	AT1G01720.1	TC21812	I	1.17	0.18	33.99	development.unspecified	TF (at1g01720): ATAF1 (Arabidopsis NAC domain containing protein 2); transcription factor
LesAffx.43329.1.A1_at	AI489164	AT5G07680.1	TC23564	I	1.54	0.9	33.99	development.unspecified	TF (at5g07680): ANAC079/ANAC080/ATNAC4 (Arabidopsis NAC domain containing protein 79, Arabidopsis NAC domain containing protein 80); transcription factor
LesAffx.57572.1.S1_at	CN385925	AT4G37060.1	TC21838	II	0.19	0.98	33.1	development.storage proteins	MULTIPLE HITS: (at4g37070,at4g37060). at4g37070: PLA IVA/PLP1; nutrient reservoir at4g37060: PLA IVB/PLP5 (Patatin-like protein 5); nutrient reservoir
Les.3759.1.S1_at	Z46654.1	AT2G46140.1	TC21776	III	1.02	1.18	33.2	development.late embryogenesis abundant	(at2g46140): late embryogenesis abundant protein, putative / LEA protein, putative
Les.3766.1.S1_at	U77719.1	AT1G01470.1	TC21779	III	2.33	2.22	33.2	development.late embryogenesis abundant	(at1g01470): LEA14 (LATE EMBRYOGENESIS ABUNDANT 14)
Les.2084.1.S1_at	BF097539	AT1G69490.1	TC22764	III	1.74	1.5	33.99	development.unspecified	TF (at1g69490): NAP (NAC-LIKE, ACTIVATED BY AP3/PI); transcription factor
Les.2494.1.S1_at	AJ785249	AT3G48140.1	BI20405	III	0.63	1	33.99	development.unspecified	(at3g48140): senescence-associated protein, putative
Les.2717.1.S1_at	AW218373	AT3G48140.1	TC22994	III	1.86	1.76	33.99	development.unspecified	(at3g48140): senescence-associated protein, putative
Les.5044.1.S1_at	BT013168.1	AT2G35980.1	TC22109	III	1.13	1.47	33.99	development.unspecified	(at2g35980): YLS9 (YELLOW-LEAF-SPECIFIC GENE 9)
LesAffx.31514.1.S1_at	BG127453	AT1G75500.1	TC22656	III	0.58	1.01	33.99	development.unspecified	(at1g75500): nodulin MtN21 family protein

Les.1348.1.A1_at	BG631005	AT4G17670.1	TC22489	IV	-1.88	-1.23	33.99	development.unspecified	(at4g17670): senescence-associated protein-related
Les.4731.1.S1_at	BT013577.1	AT5G60690.1	TC23297	IV	-1.63	-0.44	33.99	development.unspecified	TF (at5g60690): REV (REVOLUTA); DNA binding / lipid binding / transcription factor
Les.4962.1.S1_at	BI204454	AT2G19580.1	TC22450	IV	-1.36	-0.15	33.99	development.unspecified	(at2g19580): TET2 (TETRASPANIN2)
Les.4987.1.S1_at	BT013055.1	AT3G45600.1	TC23511	IV	-1.61	-0.78	33.99	development.unspecified	(at3g45600): TET3 (TETRASPANIN3)
LesAffx.56237.1.S1_at	AW218110	AT2G37460.1	TC22223	IV	-1.34	0.01	33.99	development.unspecified	(at2g37460): nodulin MtN21 family protein
LesAffx.66974.1.S1_at	BG135595	AT5G46700.1	TC23429	IV	-2	0.25	33.99	development.unspecified	(at5g46700): TRN2 (TORNADO 2)
LesAffx.26970.1.S1_at	BF050591	AT4G17230.1	BF05059	V	-0.75	-1.27	33.99	development.unspecified	TF (at4g17230): SCL13 (SCARECROW-LIKE 13); transcription factor
LesAffx.65682.1.A1_at	AI489857	AT5G50790.1	TC22844	V	-0.47	-1.67	33.99	development.unspecified	(at5g50790): nodulin MtN3 family protein
LesAffx.68607.1.S1_at	BM409831	AT3G54950.1	TC23658	VI	-1.29	-1	33.1	development.storage proteins	(at3g54950): PLA IIIA/PLP7 (Patatin-like protein 7)
Les.4362.1.A1_at	AY306154.1	AT1G69120.1	TC22524	VI	-0.82	-1.05	33.99	development.unspecified	TF (at1g69120): AP1 (APETALA1); DNA binding / transcription factor
LesAffx.66165.1.S1_at	AW649540	AT5G53090.1	TC22272	VI	-1.09	-0.89	33.99	development.unspecified	(at5g53090): oxidoreductase
LesAffx.885.1.S1_at	BI205930	AT1G63260.3	BI20593	VI	-1.84	-1.44	33.99	development.unspecified	(at1g63260): TET10 (TETRASPANIN10)
Les.5740.1.S1_at	BT014477.1	AT2G24490.2	TC22279	II	-0.15	0.98	28.2	DNA.repair	(at2g24490): ATRPA2/ROR1/RPA2 (REPLICON PROTEIN A); protein binding
LesAffx.56137.1.A1_at	AI490099	AT1G11190.1	TC22599	II	1.11	2.75	28.1	DNA.synthesis/chromatin structure	(at1g11190): BFN1 (BIFUNCTIONAL NUCLEASE I); nucleic acid binding
Les.4948.1.S1_at	BT012962.1	AT1G68290.1	TC23041	III	0.88	1.53	28.1	DNA.synthesis/chromatin structure	(at1g68290): bifunctional nuclease, putative
Les.4412.1.A1_at	BG627732	AT3G13610.1	TC23503	VI	-1.05	-1.35	28.99	DNA.unspecified	(at3g13610): oxidoreductase, 2OG-Fe(II) oxygenase family protein
Les.360.1.S1_at	M86724.1	AT1G77120.1	TC23986	IV	-1.83	0.28	5.3	fermentation.ADH	(at1g77120): ADH1 (ALCOHOL DEHYDROGENASE 1); alcohol dehydrogenase
LesAffx.13053.1.S1_at	AW032379	AT3G24503.1	TC22409	I	1.15	0.92	5.1	fermentation.aldehyde dehydrogenase	(at3g24503): ALDH2C4 (REDUCED EPIDERMAL FLUORESCENCE1); 3-chloroallyl aldehyde dehydrogenase/ aldehyde dehydrogenase (NAD)
Les.2831.1.S1_at	BG627219	AT3G48000.1	TC22500	IV	-1.48	-0.95	5.1	fermentation.aldehyde dehydrogenase	(at3g48000): ALDH2B4 (ALDEHYDE DEHYDROGENASE 2); 3-chloroallyl aldehyde dehydrogenase/ aldehyde dehydrogenase (NAD)

Les.4617.1.S1_at	BT014212.1	AT2G24270.1	TC23312	V	-0.86	-2.24	4.1.9	glycolysis.cytosolic branch.non-phosphorylating glyceraldehyde 3-phosphate dehydrogenase (NPGAP-DH)	(at2g24270): ALDH11A3 (Aldehyde dehydrogenase 11A3); 3-chloroallyl aldehyde dehydrogenase
Les.19.1.S1_at	BG626377	AT1G53310.3	TC24377	VI	-1.1	-1.42	4.1.15	glycolysis.cytosolic branch.phospho-enol-pyruvate carboxylase (PEPC)	(at1g53310): ATPPC1 (PHOSPHOENOLPYRUVATE CARBOXYLASE 1); phosphoenolpyruvate carboxylase
Les.2909.1.S1_at	AW223174	AT1G53310.3	TC24474	V	-0.96	-1.92	4.1.15	glycolysis.cytosolic branch.phospho-enol-pyruvate carboxylase (PEPC)	(at1g53310): ATPPC1 (PHOSPHOENOLPYRUVATE CARBOXYLASE 1); phosphoenolpyruvate carboxylase
Les.4099.1.S1_at	AY187634.1	AT1G08650.1	TC21751	V	-0.37	-1.08	4.1.16	glycolysis.cytosolic branch.phospho-enol-pyruvate carboxylase kinase (PPCK)	(at1g08650): PPCK1 (PHOSPHOENOLPYRUVATE CARBOXYLASE KINASE); kinase
Les.3539.1.S1_at	AF203481.1	AT3G04530.1	TC21733	III	0.67	1.04	4.3.16	glycolysis.unclear/dually targeted.phospho-enol-pyruvate carboxylase kinase (PEPCK)	(at3g04530): PPCK2 (PHOSPHOENOLPYRUVATE CARBOXYLASE KINASE 2); kinase
LesAffx.22699.1.A1_at	AI489739	AT4G19230.2	TC23987	III	3.29	3.56	17.1.1.2.1	hormone metabolism.abscisic acid.synthesis-degradation.degradation.8-hydroxylase	(at4g19230): CYP707A1 (cytochrome P450, family 707, subfamily A, polypeptide 1); oxygen binding
Les.112.1.S1_at	BG734834	AT5G67030.1	TC21719	VI	-0.87	-1.42	17.1.1.1.1	hormone metabolism.abscisic acid.synthesis-degradation.synthesis.zeaxanthin epoxidase	(at5g67030): ABA1 (ABA DEFICIENT 1); zeaxanthin epoxidase
Les.5177.1.S1_at	BT013446.1	AT2G23170.1	TC24190	II	1.15	3.86	17.2.3	hormone metabolism.auxin.induced-	(at2g23170): GH3.3; indole-3-acetic acid amido synthetase
Les.5312.1.S1_at	BT013697.1	AT2G46370.2	TC22735	V	-1.44	-2.8	17.2.3	hormone metabolism.auxin.induced-	(at2g46370): JAR1 (JASMONATE RESISTANT 1)
Les.5114.1.S1_at	BT013305.1	AT3G47620.1	TC24276	VI	-1.11	-1.15	17.2.3	hormone metabolism.auxin.induced-	(at3g47620): TCP family transcription factor, putative
Les.5745.1.S1_at	BT014484.1	AT1G05560.1	TC22393	V	-0.79	-1.56	17.2.1	hormone metabolism.auxin.synthesis-degradation	(at1g05560): UGT1 (UDP-glucosyl transferase 75B1); UDP-glycosyltransferase/ transferase, transferring glycosyl groups

Les.3216.1.S1_at	AW930422	AT1G51760.1	TC22776	VI	-1.42	-1.3	17.2.1	hormone metabolism.auxin.synthesis-degradation	MULTIPLE HITS: (at1g51760,at1g51780). at1g51760: IAR3 (IAA-ALANINE RESISTANT 3); metallopeptidase at1g51780: ILL5 (IAA-leucine resistant (ILR)-like gene 5); metallopeptidase
LesAffx.59842.1.S1_at	BI205718	AT3G50660.1	TC21823	II	0.07	2.76	17.3.1.1.2	hormone metabolism.brassinosteroid.synthesis-degradation.BRs.DWF4	(at3g50660): DWF4 (DWARF 4)
Les.1212.1.S1_at	AY584532.1	AT3G19820.2	GO37246	VI	-1.41	-0.98	17.3.1.2.8	hormone metabolism.brassinosteroid.synthesis-degradation.sterols.DWF1	(at3g19820): DWF1 (DIMINUTO 1); catalytic
Les.2474.1.S1_at	BG791226	AT3G19820.2	TC23985	VI	-2.04	-1.99	17.3.1.2.8	hormone metabolism.brassinosteroid.synthesis-degradation.sterols.DWF1	(at3g19820): DWF1 (DIMINUTO 1); catalytic
LesAffx.7.1.S1_at	AW931153	AT3G52940.1	TC22173	VI	-0.85	-1.14	17.3.1.2.4	hormone metabolism.brassinosteroid.synthesis-degradation.sterols.FACKEL	(at3g52940): FK (FACKEL); delta14-sterol reductase
Les.3818.1.S1_at	AF502085.1	AT3G23240.1	TC21752	I	1.93	1.22	17.5.2	hormone metabolism.ethylene.signal transduction	(at3g23240): ATERF1/ERF1 (ETHYLENE RESPONSE FACTOR 1); DNA binding / transcription factor/ transcriptional activator
Les.4140.1.S1_at	AY192367.1	AT5G47220.1	TC22892	I	1.05	0.31	17.5.2	hormone metabolism.ethylene.signal transduction	(at5g47220): ATERF-2/ATERF2/ERF2 (ETHYLENE RESPONSE FACTOR 2); DNA binding / transcription factor/ transcriptional activator
LesAffx.3059.1.S1_at	AI776626	AT3G23220.1	AI77662	I	1.54	0.39	17.5.2	hormone metabolism.ethylene.signal transduction	(at3g23220): DNA binding / transcription factor
Les.3662.1.S1_at	M34289.1	AT4G11280.1	TC21724	I	1	0.59	17.5.1.1	hormone metabolism.ethylene.synthesis-degradation.1-aminocyclopropane-1-carboxylate synthase	(at4g11280): ACS6 (1-AMINOCYCLOPROPANE-1-CARBOXYLIC ACID (ACC) SYNTHASE 6)
Les.3465.1.S1_at	AY079426.1	AT3G04580.2	TC21718	III	1.87	2.29	17.5.2	hormone metabolism.ethylene.signal transduction	LeETR6 (at3g04580): EIN4 (ETHYLENE INSENSITIVE 4); receptor

Les.3575.1.S1_at	U89256.1	AT4G17500.1	TC22509	III	2.07	2.23	17.5.2	hormone metabolism.ethylene.signal transduction	(at4g17500): ATERF-1 (ETHYLENE RESPONSIVE ELEMENT BINDING FACTOR 1); DNA binding / transcription factor/ transcriptional activator
LesAffx.33607.1.S1_s_at	AW223067	AT5G43450.1	TC23890	III	1.64	1.48	17.5.1	hormone metabolism.ethylene.synthesis-degradation	(at5g43450): 2-oxoglutarate-dependent dioxygenase, putative
Les.132.1.S1_at	X58885.1	AT1G05010.1	TC21743	III	2.95	2.96	17.5.1.2	hormone metabolism.ethylene.synthesis-degradation.1-aminocyclopropane-1-carboxylate oxidase	(at1g05010): EFE (ethylene forming enzyme)
Les.5917.1.S1_at	AJ715790.1	AT2G19590.1	TC21740	III	1.04	1.27	17.5.1.2	hormone metabolism.ethylene.synthesis-degradation.1-aminocyclopropane-1-carboxylate oxidase	(at2g19590): ACO1 (ACC OXIDASE 1); 1-aminocyclopropane-1-carboxylate oxidase
LesAffx.70590.1.S1_at	AI775408	AT4G21340.1	TC22762	V	-0.26	-1.1	17.5.3	hormone metabolism.ethylene.induced-regulated-responsive-activated	(at4g21340): B70; transcription factor
LesAffx.57163.2.S1_at	BI421649	AT1G62380.1	TC22802	V	1.43	-1.23	17.5.1.2	hormone metabolism.ethylene.synthesis-degradation.1-aminocyclopropane-1-carboxylate oxidase	(at1g62380): ACO2 (ACC OXIDASE 2)
Les.3500.1.S1_at	AF043085.1	AT1G66340.1	TC21717	VI	-1.4	-1.07	17.5.2	hormone metabolism.ethylene.signal transduction	LeETR2 (at1g66340): ETR1 (ETHYLENE RESPONSE 1); two-component response regulator
Les.3625.1.S1_at	L22188.1	AT3G02885.1	TC23715	II	-0.11	2.49	17.6.3	hormone metabolism.gibberelin.induced-regulated-responsive-activated	(at3g02885): GASA5 (GAST1 PROTEIN HOMOLOG 5)
Les.827.1.S1_at	BG626882	AT1G74670.1	TC23903	II	0.19	1.05	17.6.3	hormone metabolism.gibberelin.induced-regulated-responsive-activated	(at1g74670): gibberellin-responsive protein, putative
LesAffx.14450.1.S1_at	AW649659	AT1G74670.1	AW64965	II	-0.17	1.3	17.6.3	hormone metabolism.gibberelin.induced-regulated-responsive-activated	(at1g74670): gibberellin-responsive protein, putative

Les.63.1.S1_at	AF049900.1	AT4G25420.1	TC22864	II	1.13	3.46	17.6.1.11	hormone metabolism.gibberelin.synthesis-degradation.GA20 oxidase	(at4g25420): GA5 (GA REQUIRING 5); gibberellin 20-oxidase/ gibberellin 3-beta-dioxygenase
LesAffx.16672.1.A1_at	AW625117	AT3G63010.1	TC23550	VI	-1.06	-1.01	17.6.2	hormone metabolism.gibberelin.signal transduction	(at3g63010): ATGID1B/GID1B (GA INSENSITIVE DWARF1B)
LesAffx.67395.1.A1_at	AI490749	AT5G27320.1	TC23607	VI	-1.07	-0.92	17.6.2	hormone metabolism.gibberelin.signal transduction	(at5g27320): ATGID1C/GID1C (GA INSENSITIVE DWARF1C)
LesAffx.9038.1.S1_at	BI930643	AT1G05160.1	TC22956	VI	-2.54	-2.57	17.6.1.4	hormone metabolism.gibberelin.synthesis-degradation.ent-kaurenoic acid hydroxylase/oxygenase	(at1g05160): CYP88A3 (ENT-KAURENOIC ACID HYDROXYLASE 1); oxygen binding
Les.9.1.S1_at	AB010992.1	AT1G15550.1	TC22813	VI	-2.33	-2.34	17.6.1.12	hormone metabolism.gibberelin.synthesis-degradation.GA3 oxidase	(at1g15550): GA4 (GA REQUIRING 4); gibberellin 3-beta-dioxygenase
Les.3718.1.S1_at	AF384374.1	AT3G25780.1	TC21922	V	-0.46	-1.99	17.7.1.4	hormone metabolism.jasmonate.synthesis-degradation.allene oxidase cyclase	(at3g25780): AOC3 (ALLENE OXIDE CYCLASE 3)
Les.129.1.S1_at	AF317515.1	AT5G42650.1	TC21823	III	3.72	4.3	17.7.1.3	hormone metabolism.jasmonate.synthesis-degradation.allene oxidase synthase	(at5g42650): AOS (ALLENE OXIDE SYNTHASE); hydro-lyase/ oxygen binding
Les.3527.1.S1_at	AY008278.1	AT3G22400.1	TC21750	V	-0.65	-1.63	17.7.1.2	hormone metabolism.jasmonate.synthesis-degradation.lipoxygenase	(at3g22400): lipoxygenase, putative
Les.3668.1.S1_at	U09026.1	AT1G55020.1	TC21838	V	-0.6	-1.68	17.7.1.2	hormone metabolism.jasmonate.synthesis-degradation.lipoxygenase	(at1g55020): LOX1 (Lipoxygenase 1); lipoxygenase
LesAffx.33082.1.S1_at	BG133383	AT5G55250.1	TC23204	II	0.38	1.3	17.8.1	hormone metabolism.salicylic acid.synthesis-degradation	(at5g55250): IAMT1 (IAA CARBOXYLMETHYLTRANSFERASE 1); S-adenosylmethionine-dependent methyltransferase
LesAffx.69609.1.S1_at	AW929283	AT5G04370.2	TC23404	V	-0.87	-2.99	17.8.1	hormone metabolism.salicylic acid.synthesis-degradation	(at5g04370): S-adenosyl-L-methionine:carboxyl methyltransferase family protein
LesAffx.29797.1.S1_at	AJ784483	AT1G13420.1	TC23412	V	-0.95	-2.92	11.8	lipid metabolism."exotics" (steroids, squalene etc)	(at1g13420): sulfotransferase family protein

Les.2708.1.S1_at	BG734625	AT4G27270.1	TC21907	III	1.92	1.9	11.8	lipid metabolism."exotics" (steroids, squalene etc)	(at4g27270): quinone reductase family protein
Les.3113.1.S1_at	BG131753	AT1G07420.1	TC21889	V	-0.53	-1.3	11.8.2	lipid metabolism."exotics" (steroids, squalene etc).methylsterol monooxygenase	(at1g07420): SMO2-2 (sterol 4-alpha-methyl-oxidase 2); C-4 methylsterol oxidase
Les.3710.1.S1_at	AJ312131.1	AT3G25540.1	TC21745	III	1.63	1.52	11.8.1	lipid metabolism."exotics" (steroids, squalene	(at3g25540): LAG1 (Longevity assurance gene 1)
LesAffx.30807.1.S1_at	AW624957	AT2G46210.1	AW62495	IV	-1.15	-0.18	11.8.1	lipid metabolism."exotics" (steroids, squalene	(at2g46210): delta-8 sphingolipid desaturase, putative
Les.1562.1.S1_at	BG629310	AT2G29980.1	TC23006	VI	-1.21	-1.4	11.2.3	lipid metabolism.FA desaturation.omega 3 desaturase	(at2g29980): FAD3 (FATTY ACID DESATURASE 3); omega-3 fatty acid desaturase
Les.2415.2.S1_at	BE431455	AT4G30950.1	TC23842	V	-1.13	-1.9	11.2.4	lipid metabolism.FA desaturation.omega 6 desaturase	(at4g30950): FAD6 (FATTY ACID DESATURASE 6); omega-6 fatty acid desaturase
Les.5934.1.S1_at	AI895164	AT3G12120.2	TC21861	III	1.64	3	11.2.4	lipid metabolism.FA desaturation.omega 6 desaturase	(at3g12120): FAD2 (FATTY ACID DESATURASE 2); delta12-fatty acid dehydrogenase
Les.3383.1.S1_at	BI208081	AT3G48990.1	TC22532	I	0.95	0.24	11.1.8	lipid metabolism.FA synthesis and FA elongation.acyl coa ligase	(at3g48990): AMP-dependent synthetase and ligase family protein
Les.5150.1.S1_at	BT013389.1	AT1G77240.1	AW92832	VI	-1.47	-1.63	11.1.8	lipid metabolism.FA synthesis and FA elongation.acyl coa ligase	(at1g77240): AMP-binding protein, putative
Les.5293.1.S1_at	BT013651.1	AT2G26640.1	AW09681	I	1.03	0.53	11.1.10	lipid metabolism.FA synthesis and FA elongation.beta ketoacyl CoA synthase	(at2g26640): beta-ketoacyl-CoA synthase, putative
Les.4040.1.A1_at	X83420.1	AT2G26640.1	AW09681	III	0.87	1.15	11.1.10	lipid metabolism.FA synthesis and FA elongation.beta ketoacyl CoA synthase	(at2g26640): beta-ketoacyl-CoA synthase, putative
LesAffx.58502.1.S1_at	BM412364	AT2G03550.1	TC22738	I	1.34	0.75	11.9.4.5	lipid metabolism.lipid degradation.beta-oxidation.acyl-CoA thioesterase	(at2g03550): similar to unknown protein [Arabidopsis thaliana] (TAIR:AT3G48690.1); similar to unknown protein [Arabidopsis thaliana] (TAIR:AT3G48700.1); similar to Esterase/lipase/thioesterase [Medicago truncatula] (GB:ABE83378.1); contains InterPro domain Esterase/lipase/thioesterase; (InterPro:IPR000379); contains InterPro domain Alpha/beta hydrolase fold-3; (InterPro:IPR013094)

LesAffx.7472.2.S1_at	AW651303	AT2G31100.1	TC23954	VI	-1.23	-1.51	11.9.2	lipid metabolism.lipid degradation.lipases	(at2g31100): lipase, putative
LesAffx.3244.1.S1_at	BI934720	AT5G18640.1	TC22222	III	0.92	1.34	11.9.2	lipid metabolism.lipid degradation.lipases	(at5g18640): lipase class 3 family protein
LesAffx.45975.1.S1_at	BI209975	AT1G52760.1	BI20997	III	1.26	1.09	11.9.2	lipid metabolism.lipid degradation.lipases	(at1g52760): esterase/lipase/thioesterase family protein
LesAffx.6125.1.S1_at	CK468687	AT1G47480.1	TC22217	III	1.2	1.1	11.9.3.2	lipid metabolism.lipid degradation.lysophospholipases.carboxylesterase	(at1g47480): similar to unknown protein [Arabidopsis thaliana] (TAIR:AT3G48700.1); similar to CXE carboxylesterase [Malus pumila] (GB:ABB89003.1); contains InterPro domain Esterase/lipase/thioesterase; (InterPro:IPR000379); contains InterPro domain Alpha/beta hydrolase fold-3; (InterPro:IPR013094)
Les.3493.1.S1_at	AY013255.1	AT2G42010.1	TC21805	I	1.04	0.62	11.9.3.1	lipid metabolism.lipid degradation.lysophospholipases.phospholipase D	(at2g42010): PLDBETA1 (PHOSPHOLIPASE D BETA 1); phospholipase D
Les.4710.1.S1_at	BT014526.1	AT1G48600.2	TC24079	VI	-2.21	-2.49	11.3	lipid metabolism.Phospholipid synthesis	(at1g48600): phosphoethanolamine N-methyltransferase 2, putative (NMT2)
Les.4835.1.S1_at	BT012722.1	AT1G73600.1	TC24498	VI	-1.61	-1.31	11.3	lipid metabolism.Phospholipid synthesis	(at1g73600): phosphoethanolamine N-methyltransferase 3, putative (NMT3)
Les.3526.1.S1_a_at	AF198259.1	AT2G20900.4	TC22267	I	1.08	0.74	11.3.5	lipid metabolism.Phospholipid synthesis.diacylglycerol kinase	(at2g20900): diacylglycerol kinase, putative
Les.3460.1.S1_at	AF506005.1	AT3G52600.1	TC21723	III	0.76	1.47	2.2.1.3.2	major CHO metabolism.degradation.sucrose.invertases.cell wall	(at3g52600): ATCWINV2 (ARABIDOPSIS THALIANA CELL WALL INVERTASE 2); hydrolase, hydrolyzing O-glycosyl compounds
LesAffx.37983.1.S1_at	BG129477	AT1G22650.1	TC22731	I	0.94	0.67	2.2.1.3.1	major CHO metabolism.degradation.sucrose.invertases.neutral	(at1g22650): beta-fructofuranosidase, putative / invertase, putative / saccharase, putative / beta-fructosidase, putative
Les.642.1.S1_at	BG626672	AT1G22650.1	BG62667	VI	-1.7	-1.29	2.2.1.3.1	major CHO metabolism.degradation.sucrose.invertases.neutral	(at1g22650): beta-fructofuranosidase, putative / invertase, putative / saccharase, putative / beta-fructosidase, putative
Les.3669.1.S1_at	L19762.1	AT5G20830.2	TC21717	II	-0.05	1.59	2.2.1.5	major CHO metabolism.degradation.sucrose.Susy	(at5g20830): SUS1 (SUCROSE SYNTHASE 1); UDP-glycosyltransferase/ sucrose synthase

Les.204.1.S1_at	L41126.1	AT5G48300.1	TC21759	VI	-1.67	-1.18	2.1.2.1	major CHO metabolism.synthesis.starch.AGPase	(at5g48300): ADG1 (ADP GLUCOSE PYROPHOSPHORYLASE SMALL SUBUNIT 1); glucose-1-phosphate adenylyltransferase
Les.3064.1.S1_at	U81033.1	AT2G21590.2	TC21721	VI	-1.81	-1.3	2.1.2.1	major CHO metabolism.synthesis.starch.AGPase	(at2g21590): APL4 (large subunit of AGP 4); glucose-1-phosphate adenylyltransferase
LesAffx.71563.1.A1_at	AI487345	AT5G03650.1	TC22892	VI	-1.27	-1.35	2.1.2.3	major CHO metabolism.synthesis.starch.starch branching	(at5g03650): SBE2.2 (STARCH BRANCHING ENZYME 2.2)
Les.1310.1.S1_at	BT013430.1	AT1G32900.1	TC22042	VI	-1.25	-1.8	2.1.2.2	major CHO metabolism.synthesis.starch.starch synthase	(at1g32900): starch synthase, putative
Les.3522.1.S1_at	AF071786.3	AT5G20280.1	TC21714	V	-0.37	-1.04	2.1.1.1	major CHO metabolism.synthesis.sucrose.SPS	(at5g20280): ATSPS1F (sucrose phosphate synthase 1F); sucrose-phosphate synthase/ transferase, transferring glycosyl groups
LesAffx.58193.1.A1_at	CK715571	AT4G16120.1	TC23961	I	1.04	0.36	15	metal handling	(at4g16120): ATSEB1
LesAffx.68294.1.S1_at	BM408981	AT5G24580.1	TC22580	II	-0.19	1.73	15.2	metal handling.binding, chelation and storage	(at5g24580): copper-binding family protein
LesAffx.59536.1.S1_at	BI210459	AT3G56240.1	TC22441	III	0.66	1.1	15.2	metal handling.binding, chelation and storage	(at3g56240): CCH (COPPER CHAPERONE)
Les.5832.1.S1_at	BT014414.1	AT2G36790.1	TC22401	VI	-1.54	-2.26	32	micro RNA, natural antisense etc	MULTIPLE HITS: (at2g36800,at2g36792,at2g36790). at2g36800: DOGT1 (DON-GLUCOSYLTRANSFERASE); UDP-glycosyltransferase/ transferase, transferring glycosyl groups at2g36792: other RNA at2g36790: UGT73C6 (UDP-glucosyl transferase 73C6); UDP-glucosyltransferase/ UDP-glycosyltransferase/ transferase, transferring glycosyl groups
Les.2809.1.S1_at	BT014540.1	AT3G46660.1	TC24080	V	-0.81	-1.77	32	micro RNA, natural antisense etc	MULTIPLE HITS: (at3g46658,at3g46660). at3g46658: other RNA at3g46660: UDP-glucoronosyl/UDP-glucosyl transferase family protein
Les.3247.1.S1_at	BT013622.1	AT3G26380.1	TC22319	VI	-1.32	-1.03	3.8.2	minor CHO metabolism.galactose.alpha-galactosidases	(at3g26380): glycosyl hydrolase family protein 27 / alpha-galactosidase family protein / melibiase family protein

Les.24.1.S1_at	AF191823.1	AT5G08370.2	TC21735	V	-1.08	-2.72	3.8.2	minor CHO metabolism.galactose.alpha-galactosidases	(at5g08370): ATAGAL2 (ARABIDOPSIS THALIANA ALPHA-GALACTOSIDASE 2); alpha-galactosidase
LesAffx.53061.1.S1_at	BI208217	AT3G56310.2	TC23775	IV	-1.46	-0.64	3.8.2	minor CHO metabolism.galactose.alpha-galactosidases	(at3g56310): alpha-galactosidase, putative / melibiase, putative / alpha-D-galactoside galactohydrolase, putative
LesAffx.66966.1.S1_at	BG135649	AT3G56310.1	TC22051	IV	-1.45	-0.87	3.8.2	minor CHO metabolism.galactose.alpha-galactosidases	(at3g56310): alpha-galactosidase, putative / melibiase, putative / alpha-D-galactoside galactohydrolase, putative
Les.2012.1.S1_at	BG627650	AT2G22240.2	TC23373	VI	-1.26	-2.08	3.4.3	minor CHO metabolism.myo-inositol.InsP Synthases	(at2g22240): inositol-3-phosphate synthase isozyme 2 / myo-inositol-1-phosphate synthase 2 / MI-1-P synthase 2 / IPS 2
LesAffx.68107.1.S1_at	AI485479	AT3G28340.1	TC23454	III	1.46	1.46	3.1.1.2	minor CHO metabolism.raffinose family.galactinol synthases.putative	(at3g28340): GATL10 (Galacturonosyltransferase-like 10); polygalacturonate 4-alpha-galacturonosyltransferase/ transferase, transferring hexosyl groups
LesAffx.71377.1.S1_at	BG123375	AT5G51460.2	TC22940	I	1.98	1.14	3.2.2	minor CHO metabolism.trehalose.TPP	(at5g51460): ATTPPA (Arabidopsis thaliana trehalose-6-phosphate phosphatase); trehalose-phosphatase
Les.2985.1.A1_at	BG626023	AT4G25650.1	TC23280	VI	-0.9	-1.21	26.3	misc. other Ferredoxins and Rieske domain	(at4g25650): ACD1-LIKE; electron carrier
LesAffx.18686.1.S1_at	BI203812	AT1G73010.1	TC22685	II	1.11	2.72	26.13	misc.acid and other phosphatases	(at1g73010): phosphoric monoester hydrolase
Les.3368.1.S1_at	BT014303.1	AT5G34850.1	TC22868	VI	-1.21	-0.91	26.13	misc.acid and other phosphatases	(at5g34850): ATPAP26/PAP26 (purple acid phosphatase 26); acid phosphatase/ protein serine/threonine phosphatase
Les.3748.1.A1_at	AB023388.1	AT2G16430.1	TC22967	VI	-1.11	-1.29	26.13	misc.acid and other phosphatases	(at2g16430): PAP10; acid phosphatase/ protein serine/threonine phosphatase
Les.4975.1.S1_at	BT013032.1	AT4G25150.1	TC23130	V	-0.29	-1.9	26.13	misc.acid and other phosphatases	(at4g25150): acid phosphatase, putative
LesAffx.48215.1.S1_at	AW945008	AT1G18650.1	TC23968	VI	-1.88	-1.4	26.4	misc.beta 1,3 glucan hydrolases	(at1g18650): glycosyl hydrolase family protein 17
LesAffx.65984.1.S1_at	BG125327	AT2G05790.1	GO37430	VI	-1.64	-1.72	26.4.1	misc.beta 1,3 glucan hydrolases.glucan endo-1,3-beta-glucosidase	(at2g05790): glycosyl hydrolase family 17 protein

Les.2923.1.S1_at	BI925625	AT1G32860.1	TC23094	IV	-1.35	-0.67	26.4.1	misc.beta 1,3 glucan hydrolases.glucan endo-1,3-beta-glucosidase	(at1g32860): glycosyl hydrolase family 17 protein
LesAffx.39973.1.S1_at	BI932177	AT5G56590.1	DV10487	IV	-1.73	-1.16	26.4.1	misc.beta 1,3 glucan hydrolases.glucan endo-1,3-beta-glucosidase	(at5g56590): glycosyl hydrolase family 17 protein
LesAffx.22297.1.S1_at	AW041000	AT2G28850.1	TC21723	I	1	0.57	26.1	misc.cytochrome P450	MULTIPLE HITS: (at2g28860,at2g28850). at2g28860: CYP710A4 (cytochrome P450, family 710, subfamily A, polypeptide 4); oxygen binding at2g28850: CYP710A3 (cytochrome P450, family 710, subfamily A, polypeptide 3); oxygen binding
Les.3792.1.S1_s_at	AF249329.1	AT3G14610.1	GO37419	VI	-2.18	-2.23	26.1	misc.cytochrome P450	(at3g14610): CYP72A7 (cytochrome P450, family 72, subfamily A, polypeptide 7); oxygen binding
LesAffx.55225.1.A1_at	AJ785322	AT5G04660.1	TC23525	V	-0.43	-1.55	26.1	misc.cytochrome P450	(at5g04660): CYP77A4 (cytochrome P450, family 77, subfamily A, polypeptide 4); oxygen binding
LesAffx.24042.1.S1_at	CN385197	AT3G61040.1	DB67876	III	1.23	1.88	26.1	misc.cytochrome P450	(at3g61040): CYP76C7 (cytochrome P450, family 76, subfamily C, polypeptide 7); oxygen binding
LesAffx.3455.1.S1_at	BI421254	AT5G36140.1	TC23177	III	0.75	1.23	26.1	misc.cytochrome P450	(at5g36140): CYP716A2 (cytochrome P450, family 716, subfamily A, polypeptide 2); heme binding / iron ion binding / monooxygenase
LesAffx.8720.1.S1_at	BI921446	AT3G52970.1	BI92223	III	1.57	2.35	26.1	misc.cytochrome P450	(at3g52970): CYP76G1 (cytochrome P450, family 76, subfamily G, polypeptide 1); oxygen binding
Les.4880.1.S1_at	BT012820.1	AT2G40890.1	TC24340	IV	-1.23	-0.02	26.1	misc.cytochrome P450	(at2g40890): CYP98A3 (cytochrome P450, family 98, subfamily A, polypeptide 3); p-coumarate 3-hydroxylase
Les.3686.1.S1_at	AY081907.1	AT2G39030.1	TC21954	I	0.98	0.41	26.24	misc.GCN5-related N-acetyltransferase	(at2g39030): GCN5-related N-acetyltransferase (GNAT) family protein
Les.3261.1.A1_at	BG627816	AT4G37670.2	TC23483	VI	-0.94	-1.06	26.24	misc.GCN5-related N-acetyltransferase	(at4g37670): GCN5-related N-acetyltransferase (GNAT) family protein / amino acid kinase family protein

Les.254.1.S1_at	AY081905.1	AT2G39020.1	TC21784	III	1.1	1.31	26.24	misc.GCN5-related N-acetyltransferase	(at2g39020): GCN5-related N-acetyltransferase (GNAT) family protein
Les.4038.1.S1_at	AY081908.1	AT2G39020.1	TC21781	III	2.03	1.88	26.24	misc.GCN5-related N-acetyltransferase	(at2g39020): GCN5-related N-acetyltransferase (GNAT) family protein
LesAffx.55476.1.S1_at	BM410473	AT5G45920.1	TC22139	I	1.24	0.81	26.28	misc.GDSL-motif lipase	(at5g45920): carboxylic ester hydrolase
Les.5629.1.S1_at	BT014283.1	AT1G29670.1	TC24074	V	0.11	-1.08	26.28	misc.GDSL-motif lipase	(at1g29670): GDSL-motif lipase/hydrolase family protein
Les.810.1.S1_at	BG627575	AT5G45670.1	TC22972	V	-0.71	-2.22	26.28	misc.GDSL-motif lipase	(at5g45670): GDSL-motif lipase/hydrolase family protein
LesAffx.37344.2.S1_at	BG131634	AT1G28590.1	TC21881	V	-0.53	-1.41	26.28	misc.GDSL-motif lipase	(at1g28590): lipase, putative
LesAffx.26471.1.S1_at	BG126065	AT5G03610.1	TC22883	IV	-1.11	-0.4	26.28	misc.GDSL-motif lipase	(at5g03610): GDSL-motif lipase/hydrolase family protein
Les.4966.1.S1_at	BT013015.1	AT4G27830.1	BF17648	II	-0.07	1.22	26.3	misc.gluco-, galacto- and mannosidases	(at4g27830): glycosyl hydrolase family 1 protein
LesAffx.65060.1.A1_at	CK716180	AT1G61820.3	DV10448	VI	-1.13	-1.22	26.3	misc.gluco-, galacto- and mannosidases	(at1g61820): BGLU46; hydrolase, hydrolyzing O-glycosyl compounds
Les.1.1.S1_at	AF154421.1	AT3G13750.1	TC21715	II	0.42	2.1	26.3.2	misc.gluco-, galacto- and mannosidases.beta-galactosidase	(at3g13750): BGAL1 (BETA GALACTOSIDASE 1); beta-galactosidase
Les.4346.1.S1_at	AF020390.2	AT3G52840.1	TC21717	II	-0.29	1.78	26.3.2	misc.gluco-, galacto- and mannosidases.beta-galactosidase	(at3g52840): beta-galactosidase, putative / lactase, putative
Les.110.1.S1_at	AF154423.1	AT2G28470.1	TC22330	V	-0.81	-1.53	26.3.2	misc.gluco-, galacto- and mannosidases.beta-galactosidase	(at2g28470): BGAL8 (beta-glactosidase 8); beta-galactosidase
Les.109.1.S1_at	AF154424.1	AT4G36360.2	TC22392	IV	-1.11	-0.11	26.3.2	misc.gluco-, galacto- and mannosidases.beta-galactosidase	(at4g36360): BGAL3 (beta-galactosidase 3); beta-galactosidase
Les.3491.1.S1_at	AF077339.1	AT4G02290.1	TC23081	III	2.19	4.14	26.3.4	misc.gluco-, galacto- and mannosidases.endoglucanase	(at4g02290): glycosyl hydrolase family 9 protein
Les.3636.1.S1_at	Y11268.1	AT1G71380.1	TC23558	IV	-1.32	0.12	26.3.4	misc.gluco-, galacto- and mannosidases.endoglucanase	(at1g71380): glycosyl hydrolase family 9 protein
Les.68.2.S1_a_at	BT013727.1	AT4G11050.1	TC21796	IV	-2.14	-1.07	26.3.4	misc.gluco-, galacto- and mannosidases.endoglucanase	(at4g11050): endo-1,4-beta-glucanase, putative / cellulase, putative
Les.3735.1.S1_at	AY007561.1	AT2G29460.1	TC22594	II	1.54	3.19	26.9	misc.glutathione S transferases	(at2g29460): ATGSTU4 (GLUTATHIONE S-TRANSFERASE 22); glutathione transferase
Les.4501.1.S1_at	CK468710	AT3G09270.1	TC24359	II	0.42	1.32	26.9	misc.glutathione S transferases	(at3g09270): ATGSTU8 (Arabidopsis thaliana Glutathione S-transferase (class tau) 8); glutathione transferase
Les.1724.1.S1_at	AI780669	AT5G02790.1	TC22140	V	0.16	-1.06	26.9	misc.glutathione S transferases	(at5g02790): In2-1 protein, putative

LesAffx.57363.1.S1_at	AW035806	AT1G14550.1	TC21958	III	1.68	2.09	26.9	misc.glutathione S transferases	(at1g14550): anionic peroxidase, putative
Les.4287.1.S1_at	CB751564	AT5G62360.1	TC21784	III	2.48	3.31	26.18	misc.invertase/pectin methylesterase inhibitor family protein	(at5g62360): invertase/pectin methylesterase inhibitor family protein
LesAffx.58019.1.S1_at	BI929233	AT5G20740.1	TC22171	IV	-1.47	-0.17	26.18	misc.invertase/pectin methylesterase inhibitor family protein	(at5g20740): invertase/pectin methylesterase inhibitor family protein
LesAffx.70112.1.S1_at	AW092445	AT4G37560.1	TC22090	VI	-0.84	-1.31	26.1	misc.misc2	(at4g37560): formamidase, putative / formamide amidohydrolase, putative
LesAffx.29757.2.S1_at	AI898522	AT5G64250.1	TC23303	III	0.77	1	26.1	misc.misc2	(at5g64250): 2-nitropropane dioxygenase family / NPD family
Les.1465.1.S1_at	BG631822	AT5G64440.1	TC23566	VI	-1.42	-2.16	26.8	misc.nitrilases, *nitrile lyases, berberine bridge enzymes, reticuline oxidases, troponine	(at5g64440): amidase family protein
LesAffx.21101.1.S1_at	BI924446	AT1G72970.1	TC21935	V	-0.85	-1.89	26.8	misc.nitrilases, *nitrile lyases, berberine bridge enzymes, reticuline oxidases, troponine	(at1g72970): HTH (HOTHEAD); aldehyde-lyase
LesAffx.58326.1.A1_at	CD002272	AT2G23620.1	TC23714	III	1.35	1.88	26.8	misc.nitrilases, *nitrile lyases, berberine bridge enzymes, reticuline oxidases, troponine	(at2g23620): esterase, putative
LesAffx.58497.1.S1_at	BM411367	AT1G19250.1	TC23480	I	1.73	1.29	26.7	misc.oxidases - copper, flavone etc.	(at1g19250): FMO1 (FLAVIN-DEPENDENT MONOOXYGENASE 1); monooxygenase
LesAffx.44382.1.S1_at	BI921407	AT1G21850.1	GO37430	II	0.3	1.13	26.7	misc.oxidases - copper, flavone etc.	(at1g21850): SKS8 (SKU5 Similar 8); copper ion binding / oxidoreductase
Les.1478.1.S1_at	BG631500	AT1G23740.1	TC23304	V	-0.97	-2.05	26.7	misc.oxidases - copper, flavone etc.	(at1g23740): oxidoreductase, zinc-binding dehydrogenase family protein
LesAffx.54536.1.S1_at	BM409044	AT2G29720.1	TC23408	III	1.84	1.58	26.7	misc.oxidases - copper, flavone etc.	MULTIPLE HITS: (at2g35660,at2g29720). at2g35660: CTF2A; monooxygenase at2g29720: CTF2B; monooxygenase
LesAffx.32563.1.S1_at	AI778206	AT1G14130.1	TC22260	IV	-1.17	-0.13	26.7	misc.oxidases - copper, flavone etc.	(at1g14130): 2-oxoglutarate-dependent dioxygenase, putative
Les.252.1.S1_at	U35643.1	AT1G73680.1	DB68734	VI	-1.65	-1.14	26.14	misc.oxygenases	(at1g73680): pathogen-responsive alpha-dioxygenase, putative
Les.4999.1.A1_at	BT013077.1	AT2G37130.1	TC23341	I	1.41	1.01	26.12	misc.peroxidases	(at2g37130): peroxidase 21 (PER21) (P21) (PRXR5)
Les.2832.1.S1_at	CN384480	AT2G41480.1	TC21748	II	-0.62	1.26	26.12	misc.peroxidases	(at2g41480): peroxidase

Les.4976.1.S1_at	BT013033.1	AT4G36430.1	TC22335	II	0.16	1.5	26.12	misc.peroxidases	(at4g36430): peroxidase, putative
LesAffx.39.1.S1_at	CN385433	AT4G36430.1	TC22335	II	-1	1.34	26.12	misc.peroxidases	(at4g36430): peroxidase, putative
LesAffx.39466.1.S1_at	BI203178	AT5G42180.1	TC24216	II	0.8	2.01	26.12	misc.peroxidases	(at5g42180): peroxidase 64 (PER64) (P64) (PRXR4)
LesAffx.6103.1.S1_at	AW647641	AT3G21770.1	TC22739	II	0.08	1.22	26.12	misc.peroxidases	(at3g21770): peroxidase 30 (PER30) (P30) (PRXR9)
LesAffx.66953.1.S1_at	AW651552	AT2G18980.1	TC23604	III	0.71	1.22	26.12	misc.peroxidases	(at2g18980): peroxidase, putative
LesAffx.71606.1.S1_s_at	AI896002	AT2G37130.2	TC24027	III	1.51	1.34	26.12	misc.peroxidases	(at2g37130): peroxidase 21 (PER21) (P21) (PRXR5)
Les.2092.1.S1_at	BG626759	AT4G21960.1	TC22657	IV	-1.84	-0.79	26.12	misc.peroxidases	(at4g21960): PRXR1 (peroxidase 42); peroxidase
LesAffx.53517.1.S1_at	AI773309	AT1G71695.1	TC23424	IV	-1.11	-0.6	26.12	misc.peroxidases	(at1g71695): peroxidase 12 (PER12) (P12) (PRXR6)
Les.4988.1.S1_at	BT013056.1	AT1G72230.1	TC22946	V	0.11	-2.12	26.19	misc.plastocyanin-like	(at1g72230): plastocyanin-like domain-containing protein
Les.217.1.S1_at	AF243180.1	AT5G26330.1	TC22069	III	1.23	1.56	26.19	misc.plastocyanin-like	(at5g26330): plastocyanin-like domain-containing protein / mavicyanin, putative
Les.5523.1.S1_at	BT014071.1	AT5G15350.1	TC21750	III	0.79	1.01	26.19	misc.plastocyanin-like	(at5g15350): plastocyanin-like domain-containing protein
Les.3094.3.S1_at	BG125458	AT2G45180.1	TC23596	VI	-1.28	-1.75	26.21	misc.protease inhibitor/seed storage/lipid transfer protein (LTP) family protein	(at2g45180): protease inhibitor/seed storage/lipid transfer protein (LTP) family protein
Les.4312.2.S1_at	BG626083	AT2G45180.1	TC22795	VI	-1.72	-2.43	26.21	misc.protease inhibitor/seed storage/lipid transfer protein (LTP) family protein	(at2g45180): protease inhibitor/seed storage/lipid transfer protein (LTP) family protein
LesAffx.17924.1.S1_at	BG124217	AT5G64080.1	TC24101	VI	-1.34	-1.04	26.21	misc.protease inhibitor/seed storage/lipid transfer protein (LTP) family protein	(at5g64080): protease inhibitor/seed storage/lipid transfer protein (LTP) family protein
Les.2642.1.S1_at	CN384581	AT3G18280.1	TC22309	III	0.76	1.49	26.21	misc.protease inhibitor/seed storage/lipid transfer protein (LTP) family protein	(at3g18280): protease inhibitor/seed storage/lipid transfer protein (LTP) family protein
Les.5759.1.S1_at	BM956714	AT1G62790.1	TC22993	III	1.56	2.61	26.21	misc.protease inhibitor/seed storage/lipid transfer protein (LTP) family protein	(at1g62790): protease inhibitor/seed storage/lipid transfer protein (LTP) family protein
LesAffx.124.1.S1_at	AW443936	AT5G48485.1	TC23232	III	1.43	1.55	26.21	misc.protease inhibitor/seed storage/lipid transfer protein (LTP) family protein	(at5g48485): DIR1 (DEFECTIVE IN INDUCED RESISTANCE 1); lipid binding

LesAffx.67592.1.S1_at	AW093105	AT3G22600.1	TC22664	III	3.48	4.23	26.21	misc.protease inhibitor/seed storage/lipid transfer protein (LTP) family protein	(at3g22600): protease inhibitor/seed storage/lipid transfer protein (LTP) family protein
Les.1046.1.S1_at	X72730.1	AT3G55290.2	TC22785	I	1.96	1.28	26.22	misc.short chain dehydrogenase/reductase (SDR)	MULTIPLE HITS: (at3g55310,at3g55290). at3g55310: oxidoreductase at3g55290: short-chain dehydrogenase/reductase (SDR) family
LesAffx.1091.1.S1_at	BI933005	AT5G18210.1	TC22991	I	1.19	0.94	26.22	misc.short chain dehydrogenase/reductase (SDR)	(at5g18210): short-chain dehydrogenase/reductase (SDR) family protein
Les.2325.1.S1_at	BG625896	AT2G47140.1	TC22401	VI	-1.41	-0.96	26.22	misc.short chain dehydrogenase/reductase (SDR)	(at2g47140): short-chain dehydrogenase/reductase (SDR) family protein
LesAffx.50687.1.S1_at	AI773710	AT5G18210.1	TC22384	III	1.95	1.75	26.22	misc.short chain dehydrogenase/reductase (SDR)	(at5g18210): short-chain dehydrogenase/reductase (SDR) family protein
LesAffx.15898.1.S1_at	AW649847	AT2G43820.1	TC21728	I	1.02	0.31	26.2	misc.UDP glucosyl and glucoronyl transferases	(at2g43820): GT/UGT74F2 (UDP-GLUCOSYLTRANSFERASE 74F2); UDP-glucosyltransferase/ UDP-glycosyltransferase/ transferase, transferring glycosyl groups / transferase, transferring hexosyl groups
LesAffx.32379.1.S1_at	BI421517	AT3G50760.1	BI42151	I	1.19	0.66	26.2	misc.UDP glucosyl and glucoronyl transferases	(at3g50760): GATL2 (Galacturonosyltransferase-like 2); polygalacturonate 4-alpha-galacturonosyltransferase/ transferase, transferring glycosyl groups / transferase, transferring hexosyl groups
LesAffx.63776.1.S1_at	BM408779	AT5G17050.1	TC22982	V	-0.47	-1.45	26.2	misc.UDP glucosyl and glucoronyl transferases	(at5g17050): UDP-glucoronosyl/UDP-glucosyl transferase family protein
LesAffx.59059.1.S1_at	AW622913	AT1G70090.2	TC22426	III	1.29	1.13	26.2	misc.UDP glucosyl and glucoronyl transferases	(at1g70090): GATL9/LGT8 (Galacturonosyltransferase-like 9); polygalacturonate 4-alpha-galacturonosyltransferase/ transferase, transferring glycosyl groups / transferase, transferring hexosyl groups
Les.3069.1.S1_at	BG630752	AT2G22900.1	TC21938	IV	-1.99	-0.64	26.2	misc.UDP glucosyl and glucoronyl transferases	(at2g22900): galactosyl transferase GMA12/MNN10 family protein
Les.4222.1.S1_at	AY034149.1	AT1G32350.1	TC21756	I	1.93	1.52	9.4	mitochondrial electron transport / ATP synthesis.alternative oxidase	(at1g32350): AOX1D (ALTERNATIVE OXIDASE 1D); alternative oxidase
Les.4223.1.S1_at	AY034148.1	AT3G22360.1	TC21729	I	1.52	0.81	9.4	mitochondrial electron transport / ATP synthesis.alternative oxidase	(at3g22360): AOX1B (alternative oxidase 1B); alternative oxidase

LesAffx.9245.1.S1_at	BE463039	AT3G52730.1	TC24138	III	1.03	0.95	9.5	mitochondrial electron transport / ATP synthesis.cytochrome c reductase	(at3g52730): ubiquinol-cytochrome C reductase UQCRX/QCR9-like family protein
Les.4625.1.S1_at	BT013972.1	AT1G50940.1	AW03556	III	0.91	1.48	9.3	mitochondrial electron transport / ATP synthesis.electron transfer flavoprotein	(at1g50940): ETFALPHA (ELECTRON TRANSFER FLAVOPROTEIN ALPHA); FAD binding / electron carrier
Les.5350.1.A1_at	BT013752.1	AT4G28220.1	TC22353	VI	-0.91	-1.11	9.2.2	mitochondrial electron transport / ATP synthesis.NADH-DH.type II.external	(at4g28220): NDB1 (NAD(P)H DEHYDROGENASE B1); NADH dehydrogenase/ disulfide oxidoreductase
Les.224.1.A1_at	AJ277561.1	AT5G37600.1	TC21885	V	-0.57	-1.17	12.2.2	N-metabolism.ammonia metabolism.glutamine synthase	(at5g37600): ATGSR1 (Arabidopsis thaliana glutamine synthase clone R1); glutamate-ammonia ligase
LesAffx.17150.1.A1_at	AW039265	AT2G15620.1	TC23237	VI	-0.78	-1.2	12.1.2	N-metabolism.nitrate metabolism.nitrite reductase	(at2g15620): NIR1 (NITRITE REDUCTASE); ferredoxin-nitrate reductase
LesAffx.46085.1.S1_at	AW928724	AT5G25180.1	TC22038	VI	-2.09	-2.35	35.3	not assigned.disagreeing hits	MULTIPLE DISAGREEING HITS: (at5g25180,at5g25170). at5g25180: CYP71B14 (cytochrome P450, family 71, subfamily B, polypeptide 14); oxygen binding at5g25170: similar to unknown protein [Arabidopsis thaliana] (TAIR:AT2G25190.1); similar to hypothetical protein 25.t00041 [Brassica oleracea] (GB:ABD64953.1); similar to Os03g0198500 [Oryza sativa (japonica cultivar-group)] (GB:NP_001049279.1); contains InterPro domain Protein of unknown function DUF862, eukaryotic; (InterPro:IPR008580)
LesAffx.34363.1.S1_at	BE461912	AT4G38300.1	TC24206	I	1.21	0.74	35.1	not assigned.no ontology	MULTIPLE HITS: (at4g38300,at4g38650). at4g38300: glycosyl hydrolase family 10 protein at4g38650: glycosyl hydrolase family 10 protein
LesAffx.47116.1.S1_at	AW224087	AT5G40670.1	TC23403	I	1.33	1	35.1	not assigned.no ontology	(at5g40670): PQ-loop repeat family protein / transmembrane family protein
LesAffx.47529.1.S1_at	BG131749	AT1G68260.1	TC24163	I	1	0.61	35.1	not assigned.no ontology	(at1g68260): thioesterase family protein
LesAffx.63739.1.S1_at	AW033134	AT2G18360.1	TC21965	I	1.16	0.81	35.1	not assigned.no ontology	(at2g18360): hydrolase, alpha/beta fold family protein
LesAffx.68306.1.S1_at	BE461086	AT5G63130.1	TC23074	I	3.31	1.88	35.1	not assigned.no ontology	(at5g63130): octicosapeptide/Phox/Bem1p (PB1) domain-containing protein

Les.4254.1.S1_at	BG631257	AT1G16880.1	TC24002	II	0.35	1.11	35.1	not assigned.no ontology	(at1g16880): uridylyltransferase-related
Les.5567.1.S1_at	BT014164.1	AT3G05170.1	TC22700	II	0.11	0.97	35.1	not assigned.no ontology	(at3g05170): phosphoglycerate/bisphosphoglycerate mutase family protein
LesAffx.17773.1.S1_at	BF113622	AT2G32150.1	DB69166	II	0.18	1.11	35.1	not assigned.no ontology	(at2g32150): haloacid dehalogenase-like hydrolase family protein
LesAffx.344.10.S1_at	AI773570	AT2G04690.2	AI77357	II	0.6	1.48	35.1	not assigned.no ontology	(at2g04690): cellular repressor of E1A-stimulated genes (CREG) family
LesAffx.53053.1.S1_at	BI211180	AT1G76160.1	TC22538	II	0.46	0.98	35.1	not assigned.no ontology	(at1g76160): SKS5 (SKU5 Similar 5); copper ion binding / oxidoreductase
Les.4825.1.S1_at	BT012681.1	AT5G34940.1	TC22418	VI	-1.18	-0.89	35.1	not assigned.no ontology	(at5g34940): glycosyl hydrolase family 79 N-terminal domain-containing protein
Les.5255.1.S1_at	BT013579.1	AT1G21680.1	BP87874	VI	-1.11	-0.82	35.1	not assigned.no ontology	(at1g21680): similar to unknown protein [Arabidopsis thaliana] (TAIR:AT1G21670.1); similar to WD40 domain protein beta Propeller [Solibacter usitatus Ellin6076] (GB:YP_828764.1); similar to Os03g0840200 [Oryza sativa (japonica cultivar-group)] (GB:NP_001051846.1); similar to Os07g0638100 [Oryza sativa (japonica cultivar-group)] (GB:NP_001060406.1); contains InterPro domain TolB, C-terminal; (InterPro:IPR011042); contains InterPro domain WD40-like Beta Propeller; (InterPro:IPR011659)
Les.5786.1.S1_at	BT014563.1	AT3G05900.1	TC22614	VI	-1.25	-1.01	35.1	not assigned.no ontology	(at3g05900): neurofilament protein-related
Les.5861.1.S1_at	BF050616	AT5G16730.1	TC24194	VI	-0.96	-1.19	35.1	not assigned.no ontology	(at5g16730): similar to unknown protein [Arabidopsis thaliana] (TAIR:AT3G02930.1); similar to Protein kinase PKN/PRK1, effector [Medicago truncatula] (GB:ABE94710.1); contains InterPro domain Protein kinase PKN/PRK1, effector; (InterPro:IPR011072)
LesAffx.58110.1.A1_at	AJ785349	AT1G78070.2	BE43527	VI	-1.24	-1.49	35.1	not assigned.no ontology	(at1g78070): WD-40 repeat family protein
Les.3166.1.S1_at	AI775188	AT1G12570.1	TC23957	V	-0.29	-1.77	35.1	not assigned.no ontology	(at1g12570): glucose-methanol-choline (GMC) oxidoreductase family protein
Les.3924.1.S1_at	BE433308	AT2G14520.1	TC23702	V	-0.26	-1.82	35.1	not assigned.no ontology	(at2g14520): CBS domain-containing protein
Les.5301.1.S1_at	BI204978	AT1G59650.1	TC22541	V	-0.56	-1.15	35.1	not assigned.no ontology	(at1g59650): CW14

LesAffx.10091.1.S1_at	AI487223	AT1G24420.1	TC23911	V	-0.47	-2.93	35.1	not assigned.no ontology	(at1g24420): transferase family protein
LesAffx.37212.1.A1_at	AI491145	AT1G73480.1	TC23069	V	-0.71	-2.74	35.1	not assigned.no ontology	(at1g73480): hydrolase, alpha/beta fold family protein
LesAffx.41703.1.A1_at	BG352060	AT4G35250.1	TC23987	V	-0.17	-1.22	35.1	not assigned.no ontology	(at4g35250): vestitone reductase-related
LesAffx.5.1.S1_at	AW621769	AT1G42470.1	TC22795	V	-0.76	-1.46	35.1	not assigned.no ontology	(at1g42470): patched family protein
LesAffx.65757.1.S1_at	AW624713	AT4G13840.1	TC21840	V	-0.34	-1.2	35.1	not assigned.no ontology	(at4g13840): transferase family protein
Les.2565.1.S1_at	AW441199	AT2G25110.1	TC23474	III	0.66	1.01	35.1	not assigned.no ontology	(at2g25110): MIR domain-containing protein
Les.5014.1.S1_at	BT013105.1	AT4G14420.1	TC23486	III	1.37	1.69	35.1	not assigned.no ontology	(at4g14420): lesion inducing protein-related
Les.5481.1.A1_at	BT014001.1	AT4G14420.1	TC21763	III	1	0.87	35.1	not assigned.no ontology	(at4g14420): lesion inducing protein-related
LesAffx.30145.1.S1_at	AI775220	AT1G68610.1	ES89125	III	1.43	1.51	35.1	not assigned.no ontology	(at1g68610): similar to unknown protein [Arabidopsis thaliana] (TAIR:AT1G14870.1); similar to PGPS/D12 [Petunia x hybrida] (GB:AAD02554.1); contains InterPro domain Protein of unknown function Cys-rich; (InterPro:IPR006461)
LesAffx.49809.1.S1_at	CN384734	AT5G59480.1	TC21755	III	0.79	1.22	35.1	not assigned.no ontology	(at5g59480): haloacid dehalogenase-like hydrolase family protein
LesAffx.56301.1.S1_at	AI895341	AT2G22170.1	TC22182	III	2.26	2.31	35.1	not assigned.no ontology	(at2g22170): lipid-associated family protein
LesAffx.62967.1.S1_at	BG128004	AT3G52470.1	TC21896	III	1.02	0.97	35.1	not assigned.no ontology	(at3g52470): harpin-induced family protein / HIN1 family protein / harpin-responsive family protein
LesAffx.63489.1.S1_at	BI921137	AT4G15470.1	TC22082	III	2.75	2.87	35.1	not assigned.no ontology	(at4g15470): similar to glutamate binding [Arabidopsis thaliana] (TAIR:AT1G03070.1); similar to Os05g0402300 [Oryza sativa (japonica cultivar-group)] (GB:NP_001055493.1); similar to putative receptor associated protein [Capsicum chinense] (GB:CAI53895.2); contains InterPro domain Protein of unknown function UPF0005; (InterPro:IPR006214)
LesAffx.69783.1.S1_at	AW220917	AT3G55390.1	TC23448	III	1.65	1.78	35.1	not assigned.no ontology	(at3g55390): integral membrane family protein

LesAffx.8850.1.S1_at	AW034398	AT5G62180.1	TC22181	III	2.49	4.74	35.1	not assigned.no ontology	(at5g62180): similar to unknown protein [Arabidopsis thaliana] (TAIR:AT2G45600.1); similar to CXE carboxylesterase [Actinidia arguta] (GB:ABB89014.1); contains InterPro domain Esterase/lipase/thioesterase; (InterPro:IPR000379); contains InterPro domain Alpha/beta hydrolase fold-3; (InterPro:IPR013094)
Les.2626.1.S1_at	BG735008	AT4G39730.1	TC22245	IV	-1.38	-0.07	35.1	not assigned.no ontology	(at4g39730): lipid-associated family protein
Les.3819.1.S1_at	AY140893.1	AT2G18500.1	TC21842	IV	-1.23	-0.06	35.1	not assigned.no ontology	(at2g18500): ATOFP7/OFP7 (Arabidopsis thaliana ovate family protein 7)
Les.5263.1.S1_at	BT013600.1	AT1G76160.1	TC22756	IV	-1.08	-0.68	35.1	not assigned.no ontology	(at1g76160): SKS5 (SKU5 Similar 5); copper ion binding / oxidoreductase
Les.5333.1.S1_at	BT013726.1	AT1G28340.1	TC22354	IV	-1.45	-0.57	35.1	not assigned.no ontology	(at1g28340): leucine-rich repeat family protein
Les.5664.1.S1_at	BT014348.1	AT5G51550.1	AW03195	IV	-2.6	0.63	35.1	not assigned.no ontology	(at5g51550): phosphate-responsive 1 family protein
LesAffx.10346.1.A1_at	CK715067	AT4G22290.1	TC22642	IV	-1.18	-0.69	35.1	not assigned.no ontology	MULTIPLE HITS: (at4g22285,at4g22350). at4g22285: ubiquitin thiolesterase/ zinc ion binding at4g22350: ubiquitin carboxyl-terminal hydrolase family protein
LesAffx.39072.1.S1_at	BI211118	AT1G60670.2	TC22430	IV	-1.27	-0.8	35.1	not assigned.no ontology	(at1g60670): similar to unknown protein [Arabidopsis thaliana] (TAIR:AT1G10820.2); similar to Os04g0523100 [Oryza sativa (japonica cultivar-group)] (GB:NP_001053344.1); similar to H0322F07.7 [Oryza sativa (indica cultivar-group)] (GB:CAH67770.1); similar to OSIGBa0153E02-OSIGBa0093I20.5 [Oryza sativa (indica cultivar-group)] (GB:CAH67876.1); contains InterPro domain Homeodomain-like; (InterPro:IPR009057)
LesAffx.67955.1.S1_at	BM409816	AT4G16490.1	TC24493	VI	-0.8	-1.21	35.1.3	not assigned.no ontology.armadillo/beta-catenin repeat family protein	(at4g16490): armadillo/beta-catenin repeat family protein
Les.4852.1.S1_at	BT012761.1	AT1G63220.1	TC22901	III	1.18	1.45	35.1.19	not assigned.no ontology.C2 domain-containing protein	(at1g63220): C2 domain-containing protein

Les.3591.1.S1_at	CN385613	AT4G33930.1	FS19591	II	0.44	1.23	35.1.40	not assigned.no ontology.glycine rich proteins	MULTIPLE HITS: (at4g34300,at4g33930). at4g34300: glycine-rich protein at4g33930: glycine-rich protein
LesAffx.51285.1.A1_at	CN385118	AT2G15780.1	TC24451	III	1.01	1.09	35.1.40	not assigned.no ontology.glycine rich proteins	(at2g15780): glycine-rich protein
LesAffx.31451.1.S1_at	BM409478	AT3G53170.1	TC22107	VI	-1.17	-1.12	35.1.5	not assigned.no ontology.pentatricopeptide (PPR) repeat-containing protein	(at3g53170): pentatricopeptide (PPR) repeat-containing protein
LesAffx.37764.1.S1_at	CK715143	AT2G03880.1	CK71514	V	-0.29	-1.15	35.1.5	not assigned.no ontology.pentatricopeptide (PPR) repeat-containing protein	(at2g03880): pentatricopeptide (PPR) repeat-containing protein
Les.2073.1.S1_at	BM410320	AT3G07090.1	TC23644	I	1.12	0.45	35.2	not assigned.unknown	(at3g07090): similar to unknown protein [Arabidopsis thaliana] (TAIR:AT5G25170.1); similar to expressed protein [Oryza sativa (japonica cultivar-group)] (GB:ABB47783.1); similar to unknown protein [Oryza sativa] (GB:AAG16855.1); similar to Os02g0814000 [Oryza sativa (japonica cultivar-group)] (GB:NP_001048488.1); contains InterPro domain Protein of unknown function DUF862, eukaryotic; (InterPro:IPR008580)
Les.2417.1.S1_at	AI779519	AT3G47070.1	TC23220	I	1.33	0.84	35.2	not assigned.unknown	(at3g47070): similar to thylakoid soluble phosphoprotein [Spinacia oleracea] (GB:CAD45559.1)
Les.2682.1.S1_at	CN385606	AT5G17280.1	TC22504	I	1.36	0.81	35.2	not assigned.unknown	(at5g17280): similar to oxidoreductase [Trypanosoma cruzi strain CL Brener] (GB:XP_808855.1); similar to Os09g0530900 [Oryza sativa (japonica cultivar-group)] (GB:NP_001063752.1)
Les.4055.1.S1_at	BM536050	AT4G28290.1	TC23122	I	1.95	1.42	35.2	not assigned.unknown	(at4g28290): unknown protein
Les.423.1.S1_at	BM409421	AT5G02020.1	TC22953	I	1.77	1.18	35.2	not assigned.unknown	(at5g02020): similar to unknown protein [Arabidopsis thaliana] (TAIR:AT5G59080.1)
LesAffx.22466.1.S1_at	AI774712	AT3G19220.1	TC23276	I	1.22	0.81	35.2	not assigned.unknown	(at3g19220): similar to conserved hypothetical protein [Medicago truncatula] (GB:ABE80527.1)

LesAffx.26054.1.S1_at	BG135770	AT4G04630.1	TC21811	I	1.38	0.78	35.2	not assigned.unknown	(at4g04630): similar to unknown protein [Arabidopsis thaliana] (TAIR:AT4G21970.1); similar to Protein of unknown function, DUF584 [Medicago truncatula] (GB:ABE91657.1); contains InterPro domain Protein of unknown function DUF584; (InterPro:IPR007608)
LesAffx.37595.1.A1_at	CN385844	AT2G35290.1	TC22360	I	1.99	1.36	35.2	not assigned.unknown	(at2g35290): similar to Os03g0170100 [Oryza sativa (japonica cultivar-group)] (GB:NP_001049105.1)
LesAffx.56070.1.S1_at	BF113259	AT1G13340.1	TC23846	I	1.02	0.68	35.2	not assigned.unknown	(at1g13340): similar to unknown protein [Arabidopsis thaliana] (TAIR:AT4G35730.1); similar to unknown protein [Arabidopsis thaliana] (TAIR:AT1G34220.2); similar to expressed protein [Oryza sativa (japonica cultivar-group)] (GB:ABF95057.1); similar to Os03g0710000 [Oryza sativa (japonica cultivar-group)] (GB:NP_001051046.1); contains InterPro domain Protein of unknown function DUF292, eukaryotic; (InterPro:IPR005061)
LesAffx.59507.1.S1_at	BI203990	AT4G27350.1	TC21999	I	1.29	1.06	35.2	not assigned.unknown	(at4g27350): similar to unknown protein [Arabidopsis thaliana] (TAIR:AT5G54240.1); similar to membrane lipoprotein lipid attachment site-containing protein -like [Oryza sativa (japonica cultivar-group)] (GB:BAC84042.1); contains InterPro domain Protein of unknown function DUF1223; (InterPro:IPR010634)
Les.2643.1.S1_at	BG128842	AT3G03170.1	TC23690	II	0.58	1.44	35.2	not assigned.unknown	(at3g03170): similar to unknown protein [Arabidopsis thaliana] (TAIR:AT5G24890.1)
Les.5881.1.A1_at	CK716328	AT1G62045.1	TC24065	II	0.48	1.12	35.2	not assigned.unknown	(at1g62045): similar to ankyrin repeat family protein [Arabidopsis thaliana] (TAIR:AT1G11740.1); similar to conserved hypothetical protein [Medicago truncatula] (GB:ABE89099.1)

LesAffx.34771.1.S1_at	AW930136	AT1G05360.1	AW93013	II	0.46	1.51	35.2	not assigned.unknown	(at1g05360): similar to unknown protein [Arabidopsis thaliana] (TAIR:AT4G14950.1); similar to conserved hypothetical protein [Medicago truncatula] (GB:ABE91712.1); contains domain VACUOLE MEMBRANE PROTEIN 1 (PTHR10281:SF1); contains domain MEMBRANE ASSOCIATED PROGESTERONE RECEPTOR-RELATED (PTHR10281)
LesAffx.59625.1.S1_at	BI204607	AT5G48540.1	TC22866	II	0.49	1.83	35.2	not assigned.unknown	(at5g48540): 33 kDa secretory protein-related
LesAffx.60109.1.S1_at	BI204244	AT3G08030.2	TC22478	II	-0.05	1.65	35.2	not assigned.unknown	(at3g08030): similar to unknown protein [Arabidopsis thaliana] (TAIR:AT2G41800.1); similar to Os03g0807700 [Oryza sativa (japonica cultivar-group)] (GB:NP_001051644.1); similar to unknown [Ricinus communis] (GB:CAB02653.1); contains InterPro domain Galactose-binding like; (InterPro:IPR008979); contains InterPro domain Protein of unknown function DUF642; (InterPro:IPR006946)
LesAffx.62670.1.S1_at	BF113967	AT2G17710.1	TC22120	II	0.14	0.98	35.2	not assigned.unknown	(at2g17710): similar to Os04g0560700 [Oryza sativa (japonica cultivar-group)] (GB:NP_001053549.1)
LesAffx.64333.1.S1_at	CN384866	AT2G15890.1	TC21750	II	-0.46	1.19	35.2	not assigned.unknown	(at2g15890): MEE14 (maternal effect embryo arrest 14)
LesAffx.65882.1.S1_at	BG129533	AT4G28310.1	TC23537	II	-0.08	1.4	35.2	not assigned.unknown	(at4g28310): similar to unknown protein [Arabidopsis thaliana] (TAIR:AT1G52270.1); similar to unknown protein [Oryza sativa (japonica cultivar-group)] (GB:BAC22207.1)
Les.1501.1.A1_at	BG626168	AT1G11700.1	TC24040	VI	-1	-1.05	35.2	not assigned.unknown	(at1g11700): similar to unknown protein [Arabidopsis thaliana] (TAIR:AT1G61930.1); similar to Protein of unknown function DUF584 [Medicago truncatula] (GB:ABE80969.1); similar to Os07g0516300 [Oryza sativa (japonica cultivar-group)] (GB:NP_001059782.1); contains InterPro domain Protein of unknown function DUF584; (InterPro:IPR007608)

Les.4300.2.S1_at	AW217635	AT5G11420.1	TC21888	VI	-0.97	-1.04	35.2	not assigned.unknown	(at5g11420): similar to unknown protein [Arabidopsis thaliana] (TAIR:AT5G25460.1); similar to unknown [Ricinus communis] (GB:CAB02653.1); contains InterPro domain Galactose-binding like; (InterPro:IPR008979); contains InterPro domain Protein of unknown function DUF642; (InterPro:IPR006946)
Les.4938.1.S1_at	BT012940.1	AT2G32280.1	TC22310	VI	-2.5	-1.81	35.2	not assigned.unknown	(at2g32280): similar to unknown protein [Arabidopsis thaliana] (TAIR:AT4G21310.1); similar to Os07g0545100 [Oryza sativa (japonica cultivar-group)] (GB:NP_001059913.1); similar to Os05g0435100 [Oryza sativa (japonica cultivar-group)] (GB:NP_001055641.1); similar to hypothetical protein [Oryza sativa (japonica cultivar-group)] (GB:AAT47054.1); contains InterPro domain Protein of unknown function DUF1218; (InterPro:IPR009606)
Les.5696.1.S1_at	BT014400.1	AT1G29700.1	TC23901	VI	-0.69	-1.03	35.2	not assigned.unknown	(at1g29700): similar to similar to Zn-dependent hydrolases of the beta-lactamase fold [Crocosphaera watsonii WH 8501] (GB:ZP_00516888.1); similar to Os12g0641300 [Oryza sativa (japonica cultivar-group)] (GB:NP_001067402.1); contains domain no description (G3D.3.60.15.10); contains domain Metallo-hydrolase/oxidoreductase (SSF56281)
LesAffx.52453.1.S1_at	BG135842	AT5G59830.2	BI92407	VI	-0.92	-1.09	35.2	not assigned.unknown	(at5g59830): similar to unknown protein [Arabidopsis thaliana] (TAIR:AT5G13660.1); similar to predicted protein [Populus alba x Populus tremula] (GB:AAR14274.1)

LesAffx.52470.1.S1_at	BM412030	AT3G22970.1	TC23981	VI	-0.91	-1.05	35.2	not assigned.unknown	(at3g22970): similar to unknown protein [Arabidopsis thaliana] (TAIR:AT4G14620.1); similar to unknown protein [Oryza sativa (japonica cultivar-group)] (GB:AAP44687.1); similar to expressed protein [Oryza sativa (japonica cultivar-group)] (GB:AAT76408.1); similar to Os03g0796600 [Oryza sativa (japonica cultivar-group)] (GB:NP_001051556.1); contains InterPro domain Protein of unknown function DUF506, plant; (InterPro:IPR006502)
LesAffx.61000.1.S1_at	BM410987	AT2G38820.2	TC23050	VI	-1.71	-1.88	35.2	not assigned.unknown	(at2g38820): similar to unknown protein [Arabidopsis thaliana] (TAIR:AT3G22970.1); similar to unknown protein [Oryza sativa (japonica cultivar-group)] (GB:AAP44687.1); similar to expressed protein [Oryza sativa (japonica cultivar-group)] (GB:AAT76408.1); similar to Os03g0796600 [Oryza sativa (japonica cultivar-group)] (GB:NP_001051556.1); contains InterPro domain Protein of unknown function DUF506, plant; (InterPro:IPR006502)
LesAffx.64757.1.A1_at	AI490042	AT5G67390.2	TC22570	VI	-1.81	-1.51	35.2	not assigned.unknown	(at5g67390): similar to unknown protein [Arabidopsis thaliana] (TAIR:AT5G57340.1); similar to hypothetical protein MtrDRAFT_AC137703g30v2 [Medicago truncatula] (GB:ABE82530.1)
LesAffx.65306.1.S1_at	AW621362	AT5G61820.1	TC23246	VI	-0.91	-1.17	35.2	not assigned.unknown	(at5g61820): similar to hypothetical protein [Trifolium pratense] (GB:BAE71234.1); contains InterPro domain Stress up-regulated Nod 19; (InterPro:IPR011692)
LesAffx.69164.1.S1_at	AW649318	AT1G31130.1	TC22018	VI	-0.9	-1.14	35.2	not assigned.unknown	(at1g31130): similar to unknown protein [Arabidopsis thaliana] (TAIR:AT5G44860.1); similar to conserved hypothetical protein [Medicago truncatula] (GB:ABE90086.1); contains domain gb def: Hypothetical protein AT4g16850 (PTHR22597:SF10); contains domain FAMILY NOT NAMED (PTHR22597)

Les.3742.1.S1_at	AF146690.1	AT1G74950.1	TC22347	V	-0.84	-1.39	35.2	not assigned.unknown	(at1g74950): similar to unknown protein [Arabidopsis thaliana] (TAIR:AT1G19180.1); similar to PnFL-2 [Ipomoea nil] (GB:AAG49896.1); contains InterPro domain ZIM; (InterPro:IPR010399)
LesAffx.3537.1.A1_at	AJ784726	AT3G15810.1	TC24068	V	-0.49	-1.13	35.2	not assigned.unknown	(at3g15810): similar to unknown protein [Arabidopsis thaliana] (TAIR:AT1G80120.1); similar to Os01g0771000 [Oryza sativa (japonica cultivar-group)] (GB:NP_001044381.1); similar to hypothetical protein-like protein [Sorghum bicolor] (GB:AAO16702.1); similar to Os05g0510100 [Oryza sativa (japonica cultivar-group)] (GB:NP_001056006.1); contains InterPro domain Protein of unknown function DUF567; (InterPro:IPR007612)
LesAffx.38499.1.A1_at	BI209699	AT4G19160.1	TC22324	V	-0.47	-1.41	35.2	not assigned.unknown	(at4g19160): binding
LesAffx.48370.1.S1_at	AW929756	AT4G31530.1	TC22420	V	-0.4	-1.18	35.2	not assigned.unknown	(at4g31530): catalytic/ coenzyme binding
LesAffx.59375.1.A1_at	AI485882	AT5G02480.1	TC24048	V	-0.35	-1.14	35.2	not assigned.unknown	(at5g02480): similar to SLT1 (SODIUM- AND LITHIUM-TOLERANT 1) [Arabidopsis thaliana] (TAIR:AT2G37570.1); similar to SLT1 protein [Nicotiana tabacum] (GB:AAG39002.1); contains InterPro domain HSP20-like chaperone; (InterPro:IPR008978); contains InterPro domain Heat shock protein Hsp20; (InterPro:IPR002068)
LesAffx.6294.1.S1_at	BI210923	AT3G53400.1	TC22443	V	-0.18	-1.06	35.2	not assigned.unknown	(at3g53400): similar to unknown protein [Arabidopsis thaliana] (TAIR:AT5G03190.1); similar to conserved hypothetical protein [Medicago truncatula] (GB:ABD32814.1); contains domain ARM repeat (SSF48371); contains domain no description (G3D.3.40.50.1860)
LesAffx.65428.1.S1_at	BM411427	AT1G14690.2	TC22724	V	-0.64	-1.15	35.2	not assigned.unknown	no match

LesAffx.66847.1.S1_at	AI778064	AT1G04030.1	AI77806	V	-0.27	-1.09	35.2	not assigned.unknown	(at1g04030): similar to unknown protein [Arabidopsis thaliana] (TAIR:AT5G44040.1); similar to At1g04030 [Coffea canephora] (GB:ABD93477.2); similar to Os04g0511700 [Oryza sativa (japonica cultivar-group)] (GB:NP_001053294.1); similar to OSIGBa0157K09-H0214G12.18 [Oryza sativa (indica cultivar-group)] (GB:CAH68007.1)
LesAffx.67482.1.S1_at	BG123945	AT5G20270.1	TC23975	V	-0.15	-1.24	35.2	not assigned.unknown	(at5g20270): HHP1 (HEPTAHELICAL TRANSMEMBRANE PROTEIN1)
LesAffx.71003.1.S1_at	AI779846	AT1G64680.1	TC22050	V	-0.15	-1.14	35.2	not assigned.unknown	(at1g64680): similar to unknown protein [Arabidopsis thaliana] (TAIR:AT1G03055.1); similar to hypothetical protein LOC_Os11g37650 [Oryza sativa (japonica cultivar-group)] (GB:ABA94460.1); similar to Os08g0114100 [Oryza sativa (japonica cultivar-group)] (GB:NP_001060847.1)
Les.1159.1.S1_at	AW096608	AT3G52040.1	TC23740	III	1	0.96	35.2	not assigned.unknown	(at3g52040): similar to unknown [Hyacinthus orientalis] (GB:AAS20983.1)
Les.2631.2.S1_at	BI208990	AT3G01130.1	TC23151	III	0.66	1.02	35.2	not assigned.unknown	no match
Les.5130.1.S1_at	BT013342.1	AT1G22030.1	TC23433	III	1.11	1.59	35.2	not assigned.unknown	(at1g22030): similar to unknown protein [Arabidopsis thaliana] (TAIR:AT1G77855.1); similar to Os08g0104800 [Oryza sativa (japonica cultivar-group)] (GB:NP_001060781.1); similar to unknown protein [Oryza sativa (japonica cultivar-group)] (GB:BAD08928.1); similar to Os01g0875000 [Oryza sativa (japonica cultivar-group)] (GB:NP_001044961.1); contains domain RHOMBOID-RELATED (PTHR22936); contains domain UNCHARACTERIZED (PTHR22936:SF1)
Les.5804.1.S1_at	BT014601.1	AT5G24165.1	TC22840	III	0.68	1	35.2	not assigned.unknown	(at5g24165): similar to unknown protein [Arabidopsis thaliana] (TAIR:AT4G23885.1); similar to Os06g0530200 [Oryza sativa (japonica cultivar-group)] (GB:NP_001057777.1)

Les.638.1.S1_at	BT012771.1	AT1G07040.1	TC21816	III	1.02	1.04	35.2	not assigned.unknown	(at1g07040): similar to unknown protein [Arabidopsis thaliana] (TAIR:AT1G27030.1); similar to unknown protein [Oryza sativa (japonica cultivar-group)] (GB:AAN05517.1); similar to Os10g0463800 [Oryza sativa (japonica cultivar-group)] (GB:NP_001064789.1)
LesAffx.17017.1.S1_at	CN384671	AT5G17350.1	TC24170	III	1.57	1.48	35.2	not assigned.unknown	(at5g17350): similar to unknown protein [Arabidopsis thaliana] (TAIR:AT3G03280.1); similar to conserved hypothetical protein [Medicago truncatula] (GB:ABE92227.1); contains InterPro domain TGS-like; (InterPro:IPR012676)
LesAffx.3460.1.S1_at	CN384958	AT4G09890.1	EG55377	III	1.1	1.53	35.2	not assigned.unknown	(at4g09890): similar to unknown protein [Arabidopsis thaliana] (TAIR:AT5G11970.1); similar to conserved hypothetical protein [Medicago truncatula] (GB:ABE81195.1)
LesAffx.50466.1.S1_at	AI780511	AT2G20820.1	TC23061	III	1.1	1.2	35.2	not assigned.unknown	(at2g20820): similar to hypothetical protein [Capsicum chinense] (GB:CAJ13713.1)
LesAffx.57303.1.S1_at	AW625293	AT5G40690.1	TC22186	III	1.76	2.08	35.2	not assigned.unknown	(at5g40690): similar to unknown protein [Arabidopsis thaliana] (TAIR:AT2G41730.1)
LesAffx.60006.1.S1_at	BG643009	AT5G26230.1	TC22674	III	1.07	1.59	35.2	not assigned.unknown	(at5g26230): similar to unknown protein [Arabidopsis thaliana] (TAIR:AT5G42750.1); similar to Os05g0390600 [Oryza sativa (japonica cultivar-group)] (GB:NP_001055441.1); similar to PREDICTED: hypothetical protein [Rattus norve (GB:XP_001078830.1)
LesAffx.60171.1.S1_at	BG134335	AT5G14105.1	TC22657	III	0.65	1.01	35.2	not assigned.unknown	(at5g14105): similar to Os05g0517800 [Oryza sativa (japonica cultivar-group)] (GB:NP_001056054.1); similar to Os03g0733000 [Oryza sativa (japonica cultivar-group)] (GB:NP_001051172.1)

LesAffx.60242.1.S1_at	BI207558	AT2G16460.2	TC23660	III	1.09	0.98	35.2	not assigned.unknown	(at2g16460): similar to unknown protein [Arabidopsis thaliana] (TAIR:AT3G51090.1); similar to Os06g0713100 [Oryza sativa (japonica cultivar-group)] (GB:NP_001058564.1); similar to conserved hypothetical protein [Medicago truncatula] (GB:ABE89621.1); contains InterPro domain Protein of unknown function DUF1640; (InterPro:IPR012439)
LesAffx.61510.2.S1_at	BE433504	AT1G27200.1	TC23546	III	0.76	1.19	35.2	not assigned.unknown	(at1g27200): similar to zinc finger (C3HC4-type RING finger) family protein [Arabidopsis thaliana] (TAIR:AT3G27330.1); similar to Os08g0121900 [Oryza sativa (japonica cultivar-group)] (GB:NP_001060883.1); similar to zinc finger (C3HC4-type RING finger) protein family-like [Oryza sativa (japonica cultivar-group)] (GB:BAD10590.1); contains InterPro domain Protein of unknown function DUF23; (InterPro:IPR008166)
LesAffx.62690.1.S1_at	BI206793	AT5G11950.2	TC22770	III	0.97	1.72	35.2	not assigned.unknown	(at5g11950): Identical to Lysine decarboxylase-like protein At5g11950 [Arabidopsis Thaliana] (GB:Q84MC2;GB:Q570P8;GB:Q9LYH8); similar to unknown protein [Arabidopsis thaliana] (TAIR:AT2G37210.1); similar to Conserved hypothetical protein 730 [Medicago truncatula] (GB:ABE86251.1); contains InterPro domain Conserved hypothetical protein 730; (InterPro:IPR005269)
LesAffx.62803.1.S1_at	AW223168	AT1G29040.1	TC22502	III	0.94	0.88	35.2	not assigned.unknown	(at1g29040): similar to unknown protein [Oryza sativa (japonica cultivar-group)] (GB:BAD61226.1); similar to Os01g0613300 [Oryza sativa (japonica cultivar-group)] (GB:NP_001043561.1); contains InterPro domain Conserved hypothetical protein 2058; (InterPro:IPR011719)
LesAffx.63980.1.S1_at	AI780160	AT3G02800.1	TC22905	III	1.06	1.03	35.2	not assigned.unknown	(at3g02800): phosphoprotein phosphatase

LesAffx.68459.2.S1_at	BM411992	AT3G28630.1	TC22747	III	1.78	1.84	35.2	not assigned.unknown	(at3g28630): similar to unknown protein [Arabidopsis thaliana] (TAIR:AT1G59710.1); similar to Cytosolic fatty-acid binding; Actin-crosslinking proteins [Medicago truncatula] (GB:ABE82702.1); contains InterPro domain Protein of unknown function DUF569; (InterPro:IPR007679); contains InterPro domain Actin-crosslinking proteins; (InterPro:IPR008999)
LesAffx.69747.1.S1_at	BM536068	AT5G39890.1	TC21831	III	1.08	1.01	35.2	not assigned.unknown	(at5g39890): similar to unknown protein [Arabidopsis thaliana] (TAIR:AT5G15120.1); similar to unknown protein [Oryza sativa (japonica cultivar-group)] (GB:BAB03364.1); similar to Os01g0185500 [Oryza sativa (japonica cultivar-group)] (GB:NP_001042241.1); contains InterPro domain Protein of unknown function DUF1637; (InterPro:IPR012864); contains InterPro domain Cupin, RmlC-type; (InterPro:IPR011051)
LesAffx.69872.1.S1_at	CN385882	AT1G05575.1	TC23745	III	1.94	2.64	35.2	not assigned.unknown	(at1g05575): similar to unknown protein [Arabidopsis thaliana] (TAIR:AT2G31945.1); similar to hypothetical protein [Oryza sativa (japonica cultivar-group)] (GB:BAD38225.1); similar to Os06g0207200 [Oryza sativa (japonica cultivar-group)] (GB:NP_001057099.1); similar to Os02g0774200 [Oryza sativa (japonica cultivar-group)] (GB:NP_001048273.1)

LesAffx.71530.1.S1_at	BI930780	AT1G60060.1	TC23505	III	1.29	1.27	35.2	not assigned.unknown	(at1g60060): similar to unknown protein [Arabidopsis thaliana] (TAIR:AT5G53900.2); similar to Os02g0742700 [Oryza sativa (japonica cultivar-group)] (GB:NP_001048090.1); similar to unknown protein [Oryza sativa (japonica cultivar-group)] (GB:BAD37771.1); similar to Os06g0233800 [Oryza sativa (japonica cultivar-group)] (GB:NP_001057234.1); contains domain gb def: At3g15240 (PTHR13902:SF3); contains domain SERINE/THREONINE-PROTEIN KINASE WNK (WITH NO LYSINE)-RELATED (PTHR13902)
LesAffx.71544.1.S1_at	BI422043	AT5G11000.1	TC21855	III	0.88	1.04	35.2	not assigned.unknown	(at5g11000): similar to unknown protein [Arabidopsis thaliana] (TAIR:AT2G25200.1); similar to Os08g0439600 [Oryza sativa (japonica cultivar-group)] (GB:NP_001061898.1); similar to unknown protein [Oryza sativa (japonica cultivar-group)] (GB:BAD28921.1); contains InterPro domain Protein of unknown function DUF868, plant; (InterPro:IPR008586)
Les.2545.1.S1_at	BT014360.1	AT1G29980.1	TC22148	IV	-1.37	-0.36	35.2	not assigned.unknown	(at1g29980): similar to unknown protein [Arabidopsis thaliana] (TAIR:AT2G34510.1); similar to unknown [Ricinus communis] (GB:CAB02653.1); similar to Os01g0756600 [Oryza sativa (japonica cultivar-group)] (GB:NP_001044289.1); similar to Os01g0531400 [Oryza sativa (japonica cultivar-group)] (GB:NP_001043243.1); contains InterPro domain Galactose-binding like; (InterPro:IPR008979); contains InterPro domain Protein of unknown function DUF642; (InterPro:IPR006946)

Les.5223.1.S1_at	BT013527.1	AT2G12400.1	TC23738	IV	-1.29	-0.32	35.2	not assigned.unknown	(at2g12400): similar to unknown protein [Arabidopsis thaliana] (TAIR:AT2G25270.1); similar to H0306F03.12 [Oryza sativa (indica cultivar-group)] (GB:CAH68245.1); similar to Os04g0644000 [Oryza sativa (japonica cultivar-group)] (GB:NP_001054054.1); similar to Os02g0799300 [Oryza sativa (japonica cultivar-group)] (GB:NP_001048408.1)
Les.5419.1.S1_at	BT013877.1	AT5G53900.1	TC23973	IV	-1.38	-0.48	35.2	not assigned.unknown	(at5g53900): similar to unknown protein [Arabidopsis thaliana] (TAIR:AT3G15240.2); similar to Os11g0446000 [Oryza sativa (japonica cultivar-group)] (GB:NP_001067826.1); similar to expressed protein [Oryza sativa (japonica cultivar-group)] (GB:ABA93307.2); contains domain gb def: At3g15240 (PTHR13902:SF3); contains domain SERINE/THREONINE-PROTEIN KINASE WNK (WITH NO LYSINE)-RELATED (PTHR13902)
Les.744.1.S1_at	BT013096.1	AT1G72480.1	TC23212	IV	-1.09	-0.54	35.2	not assigned.unknown	(at1g72480): similar to unknown protein [Arabidopsis thaliana] (TAIR:AT2G01070.1); similar to Os11g0546100 [Oryza sativa (japonica cultivar-group)] (GB:NP_001068063.1); similar to Os09g0439700 [Oryza sativa (japonica cultivar-group)] (GB:NP_001063277.1); similar to Lung seven transmembrane receptor family protein, expressed [Oryza sativa (japonica cultivar-group)] (GB:ABF95807.1); contains InterPro domain Lung seven transmembrane receptor; (InterPro:IPR009637)

LesAffx.65028.1.S1_at	BG128587	AT1G13380.1	TC22082	IV	-1.55	-0.98	35.2	not assigned.unknown	(at1g13380): similar to unknown protein [Arabidopsis thaliana] (TAIR:AT4G27435.1); similar to Os09g0281300 [Oryza sativa (japonica cultivar-group)] (GB:NP_001062767.1); similar to fiber protein Fb34 [Gossypium barbadense] (GB:AAR07596.1); contains InterPro domain Protein of unknown function DUF1218; (InterPro:IPR009606)
Les.3935.1.S1_at	BT013403.1	AT5G28050.2	TC21790	III	1.37	1.28	23.2	nucleotide metabolism.degradation	(at5g28050): hydrolase/ zinc ion binding
LesAffx.62121.1.S1_at	AW220705	AT2G36310.1	TC24448	III	0.64	1.14	23.2	nucleotide metabolism.degradation	(at2g36310): inosine-uridine preferring nucleoside hydrolase family protein
LesAffx.69601.1.S1_at	AW030431	AT5G54390.1	TC21962	IV	-1.18	-0.62	23.2	nucleotide metabolism.degradation	(at5g54390): AHL (HAL2-LIKE); 3'(2'),5'-bisphosphate nucleotidase/ inositol or phosphatidylinositol phosphatase
Les.3597.1.S1_at	S40549.1	AT3G46940.1	TC22411	II	-0.96	1.36	23.5.5	nucleotide metabolism.deoxynucleotide metabolism.dUTP diphosphatase	(at3g46940): deoxyuridine 5'-triphosphate nucleotidohydrolase family
LesAffx.2226.1.S1_at	BI422950	AT3G27060.1	TC23401	III	1.04	1.21	23.5.4	nucleotide metabolism.deoxynucleotide metabolism.ribonucleoside-diphosphate reductase	(at3g27060): TSO2 (TSO2); ribonucleoside-diphosphate reductase
LesAffx.7602.1.S1_at	AW443858	AT5G63400.1	TC23283	III	1.37	1.18	23.4.1	nucleotide metabolism.phosphotransfer and pyrophosphatases.adenylate kinase	(at5g63400): ADK1 (ADENYLATE KINASE 1); adenylate kinase
LesAffx.63172.1.S1_at	AW031388	AT3G53620.1	TC22898	II	0.85	1.78	23.4.99	nucleotide metabolism.phosphotransfer and pyrophosphatases.misc	(at3g53620): inorganic pyrophosphatase, putative (soluble) / pyrophosphate phospho-hydrolase, putative / PPase, putative
Les.4471.1.S1_at	AF514776.1	AT1G72040.1	TC22151	VI	-0.81	-1.21	23.3	nucleotide metabolism.salvage	(at1g72040): deoxynucleoside kinase family
LesAffx.26180.1.S1_at	BG132890	AT1G68760.1	BG13289	II	1	2.13	23.3.3	nucleotide metabolism.salvage.NUDIX hydrolases	(at1g68760): ATNUDT1 (Arabidopsis thaliana Nudix hydrolase homolog 1); dihydroneopterin triphosphate pyrophosphohydrolase/ hydrolase
Les.4888.1.A1_at	BT012839.1	AT5G47650.1	AW39852	III	1.85	1.8	23.3.3	nucleotide metabolism.salvage.NUDIX hydrolases	(at5g47650): ATNUDT2 (Arabidopsis thaliana Nudix hydrolase homolog 2); ADP-ribose diphosphatase/ NAD binding / hydrolase

Les.4857.2.S1_at	BT013315.1	AT5G47240.1	TC24509	IV	-1.13	-0.23	23.3.3	nucleotide metabolism.salvage.NUDIX hydrolases	(at5g47240): ATNUDT8 (Arabidopsis thaliana Nudix hydrolase homolog 8); hydrolase
Les.2959.1.S1_at	BG734632	AT2G45290.1	TC22039	VI	-0.76	-1.14	7.2.1	OPP.non-reductive PP.transketolase	(at2g45290): transketolase, putative
Les.702.1.S1_at	BG627003	AT3G25570.1	TC21772	I	1.72	0.2	22.1.2	polyamine metabolism.synthesis.SAM decarboxylase	(at3g25570): adenosylmethionine decarboxylase family protein
Les.172.2.S1_a_at	BT014416.1	AT5G53120.3	TC22899	V	-0.66	-1.68	22.1.7	polyamine metabolism.synthesis.spermine synthase	(at5g53120): SPDS3 (SPERMIDINE SYNTHASE 3)
Les.4473.1.S1_at	AJ606077.1	AT5G19530.2	TC22116	IV	-1.59	-1.04	22.1.7	polyamine metabolism.synthesis.spermine synthase	(at5g19530): ACL5 (ACAULIS 5)
Les.4563.1.S1_at	BT014344.1	AT1G35340.3	TC23345	VI	-1.2	-0.88	29.5	protein.degradation	(at1g35340): ATP-dependent protease La (LON) domain-containing protein
Les.3266.2.S1_at	BI931445	AT4G17830.1	TC23669	V	0.02	-2.1	29.5	protein.degradation	(at4g17830): peptidase M20/M25/M40 family protein
Les.84.1.S1_at	U50151.1	AT2G24200.2	TC21723	V	-0.17	-2.77	29.5	protein.degradation	(at2g24200): cytosol aminopeptidase
Les.1042.1.A1_at	BG629025	AT1G10750.1	TC22972	IV	-1.52	-0.17	29.5	protein.degradation	(at1g10750): similar to unknown protein [Arabidopsis thaliana] (TAIR:AT1G70550.1); similar to unknown protein [Arabidopsis thaliana] (TAIR:AT1G23340.2); similar to putative carboxyl-terminal proteinase [Gossypium hirsutum] (GB:AAK84952.2); contains InterPro domain Protein of unknown function DUF239, plant; (InterPro:IPR004314)
Les.2068.1.A1_at	BG631401	AT3G45010.1	TC24040	IV	-1.19	-0.78	29.5	protein.degradation	(at3g45010): SCPL48 (serine carboxypeptidase-like 48); serine carboxypeptidase
LesAffx.16102.1.S1_at	BI208311	AT3G28510.1	TC23896	I	1.67	1.31	29.5.9	protein.degradation.AAA type	(at3g28510): AAA-type ATPase family protein
Les.4026.1.S1_at	AY155579.1	AT1G03220.1	TC21731	II	0.74	1.77	29.5.4	protein.degradation.aspartate protease	MULTIPLE HITS: (at1g03220,at1g03230). at1g03220: extracellular dermal glycoprotein, putative / EDGP, putative at1g03230: extracellular dermal glycoprotein, putative / EDGP, putative

Les.184.1.S1_at	L46681.1	AT1G11910.1	TC21791	VI	-0.84	-1.24	29.5.4	protein.degradation.aspartate protease	(at1g11910): aspartyl protease family protein
LesAffx.64206.1.S1_at	AW034232	AT1G01300.1	TC21738	VI	-1.34	-1.01	29.5.4	protein.degradation.aspartate protease	(at1g01300): aspartyl protease family protein
LesAffx.31022.1.S1_at	CK715372	AT4G16563.1	TC23408	V	-0.31	-1.17	29.5.4	protein.degradation.aspartate protease	(at4g16563): aspartyl protease family protein
Les.2513.1.S1_at	BI205120	AT4G16520.2	TC22383	II	0.36	1.07	29.5.2	protein.degradation.autophagy	(at4g16520): ATG8F (AUTOPHAGY 8F); microtubule binding
Les.2256.1.S1_at	BT014302.1	AT5G50260.1	GO37635	III	1.88	2.66	29.5.3	protein.degradation.cysteine protease	(at5g50260): cysteine proteinase, putative
Les.5711.1.S1_at	BT014429.1	AT4G39090.1	TC21831	III	1.15	2.11	29.5.3	protein.degradation.cysteine protease	(at4g39090): RD19 (RESPONSIVE TO DEHYDRATION 19); cysteine-type peptidase
LesAffx.1244.1.S1_at	BG126114	AT3G22260.2	TC22939	III	1.15	1.37	29.5.3	protein.degradation.cysteine protease	(at3g22260): OTU-like cysteine protease family protein
Les.3369.2.S1_at	AW934098	AT2G30950.1	TC23328	V	-0.58	-1.16	29.5.7	protein.degradation.metalloprotease	(at2g30950): VAR2 (VARIEGATED 2); ATP-dependent peptidase/ ATPase/ metallopeptidase/ zinc ion binding
LesAffx.63935.1.S1_at	AW442721	AT1G24140.1	TC23203	III	1.64	2.01	29.5.7	protein.degradation.metalloprotease	(at1g24140): matrixin family protein
Les.3515.1.S1_at	AF242849.1	AT3G25420.1	TC21891	V	-0.22	-1.9	29.5.5	protein.degradation.serine protease	(at3g25420): SCPL21 (serine carboxypeptidase-like 21); serine carboxypeptidase
Les.21.1.S1_at	AJ006377.1	AT5G51750.1	TC23436	VI	-1.16	-1.44	29.5.1	protein.degradation.subtilases	(at5g51750): subtilase family protein
Les.513.1.S1_at	BG626001	AT5G51750.1	TC22866	VI	-1.66	-2.13	29.5.1	protein.degradation.subtilases	(at5g51750): subtilase family protein
Les.3648.1.S1_at	X95270.1	AT5G67360.1	TC21719	V	-0.6	-1.43	29.5.1	protein.degradation.subtilases	(at5g67360): ARA12; subtilase
Les.15.1.S1_at	X98929.1	AT5G67360.1	TC21718	IV	-2.1	-0.53	29.5.1	protein.degradation.subtilases	(at5g67360): ARA12; subtilase
Les.3312.2.S1_at	BI932707	AT2G05920.1	TC21843	IV	-1.45	-0.79	29.5.1	protein.degradation.subtilases	(at2g05920): subtilase family protein
Les.3155.1.S1_at	BT013204.1	AT1G64230.2	TC21967	III	1.14	1.26	29.5.11.3	protein.degradation.ubiquitin.E2	(at1g64230): UBC28; ubiquitin-protein ligase
LesAffx.20391.1.S1_at	BF176422	AT1G23390.1	TC22123	I	1.01	0.49	29.5.11.4.3.	protein.degradation.ubiquitin.E3.SCF.FBOX	(at1g23390): kelch repeat-containing F-box family protein
LesAffx.54132.1.S1_at	BM535606	AT3G45620.1	TC23181	IV	-1.15	-0.71	29.5.11.4.9	protein.degradation.ubiquitin.E3.unspecified	(at3g45620): transducin family protein / WD-40 repeat family protein
Les.4819.1.S1_at	BT013586.1	AT5G02500.2	TC24267	I	1.86	0.27	29.6	protein.folding	(at5g02500): HSC70-1 (heat shock cognate 70 kDa protein 1); ATP binding
LesAffx.22583.1.S1_at	AI484022	AT1G77810.2	TC23670	I	1.95	1.48	29.7	protein.glycosylation	(at1g77810): galactosyltransferase family protein

LesAffx.41816.1.S1_at	AW649474	AT1G65590.1	TC22094	IV	-1.11	-0.45	29.7.8	protein.glycosylation.alpha-1,6-mannosyl-glycoprotein-beta-1,2-N-acetylglucosaminyltransferase(GnT II)	(at1g65590): glycosyl hydrolase family 20 protein
Les.2899.1.S1_at	BG628131	AT5G61640.1	TC22810	I	2.36	1.89	29.4	protein.postranslational modification	(at5g61640): PMSR1 (PEPTIDEMETHIONINE SULFOXIDE REDUCTASE 1); protein-methionine-S-oxide reductase
Les.31.1.S1_s_at	AF110518.1	AT5G03730.1	NP00011	I	1.2	0.36	29.4	protein.postranslational modification	(at5g03730): CTR1 (CONSTITUTIVE TRIPLE RESPONSE 1); kinase
LesAffx.47179.1.S1_at	BM411655	AT1G03920.1	TC24073	I	1.37	0.75	29.4	protein.postranslational modification	(at1g03920): protein kinase, putative
LesAffx.27440.1.S1_at	BM410131	AT1G68830.1	TC23837	VI	-0.94	-1.07	29.4	protein.postranslational modification	(at1g68830): STN7 (STT7 HOMOLOG STN7); kinase/ protein kinase
LesAffx.65695.2.A1_at	AI484733	AT5G40540.1	TC23451	VI	-1.16	-1.05	29.4	protein.postranslational modification	(at5g40540): protein kinase, putative
LesAffx.41333.2.S1_at	AW934014	AT1G07430.1	TC23167	V	0.03	-1.21	29.4	protein.postranslational modification	(at1g07430): protein phosphatase 2C, putative / PP2C, putative
LesAffx.66270.1.S1_at	BM410146	AT5G01820.1	TC23074	V	-0.51	-1.13	29.4	protein.postranslational modification	(at5g01820): ATSR1 (SERINE/THREONINE PROTEIN KINASE 1); kinase
Les.150.1.S1_at	BT013117.1	AT3G01490.1	AW65015	III	0.65	1.15	29.4	protein.postranslational modification	(at3g01490): protein kinase, putative
LesAffx.53506.1.S1_at	BI206456	AT5G59770.1	TC22555	IV	-1.06	0.15	29.4	protein.postranslational modification	(at5g59770): similar to PAS2 (PASTICCINO 2) [Arabidopsis thaliana] (TAIR:AT5G10480.2); similar to Protein tyrosine phosphatase-like protein, PTPLA [Medicago truncatula] (GB:ABE85916.1); contains InterPro domain Protein tyrosine phosphatase-like protein, PTPLA; (InterPro:IPR007482)
Les.5160.1.S1_at	BT013410.1	AT1G56720.3	TC23771	IV	-1.15	-0.17	29.4.1.55	protein.postranslational modification.kinase.receptor like cytoplasmatic kinase V	(at1g56720): protein kinase family protein
Les.4696.1.S1_at	BT014502.1	AT5G56460.1	TC22932	VI	-1.51	-1.33	29.4.1.57	protein.postranslational modification.kinase.receptor like cytoplasmatic kinase VII	(at5g56460): protein kinase, putative
Les.2571.1.S1_at	CN384497	AT5G56670.1	TC23740	III	0.83	0.99	29.2.1.2.1.3	protein.synthesis.ribosomal protein.eukaryotic.40S subunit.S30	(at5g56670): 40S ribosomal protein S30 (RPS30C)

LesAffx.58481.1.A1_at	BM535000	AT5G39785.2	TC23994	V	-0.34	-1.73	29.2.1.2.2.3	protein.synthesis.ribosomal protein.eukaryotic.60S subunit.L34	(at5g39785): similar to unknown protein [Arabidopsis thaliana] (TAIR:AT1G69610.1); similar to conserved hypothetical protein [Medicago truncatula] (GB:ABD32213.1); contains InterPro domain Protein of unknown function DUF1666; (InterPro:IPR012870)
Les.1444.1.S1_at	AI777910	AT5G24510.1	TC22510	III	0.9	1.11	29.2.1.2.2.8	protein.synthesis.ribosomal protein.eukaryotic.60S subunit.P1	(at5g24510): 60s acidic ribosomal protein P1, putative
LesAffx.13365.1.S1_at	BM411110	AT3G01740.1	TC21920	I	2.41	0.26	29.2.1.1.2.2	protein.synthesis.ribosomal protein.prokaryotic.mitochondrion. 50S subunit.L37	(at3g01740): similar to unknown protein [Arabidopsis thaliana] (TAIR:AT5G14290.1); similar to conserved hypothetical protein [Medicago truncatula] (GB:ABE80353.1)
Les.4542.1.A1_at	BT014515.1	AT5G03940.1	DB71533	VI	-0.7	-1.1	29.3.3	protein.targeting.chloroplast	(at5g03940): FFC (FIFTY-FOUR CHLOROPLAST HOMOLOGUE); 7S RNA binding / GTP binding / mRNA binding
Les.2568.1.S1_at	BI922309	AT2G24820.1	TC23413	V	-0.54	-1.38	29.3.3	protein.targeting.chloroplast	(at2g24820): TIC55 (TRANSLOCON AT THE INNER ENVELOPE MEMBRANE OF CHLOROPLASTS 55); oxidoreductase
Les.2364.1.S1_at	CN385609	AT1G61570.1	TC23659	III	0.94	0.94	29.3.2	protein.targeting.mitochondria	(at1g61570): TIM13 (TIM13); protein translocase
Les.4096.1.S1_at	AW220281	AT5G50810.1	TC22846	III	0.67	1.15	29.3.2	protein.targeting.mitochondria	(at5g50810): TIM8 (Arabidopsis thaliana translocase inner membrane subunit 8); protein translocase
LesAffx.53698.1.S1_at	BG127688	AT3G24570.1	EG55334	I	1.31	1.03	29.3.5	protein.targeting.peroxisomes	(at3g24570): peroxisomal membrane 22 kDa family protein
LesAffx.43793.1.A1_at	BI206057	AT3G25160.1	TC23097	III	1.05	1.19	29.3.4.1	protein.targeting.secretory pathway.ER	(at3g25160): ER lumen protein retaining receptor family protein
LesAffx.52595.1.S1_at	AI778688	AT1G72280.1	TC22841	III	1.37	1.41	29.3.4.1	protein.targeting.secretory pathway.ER	(at1g72280): AERO1 (ARABIDOPSIS ENDOPLASMIC RETICULUM OXIDOREDUCTINS 1)
Les.3086.2.S1_at	CN385936	AT3G60540.2	TC22850	III	0.92	1.03	29.3.4.99	protein.targeting.secretory pathway.unspecified	(at3g60540): sec61beta family protein
Les.3415.3.S1_at	AI779314	AT2G47400.1	TC24218	II	-0.41	1.35	1.3	PS.calvin cyle	(at2g47400): CP12-1 (CP12 domain-containing protein 1)
Les.3174.1.S1_at	BT012701.1	AT4G38970.1	TC22568	V	0.01	-1.32	1.3.6	PS.calvin cyle.aldolase	(at4g38970): fructose-bisphosphate aldolase, putative

Les.2024.1.A1_at	BG631036	AT3G54050.1	TC23275	V	-0.88	-1.52	1.3.7	PS.calvin cyle.FBPase	(at3g54050): fructose-1,6-bisphosphatase, putative / D-fructose-1,6-bisphosphate 1-phosphohydrolase, putative / FBPase, putative
Les.3072.1.S1_at	BG627918	AT1G12900.2	TC23388	VI	-0.79	-1.1	1.3.4	PS.calvin cyle.GAP	(at1g12900): GAPA-2; glyceraldehyde-3-phosphate dehydrogenase
Les.2888.1.S1_at	AI491181	AT1G12900.1	TC22589	V	-0.63	-1.53	1.3.4	PS.calvin cyle.GAP	(at1g12900): GAPA-2; glyceraldehyde-3-phosphate dehydrogenase
Les.2933.1.S1_at	BT012764.1	AT1G42970.1	TC23454	V	-1.15	-2	1.3.4	PS.calvin cyle.GAP	(at1g42970): GAPB (GLYCERALDEHYDE-3-PHOSPHATE DEHYDROGENASE B SUBUNIT); glyceraldehyde-3-phosphate dehydrogenase
Les.424.1.S1_at	BG628349	AT1G32060.1	TC22847	V	-0.66	-2.02	1.3.12	PS.calvin cyle.PRK	(at1g32060): PRK (PHOSPHORIBULOKINASE); ATP binding / phosphoribulokinase/ protein binding
LesAffx.50610.1.S1_at	AI775987	AT4G20130.1	TC24497	VI	-1.18	-1.11	1.3.13	PS.calvin cyle.rubisco interacting	(at4g20130): PTAC14 (PLASTID TRANSCRIPTIONALLY ACTIVE14)
Les.4493.1.S1_at	BG734807	AT2G39730.3	TC22690	V	-0.83	-1.53	1.3.13	PS.calvin cyle.rubisco interacting	(at2g39730): RCA (RUBISCO ACTIVASE)

Les.376.1.S1_at	BG627516	AT1G67090.1	TC24390	V	-0.64	-1.3	1.3.2	PS.calvin cyle.rubisco small subunit	MULTIPLE HITS: (at5g38420,at5g38430,at5g38410,at1g67090). at5g38420: ribulose bisphosphate carboxylase small chain 2B / RuBisCO small subunit 2B (RBCS-2B) (ATS2B) at5g38430: ribulose bisphosphate carboxylase small chain 1B / RuBisCO small subunit 1B (RBCS-1B) (ATS1B) at5g38410: ribulose bisphosphate carboxylase small chain 3B / RuBisCO small subunit 3B (RBCS-3B) (ATS3B); Identical to Ribulose bisphosphate carboxylase small chain 3B, chloroplast precursor (RBCS-3B) [Arabidopsis Thaliana] (GB:P10798;GB:Q9FF21); similar to ribulose bisphosphate carboxylase small chain 2B / RuBisCO small subunit 2B (RBCS-2B) (ATS2B) [Arabidopsis thaliana] (TAIR:AT5G38420.1); similar to Ribulose bisphosphate carboxylase small chain, chloroplast precursor (RuBisCO small subunit) (GB:P08135); contains InterPro domain Ribulose bisphosphate carboxylase, small chain; (InterPro:IPR000894) at1g67090: ribulose bisphosphate carboxylase small chain 1A / RuBisCO small subunit 1A (RBCS-1A) (ATS1A)
Les.4615.1.S1_at	BE462687	AT2G31040.1	TC22537	IV	-1.23	0.01	1.1.4	PS.lightreaction.ATP synthase	(at2g31040): ATP synthase protein I -related
Les.1832.1.A1_at	BG629451	AT2G05620.1	TC23307	V	0.62	-1.56	1.1.40	PS.lightreaction.cyclic electron flow-chlororespiration	(at2g05620): PGR5 (PROTON GRADIENT REGULATION 5)
Les.3234.1.A1_at	BG735047	AT1G60950.1	TC23423	V	-0.06	-1.05	1.1.5.2	PS.lightreaction.other electron carrier (ox/red).ferredoxin	(at1g60950): FED A (FERREDOXIN 2); electron carrier/ iron ion binding
LesAffx.5776.1.S1_at	BG127982	AT3G09150.2	BG12798	VI	-1.43	-1.14	1.1.5.4	PS.lightreaction.other electron carrier (ox/red).ferredoxin oxireductase	(at3g09150): HY2 (ELONGATED HYPOCOTYL 2); phytochromobilin:ferredoxin oxidoreductase
Les.4259.1.S1_at	BG629037	AT1G61520.2	TC24424	VI	-1.37	-1.51	1.1.2.1	PS.lightreaction.photosystem I.LHC-I	(at1g61520): LHCA3 (Photosystem I light harvesting complex gene 3); chlorophyll binding

Les.3297.1.S1_at	BG630303	AT3G47470.1	TC21776	V	-1.18	-2.2	1.1.2.1	PS.lightreaction.photosystem I.LHC-I	(at3g47470): LHCA4 (Photosystem I light harvesting complex gene 4); chlorophyll binding
Les.4083.1.S1_at	BG735070	AT3G61470.1	TC22596	V	-0.6	-1.25	1.1.2.1	PS.lightreaction.photosystem I.LHC-I	MULTIPLE HITS: (at3g61470,at5g28450). at3g61470: LHCA2 (Photosystem I light harvesting complex gene 2); chlorophyll binding at5g28450: chlorophyll A-B binding protein, chloroplast, putative / LHCI type II CAB, putative
Les.3038.1.S1_at	AW094037	AT2G46820.2	TC22677	VI	-0.66	-1.08	1.1.2.2	PS.lightreaction.photosystem I.PSI polypeptide subunits	(at2g46820): TMP14 (THYLAKOID MEMBRANE PHOSPHOPROTEIN OF 14 KDA)
Les.2168.1.S1_at	BT013274.1	AT5G64040.1	TC23964	V	-1.3	-2.6	1.1.2.2	PS.lightreaction.photosystem I.PSI polypeptide subunits	(at5g64040): PSAN (photosystem I reaction center subunit PSI-N); calmodulin binding
Les.4508.1.S1_s_at	AI780576	AT1G08380.1	BG12438	V	-0.59	-2.1	1.1.2.2	PS.lightreaction.photosystem I.PSI polypeptide subunits	(at1g08380): PSAO (photosystem I subunit O)
Les.5157.1.S1_at	BT013405.1	AT1G08380.1	TC23882	V	-0.48	-1.69	1.1.2.2	PS.lightreaction.photosystem I.PSI polypeptide subunits	(at1g08380): PSAO (photosystem I subunit O)
Les.4492.2.S1_at	CK714954	AT1G15820.1	TC22356	VI	-0.77	-1.1	1.1.1.1	PS.lightreaction.photosystem II.LHC-II	(at1g15820): LHCB6 (LIGHT HARVESTING COMPLEX PSII); chlorophyll binding
Les.147.1.S1_at	BG629070	AT3G27690.1	TC22473	V	-0.35	-1.95	1.1.1.1	PS.lightreaction.photosystem II.LHC-II	(at3g27690): LHCB2:4 (Photosystem II light harvesting complex gene 2.3); chlorophyll
Les.233.1.S1_at	M17558.1	AT3G27690.1	TC23200	V	-0.87	-1.86	1.1.1.1	PS.lightreaction.photosystem II.LHC-II	(at3g27690): LHCB2:4 (Photosystem II light harvesting complex gene 2.3); chlorophyll
Les.3016.1.S1_at	BT012757.1	AT5G54270.1	TC24030	V	-1.31	-2.74	1.1.1.1	PS.lightreaction.photosystem II.LHC-II	(at5g54270): LHCB3 (LIGHT-HARVESTING CHLOROPHYLL BINDING PROTEIN 3)
Les.3054.1.S1_at	AI773693	AT5G54270.1	TC23429	V	-1.16	-2.7	1.1.1.1	PS.lightreaction.photosystem II.LHC-II	(at5g54270): LHCB3 (LIGHT-HARVESTING CHLOROPHYLL BINDING PROTEIN 3)
Les.608.1.S1_at	BG628276	AT4G10340.1	TC21796	V	-0.69	-1.41	1.1.1.1	PS.lightreaction.photosystem II.LHC-II	(at4g10340): LHCB5 (LIGHT HARVESTING COMPLEX OF PHOTOSYSTEM II 5); chlorophyll binding
Les.4007.1.S1_at	BG629658	AT3G50820.1	TC22991	VI	-1.38	-1.03	1.1.1.2	PS.lightreaction.photosystem II.PSII polypeptide subunits	(at3g50820): PSBO-2/PSBO2 (PHOTOSYSTEM II SUBUNIT O-2); oxygen evolving
Les.482.1.S1_at	BG630028	AT1G06680.1	TC22380	VI	-0.8	-1.03	1.1.1.2	PS.lightreaction.photosystem II.PSII polypeptide subunits	(at1g06680): PSBP-1 (OXYGEN-EVOLVING ENHANCER PROTEIN 2); calcium ion binding
Les.1923.1.S1_at	BG630193	AT1G77090.1	TC23497	V	-0.56	-1.54	1.1.1.2	PS.lightreaction.photosystem II.PSII polypeptide subunits	(at1g77090): thylakoid lumenal 29.8 kDa protein

Les.3073.1.S1_at	AJ785415	AT1G44575.1	TC21760	V	-0.71	-1.41	1.1.1.2	PS.lightreaction.photosystem II.PSII polypeptide subunits	(at1g44575): NPQ4 (NONPHOTOCHEMICAL QUENCHING)
LesAffx.17387.1.S1_at	BM534931	AT1G06620.1	BM53493	III	0.95	1.4	21.2	redox.ascorbate and glutathione	(at1g06620): 2-oxoglutarate-dependent dioxygenase, putative
LesAffx.69349.2.S1_at	AW623862	AT4G18260.1	AW62386	III	1.87	1.95	21.2	redox.ascorbate and glutathione	(at4g18260): cytochrome B561-related
LesAffx.3918.1.S1_at	BM410706	AT3G09640.2	TC21958	V	0.2	-1.27	21.2.1	redox.ascorbate and glutathione.ascorbate	(at3g09640): APX2 (ASCORBATE PEROXIDASE 2); L-ascorbate peroxidase
Les.3214.1.S1_at	BG631018	AT5G28840.1	TC21991	VI	-1.02	-1.19	21.2.1.1	redox.ascorbate and glutathione.ascorbate.GME	(at5g28840): GME (GDP-D-MANNOSE 3',5'-EPIMERASE); GDP-mannose 3,5-epimerase/ NAD binding / catalytic
Les.3549.1.A1_at	AF096252.1	AT4G35090.2	TC23183	V	-0.56	-1.29	21.6	redox.dismutases and catalases	(at4g35090): CAT2 (CATALASE 2); catalase
Les.4270.1.S1_at	AF112368.1	AT4G35090.1	TC23183	V	-0.55	-1.21	21.6	redox.dismutases and catalases	(at4g35090): CAT2 (CATALASE 2); catalase
Les.170.1.S1_at	AY026344.1	AT3G10520.1	TC22471	II	0.28	1.07	21.3	redox.heme	(at3g10520): AHB2 (NON-SYMBIOTIC HAEMOGLOBIN 2)
Les.3700.1.S1_at	AY026343.1	AT2G16060.1	TC22149	II	0.24	1.74	21.3	redox.heme	(at2g16060): AHB1 (ARABIDOPSIS HEMOGLOBIN 1)
Les.5597.1.S1_at	BT014226.1	AT1G77510.1	AW09450	II	0.17	1.49	21.1	redox.thioredoxin	(at1g77510): ATPDIL1-2 (PDI-LIKE 1-2); protein disulfide isomerase
LesAffx.66600.1.A1_at	AW036163	AT3G02730.1	TC23173	V	-0.32	-1.12	21.1	redox.thioredoxin	(at3g02730): ATF1/TRXF1 (THIOREDOXIN F-TYPE 1); thiol-disulfide exchange intermediate
LesAffx.55662.1.S1_at	AW625299	AT1G07960.1	TC24518	III	0.99	1.05	21.1	redox.thioredoxin	(at1g07960): ATPDIL5-1 (PDI-LIKE 5-1); thiol-disulfide exchange intermediate
Les.50.1.S1_at	X79338.1	AT2G02990.1	TC22030	II	0.84	2.2	27.1.19	RNA.processing.ribonucleases	(at2g02990): RNS1 (RIBONUCLEASE 1); endoribonuclease
Les.5958.1.S1_at	CN384731	AT3G44260.1	TC23315	III	1.22	1.39	27.1.19	RNA.processing.ribonucleases	(at3g44260): CCR4-NOT transcription complex protein, putative
Les.3210.1.S1_at	AI483637	AT1G26820.1	TC22662	V	-0.25	-1.59	27.1.19	RNA.processing.ribonucleases	(at1g26820): RNS3 (RIBONUCLEASE 3); endoribonuclease
Les.3286.1.S1_at	BT013249.1	AT3G20390.1	TC23425	V	0.06	-2.18	27.1.19	RNA.processing.ribonucleases	(at3g20390): endoribonuclease L-PSP family protein
LesAffx.20450.1.S1_at	AW223514	AT3G16770.1	TC21776	III	1.27	1.11	27.3.3	RNA.regulation of transcription.AP2/EREBP, APETALA2/Ethylene-responsive element binding protein family	(at3g16770): ATEBP/RAP2.3 (RELATED TO AP2 3); DNA binding / protein binding / transcription factor/ transcriptional activator

LesAffx.64978.1.S1_at	CN385434	AT5G67190.1	FS18759	III	1.32	1.17	27.3.3	RNA.regulation of transcription.AP2/EREBP, APETALA2/Ethylene-responsive element binding protein family	(at5g67190): AP2 domain-containing transcription factor, putative
Les.305.2.S1_at	CD002775	AT2G27040.1	TC23749	VI	-1.58	-1.72	27.3.36	RNA.regulation of transcription.Argonaute	(at2g27040): AGO4 (ARGONAUTE 4)
LesAffx.16472.1.S1_at	AI774397	AT1G31320.1	TC24204	IV	-2.14	-0.56	27.3.37	RNA.regulation of transcription.AS2,Lateral Organ Boundaries Gene Family	(at1g31320): LOB domain protein 4 / lateral organ boundaries domain protein 4 (LBD4)
Les.3706.1.A1_at	AF022014.1	AT4G14560.1	TC21953	II	0.12	1.21	27.3.40	RNA.regulation of transcription.Aux/IAA family	(at4g14560): IAA1 (INDOLE-3-ACETIC ACID INDUCIBLE); transcription factor
Les.5442.1.S1_at	BT013931.1	AT4G32280.1	TC22814	III	2.11	3.53	27.3.40	RNA.regulation of transcription.Aux/IAA family	(at4g32280): IAA29 (indoleacetic acid-induced protein 29); transcription factor
Les.4097.1.A1_at	AF022018.1	AT4G14550.1	TC22516	VI	-1.3	-1.49	27.3.40	RNA.regulation of transcription.Aux/IAA family	(at4g14550): IAA14 (SOLITARY ROOT); transcription factor
Les.4085.1.S1_at	BE459897	AT2G27230.2	TC23484	VI	-0.89	-1.45	27.3.6	RNA.regulation of transcription.bHLH,Basic Helix-Loop-Helix family	(at2g27230): transcription factor-related
LesAffx.64675.1.S1_a_at	CN385903	AT5G57150.3	TC21939	VI	-1.17	-0.82	27.3.6	RNA.regulation of transcription.bHLH,Basic Helix-Loop-Helix family	(at5g57150): basic helix-loop-helix (bHLH) family protein
Les.3543.1.S1_at	AJ011914.1	AT5G11260.1	TC23261	I	1.09	0.75	27.3.35	RNA.regulation of transcription.bZIP transcription factor family	(at5g11260): HY5 (ELONGATED HYPOCOTYL 5); DNA binding / transcription factor
Les.5916.1.S1_at	AJ715788.1	AT3G62420.1	TC23545	III	1.24	1.25	27.3.35	RNA.regulation of transcription.bZIP transcription factor family	(at3g62420): ATBZIP53 (BASIC REGION/LEUCINE ZIPPER MOTIF 53); DNA binding / sequence-specific DNA binding / transcription factor
LesAffx.65689.1.A1_at	AI486980	AT5G24800.1	TC22449	V	-0.14	-1.14	27.3.35	RNA.regulation of transcription.bZIP transcription factor family	(at5g24800): BZO2H2 (basic leucine zipper O2 homolog 2); DNA binding / transcription factor
LesAffx.67368.1.S1_at	BG126810	AT5G06950.4	BG12681	V	-0.88	-1.53	27.3.35	RNA.regulation of transcription.bZIP transcription factor family	(at5g06950): AHBP-1B (bZIP transcription factor HBP-1b homolog); DNA binding / transcription factor

Les.3376.1.A1_at	BG630058	AT1G06040.1	TC21732	V	-0.46	-2.49	27.3.7	RNA.regulation of transcription.C2C2(Zn) CO-like, Constans-like zinc finger family	(at1g06040): STO (SALT TOLERANCE); transcription factor/ zinc ion binding
LesAffx.18330.1.S1_at	BG129699	AT5G57660.1	TC24342	V	-0.67	-1.52	27.3.7	RNA.regulation of transcription.C2C2(Zn) CO-like, Constans-like zinc finger family	(at5g57660): zinc finger (B-box type) family protein
LesAffx.71242.2.S1_at	AW093460	AT1G73870.1	TC22502	V	-0.9	-1.64	27.3.7	RNA.regulation of transcription.C2C2(Zn) CO-like, Constans-like zinc finger family	(at1g73870): zinc finger (B-box type) family protein
LesAffx.71503.1.S1_at	AW648310	AT5G60200.1	TC22254	IV	-1.51	-0.84	27.3.8	RNA.regulation of transcription.C2C2(Zn) DOF zinc finger family	(at5g60200): Dof-type zinc finger domain-containing protein
LesAffx.14110.2.A1_at	AW932597	AT3G47500.1	TC23949	V	-0.77	-1.81	27.3.8	RNA.regulation of transcription.C2C2(Zn) DOF zinc finger family	(at3g47500): CDF3 (CYCLING DOF FACTOR 3); DNA binding / protein binding / transcription factor
LesAffx.70928.1.S1_at	BI209612	AT3G24050.1	TC23336	III	1.08	1.07	27.3.9	RNA.regulation of transcription.C2C2(Zn) GATA transcription factor family	(at3g24050): GATA transcription factor 1 (GATA-1)
LesAffx.54718.1.S1_at	BM411779	AT3G54810.1	TC22617	VI	-1.26	-0.99	27.3.9	RNA.regulation of transcription.C2C2(Zn) GATA transcription factor family	(at3g54810): BME3/BME3-ZF (BLUE MICROPLYLAR END3); transcription factor
LesAffx.22830.1.S1_at	CN385777	AT3G28210.1	TC22584	I	1.7	1.28	27.3.11	RNA.regulation of transcription.C2H2 zinc finger family	(at3g28210): PMZ; zinc ion binding
LesAffx.71311.1.S1_at	CN384672	AT2G37430.1	TC22685	I	1.39	0.55	27.3.11	RNA.regulation of transcription.C2H2 zinc finger family	(at2g37430): zinc finger (C2H2 type) family protein (ZAT11)
Les.5126.1.S1_at	BT013336.1	AT3G19580.2	TC22310	III	1.49	1.55	27.3.11	RNA.regulation of transcription.C2H2 zinc finger family	(at3g19580): AZF2 (ARABIDOPSIS ZINC-FINGER PROTEIN 2); nucleic acid binding / transcription factor/ zinc ion binding
LesAffx.36193.1.S1_at	AW034622	AT5G04340.1	TC22914	III	1.57	1.7	27.3.11	RNA.regulation of transcription.C2H2 zinc finger family	(at5g04340): C2H2; nucleic acid binding / transcription factor/ zinc ion binding
LesAffx.51298.1.S1_at	BI422808	AT2G28710.1	TC21917	III	1.22	2.01	27.3.11	RNA.regulation of transcription.C2H2 zinc finger family	(at2g28710): zinc finger (C2H2 type) family protein

Les.2608.1.S1_at	BT014160.1	AT3G02830.1	TC23356	V	-0.6	-1.07	27.3.11	RNA.regulation of transcription.C2H2 zinc finger family	(at3g02830): ZFN1 (ZINC FINGER PROTEIN 1); nucleic acid binding
LesAffx.68092.1.S1_at	BE459012	AT3G46640.2	TC22815	III	0.89	1.79	27.3.20	RNA.regulation of transcription.G2-like transcription factor family, GARP	MULTIPLE HITS: (at5g59570,at3g46640). at5g59570: myb family transcription factor at3g46640: PCL1 (PHYTOCLOCK 1); DNA binding / transcription factor
LesAffx.51158.1.S1_at	AI897068	AT2G18550.1	TC23014	I	1.4	0.98	27.3.22	RNA.regulation of transcription.HB,Homeobox transcription factor family	(at2g18550): ATHB21/HB-2 (homeobox-2); DNA binding / transcription factor
LesAffx.2632.2.S1_at	BI205756	AT2G22800.1	TC23448	II	0.08	0.99	27.3.22	RNA.regulation of transcription.HB,Homeobox transcription factor family	(at2g22800): HAT9 (homeobox-leucine zipper protein 9); DNA binding / transcription factor
Les.5941.1.A1_at	CK715706	AT1G52150.2	TC22893	IV	-2.11	-0.94	27.3.22	RNA.regulation of transcription.HB,Homeobox transcription factor family	(at1g52150): ATHB-15 (INCURVATA 4); DNA binding / transcription factor
Les.70.1.S1_at	AF000141.1	AT1G62360.1	NP00014	VI	-1.62	-1.34	27.3.22	RNA.regulation of transcription.HB,Homeobox transcription factor family	(at1g62360): STM (SHOOT MERISTEMLESS); transcription factor
LesAffx.24696.1.S1_at	AW034874	AT2G26150.1	DB70637	IV	-1.23	0.43	27.3.23	RNA.regulation of transcription.HSF,Heat-shock transcription factor family	(at2g26150): ATHSFA2 (Arabidopsis thaliana heat shock transcription factor A2); DNA binding / transcription factor
LesAffx.56.6.S1_at	BM536076	AT3G22830.1	TC23980	IV	-1.17	0.32	27.3.23	RNA.regulation of transcription.HSF,Heat-shock transcription factor family	(at3g22830): AT-HSFA6B (Arabidopsis thaliana heat shock transcription factor A6B); DNA binding / transcription factor
Les.3833.1.S1_at	AY098737.2	AT1G71692.1	TC22193	III	1.14	1.79	27.3.24	RNA.regulation of transcription.MADS box transcription factor family	(at1g71692): AGL12 (AGAMOUS-LIKE 12); transcription factor
Les.5091.1.A1_at	BT013255.1	AT3G23250.1	TC22888	I	1.28	0.08	27.3.25	RNA.regulation of transcription.MYB domain transcription factor family	(at3g23250): AtMYB15/AtY19/MYB15 (myb domain protein 15); DNA binding / transcription factor
LesAffx.62138.1.S1_at	AW737374	AT5G49620.1	TC24364	III	1.69	1.74	27.3.25	RNA.regulation of transcription.MYB domain transcription factor family	(at5g49620): AtMYB78 (myb domain protein 78); DNA binding / transcription factor

Les.33.1.S1_at	AF148934.1	AT2G37630.1	TC22018	IV	-2.26	-0.53	27.3.25	RNA.regulation of transcription.MYB domain transcription factor family	(at2g37630): AS1 (ASYMMETRIC LEAVES 1); DNA binding / transcription factor
LesAffx.53591.1.S1_at	AI899018	AT1G06180.1	TC22292	VI	-2.05	-1.68	27.3.25	RNA.regulation of transcription.MYB domain transcription factor family	(at1g06180): ATMYB13 (myb domain protein 13); DNA binding / transcription factor
LesAffx.54522.1.S1_at	BM411853	AT2G23290.1	TC22000	VI	-0.9	-1.4	27.3.25	RNA.regulation of transcription.MYB domain transcription factor family	(at2g23290): AtMYB70 (myb domain protein 70); DNA binding / transcription factor
LesAffx.70738.1.A1_at	BG630069	AT5G40350.1	TC23199	VI	-1.77	-1.8	27.3.25	RNA.regulation of transcription.MYB domain transcription factor family	(at5g40350): MYB24 (myb domain protein 24); DNA binding / transcription factor
Les.4895.1.S1_at	BT012856.1	AT5G04760.1	TC23595	I	1.02	0.38	27.3.26	RNA.regulation of transcription.MYB-related transcription factor family	(at5g04760): myb family transcription factor
Les.2886.1.A1_at	BG628514	AT5G17300.1	TC24034	V	0.62	-1.28	27.3.26	RNA.regulation of transcription.MYB-related transcription factor family	(at5g17300): myb family transcription factor
Les.4923.1.S1_at	BT012912.1	AT1G01060.4	TC22925	V	0.22	-2.9	27.3.26	RNA.regulation of transcription.MYB-related transcription factor family	(at1g01060): LHY (LATE ELONGATED HYPOCOTYL); DNA binding / transcription factor
LesAffx.56221.1.S1_at	AI894713	AT3G09600.2	TC24290	V	-0.29	-2.94	27.3.26	RNA.regulation of transcription.MYB-related transcription factor family	(at3g09600): myb family transcription factor
LesAffx.1959.2.S1_at	AW216683	AT1G74840.1	TC23208	VI	-1.1	-0.83	27.3.26	RNA.regulation of transcription.MYB-related transcription factor family	(at1g74840): myb family transcription factor
LesAffx.50112.1.S1_at	AI898985	AT5G09800.1	TC23361	III	1.36	1.51	27.3.64	RNA.regulation of transcription.PHOR1	(at5g09800): U-box domain-containing protein
LesAffx.63659.1.S1_at	BI921813	AT3G18710.1	TC22794	III	1.53	1.55	27.3.64	RNA.regulation of transcription.PHOR1	(at3g18710): U-box domain-containing protein
Les.2949.1.S1_at	AJ515747.1	AT2G29570.1	TC21956	II	0.17	1.03	27.3.67	RNA.regulation of transcription.putative transcription regulator	(at2g29570): PCNA2 (PROLIFERATING CELL NUCLEAR 2); DNA binding / DNA polymerase processivity factor

LesAffx.20929.1.S1_at	BI206813	AT2G41870.1	TC23621	V	-0.39	-1.52	27.3.67	RNA.regulation of transcription.putative transcription regulator	(at2g41870): remorin family protein
LesAffx.66014.1.S1_at	BI926363	AT5G14260.1	TC24281	VI	-0.75	-1.04	27.3.69	RNA.regulation of transcription.SET-domain transcriptional regulator family	(at5g14260): SET domain-containing protein
LesAffx.43198.1.S1_at	BF113993	AT5G67480.2	BF11399	III	1.72	2.38	27.3.72	RNA.regulation of transcription.Transcriptional Adaptor Zinc Bundle (TAZ) domain family	(at5g67480): BT4 (BTB AND TAZ DOMAIN PROTEIN 4); protein binding
LesAffx.44609.1.S1_at	BG631285	AT3G10040.1	TC23469	IV	-2.11	-0.57	27.3.30	RNA.regulation of transcription.Trihelix, Triple-Helix transcription factor family	(at3g10040): transcription factor
Les.4920.1.S1_at	BT012902.1	AT1G47490.1	TC23329	III	0.85	1	27.3.99	RNA.regulation of transcription.unclassified	MULTIPLE HITS: (at1g47500,at1g47490). at1g47500: ATRBP47C' (RNA-BINDING PROTEIN 47C'); RNA binding at1g47490: ATRBP47C; RNA binding
LesAffx.61034.1.S1_at	BG643132	AT1G32540.3	TC21983	IV	-1.91	-0.67	27.3.99	RNA.regulation of transcription.unclassified	(at1g32540): LOL1 (LSD ONE LIKE 1)
LesAffx.4981.1.S1_at	AW623022	AT3G54400.1	TC22429	VI	-1.89	-2.27	27.3.99	RNA.regulation of transcription.unclassified	(at3g54400): aspartyl protease family protein
Les.2667.2.S1_at	AW621251	AT2G38470.1	TC22610	I	1.02	0.55	27.3.32	RNA.regulation of transcription.WRKY domain transcription factor family	(at2g38470): WRKY33 (WRKY DNA-binding protein 33); transcription factor
LesAffx.735.1.S1_at	AW217158	AT2G38470.1	TC22685	I	1.77	1.33	27.3.32	RNA.regulation of transcription.WRKY domain transcription factor family	(at2g38470): WRKY33 (WRKY DNA-binding protein 33); transcription factor
LesAffx.837.1.S1_at	AJ831935	AT3G56400.1	TC22608	III	0.88	1.12	27.3.32	RNA.regulation of transcription.WRKY domain transcription factor family	(at3g56400): WRKY70 (WRKY DNA-binding protein 70); transcription factor
LesAffx.9910.1.S1_at	CN385590	AT1G80840.1	TC22147	III	1.76	1.66	27.3.32	RNA.regulation of transcription.WRKY domain transcription factor family	(at1g80840): WRKY40 (WRKY DNA-binding protein 40); transcription factor
Les.3962.1.A1_at	BG630092	AT2G30590.1	TC24180	IV	-1.05	-0.65	27.3.32	RNA.regulation of transcription.WRKY domain transcription factor family	(at2g30590): WRKY21 (WRKY DNA-binding protein 21); transcription factor

LesAffx.57811.1.A1_at	AI488979	AT4G26640.1	AI48897	VI	-0.9	-1.13	27.3.32	RNA.regulation of transcription.WRKY domain transcription factor family	(at4g26640): WRKY20 (WRKY DNA-binding protein 20); transcription factor
LesAffx.35954.1.S1_at	AW092295	AT3G28920.1	AW09229	II	0.47	1.07	27.3.80	RNA.regulation of transcription.zf-HD	(at3g28920): ATHB34 (ARABIDOPSIS THALIANA HOMEOBOX PROTEIN 34); DNA binding / transcription factor
Les.928.1.S1_at	BG735170	AT2G37220.1	TC22279	IV	-1.08	0.23	27.4	RNA.RNA binding	(at2g37220): 29 kDa ribonucleoprotein, chloroplast, putative / RNA-binding protein cp29, putative
LesAffx.66331.1.S1_at	BI421673	AT5G24120.1	TC21932	V	-0.32	-1.61	27.2	RNA.transcription	(at5g24120): SIGE (RNA polymerase sigma subunit E); DNA binding / DNA-directed RNA polymerase/ sigma factor/ transcription factor
Les.61.1.S1_at	Y10403.1	AT1G14790.1	TC22152	VI	-1.12	-1.14	27.2	RNA.transcription	(at1g14790): RDR1 (RNA-DEPENDENT RNA POLYMERASE 1); RNA-directed RNA polymerase/ nucleic acid binding
Les.4885.1.S1_at	BT012835.1	AT2G39980.1	TC24138	I	3.53	2.69	16.8.1	secondary metabolism.flavonoids.anthocyanins	(at2g39980): transferase family protein
LesAffx.70136.2.S1_at	AI899680	AT4G09500.1	TC23538	V	-1.15	-2.26	16.8.1	secondary metabolism.flavonoids.anthocyanins	(at4g09500): glycosyltransferase family protein
LesAffx.61149.1.S1_at	BE460847	AT5G49690.1	TC24084	III	1.48	1.94	16.8.1.12	secondary metabolism.flavonoids.anthocyanins.anthocyanidin 3-O-glucosyltransferase	(at5g49690): UDP-glucoronosyl/UDP-glucosyl transferase family protein
Les.1205.1.A1_at	BG630057	AT3G29590.1	TC21911	VI	-1.83	-2.04	16.8.1.21	secondary metabolism.flavonoids.anthocyanins.anthocyanin 5-aromatic acyltransferase	(at3g29590): AT5MAT; O-malonyltransferase/ transferase
LesAffx.68320.1.S1_at	BM535633	AT5G05270.2	ES89343	V	-0.17	-1.19	16.8.2	secondary metabolism.flavonoids.chalcones	(at5g05270): chalcone-flavanone isomerase family protein
LesAffx.56389.1.S1_at	AW034707	AT1G59960.1	TC23934	III	3	3.16	16.8.2	secondary metabolism.flavonoids.chalcones	(at1g59960): aldo/keto reductase, putative
Les.3649.1.S1_at	AJ785484	AT5G13930.1	TC23379	V	-0.78	-2.31	16.8.2.1	secondary metabolism.flavonoids.chalcones.naringenin-chalcone synthase	(at5g13930): CHS (CHALCONE SYNTHASE); naringenin-chalcone synthase

Les.5864.1.S1_at	U20596.1	AT4G10490.1	TC23921	VI	-1.07	-1.63	16.8.3	secondary metabolism.flavonoids.dihydroflavonols	(at4g10490): oxidoreductase, 2OG-Fe(II) oxygenase family protein
Les.842.1.S1_at	BI931450	AT1G01420.1	TC23080	VI	-1.13	-1.63	16.8.3	secondary metabolism.flavonoids.dihydroflavonols	(at1g01420): UDP-glucoronosyl/UDP-glucosyl transferase family protein
LesAffx.29801.1.A1_at	AI489456	AT5G24530.1	TC23187	V	0.24	-1.17	16.8.3	secondary metabolism.flavonoids.dihydroflavonols	(at5g24530): oxidoreductase, 2OG-Fe(II) oxygenase family protein
Les.5601.1.S1_at	BT014233.1	AT1G17020.1	TC22079	VI	-1.12	-1.59	16.8.4	secondary metabolism.flavonoids.flavonols	(at1g17020): SRG1 (SENESCENCE-RELATED GENE 1); oxidoreductase, acting on paired donors, with incorporation or reduction of molecular oxygen, 2-oxoglutarate as one donor, and incorporation of one atom each of oxygen into both donors
Les.3085.1.S1_at	BM413323	AT5G08640.1	TC22204	V	-0.54	-1.95	16.8.4	secondary metabolism.flavonoids.flavonols	(at5g08640): FLS (FLAVONOL SYNTHASE)
Les.3545.1.S1_at	Y14809.1	AT4G25700.1	TC22310	I	1.14	0.16	16.1.4	secondary metabolism.isoprenoids.carotenoids	(at4g25700): BETA-OHASE 1 (BETA-HYDROXYLASE 1); beta-carotene hydroxylase
Les.3544.1.S1_at	AW930004	AT5G52570.1	TC21734	V	0.02	-1.19	16.1.4	secondary metabolism.isoprenoids.carotenoids	(at5g52570): BETA-OHASE 2 (BETA-CAROTENE HYDROXYLASE 2); beta-carotene hydroxylase
Les.3644.1.S1_at	X86452.1	AT3G10230.1	TC23849	V	-0.68	-1.23	16.1.4.5	secondary metabolism.isoprenoids.carotenoids.lycopene beta cyclase	(at3g10230): LYC (LYCOPENE CYCLASE); lycopene beta cyclase
Les.3771.1.S1_at	Y14387.1	AT5G57030.1	TC22876	IV	-1.28	-0.68	16.1.4.4	secondary metabolism.isoprenoids.carotenoids.lycopene epsilon cyclase	(at5g57030): LUT2 (LUTEIN DEFICIENT 2); lycopene epsilon cyclase
Les.3958.1.A1_at	AF385366.1	AT1G08550.2	TC23696	VI	-0.98	-1.11	16.1.4.21	secondary metabolism.isoprenoids.carotenoids.violaxanthin de-epoxidase	(at1g08550): NPQ1 (NON-PHOTOCHEMICAL QUENCHING 1)
Les.4735.1.S1_at	BT013429.1	AT4G11820.2	TC21799	I	0.99	0.46	16.1.2.2	secondary metabolism.isoprenoids.mevalonate pathway.HMG-CoA synthase	(at4g11820): BAP1 (hydroxymethylglutaryl-CoA synthase)

Les.3123.1.S1_at	BT013406.1	AT1G74470.1	TC23016	VI	-1.59	-1.48	16.1.1	secondary metabolism.isoprenoids.non-mevalonate pathway	(at1g74470): geranylgeranyl reductase
Les.6.1.S1_at	AF261141.1	AT3G57030.1	TC22389	VI	-1.54	-1.54	16.4.1	secondary metabolism.N misc.alkaloid-like	(at3g57030): strictosidine synthase family protein
LesAffx.60369.1.S1_at	BG130091	AT3G23490.1	TC22835	II	0.46	1.16	16.4.3.1	secondary metabolism.N misc.cyanogenic glycosides	(at3g23490): CYN (CYANASE); cyanate hydratase
LesAffx.66478.1.S1_at	AI773775	AT1G77670.1	TC22999	I	0.95	0.46	16.2	secondary metabolism.phenylpropanoids	(at1g77670): aminotransferase class I and II family protein
LesAffx.12150.1.A1_at	BG643920	AT2G23910.1	TC24476	V	0.21	-1.11	16.2	secondary metabolism.phenylpropanoids	(at2g23910): cinnamoyl-CoA reductase-related
Les.5848.1.A1_at	BG631837	AT1G65060.1	TC23527	I	1.22	0.74	16.2.1.3	secondary metabolism.phenylpropanoids.lignin biosynthesis.4CL	(at1g65060): 4CL3 (4-coumarate:CoA ligase 3); 4-coumarate-CoA ligase
Les.2988.1.S1_at	CK715255	AT2G30490.1	TC22567	I	1.09	-0.3	16.2.1.2	secondary metabolism.phenylpropanoids.lignin biosynthesis.C4H	(at2g30490): ATC4H (CINNAMATE-4-HYDROXYLASE)
LesAffx.37563.1.S1_at	Z70216.1	AT2G30490.1	TC23549	I	1.41	0.8	16.2.1.2	secondary metabolism.phenylpropanoids.lignin biosynthesis.C4H	(at2g30490): ATC4H (CINNAMATE-4-HYDROXYLASE)
Les.2934.2.S1_at	BG128445	AT4G39330.2	TC22376	V	-0.49	-2	16.2.1.10	secondary metabolism.phenylpropanoids.lignin biosynthesis.CAD	(at4g39330): mannitol dehydrogenase, putative
Les.3741.1.A1_at	AF146691.1	AT4G37990.1	TC23195	V	0.05	-2.34	16.2.1.10	secondary metabolism.phenylpropanoids.lignin biosynthesis.CAD	(at4g37990): ELI3-2 (ELICITOR-ACTIVATED GENE 3)
Les.206.1.S1_at	AW625821	AT4G34230.2	TC23716	IV	-1.28	0.42	16.2.1.10	secondary metabolism.phenylpropanoids.lignin biosynthesis.CAD	(at4g34230): CAD5 (CINNAMYL ALCOHOL DEHYDROGENASE 5); cinnamyl-alcohol dehydrogenase
LesAffx.34276.1.S1_at	BE433400	AT5G14700.1	TC22379	V	-1.15	-1.94	16.2.1.7	secondary metabolism.phenylpropanoids.lignin biosynthesis.CCR1	(at5g14700): cinnamoyl-CoA reductase-related
LesAffx.34986.1.A1_at	CK715664	AT5G54160.1	TC23304	V	-0.56	-1.46	16.2.1.9	secondary metabolism.phenylpropanoids.lignin biosynthesis.COMT	(at5g54160): ATOMT1 (O-METHYLTRANSFERASE 1)

LesAffx.60722.1.S1_at	AW092522	AT3G51970.1	TC22954	I	1.24	0.9	16.7	secondary metabolism.wax	(at3g51970): long-chain-alcohol O-fatty-acyltransferase family protein / wax synthase family protein
Les.971.1.A1_at	CK575003	AT1G68530.1	TC22472	V	-0.01	-1.54	16.7	secondary metabolism.wax	(at1g68530): CUT1 (CUTICULAR 1); acyltransferase
LesAffx.55337.1.S1_at	BM410261	AT1G25450.1	TC22580	V	-0.15	-1.52	16.7	secondary metabolism.wax	(at1g25450): very-long-chain fatty acid condensing enzyme, putative
LesAffx.15921.1.S1_at	BM412286	AT3G49050.1	TC23758	I	1.64	1.3	30.3	signalling.calcium	(at3g49050): lipase class 3 family protein / calmodulin-binding heat-shock protein, putative
Les.1977.1.S1_at	BG630657	AT2G45670.1	TC22750	VI	-1.1	-1.37	30.3	signalling.calcium	(at2g45670): calcineurin B subunit-related
Les.1997.1.S1_at	BE354113	AT1G08450.1	TC22008	VI	-1.03	-1.2	30.3	signalling.calcium	(at1g08450): CRT3 (CALRETICULIN 3); calcium ion binding
Les.5318.1.S1_at	BT013703.1	AT3G52870.1	TC24128	VI	-1.14	-1.89	30.3	signalling.calcium	(at3g52870): calmodulin-binding family protein
LesAffx.56.3.S1_at	BG132247	AT3G21180.1	TC23902	V	-0.42	-1.34	30.3	signalling.calcium	(at3g21180): ACA9 (autoinhibited Ca2+ -ATPase 9); calcium-transporting ATPase/ calmodulin binding
LesAffx.61165.1.S1_at	BM409862	AT5G62570.1	TC22968	V	-0.59	-1.05	30.3	signalling.calcium	(at5g62570): calmodulin-binding protein
LesAffx.9367.1.S1_at	CN385420	AT5G49480.1	TC22317	V	-0.17	-1.08	30.3	signalling.calcium	(at5g49480): ATCP1 (CA2+-BINDING PROTEIN 1); calcium ion binding
Les.5939.1.S1_at	CN385618	AT4G27280.1	TC23300	III	0.7	1.17	30.3	signalling.calcium	(at4g27280): calcium-binding EF hand family protein
Les.934.1.S1_at	BG628248	AT1G18210.2	GO37331	III	2.24	1.94	30.3	signalling.calcium	(at1g18210): calcium-binding protein, putative
LesAffx.11542.1.S1_at	BI922302	AT3G50770.1	TC21973	III	1.63	1.72	30.3	signalling.calcium	(at3g50770): calmodulin-related protein, putative
LesAffx.47666.1.S1_at	BE458190	AT4G34150.1	TC22130	III	0.71	1.06	30.3	signalling.calcium	(at4g34150): C2 domain-containing protein
LesAffx.57454.1.S1_at	BI923132	AT1G18530.1	TC22139	III	1.02	2.02	30.3	signalling.calcium	(at1g18530): calmodulin, putative
Les.4861.1.A1_at	BT012784.1	AT1G07410.1	TC23346	I	0.97	0.62	30.5	signalling.G-proteins	(at1g07410): AtRABA2b (Arabidopsis Rab GTPase homolog A2b); GTP binding
LesAffx.64861.1.S1_at	BG127249	AT2G31680.1	TC22312	I	1.01	0.8	30.5	signalling.G-proteins	(at2g31680): AtRABA5d (Arabidopsis Rab GTPase homolog A5d); GTP binding
Les.4873.1.S1_at	BT012806.1	AT2G43130.1	TC23935	II	0.33	1.53	30.5	signalling.G-proteins	(at2g43130): ARA4 (Arabidopsis Rab GTPase homolog A5c); GTP binding
Les.3564.1.A1_at	X55684.1	AT5G08650.1	TC24079	V	-0.62	-1.1	30.5	signalling.G-proteins	(at5g08650): GTP-binding protein LepA, putative
Les.176.1.S1_at	U38464.1	AT4G17530.1	TC21796	III	1.01	0.89	30.5	signalling.G-proteins	(at4g17530): RAB1C; GTP binding
Les.3623.1.S1_at	L12051.1	AT3G62560.1	TC22145	III	0.64	1.21	30.5	signalling.G-proteins	(at3g62560): GTP-binding protein, putative

LesAffx.62232.1.S1_at	AW649113	AT4G08950.1	TC23914	III	1.22	1.63	30.1.1	signalling.in sugar and nutrient physiology	(at4g08950): phosphate-responsive protein, putative (EXO)
LesAffx.59622.1.S1_at	BI210866	AT2G40080.1	TC22863	II	0.31	1.66	30.11	signalling.light	(at2g40080): ELF4 (EARLY FLOWERING 4)
Les.5791.1.S1_at	BT014572.1	AT5G48150.2	TC22816	VI	-1.32	-1.09	30.11	signalling.light	(at5g48150): PAT1 (PHYTOCHROME A SIGNAL TRANSDUCTION 1); transcription factor
Les.327.1.S1_at	BT014378.1	AT2G30520.3	TC22961	V	-0.32	-2.37	30.11	signalling.light	(at2g30520): RPT2 (ROOT PHOTOTROPISM 2); protein binding
Les.3488.1.S1_at	AF348461.1	AT4G08920.1	TC21724	V	-0.85	-1.41	30.11	signalling.light	(at4g08920): CRY1 (CRYPTOCHROME 1, OUT OF PHASE 2)
Les.2219.1.A1_at	BG735134	AT5G43310.1	TC23582	VI	-0.98	-1.36	30.11.1	signalling.light.COP9 signalosome	(at5g43310): COP1-interacting protein-related
Les.5152.1.S1_at	BT013393.1	AT4G01370.1	TC24469	III	1.16	1.23	30.6	signalling.MAP kinases	(at4g01370): ATMPK4 (MAP KINASE 4); MAP kinase/ kinase
LesAffx.53474.1.S1_at	BG133264	AT2G15320.1	TC22918	VI	-1.43	-1.33	30.8	signalling.misc	(at2g15320): leucine-rich repeat family protein
LesAffx.65678.1.S1_at	AW442323	AT1G53730.2	AW22314	VI	-1.31	-1.3	30.2.5	signalling.receptor kinases.leucine rich repeat V	(at1g53730): leucine-rich repeat transmembrane protein kinase, putative
LesAffx.46815.1.S1_at	BI934723	AT5G12940.1	TC21911	VI	-1.2	-0.98	30.2.11	signalling.receptor kinases.leucine rich repeat XI	(at5g12940): leucine-rich repeat family protein
Les.1951.1.A1_at	BG630440	AT3G19320.1	TC23845	IV	-2.07	-1.32	30.2.99	signalling.receptor kinases.misc	(at3g19320): leucine-rich repeat family protein
Les.3683.1.S1_at	AY093595.1	AT4G11650.1	TC24086	III	1.63	2.34	20.2	stress.abiotic	(at4g11650): ATOSM34 (OSMOTIN 34)
Les.4307.1.S1_at	AY257487.1	AT4G11650.1	TC21819	III	2.21	2.49	20.2	stress.abiotic	(at4g11650): ATOSM34 (OSMOTIN 34)
Les.5915.1.S1_at	AY344539.1	AT3G01420.1	TC22182	III	3.93	4.42	20.2	stress.abiotic	(at3g01420): ALPHA-DOX1 (ALPHA-DIOXYGENASE 1)
LesAffx.62570.2.A1_at	CD003040	AT4G00750.1	TC24016	I	1.9	1.36	20.2.3	stress.abiotic.drought/salt	(at4g00750): dehydration-responsive family protein
LesAffx.10495.1.S1_at	AW035719	AT4G16660.1	TC23875	I	1.04	0.81	20.2.1	stress.abiotic.heat	(at4g16660): heat shock protein 70, putative / HSP70, putative
LesAffx.68054.1.S1_at	AI898501	AT1G56300.1	TC22471	II	0.5	1.29	20.2.1	stress.abiotic.heat	(at1g56300): DNAJ heat shock N-terminal domain-containing protein
Les.4326.1.S1_at	AY235681.1	AT5G03160.1	TC22243	III	0.93	1.25	20.2.1	stress.abiotic.heat	(at5g03160): DNAJ heat shock N-terminal domain-containing protein
LesAffx.58365.1.S1_at	BM536079	AT3G08970.1	TC22511	III	1.13	1.18	20.2.1	stress.abiotic.heat	(at3g08970): DNAJ heat shock N-terminal domain-containing protein
LesAffx.805.1.S1_at	BI203776	AT3G62600.1	TC23079	III	1.18	1.49	20.2.1	stress.abiotic.heat	(at3g62600): DNAJ heat shock family protein
LesAffx.49201.1.S1_at	AW222231	AT5G63860.1	TC24148	VI	-0.74	-1.15	20.2.5	stress.abiotic.light	(at5g63860): UVR8 (UVB-RESISTANCE 8)

Les.3593.1.S1_at	BT012763.1	AT5G66400.1	TC23407	V	-0.13	-1.61	20.2.99	stress.abiotic.unspecified	(at5g66400): RAB18 (RESPONSIVE TO ABA 18)
LesAffx.23349.1.S1_at	AI782014	AT5G20630.1	TC21920	IV	-2.38	-0.39	20.2.99	stress.abiotic.unspecified	(at5g20630): GLP3 (GERMIN-LIKE PROTEIN 3); manganese ion binding / metal ion binding / nutrient reservoir
Les.4693.1.S1_at	M69247.1	AT1G50060.1	TC23231	II	0.08	1.94	20.1	stress.biotic	(at1g50060): pathogenesis-related protein, putative
Les.122.1.S1_at	Z15141.1	AT3G12500.1	TC22826	III	0.99	1.11	20.1	stress.biotic	(at3g12500): ATHCHIB (BASIC CHITINASE); chitinase
Les.2137.1.S1_at	AY359965.1	AT2G25470.1	TC21714	III	1.07	1.03	20.1	stress.biotic	(at2g25470): leucine-rich repeat family protein
Les.3619.1.S1_at	J04099.1	AT2G38870.1	TC23203	III	1.42	1.82	20.1	stress.biotic	(at2g38870): protease inhibitor, putative
Les.54.1.S1_at	AW218809	AT3G19690.1	TC22203	III	1.28	2.49	20.1	stress.biotic	(at3g19690): pathogenesis-related protein, putative
LesAffx.61012.1.S1_at	BF050668	AT3G49750.1	TC23271	III	0.88	1.21	20.1	stress.biotic	(at3g49750): leucine-rich repeat family protein
LesAffx.71065.1.S1_at	BM413117	AT1G78780.2	TC22472	III	1.77	1.89	20.1	stress.biotic	(at1g78780): pathogenesis-related family protein
LesAffx.66226.1.S1_at	BI926952	AT1G18250.1	TC22735	IV	-1.36	0.17	20.1	stress.biotic	(at1g18250): ATLP-1 (Arabidopsis thaumatin-like protein 1)
LesAffx.71532.1.S1_at	AI772343	AT1G33590.1	TC22607	I	1.15	0.92	20.1.7	stress.biotic.PR-proteins	(at1g33590): disease resistance protein-related / LRR protein-related
Les.5884.1.S1_at	CK716273	AT1G17860.1	TC21763	III	1.82	2.8	20.1.7.6.1	stress.biotic.PR-proteins.proteinase inhibitors.trypsin inhibitor	(at1g17860): trypsin and protease inhibitor family protein / Kunitz family protein
Les.26.1.S1_at	AF088276.1	AT1G64060.1	TC21861	III	2.05	3.27	20.1.1	stress.biotic.respiratory burst	(at1g64060): ATRBOH F (RESPIRATORY BURST OXIDASE PROTEIN F); NAD(P)H oxidase
Les.2817.1.S1_at	BF050945	AT1G10670.2	TC24089	I	1.1	0.75	8.2.11	TCA / org. transformation.other organic acid transformaitons.atp-citrate lyase	(at1g10670): ACLA-1 (ATP-citrate lyase A-1)
Les.5831.1.S1_at	BT013888.1	AT5G58330.2	TC21723	V	-0.72	-1.36	8.2.9	TCA / org. transformation.other organic acid transformaitons.cyt MDH	(at5g58330): malate dehydrogenase (NADP), chloroplast, putative
LesAffx.21904.1.S1_at	BM536146	AT1G44446.2	TC23302	V	-0.75	-1.67	19.16	tetrapyrrole synthesis.chlorophyll b synthase	(at1g44446): CH1 (CHLORINA 1); chlorophyll a oxygenase
Les.3322.1.S1_at	BI935557	AT5G13630.2	TC22083	V	-1.8	-3.14	19.1	tetrapyrrole synthesis.magnesium chelatase	(at5g13630): GUN5 (GENOMES UNCOUPLED 5)

Les.2924.1.S1_at	BT012789.1	AT4G18480.1	TC23389	IV	-1.09	-0.59	19.1	tetrapyrrole synthesis.magnesium chelatase	(at4g18480): CHLI1 (CHLORINA 42); magnesium chelatase
Les.3175.1.S1_at	BG626608	AT3G56940.1	TC23958	VI	-1.12	-1.29	19.12	tetrapyrrole synthesis.magnesium-protoporphyrin IX monomethyl ester (oxidative) cyclase	(at3g56940): AT103 (DICARBOXYLATE DIIRON 1)
Les.5120.1.S1_at	BT013322.1	AT4G27440.2	TC21818	VI	-1.48	-1.57	19.14	tetrapyrrole synthesis.protochlorophyllide reductase	(at4g27440): PORB (PROTOCHLOROPHYLLIDE OXIDOREDUCTASE B); oxidoreductase/ protochlorophyllide reductase
Les.3011.1.S1_at	BT012732.1	AT1G03630.1	TC23275	IV	-1.24	-0.79	19.14	tetrapyrrole synthesis.protochlorophyllide reductase	(at1g03630): POR C (PROTOCHLOROPHYLLIDE OXIDOREDUCTASE); NADPH dehydrogenase/ oxidoreductase/ protochlorophyllide reductase
Les.5850.1.S1_at	BT014189.1	AT4G27440.2	TC21894	IV	-1.93	-1.1	19.14	tetrapyrrole synthesis.protochlorophyllide reductase	(at4g27440): PORB (PROTOCHLOROPHYLLIDE OXIDOREDUCTASE B); oxidoreductase/ protochlorophyllide reductase
LesAffx.41330.1.A1_at	CK574967	AT3G59400.1	TC24064	IV	-1.8	-1.19	19.4	tetrapyrrole synthesis.regulation	(at3g59400): GUN4 (Genomes uncoupled 4)
Les.5221.1.S1_at	BT013523.1	AT5G43860.1	TC24473	IV	-1.23	-0.36	19.99	tetrapyrrole synthesis.unspecified	(at5g43860): ATCLH2 (Chlorophyll-chlorophyllido hydrolase 2)
Les.797.1.S1_at	X92854.1	AT4G13510.1	TC24488	III	1	1.3	34.5	transport.ammonium	MULTIPLE HITS: (at4g13510,at4g13505). at4g13510: AMT1;1 (AMMONIUM TRANSPORT 1); ammonium transporter at4g13505: other RNA
Les.34.1.S1_at	AF050496.1	AT4G00900.1	TC21713	VI	-1.06	-1.4	34.21	transport.calcium	(at4g00900): ECA2 ("calcium-transporting ATPase 2, endoplasmic reticulum-type"); calcium-transporting ATPase
LesAffx.23546.1.S1_at	BG126449	AT3G51860.1	TC22663	V	-0.69	-1.29	34.21	transport.calcium	(at3g51860): CAX3 (cation exchanger 3); cation:cation antiporter
LesAffx.16710.2.A1_at	AI899221	AT1G80760.1	TC23788	V	-0.16	-1.04	34.19.3	transport.Major Intrinsic Proteins.NIP	(at1g80760): NIP6;1 (NOD26-like intrinsic protein 6;1); water channel
Les.5610.1.S1_at	BT014251.1	AT1G01620.2	TC22162	IV	-1.25	-0.22	34.19.1	transport.Major Intrinsic Proteins.PIP	(at1g01620): PIP1C (PLASMA MEMBRANE INTRINSIC PROTEIN 1;3); water channel
Les.5960.1.S1_at	AY731066.1	AT4G17340.1	TC22652	II	0.39	1.9	34.19.2	transport.Major Intrinsic Proteins.TIP	(at4g17340): DELTA-TIP2/TIP2;2 (tonoplast intrinsic protein 2;2); water channel

Les.4953.1.S1_at	BT012976.1	AT4G01470.1	TC23170	VI	-1.05	-1.65	34.19.2	transport.Major Intrinsic Proteins.TIP	(at4g01470): GAMMA-TIP3/TIP1;3 (tonoplast intrinsic protein 1;3); water channel
LesAffx.286.2.S1_a_at	BG629460	AT2G36830.1	BP89354	VI	-1.08	-1.07	34.19.2	transport.Major Intrinsic Proteins.TIP	(at2g36830): GAMMA-TIP (Tonoplast intrinsic protein (TIP) gamma); water channel
LesAffx.52329.1.S1_at	BI927716	AT5G17520.1	BI92771	VI	-1.17	-1.25	34.8	transport.metabolite transporters at the envelope membrane	(at5g17520): RCP1 (ROOT CAP 1)
Les.4912.1.S1_at	BT012886.1	AT3G05290.1	TC22592	III	0.99	0.84	34.9	transport.metabolite transporters at the mitochondrial membrane	(at3g05290): mitochondrial substrate carrier family protein
LesAffx.59668.1.S1_at	BI208638	AT5G46800.1	TC24026	III	1.97	1.79	34.9	transport.metabolite transporters at the mitochondrial membrane	(at5g46800): BOU (A BOUT DE SOUFFLE); binding
LesAffx.62906.1.S1_at	BI923447	AT1G79900.1	TC23590	III	0.65	1.29	34.9	transport.metabolite transporters at the mitochondrial membrane	(at1g79900): ATMBAC2/BAC2 (Arabidopsis mitochondrial basic amino acid carrier 2); L-ornithine transporter/ binding / carnitine:acyl carnitine antiporter
LesAffx.67116.1.S1_at	BM413085	AT5G46800.1	TC21926	III	2.03	1.96	34.9	transport.metabolite transporters at the mitochondrial membrane	(at5g46800): BOU (A BOUT DE SOUFFLE); binding
Les.4844.1.S1_at	BI921923	AT5G24380.1	TC23893	V	-0.8	-1.52	34.12	transport.metal	(at5g24380): YSL2 (YELLOW STRIPE LIKE 2); oligopeptide transporter
Les.5530.1.S1_at	BT014086.1	AT5G03380.1	TC23075	III	2.12	1.82	34.12	transport.metal	(at5g03380): heavy-metal-associated domain-containing protein
LesAffx.41518.1.S1_at	AW930080	AT1G31260.1	TC23094	IV	-1.62	-0.92	34.12	transport.metal	(at1g31260): ZIP10 (ZINC TRANSPORTER 10 PRECURSOR); cation transporter
LesAffx.14321.1.S1_at	AW737808	AT5G49130.1	AW73780	VI	-1.48	-1.78	34.99	transport.misc	(at5g49130): MATE efflux family protein
LesAffx.3570.4.S1_at	AW624180	AT1G72160.1	TC22612	VI	-1.16	-0.82	34.99	transport.misc	(at1g72160): SEC14 cytosolic factor family protein / phosphoglyceride transfer family protein
LesAffx.64583.1.S1_at	BM409701	AT1G15170.1	TC22829	V	-0.19	-1.16	34.99	transport.misc	(at1g15170): MATE efflux family protein
LesAffx.68945.1.S1_at	BM413364	AT5G13750.1	TC23656	V	-0.12	-1.34	34.99	transport.misc	(at5g13750): ZIFL1 (ZINC INDUCED FACILITATOR-LIKE 1); tetracycline:hydrogen antiporter/ transporter
Les.4738.1.S1_at	BT013480.1	AT5G62890.3	GO37558	IV	-1.66	-0.74	34.99	transport.misc	(at5g62890): permease, putative
LesAffx.69264.1.S1_at	BF113833	AT5G50200.1	TC23903	IV	-1.54	-0.44	34.4	transport.nitrate	(at5g50200): WR3 (WOUND-RESPONSIVE 3)
Les.5670.1.S1_at	BT014359.1	AT4G18210.1	TC22878	IV	-1.34	-0.28	34.1	transport.nucleotides	MULTIPLE HITS: (at4g18220,at4g18210). at4g18220: purine permease family protein at4g18210: ATPUP10 (Arabidopsis thaliana purine permease 10); purine transporter

LesAffx.10279.1.A1_at	CK720579	AT1G28220.1	TC23575	IV	-1.66	-1.01	34.1	transport.nucleotides	(at1g28220): ATPUP3 (Arabidopsis thaliana purine permease 3); purine transporter
Les.3671.1.S1_at	M60166.1	AT5G62670.1	TC23533	VI	-0.92	-1.11	34.1	transport.p- and v-ATPases	(at5g62670): AHA11 (ARABIDOPSIS H(+)-ATPASE 11); ATPase
Les.3372.1.S1_at	BG631141	AT1G52190.1	TC23249	VI	-2.29	-2.24	34.13	transport.peptides and oligopeptides	(at1g52190): proton-dependent oligopeptide transport (POT) family protein
Les.5282.1.S1_at	BT013633.1	AT1G52190.1	BE46052	VI	-1.14	-0.83	34.13	transport.peptides and oligopeptides	(at1g52190): proton-dependent oligopeptide transport (POT) family protein
LesAffx.12670.3.S1_at	BF113197	AT2G38060.1	TC22012	I	1.11	0.29	34.7	transport.phosphate	(at2g38060): transporter-related
Les.2044.2.S1_at	AW443278	AT4G22200.1	TC23957	VI	-1.15	-1.12	34.15	transport.potassium	(at4g22200): AKT2 (Arabidopsis K+ transporter 2); cyclic nucleotide binding / inward rectifier potassium channel
LesAffx.52960.1.S1_at	BI207845	AT2G24240.1	TC23617	III	0.69	1.08	34.15	transport.potassium	(at2g24240): potassium channel tetramerisation domain-containing protein
Les.3479.1.S1_at	AF347614.1	AT1G22150.1	TC22134	VI	-1.06	-1.19	34.6	transport.sulphate	(at1g22150): SULTR1;3 (sulfate transporter); sulfate transporter
Les.3774.1.S1_at	AJ010942.1	AT5G26340.1	TC23863	I	1.38	0.57	34.2	transporter.sugars	(at5g26340): MSS1 (SUGAR TRANSPORT PROTEIN 13); carbohydrate transporter/ hexose:hydrogen symporter/ high-affinity hydrogen:glucose transporter/ sugar porter
Les.1784.1.S1_at	BI931777	AT3G05400.1	TC24140	VI	-1.44	-1.97	34.2	transporter.sugars	(at3g05400): sugar transporter, putative
LesAffx.51641.1.S1_at	BM535284	AT1G75220.1	TC23056	VI	-1.16	-0.95	34.2	transporter.sugars	(at1g75220): integral membrane protein, putative
