# Supplementary material for: Transcriptome Analysis of Tomato Flower Pedicel Tissues Reveals Abscission Zone-Specific Modulation of Key Meristem Activity Genes
Source: PLoS One. 2013 Feb 4;8(2):e55238. doi: 10.1371/journal.pone.0055238 (PMC3563536; doi:10.1371/journal.pone.0055238)
Supplement: Table S7 — The full list of the abscission zone-preferentially expressed genes. (PDF) [file pone.0055238.s009.pdf]

Table S7. The full list of AZ-preferentially expressed genes.

Affy_Probe Set ID	GenBank accession	Tomato Gene Index	Ratio		Raw reading			Annotation
			AZ:AP	AZ:BP	AP	AZ	BP	
Up regualted (68)								
LesAffx.39.1.S1_at	CN385433	TC22335	108.7	11.37	4.21	457.63	40.26	anionic peroxidase swpb2 [Ipomoea batatas]
Les.3646.1.S1_at	U70481.1	TC21735	68.41	5.1	14.13	966.66	189.61	polygalacturonase 4 [Lycopersicon esculentum]
Les.3693.1.S1_at	AF426174.1	TC22813	24.79	9.73	14.86	368.41	37.88	blind [Lycopersicon esculentum]
Les.3668.1.S1_at	U09026.1	TC21838	22.81	2.1	88.51	2019.17	963.19	lipoxygenase
LesAffx.31873.1.S1_at	BI928574	BI92857	22.63	4.53	21.54	487.5	107.55	unnamed protein product [Vitis vinifera]
Les.4938.1.S1_at	BT012940.1	TC22310	16.83	3.59	134	2255.02	627.99	unnamed protein product [Vitis vinifera]
Les.4136.1.S1_at	AJ538329.1	NP59756	16.62	15.32	61.15	1016.33	66.33	wuschel protein [Solanum lycopersicum]
Les.1175.2.S1_at	AI777697	FS18013	13.73	4.42	283.75	3897.03	881.16	PAR-1c [Nicotiana tabacum]
Les.3571.1.S1_at	U76408.1	NP00012	10.86	2.05	51.39	558.11	271.83	knotted 2 protein [Lycopersicon esculentum]
LesAffx.61901.1.S1_at	AW931428	AW93142	10.78	8.73	60.14	648.16	74.28	unnamed protein product [Vitis vinifera]
Les.4309.1.S1_at	AF375967.1	NP80337	10.61	4.29	30.54	323.88	75.47	bell-like homeodomain protein 4 [Lycopersicon esculentum]
Les.796.1.A1_at	BG627506	TC22633	10.56	2.27	145.36	1534.32	675.27	unknown
LesAffx.24134.1.A1_at	CK720539	CK72053	8.7	4.82	25.41	221.11	45.89	unnamed protein product [Vitis vinifera]
Les.2922.1.S1_at	BI924348	TC22195	8.6	5.55	241.84	2080.01	374.87	cytochrome P450 [Petunia x hybrida]
Les.69.1.S1_at	AF098674.1	TC21732	8	7.73	16.97	135.83	17.58	lateral suppressor protein [Lycopersicon esculentum]
Les.4976.1.S1_at	BT013033.1	TC22335	7.67	5.93	35.65	273.27	46.12	anionic peroxidase swpb2 [Ipomoea batatas]
LesAffx.66953.1.S1_at	AW651552	TC23604	6.8	2.31	508.24	3457.16	1494.43	unnamed protein product [Vitis vinifera]
LesAffx.66974.1.S1_at	BG135595	TC23429	6.26	3.7	16.87	105.59	28.51	hypothetical protein [Vitis vinifera]
Les.3645.1.S1_at	U54770.1	NP924378	5.39	3.89	668.12	3600.55	926.32	cytochrome P450 homolog [Lycopersicon esculentum]
Les.3625.1.S1_at	L22188.1	TC23715	5.06	2.12	121.3	613.25	288.8	RSI-1 protein
Les.3818.1.S1_at	AF502085.1	TC21752	4.92	2.14	38.97	191.84	89.52	ethylene responsive element binding protein [Lycopersicon esculentum]
Les.2278.1.S1_at	BT014352.1	TC24112	4.73	2.18	1119.56	5293.55	2433.11	flavanone 3 beta-hydroxylase [Solanum tuberosum]
LesAffx.63739.1.S1_at	AW033134	TC21965	4.72	2.93	104.08	491.15	167.45	hypothetical protein [Vitis vinifera]
LesAffx.39043.1.S1_at	AW032318	TC22833	4.5	2.25	53.57	240.95	106.94	hypothetical protein [Vitis vinifera]
Les.113.1.S1_at	CN384955	TC23130	4.44	2.1	443.47	1970.43	940.21	hypothetical protein MtrDRAFT_AC148340g20v2 [Medicago truncatula]
Les.4443.1.A1_s_at	BG630528	TC21730	4.39	3.23	927.52	4070.88	1260.97	cytochrome P450 homolog [Lycopersicon esculentum]
Les.3819.1.S1_at	AY140893.1	TC21842	4.3	3.34	260.44	1120.65	335.53	ovate protein [Lycopersicon esculentum]
LesAffx.59569.1.S1_at	BI203707	TC22974	4.27	2.09	987.43	4217.16	2016.93	basic blue copper protein [Cicer arietinum]
Les.3299.1.S1_at	BI933484	TC23916	4.23	2.28	60.96	257.62	113.11	unknown
LesAffx.30544.1.A1_at	AI489986	TC24194	3.96	2.13	427.35	1691.92	794.28	unnamed protein product [Vitis vinifera]

LesAffx.56130.1.S1_at	AW220490	TC24108	3.88	2.51	158.73	615.87	245.37	unnamed protein product [Vitis vinifera]
Les.3972.1.S1_at	AJ243340.1	TC22219	3.79	2.35	1093.82	4144.21	1761.08	expansin9 [Solanum lycopersicum]
Les.4527.1.S1_at	AJ635323.1	TC22837	3.72	2.78	32.68	121.72	43.78	polyphenol oxidase A [Solanum lycopersicum]
LesAffx.47221.1.S1_at	AW033464	AW03346	3.69	2.33	1055.85	3893.21	1671.76	unknown
LesAffx.62669.1.S1_at	AW442297	TC22170	3.62	2.1	217.24	786.86	374.61	LOB domain protein 1, putative [Solanum demissum]
LesAffx.56596.1.S1_at	AW036395	AW03639	3.46	4.04	43.62	151.14	37.4	unnamed protein product [Vitis vinifera]
LesAffx.62570.2.S1_at	CD003040	TC24016	3.46	2.52	137.49	476.04	188.59	Generic methyltransferase [Medicago truncatula]
LesAffx.36837.1.S1_at	AI780027	TC23516	3.24	2.84	107.67	348.35	122.62	unnamed protein product [Vitis vinifera]
LesAffx.57437.2.S1_at	AW621893	TC23850	3.16	3.1	71.04	224.38	72.47	salicylic acid-binding protein 2 [Nicotiana tabacum]
LesAffx.53517.1.S1_at	AI773309	TC23424	3.11	2.01	1062	3299.77	1645.64	peroxidase [Nicotiana tabacum]
LesAffx.10091.1.S1_at	AI487223	TC23911	3.05	2.05	156.42	477.66	233.04	hypothetical protein [Vitis vinifera]
Les.214.1.S1_at	AJ278331.1	TC21776	3.03	3.06	240.89	729.83	238.25	putative 12-oxophytodienoate reductase 2 [Solanum lycopersicum]
LesAffx.62933.1.S1_at	BE451393	BM41100	3.02	3.15	527.61	1591.16	504.4	inositol-1,4,5-triphosphate-5-phosphatase [Solanum lycopersicum]
LesAffx.470.1.S1_at	BI929841	TC23763	2.91	2.1	84.54	245.86	116.88	unnamed protein product [Vitis vinifera]
Les.3042.2.S1_at	AW934591	BE46335	2.87	4.99	58.64	168.13	33.7	hypothetical protein [Vitis vinifera]
Les.1841.1.S1_at	U72389.1	TC22336	2.8	2.06	83.95	234.74	114.13	1-aminocyclopropane-1-carboxylate synthase [Lycopersicon esculentum]
Les.1431.1.A1_at	BG631562	TC23902	2.74	2.17	297.93	816.63	376.32	unknown
Les.3630.1.S1_at	U50985.1	TC21720	2.73	4.6	82.18	224.43	48.78	pectin esterase [Lycopersicon esculentum]
Les.5855.1.S1_at	BT014384.1	TC22983	2.72	3.17	1523.49	4138.73	1305.81	hypothetical protein [Vitis vinifera]
LesAffx.24637.1.S1_at	CN550618	TC22796	2.71	4.5	275.88	748.34	166.48	unnamed protein product [Vitis vinifera]
LesAffx.69609.1.S1_at	AW929283	TC23404	2.66	2.71	211.3	562.96	207.57	salicylic acid/benzoic acid carboxyl methyltransferase [Datura wrightii]
LesAffx.62138.1.S1_at	AW737374	TC24364	2.63	2.7	110.1	289.36	107.14	myb-related transcription factor Cpm10 [Craterostigma plantagineum]
Les.726.1.S1_at	BG627133	TC24162	2.58	2.39	1068.14	2751.48	1152.21	Myosin heavy chain-like protein, putative [Solanum demissum]
LesAffx.295.2.S1_at	AI772896	TC22228	2.57	2.1	1020.61	2624.98	1250.44	cytochrome P450-dependent fatty acid hydroxylase [Nicotiana tabacum]
LesAffx.56802.1.S1_at	BI423210	TC21932	2.54	2.64	894.06	2268.16	858.64	hypothetical protein [Vitis vinifera]
LesAffx.63659.1.S1_at	BI921813	TC22794	2.53	2.47	379.53	962.09	389.26	photoperiod responsive protein [Solanum tuberosum subsp. andigena]
LesAffx.50112.1.S1_at	AI898985	TC23361	2.49	2.45	523.6	1305.03	531.96	photoperiod responsive protein [Solanum tuberosum subsp. andigena]
LesAffx.57010.1.S1_at	AI898096	TC21847	2.47	2.59	335.58	828.31	320.33	unnamed protein product [Vitis vinifera]
Les.3122.1.S1_a_at	BT013364.1	TC21726	2.44	3.5	44.25	107.99	30.86	pectin esterase [Lycopersicon esculentum]
LesAffx.51641.1.S1_at	BM535284	TC23056	2.34	2.48	49.64	116.16	46.79	integral membrane protein [Beta vulgaris]
LesAffx.19424.1.S1_at	BG128567	DB72515	2.29	2.46	161.34	368.85	149.91	unknown
LesAffx.65068.1.A1_at	CK716006	DV10465	2.29	2.13	564.47	1290.17	606.5	cytochrome P450 [Petunia x hybrida]
Les.3403.2.S1_at	BI925376	TC22472	2.27	2.09	1151.11	2609.09	1248.36	Protein phosphatase 2C-like [Medicago truncatula]
Les.4718.1.S1_at	BT013487.1	BI92390	2.25	3.27	180.74	406.87	124.31	unknown
Les.3486.1.S1_at	AF416289.1	TC22056	2.25	2.41	909.69	2043.41	846.89	auxin-regulated protein [Lycopersicon esculentum]
LesAffx.49935.1.S1_at	BF097557	TC23282	2.19	2.98	945.35	2067.63	694.82	pyruvate decarboxylase [Solanum tuberosum]

Les.2504.1.A1_at	BG628687	TC23404	2.16	2.58	576.19	1244.32	482.77	unknown
Les.3632.1.S1_at	U37840.1	TC21802	2.08	2.92	1593.72	3319.2	1135.43	lipoxygenase

Down regualted (39)

LesAffx.59706.1.S1_at	BI204252	TC23189	0.5	0.42	160.07	79.39	189.88	unnamed protein product [Vitis vinifera]
Les.1492.2.A1_at	BG626068	TC22441	0.49	0.37	316.16	155.07	423.82	unknown
Les.1230.1.A1_at	BG630221	GO37582	0.49	0.48	1251.09	612.23	1264.97	unknown
Les.228.1.S1_at	CK714929	TC23208	0.49	0.45	6130.51	2987.25	6619.26	putative proline-rich protein [Lycopersicon esculentum]
Les.1762.1.S1_at	AI780683	TC22523	0.49	0.27	275.64	134.09	488.28	H-protein [Flaveria cronquistii]
Les.4769.1.S1_at	BT012708.1	TC21756	0.49	0.44	1313.85	639.13	1459.83	expansin-like protein precursor [Lycopersicon esculentum]
Les.46.1.S1_at	X60760.1	NP00035	0.49	0.3	148.15	71.98	238.13	TDR8 [Solanum lycopersicum]
LesAffx.70088.1.S1_at	BI924873	TC22069	0.48	0.5	960.68	461.9	917.69	unknown
LesAffx.24799.2.S1_at	BI209670	TC24255	0.48	0.46	986.48	472.36	1037.1	unnamed protein product [Vitis vinifera]
LesAffx.69296.1.S1_at	AW092917	TC22654	0.47	0.46	2841.7	1334.34	2876.58	unnamed protein product [Vitis vinifera]
LesAffx.64757.1.S1_at	AI490042	TC22570	0.46	0.42	999.17	464.07	1100.56	unknown [Solanum tuberosum]
LesAffx.53966.1.S1_at	AY026037.1	TC23466	0.46	0.21	922.28	424.38	1999.71	glycine-rich protein TomR2 [Lycopersicon esculentum]
LesAffx.26489.1.S1_at	AW041597	TC21998	0.45	0.49	1050.24	476.88	983	chloroplast ribosomal protein L17 [Nicotiana tabacum]
Les.119.1.S1_at	AF218774.1	TC21978	0.45	0.47	1243.06	562.3	1198.07	putative water channel protein [Lycopersicon esculentum]
Les.4412.1.A1_at	BG627732	TC23503	0.45	0.5	1315.17	592.24	1187.84	unnamed protein product [Vitis vinifera]
Les.4459.1.S1_s_at	X99148.1	ES89373	0.45	0.27	1247.7	555.84	2037.58	putative arabinogalactan-protein [Solanum lycopersicum]
LesAffx.6110.1.S1_at	BF098450	TC22721	0.44	0.24	79.16	34.47	141.37	hypothetical protein [Vitis vinifera]
Les.24.1.S1_at	AF191823.1	TC21735	0.42	0.49	473.73	201.02	410.44	alpha-galactosidase [Lycopersicon esculentum]
LesAffx.63489.1.S1_at	BI921137	TC22082	0.42	0.5	654.36	276.9	555.19	unnamed protein product [Vitis vinifera]
Les.1721.2.S1_at	BF113580	TC22831	0.41	0.37	140.69	58.04	156.6	expressed protein [Oryza sativa (japonica cultivar-group)]
LesAffx.29104.1.S1_at	AW096456	TC21791	0.39	0.38	938.5	368.99	964.82	hypothetical protein OsI_008518 [Oryza sativa (indica cultivar-group)]
LesAffx.37344.1.S1_at	AI897449	TC21881	0.39	0.26	471.68	185.18	700.33	acetylajmalan acetylesterase [Rauvolfia serpentina]
Les.3594.1.S1_at	Z47980.1	TC21807	0.39	0.25	2402.16	927.9	3702.33	unknown
LesAffx.64062.1.S1_at	AW040768	TC22080	0.38	0.44	133.11	50.74	114.2	germin-like protein [Capsicum chinense]
LesAffx.105.1.S1_at	CK715483	TC22543	0.38	0.25	184.61	69.65	276.45	unnamed protein product [Vitis vinifera]
LesAffx.33890.1.S1_at	BG134582	TC22584	0.37	0.32	160.45	60.11	190.11	unnamed protein product [Vitis vinifera]
Les.3995.1.S1_at	AW443014	TC21771	0.37	0.29	2367	882.06	3091.19	methionine rich arabinogalactan [Solanum lycopersicum]
Les.2286.1.S1_at	BT013195.1	TC22726	0.37	0.4	2309.24	855.95	2166.58	unnamed protein product [Vitis vinifera]
Les.144.1.S1_at	Z75519.1	TC22816	0.35	0.34	600.28	208.98	607.78	unknown [Solanum lycopersicum]
Les.3436.2.S1_a_at	CN385216	TC22872	0.34	0.39	9345.71	3173.89	8087.23	metallocarboxypeptidase inhibitor [Solanum tuberosum]
Les.479.1.S1_at	AY509122.1	TC22844	0.33	0.31	418.47	137.28	439.55	arachidonic acid-induced DEA1 [Lycopersicon esculentum]

Les.3065.1.S1_at	BI929558	TC22543	0.32	0.5	965.19	309.75	618.07	squamosa promoter binding protein-like protein [Capsicum annuum]
Les.208.1.S1_at	U44386.1	TC22299	0.31	0.45	2359.05	739.17	1642.75	ORF
LesAffx.53591.1.S1_at	AI899018	TC22292	0.31	0.5	390.02	121.56	242.3	methyl jasmonate induced MYB-related transcription factor [Nicotiana tabacum]
Les.3620.1.S1_at	L26295.1	TC21804	0.31	0.47	335.2	102.7	220.81	TAG1
Les.4896.1.S1_at	BT012857.1	TC23109	0.28	0.25	2173.94	609.73	2404.3	allergen-like protein BRSn20 [Sambucus nigra]
LesAffx.26180.1.S1_at	BG132890	BG13289	0.21	0.37	272.21	58.23	158.97	hypothetical protein [Vitis vinifera]
Les.2460.1.S1_at	AW218727	TC22357	0.16	0.12	1966.34	313.46	2614.26	specific tissue protein 2 [Cicer arietinum]
Les.4493.3.S1_at	CN385077	TC23105	0.16	0.48	595.29	94.18	194.7	hypothetical protein [Plantago major]
